# *N*‐Heterocyclic Carbene Organocatalysis Enabled Modular Synthesis of Fluorinated Isoflavonoids to Suppress Proliferation and Migration in Breast Cancer Cells

**DOI:** 10.1002/advs.202413851

**Published:** 2025-02-13

**Authors:** Yan‐Qing Liu, Lei‐Lei Fu, Long‐Hai Hong, Xin‐Xin Kou, Xiang Zhang, Rong Zeng, Yong‐Qi Zhen, Bo Han, Jun‐Long Li

**Affiliations:** ^1^ Anti‐infective Agent Creation Engineering Research Centre of Sichuan Province Sichuan Industrial Institute of Antibiotics School of Pharmacy Chengdu University Chengdu 610106 China; ^2^ State Key Laboratory of Southwestern Chinese Medicine Resources School of Pharmacy Chengdu University of Traditional Chinese Medicine Chengdu 611137 China; ^3^ Sichuan Engineering Research Center for Biomimetic Synthesis of Natural Drugs School of Life Science and Engineering Southwest Jiaotong University Chengdu 610031 China; ^4^ Department of Pharmacy the Thirteenth People's Hospital of Chongqing Chongqing Geriatrics Hospital Chongqing 400053 China

**Keywords:** anti‐breast cancer, cascade intramolecular annulation, fluorinated isoflavonoids, NHC organocatalysis, radical acylalkylation

## Abstract

Isoflavonoids represent a privileged scaffold among various bioactive natural products, rendering their structural diversification through green synthesis and subsequent biological evaluations a compelling research area. In this study, an NHC organocatalytic radical acylalkylation of 1,3‐enynes using salicylaldehydes is presented, followed by a cascade intramolecular annulation, yielding a series of fluorinated isoflavone derivatives with substantial yields under environmental‐friendly conditions. This approach, distinguished by its excellent modularity and high functional group tolerance, represents an unprecedented organocatalytic 1,3,4‐trifunctionalization of 1,3‐enynes designed for the green synthesis of bioactive isoflavones in a single step. Furthermore, it is demonstrated that these synthesized fluorinated isoflavonoids effectively suppress proliferation in breast cancer cells, with the most potent compound 8 also inhibiting migration in MDA‐MB‐231 cells.

## Graphical abstract

An NHC‐catalyzed transformation is successfully developed to modularly synthesize novel fluorinated isoflavone derivatives under environmental‐friendly conditions. This approach represents an unprecedented organocatalytic 1,3,4‐trifunctionalization of 1,3‐enynes designed for the green synthesis of bioactive isoflavones in a single step. Additionally, we demonstrated that these synthesized fluorinated isoflavonoids effectively suppress proliferation in breast cancer cells, with the most potent compound **8** also inhibiting migration in MDA‐MB‐231 cells.

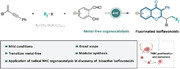

## 1 Introduction

**Figure 1:**
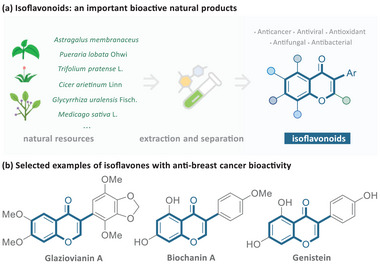
Isoflavonoid as a privileged scaffold among various natural products and examples of anticancer isoflavonoids.

Isoflavonoids, a class of plant secondary metabolites, are typically obtained through advanced separation technologies. These high‐value compounds are widely distributed in various natural plants, including *Astragalus membranaceus*, *Pueraria lobata* Ohwi, and *Glycyrrhiza uralensis* Fisch.^[^ ^1^ ^]^ Extensive research has demonstrated that many isoflavonoid‐containing natural products possess a wide array of bioactive properties, such as antioxidant and antiosteoporosis effects, as well as notable anticancer activity (**Figure** **1**a).^[^ ^2^ ^]^ For instance, Glaziovianin A, Biochanin A, and Genistein, isolated from the leaves of *Ateleia glazioviana*, soybean, and Psoralea corylifolia, respectively, have been identified as potent agents against breast cancer (Figure 1b).^[^ ^3^ ^]^ Due to their diverse biological activities, isoflavonoids have garnered substantial interest in both medicinal and synthetic chemistry communities.

**Figure 2:**
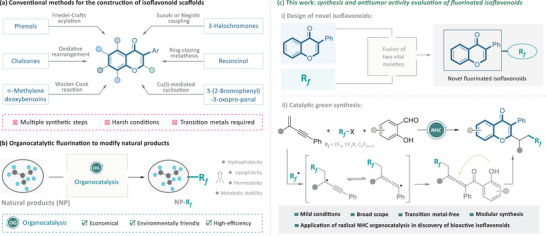
Research motivation for the modular synthesis of fluorinated isoflavones through NHC organocatalysis.

In addition to extraction from natural sources, isoflavonoids have also been synthesized by organic chemists using a variety of methods. Conventional synthetic strategies for creating an isoflavonoid scaffold include Friedel‐Crafts acylation of phenols,^[^ ^4^ ^]^ ring‐closing metathesis,^[^ ^5^ ^]^ oxidative rearrangement of chalcones,^[^ ^6^ ^]^ Suzuki or Negishi cross‐coupling of 3‐halochromones,^[^ ^7^, ^8^ ^]^ Wacker–Cook tandem reaction with α‐methylene deoxybenzoins,^[^ ^9^ ^]^ Cu‐catalyzed cyclization of 3‐(2‐bromophenyl)‐3‐oxopro‐panal,^[^ ^10^ ^]^ and others.^[^ ^11^ ^]^ These traditional methods, however, often involve complex, multi‐step processes, requiring harsh conditions and hazardous reagents, and offer limited functional group tolerance. Additionally, the use of transition metal catalysts can leave residual metals in the final products, posing contamination risks (**Figure** **2**a).^[^ ^12^ ^]^ Therefore, developing green synthetic methods that are efficient, environmentally friendly, and capable of operating under mild conditions with readily available materials is highly desirable.

In recent years, the incorporation of fluorine atoms into drug candidates has become increasingly prevalent.^[^ ^13^ ^]^ Fluorinated natural products, in particular, show enhanced hydrophobicity, lipophilicity, permeability, bioactivity, and structural stability, making them more promising as potential pharmaceuticals.^[^ ^14^ ^]^ This fluorination process is typically achieved through various catalytic strategies. Among these, organocatalysis stands out for its high efficiency, cost‐effectiveness, and environmental friendliness (Figure 2b).

Given the critical biological roles of isoflavonoids and the therapeutic advantages of fluorinated compounds, designing and synthesizing hybrid molecules that combine these two elements via organocatalysis could provide a foundation for discovering new and potent bioactive substances. This molecular design and catalytic synthesis strategy hold promise for accelerating drug discovery by creating compounds with enhanced medicinal properties. (Figure 2c–i). Recently, *N*‐heterocyclic carbene (NHC) organocatalytic radical reactions^[^ ^15^ ^]^ using various fluoroalkyl sources, such as fluoroalkyl iodides and Togni reagents, have provided a versatile and effective means of incorporating fluorinated groups into bioactive compounds. These methods offer a promising route to create fluorinated molecules with significant pharmaceutical potential.^[^ ^16^ ^]^ While 1,3‐enynes have emerged as valuable substrates for generating medicinally relevant molecules,^[^ ^17^ ^]^ the direct conversion of 1,3‐enynes into diverse bioactive isoflavonoids has not been fully explored. Building on our ongoing research into NHC‐catalyzed radical reactions for constructing drug‐like molecules, we report an NHC‐catalyzed transformation that involves 1,3‐enynes, salicylaldehydes, and various fluoroalkyl sources to modularly synthesize novel fluorinated isoflavone derivatives (Figure 2c). This reaction proceeds via radical acylalkylation of 1,3‐enynes followed by a cascade intramolecular annulation of functionalized allenic ketones, ultimately yielding a collection of fluorinated isoflavonoids. Our research demonstrates that these newly synthesized isoflavonoid derivatives exhibit strong antiproliferative and antimigratory effects on breast cancer cell lines, underscoring their potential as novel therapeutic agents. This organocatalytic approach not only expands the chemical space of isoflavonoids but also provides a green and efficient pathway for developing new bioactive compounds.

## 2 Results and Discussion

We initiated our synthetic study by employing 1,3‐enyne **1a**, salicylaldehyde **2a**, and Togni I reagent as model substrates to verify the feasibility of the NHC organocatalytic 1,3,4‐tri‐functionalization of 1,3‐enynes. To our gratification, the reaction could smoothly provide the desired product **4a** in the presence of NHC precatalyst **3a** with Cs_2_CO_3_ as the base at 60 °C in PhCF_3_, albeit with an inferior yield (**Table** **1**, entry 1). To improve the reaction efficiency, various thiazolium‐based NHC precursors were subsequently investigated. Fortunately, the use of *N*‐2,6‐disubstituted phenyl NHC catalyst **3b** and **3c** dramatically increased the yield to 68% and 70%, respectively (entries 2–3). A similar result was obtained when cyclohexane‐fused thiazolium NHC precursor **3d** was used as the catalyst (entry 4). However, cyclopentane‐fused thiazolium **3e** or the commercially available *N*‐methyl or benzyl thiazolium salts **3f** and **3g** failed to catalyze the reaction in an efficient manner (entries 5–7). No better results were achieved by employing triazolium or imidazolium‐based NHCs **3h**−**3k** (entries 8–11). Further reaction condition optimization, including changing bases (entries 12−16) and solvents (entries 17−21), as well as lowering temperature (entry 22) led to inferior results.

**Table 1:** Optimization of the NHC‐catalyzed trifunctionalization of 1,3‐enynes.

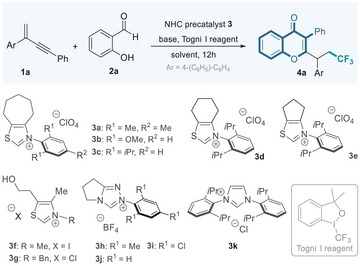					
Entry^a)^	NHC 3	Base	Solvent	T (°C)	Yield (%)^b)^
1	**3a**	Cs_2_CO_3_	CF_3_Ph	60	30
2	**3b**	Cs_2_CO_3_	CF_3_Ph	60	68
3	**3c**	Cs_2_CO_3_	CF_3_Ph	60	70
4	**3d**	Cs_2_CO_3_	CF_3_Ph	60	69
5	**3e**	Cs_2_CO_3_	CF_3_Ph	60	<5
6	**3f**	Cs_2_CO_3_	CF_3_Ph	60	9
7	**3g**	Cs_2_CO_3_	CF_3_Ph	60	11
8	**3h**	Cs_2_CO_3_	CF_3_Ph	60	<5
9	**3i**	Cs_2_CO_3_	CF_3_Ph	60	17
10	**3j**	Cs_2_CO_3_	CF_3_Ph	60	<5
11	**3k**	Cs_2_CO_3_	CF_3_Ph	60	<5
12	**3c**	K_2_CO_3_	CF_3_Ph	60	68
13	**3c**	K_3_PO_4_	CF_3_Ph	60	43
14	**3c**	Et_3_N	CF_3_Ph	60	49
15	**3c**	DBU	CF_3_Ph	60	19
16	**3c**	DABCO	CF_3_Ph	60	68
17	**3c**	Cs_2_CO_3_	DCM	60	64
18	**3c**	Cs_2_CO_3_	MeCN	60	57
19	**3c**	Cs_2_CO_3_	DMSO	60	<5
20	**3c**	Cs_2_CO_3_	acetone	60	47
21	**3c**	Cs_2_CO_3_	*i‐*Pr_2_O	60	64
22	**3c**	Cs_2_CO_3_	CF_3_Ph	40	63

^a)^ The reactions were carried out with **1a** (0.15 mmol), aldehyde **2a** (0.10 mmol), NHC **3** (0.02 mmol), base (0.04 mmol), and Togni I reagent (0.15 mmol) in solvent (1.0 mL) for 12h;

^b)^ Isolated yield of **4a**.

With the established optimal conditions in hand, we investigated the generality and limitation of this organocatalytic radical trifunctionalization of 1,3‐enynes to construct a collection of novel fluorinated isoflavone derivatives. First, we tested the substrate scope of salicylaldehydes. As summarized in **Table** **2**, various salicylaldehydes **2** bearing electron‐withdrawing or electron‐donating groups at the 4‐ or 5‐positions on the aromatic ring reacted smoothly with 1,3‐enyne **1a** and Togni reagent, delivering the desired isoflavones **4a**−**4g** in 62%−89% isolated yields. Additionally, 3‐substituted benzyaldehydes were well‐tolerated, providing **4h**−**4i** in 62%−78% yields. Notably, the 1‐naphthol‐2‐carboxaldehyde also participated in this transformation smoothly, yielding the polycyclic fluorinated isoflavone product **4j** in 41% yield.

**Table 2:** Salicylaldehyde scope of the organocatalytic radical synthesis of isoflavonoids.^a)^

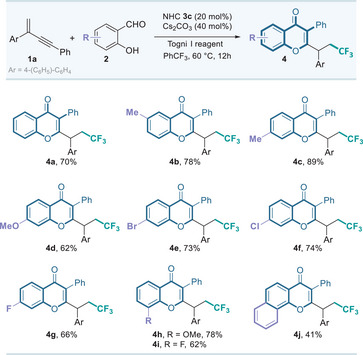

^a)^ The reactions were carried out with **1a** (0.15 mmol), aldehyde **2** (0.10 mmol), NHC **3c** (0.02 mmol), Cs_2_CO_3_ (0.04 mmol), and Togni I reagent (0.15 mmol) in PhCF_3_ (1.0 mL) for 12h; isolated yield.

Evaluation of 1,3‐enyne substrates was subsequently carried out. As shown in **Table** **3**, aryl substituents on the alkene moiety of 1,3‐enynes **1**, including *para*‐, *meta*‐, and *ortho*‐substituted groups with electron‐withdrawing or electron‐donating properties, were compatible with this catalytic system. affording the fluorinated isoflavones **4k**−**4r** in good yields. Furthermore, 1,3‐enyne possessing 1,3‐benzodioxole motif also proceeded smoothly, producing **4s** in 42% yield. To our gratification, the alkyl substituted substrate is also suitable for this reaction, affording the corresponding product **4t** in 46% isolated yield. However, substrates with aromatic heterocyclic R^1^ groups, such as furyl, thienyl or pyridyl, failed to deliver the desired products. It is noteworthy that several fluorinated isoflavone derivatives **4u**−**4z** containing multiple methoxy groups as the hydrogen bond receptors could also be smoothly synthesized, which might be attractive in biological evaluation. Importantly, the structural correctness of a representative product **4n** was confirmed via single‐crystal X‐ray diffraction analysis.

**Table 3:** 1,3‐Enyne scope of the organocatalytic radical synthesis of isoflavonoids.^a)^

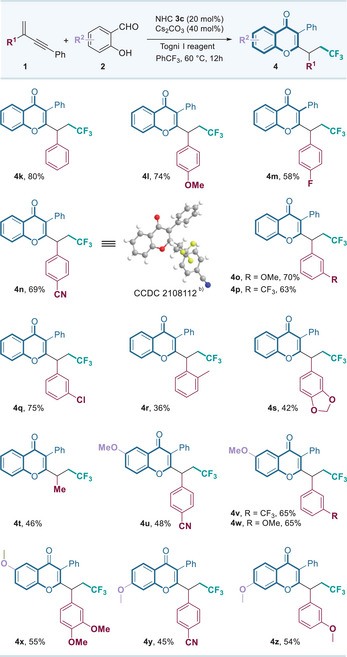

^a)^ The reactions were carried out with **1** (0.15 mmol), aldehyde **2** (0.10 mmol), NHC **3c** (0.02 mmol), Cs_2_CO_3_ (0.04 mmol) and Togni I reagent (0.15 mmol) in PhCF_3_ (1.0 mL) for 12h; isolated yield;

^b)^ The structure of **4n** were determined by X‐ray analysis, and other products were assigned by analogy.^[^ ^18^ ^]^

We further explored the use of alternative fluoroalkyl sources in this catalytic system, expanding its utility. For instance, methyl bromodifluoroacetate was successfully employed, delivering the difluoromethyl isoflavone **8** in 57% yield. Similarly, perfluoroalkyl iodides were amenable to the reaction, yielding perfluorinated isoflavone derivatives **9**–**10** in reasonable yields. These results, summarized in **Table** **4**, demonstrate the flexibility of this system in accommodating diverse fluoroalkyl sources and producing a range of fluorinated isoflavones with potential synthetic and biological value.

**Table 4:** Various fluoroalkyl sources applied in the organocatalytic radical synthesis of isoflavonoids.^a)^

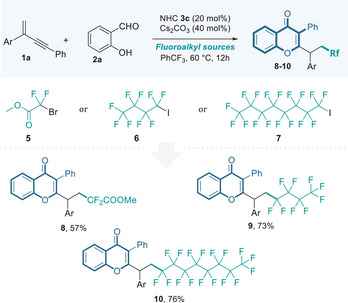

^a)^ The reactions were carried out with **1a** (0.15 mmol), aldehyde **2a** (0.10 mmol), NHC **3c** (0.02 mmol), Cs_2_CO_3_ (0.15 mmol), and other fluoroalkyl sources (0.15 mmol) in CF_3_Ph (1.0 mL) for 12h; isolated yield.

**Figure 3:**
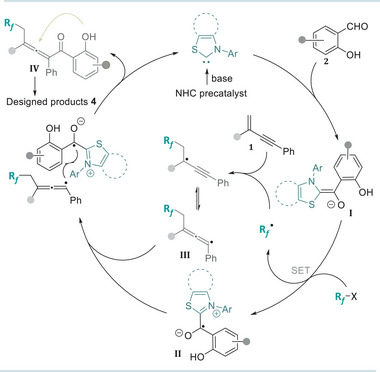
Proposed mechanism of the NHC‐catalyzed radical synthesis of fluorinated isoflavonoids.

A plausible mechanism for this NHC‐catalyzed radical acylalkylation of 1,3‐enynes and cascade annulation was proposed based on our previous reports.^[^ ^16a,f^ ^]^ As shown in **Figure** **3**, the deprotonated Breslow intermediate **I** was generated in situ from the NHC catalyst and salicylaldehydes **2**. Then, single‐electron reduction of the fluoroalkyl reagents by **I** produced a fluoroalkyl radical and a persistent ketyl radical **II**. Addition of fluoroalkyl radical to the 1,3‐enynes **1** and subsequent isomerization gave the allenyl radical **III**, which was recombined with radical **II** to deliver the allenic ketones **IV** to release the carbene catalyst. Finally, the intramolecular annulation of **IV** gave the desired fluorinated isoflavonoids **4**.

**Figure 4:**
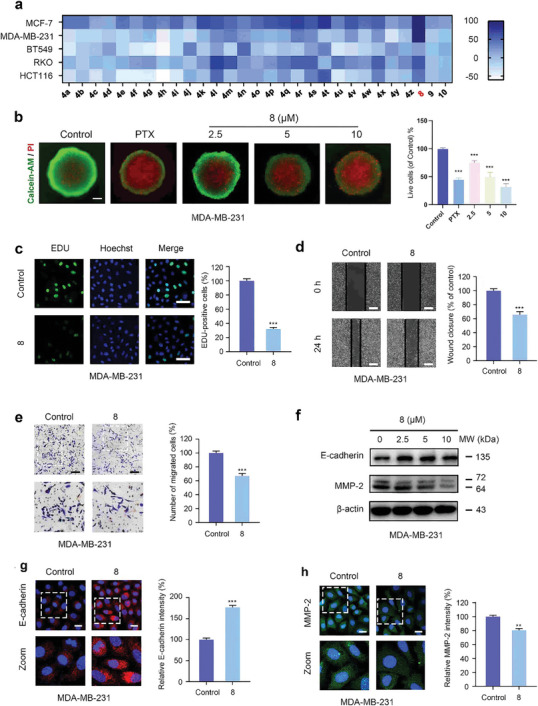
Product **8** inhibits cell proliferation and migration in MDA‐MB‐231 cells. a) Heat map of inhibition rates of isoflavone fluorinated products in multiple tumor cell lines and normal breast epithelial cell at a concentration of 30 µm for 24 h. b) 3D cultures of MDA‐MB‐231 cells were treated with 10 µm Paclitaxel (PTX) and 5 µm compound **8**, followed by Calcein/PI staining. Representative images and quantification of 3D‐spheroid size were shown. Scale bar, 100 µm. c) Edu assay of MDA‐MB‐231 cells treated with or without product **8** (5 µm). Representative images and quantification of Edu‐positive cells were shown. Scale bar, 50 µm. d) The scratch assay of MDA‐MB‐231 cells treated with or without product **8** (5 µm). The wound closure ratio represents the level of cell migration ability. Representative images and statistics were shown. Scale bar, 100 µm. e) Transwell assay was used to measure the number of migrated cells. Representative images and statistics were shown Scale bar, 50 µm. f) Immunoblotting analysis of E‐cadherin and MMP‐2 expression in MDA‐MB‐231 cells treated with 0, 2.5, 5, and 10 µm of product **8** for 24 h. β‐actin was used as a loading control. (g‐h) The expression of E‐cadherin and MMP‐2 were analyzed by immunofluorescence in MDA‐MB‐231 cells with or without product **8** (5 µm). Scale bar, 10 µm. Representative images and statistics were shown. Data represent mean ± SD. ^*^ *p* < 0.05, ^**^ *p* < 0.01, ^***^ *p* < 0.001 compared with the control groups.

Since previous reports indicated that several natural isoflavonoids exhibited significant anti‐tumor bioactivity,^[^ ^19^ ^]^ we therefore examined inhibitory activity of our synthetic fluorinated isoflavonoids against cancer cells. Initially, MTT assays were performed to evaluate the proliferation of tumor cell lines, including MDA‐MB‐231, BT549, MCF‐7, RKO, and HCT‐116 cells, following treatment with fluorinated isoflavone products. We found that product **8** presents a high inhibitory rate in TNBC lines and has no toxic effect at a concentration of 30 µm in MCF‐10A cells and other tumor cell lines (**Figure** **4**a). Notably, the MDA‐MB‐231 and MCF‐7 cells were more sensitive to product **8** (*IC*_50_ values of 5.4 and 8.3 µm, respectively) (for detail, see: Figure S1a, Supporting Information). Further evaluation in the MDA‐MB‐231 cell line revealed that the activity of 8 at a concentration of 10 µm surpassed that of paclitaxel (for detail, see: Figure S1b, Supporting Information).

To further investigate the anti‐tumor effects of product **8**, we evaluated the proliferation of MDA‐MB‐231 cells following treatment with **8**. The results demonstrated that product **8** significantly reduced the number of colonies (for detail, see: Figure S2a, Supporting Information). Consistent with the colony formation assay, the 3D spheroid assay utilizing Calcein‐AM/PI staining (Figure 4b) similarly indicated that product **8** decreases tumor cell viability, thereby reinforcing its role in inhibiting TNBC cell proliferation. Additionally, treatment with product **8** led to a decrease in the colocalization of EdU with Hoechst (Figure 4c), further confirming the suppression of DNA synthesis and cell proliferation in TNBC cells. Collectively, these findings reveal that product **8** effectively inhibits cell proliferation in TNBC cells. Subsequent scratch assays demonstrated a reduced wound closure ratio in MDA‐MB‐231 cells treated with product **8** (Figure 4d), indicating impaired cell migration. This inhibitory effect on cell migration was further corroborated by transwell assays, which showed a significant decrease in the number of migrated TNBC cells upon treatment with product **8** (Figure 4e). At the molecular level, immunofluorescence and western blot analyses revealed that product **8** treatment resulted in the upregulation of E‐cadherin and the downregulation of MMP‐2 expression (Figure 4f–h; Figure S2b, Supporting Information). The upregulation of E‐cadherin suggests an enhancement of cell‐cell adhesion, while the downregulation of MMP‐2 indicates a reduction in extracellular matrix degradation, both of which are critical factors in inhibiting cancer cell migration and metastasis. Taken together, these results imply that product **8** not only inhibits the proliferation of TNBC cells but also impedes their migratory capabilities, highlighting its potential as a promising therapeutic agent for the treatment of triple‐negative breast cancer.

## 3 Conclusion

In conclusion, we developed an NHC‐catalyzed three‐component reactions for the modular synthesis of fluorinated isoflavonoids, providing a novel and green strategy for the rapid construction of medicinally interesting molecules. This appears to be the first organocatalytic radical 1,3,4‐trifunctionalization of 1,3‐enynes for the synthesis of drug‐like molecules inspired by natural products. Importantly, we demonstrated that our synthesized fluorinated isoflavonoids could suppress proliferation in breast cancer cell lines. The most potent isoflavonoid **8** could also inhibit cell proliferation and migration in MDA‐MB‐231 cells. In future research, we plan to employ high‐throughput screening and molecular docking techniques to identify specific targets and optimize compound structures, further investigating structure‐activity relationships and enhancing their therapeutic potential.

## Conflict of Interest

The authors declare no conflict of interest.

## Supporting information



Supporting Information

## Data Availability

The data that support the findings of this study are available from the corresponding author upon reasonable request.
