# Supplementary material for: N‐Heterocyclic Carbene Organocatalysis Enabled Modular Synthesis of Fluorinated Isoflavonoids to Suppress Proliferation and Migration in Breast Cancer Cells
Source: Adv Sci (Weinh). 2025 Feb 13;12(13):2413851. doi: 10.1002/advs.202413851 (PMC11967825; doi:10.1002/advs.202413851)
Supplement: Supplementary file 1 — Supporting Information [file ADVS-12-2413851-s001.pdf]

ADVANCED SCIENCE
Open Access

Supporting Information

for *Adv. Sci.*, DOI 10.1002/advs.202413851

N-Heterocyclic Carbene Organocatalysis Enabled Modular Synthesis of Fluorinated Isoflavonoids to Suppress Proliferation and Migration in Breast Cancer Cells

Yan-Qing Liu, Lei-Lei Fu, Long-Hai Hong, Xin-Xin Kou, Xiang Zhang, Rong Zeng, Yong-Qi Zhen, Bo Han and Jun-Long Li**

N-Heterocyclic Carbene Organocatalysis Enabled Modular Synthesis of Fluorinated Isoflavonoids to Suppress Proliferation and Migration in Breast Cancer Cells

Yan-Qing Liu,[1,2,4,#] Lei-Lei Fu,[3,#] Long-Hai Hong,[1] Xin-Xin Kou,[1] Xiang Zhang,[1] Rong Zeng,[1] Yong-Qi Zhen,[3] Bo Han,[2,*] and Jun-Long Li,[1,2,*]

[1]*Anti-infective Agent Creation Engineering Research Centre of Sichuan Province, Sichuan Industrial Institute of Antibiotics, School of Pharmacy, Chengdu University, Chengdu 610106, China.*
[2]*State Key Laboratory of Southwestern Chyinese Medicine Resources, School of Pharmacy, Chengdu University of Traditional Chinese Medicine, Chengdu 611137, China.*
[3]*Sichuan Engineering Research Center for Biomimetic Synthesis of Natural Drugs, School of Life Science and Engineering, Southwest Jiaotong University, Chengdu 610031, China.*
[4]*Department of Pharmacy, the Thirteenth People's Hospital of Chongqing, Chongqing Geriatrics Hospital, Chongqing 400053, China*

[†]These authors contributed equally to this work.
*Corresponding authors.
E-mail addresses: lijunlong709@hotmail.com; hanbo@cdutcm.edu.cn

Supplementary Information

Table of Contents

1. **General Information** 2
2. **Further Optimization Studies** 4
3. **General Procedure for the Synthesis of Isoflavone Fluorinated Derivatives** 6
4. **Crystal Data and Structure Refinement for 4n** 26
5. **Biological Procedures** 28
6. **References and Notes** 32
7. **Copies of ^{1}H, ^{13}C and ^{19}F NMR Spectra** 33

1. General Information

General Procedures. All reactions were performed in oven-dried or flame-dried reaction vessels, modified Schlenk flasks, or round-bottom flasks. The flasks were fitted with Teflon screw caps and reactions were conducted under an atmosphere of argon if needed. Gas-tight syringes with stainless steel needles were used to transfer air- and moisture-sensitive liquids. All moisture and/or air sensitive solid compounds were manipulated inside normal desiccators. Flash column chromatography was performed over silica gel (40 – 45 μm, 300 – 400 mesh).

Analytical thin layer chromatography (TLC) was performed on silica gel $HSGF_{254}$ glass plates (purchased from Jiangyou silica gel development Co., Ltd, Yantai, China) containing a 254 nm fluorescent indicator. TLC plates were visualized by exposure to short wave ultraviolet light (254 nm) or I_2 and to a solution of $KMnO_4$ (1 g of $KMnO_4$, 6 g of K_2CO_3 and 0.1 g of KOH in 100 mL of H_2O) or vanillin (2 g of vanillin and 4 mL of concentrated H_2SO_4 in 100 mL of EtOH) followed by heating.

Organic solutions were concentrated at 30 – 40 °C on rotary evaporators at ~80 mbar followed by drying on vacuum pump below 1 mbar. Reaction temperatures are reported as the temperature of the bath surrounding the vessel unless otherwise stated.

Materials. Commercial reagents and solvents were obtained from Adamas-beta, Aldrich Chemical Co., Alfa Aesar, Macklin and Energy Chemical and used as received. All solvents were dried and/or distilled by standard methods.[1-3] The 1,3-enynes **1** were synthesized according to the reported literature procedures.[4-5]

Instrumentation.

- Proton nuclear magnetic resonance (^1H NMR) spectra were measured on a JEOL JNM-ECZ600R/S1 spectrometer at ambient temperature for ^1H at 600 MHz. Proton chemical shifts are reported in parts per million (δ scale), and are referenced using tetramethylsilane (TMS) as an internal standard or residual protium in the NMR solvent ($CDCl_3$: δ 7.26 ($CHCl_3$) or DMSO-d_6: δ 2.50 (CD_2HSOCD_3)). Data are reported as follows: chemical shift [multiplicity (s = singlet, d = doublet, t = triplet, q = quartet, m = multiplet, dd = doublet of doublets, td = triplet of doublets, brs = broad singlet), coupling constant(s) (Hz), integration].
- Carbon-13 nuclear magnetic resonance (^{13}C NMR) spectra measured on a JEOL JNM-ECZ600R/S1 spectrometer at ambient temperature for ^{13}C at 151 MHz.. Carbon chemical shifts are reported in parts per million (δ scale), and are referenced using the carbon resonances of the solvent (δ 77.00 ($CDCl_3$) or δ 39.52 (DMSO-d_6)). Data are reported as follows: chemical shift [multiplicity (if not singlet), assignment (C_q = fully substituted

carbon)].

- High resolution mass spectra (HRMS) were performed on an Agilent 6230 time-of-flight (TOF) LC/MS instrument or a Waters SYNAPT G2 mass spectrometer by using an electrospray ionization (ESI) ionization source analyzed by quadrupole time-of-flight (Q-TOF). Melting points were determined on a SGW X-4 digital melting point apparatus and temperatures were not corrected.

2. Further Optimization Studies

Table S1. Optimization of the 1,3,4-trifunctionalization of 1,3-enynes **1a**[a]

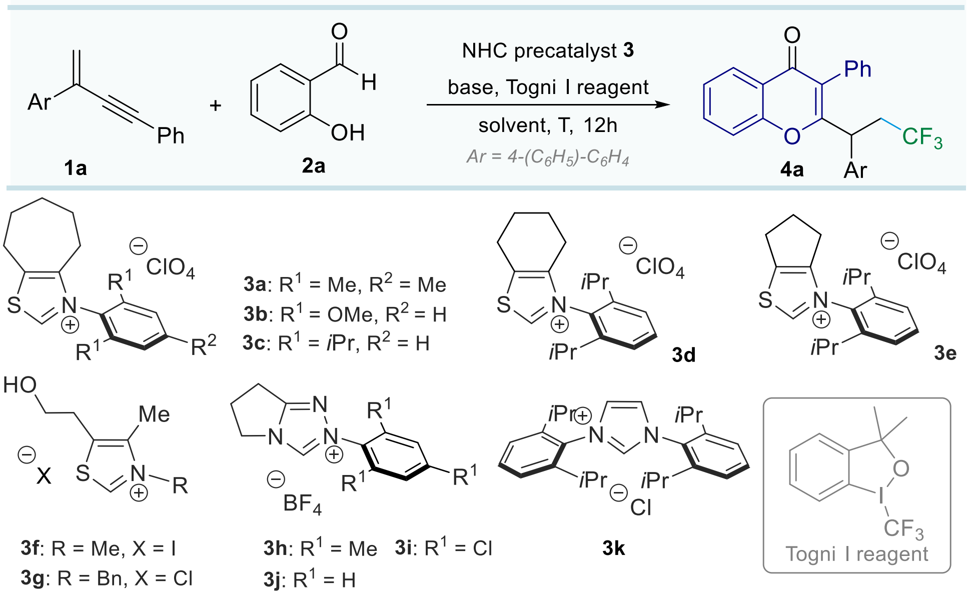

Entry	NHC **3**	Base	Solvent	Temp. (°C)	Yield (%)
1	**3a**	Cs_2CO_3	CF_3Ph	60	30
2	**3b**	Cs_2CO_3	CF_3Ph	60	68
3	**3c**	Cs_2CO_3	CF_3Ph	60	70
4	**3d**	Cs_2CO_3	CF_3Ph	60	69
5	**3e**	Cs_2CO_3	CF_3Ph	60	<5
6	**3f**	Cs_2CO_3	CF_3Ph	60	9
7	**3g**	Cs_2CO_3	CF_3Ph	60	11
8	**3h**	Cs_2CO_3	CF_3Ph	60	<5
9	**3i**	Cs_2CO_3	CF_3Ph	60	17
10	**3j**	Cs_2CO_3	CF_3Ph	60	<5
11	**3k**	Cs_2CO_3	CF_3Ph	60	<5
12	**3c**	K_2CO_3	CF_3Ph	60	68
13	**3c**	K_3PO_4	CF_3Ph	60	43
14	**3c**	Et_3N	CF_3Ph	60	49
15	**3c**	DBU	CF_3Ph	60	19
16	**3c**	DABCO	CF_3Ph	60	68
17	**3c**	Cs_2CO_3	DCM	60	64
18	**3c**	Cs_2CO_3	MeCN	60	57
19	**3c**	Cs_2CO_3	DMSO	60	<5
20	**3c**	Cs_2CO_3	Actone	60	47
21	**3c**	Cs_2CO_3	*i*Pr_2O	60	64
22	**3c**	Cs_2CO_3	CF_3Ph	40	63

The reactions were carried out with **1a** (0.15 mmol), aldehyde **2a** (0.10 mmol), NHC **3** (0.02 mmol), base (0.04 mmol) and Togni I reagent (0.15 mmol) in solvent (1.0 mL) for 12h.

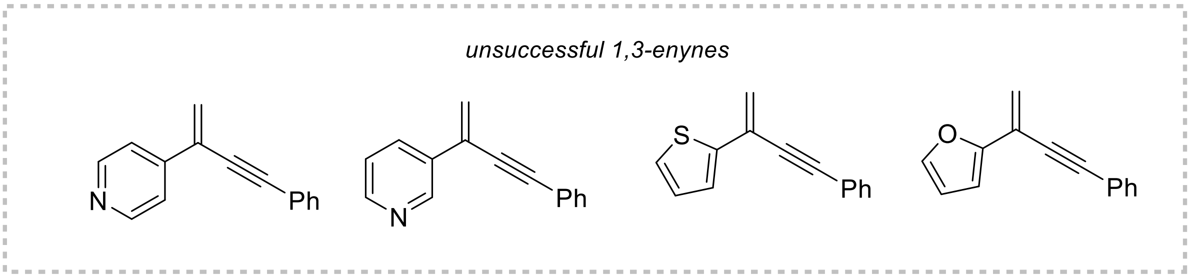

Schemem S1. unsuccessful substrates

Table S2. Optimization of the 1,3,4-trifunctionalization of 1,3-enynes bearing pyridyl[a]

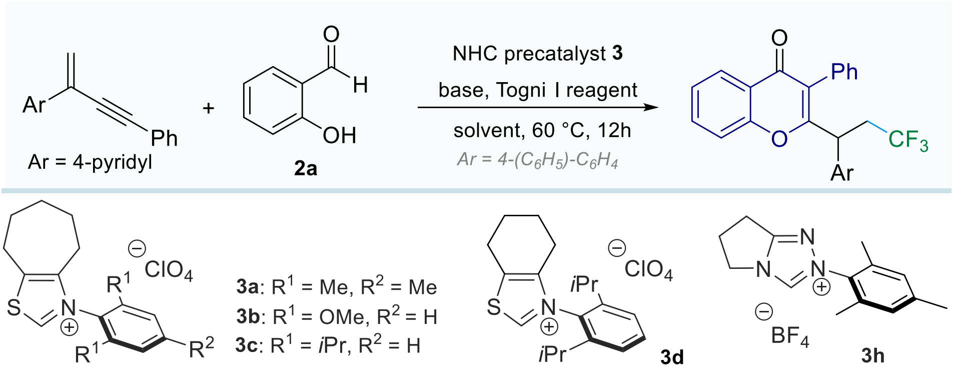

Entry	NHC **3**	Base	Solvent	Yield (%)
1	**3a**	Cs_2CO_3	CF_3Ph	<5
2	**3b**	Cs_2CO_3	CF_3Ph	<5
3	**3c**	Cs_2CO_3	CF_3Ph	<5
4	**3d**	Cs_2CO_3	CF_3Ph	<5
5	**3h**	Cs_2CO_3	CF_3Ph	<5
6	**3a**	Cs_2CO_3	DCM	<5
7	**3a**	Cs_2CO_3	Et_2O	<5
8	**3a**	Cs_2CO_3	MeCN	<5
9	**3a**	Cs_2CO_3	DMSO	<5
10	**3a**	Cs_2CO_3	Actone	<5
11	**3a**	Et_3N	CF_3Ph	<5
12	**3a**	DABCO	CF_3Ph	<5
13	**3a**	K_3PO_4	CF_3Ph	<5
14	**3a**	K_2CO_3	CF_3Ph	<5
15	**3a**	DBU	CF_3Ph	<5

3. General Procedure for the Synthesis of Isoflavone Fluorinated Derivatives

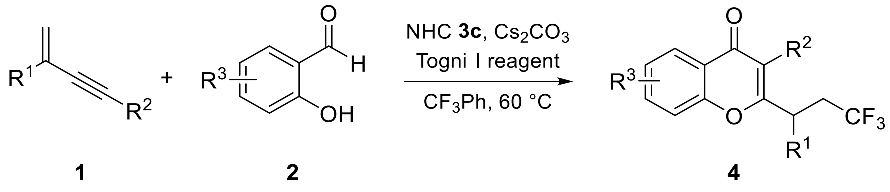

To a flame-dried Schlenk tube were added NHC **3c** (0.02 mmol), Togni I reagent (0.15 mmol) and Cs_2CO_3 (0.04 mmol), after which the tube was evacuated and back-filled with argon three times. Subsequently, under the protection of Ar, a solution of the corresponding aldehydes **2** (0.10 mmol) and 1,3-enynes **1** (0.15 mmol) in anhydrous CF_3Ph (1.0 mL) were added via syringe. The resulting suspension was heated to 60 °C for 12 h, after which the reaction mixture was concentrated under reduced pressure and the resulting crude material was purified by column chromatography on silica gel to afford the corresponding products **4**, which were dried under vacuum and further analyzed by ^{1}H NMR, ^{13}C NMR, HRMS, etc.

2-(1-([1,1'-biphenyl]-4-yl)-3,3,3-trifluoropropyl)-3-phenyl-4H-chromen-4-one 4a

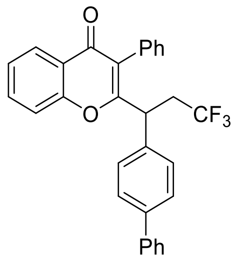

Prepared according to the general procedure to afford 32.9 mg of **4a** in 70% yield as creamy white solid; m.p. = 119.3 – 123.7 ºC.

NMR and HRMS data for the substrate ***4a***

^{1}H NMR (600 MHz, $CDCl_3$) δ (ppm): 8.23 (d, J = 7.8 Hz, 1H), 7.71 (t, J = 8.4 Hz, 1H), 7.58 – 7.52 (m, 6H), 7.46 – 7.40 (m, 6H), 7.34 (t, J = 7.8 Hz, 1H), 7.25 (d, J = 8.4 Hz, 2H), 7.12 – 7.07 (m, 1H), 4.38 (dd, J = 10.8, 4.2 Hz, 1H), 3.35 – 3.26 (m, 1H), 2.69 – 2.60 (m, 1H).

^{13}C NMR (151 MHz, $CDCl_3$) δ (ppm): 177.1, 162.7, 155.7, 141.0, 140.2, 137.3, 133.7, 132.1, 130.4, 128.8, 128.6, 128.3, 127.9, 127.7, 127.5, 127.0, 126.4, 125.8 (C-F, $^{1}J_{C-F}$ = 277.7 Hz), 125.3, 124.2, 123.5, 117.6, 41.2, 37.0 (C-F, $^{2}J_{C-F}$ = 29.0 Hz).

^{19}F NMR (564 MHz, $CDCl_3$) δ (ppm): -65.18 (t, J = 10.7 Hz, 3F).

HRMS (ESI-TOF) *m/z*: $[M + H]^+$ Calcd for $C_{30}H_{22}F_3O_2^+$ 471.1566; Found 471.1562.

2-(1-([1,1'-biphenyl]-4-yl)-3,3,3-trifluoropropyl)-6-methyl-3-phenyl-4H-chromen-4-one 4b

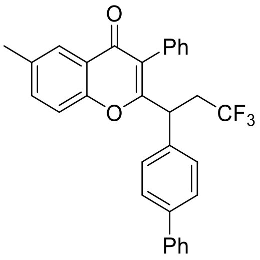

Prepared according to the general procedure to afford 37.8 mg of **4b** in 78% yield as light-yellow oil.

NMR and HRMS data for the substrate ***4b***

^{1}H NMR (600 MHz, CDCl$_3$) δ (ppm): 8.00 (s, 1H), 7.55 – 7.41 (m, 12H), 7.34 (t, J = 7.2 Hz, 1H), 7.24 (d, J = 8.4 Hz, 2H), 7.18 – 7.04 (m, 1H), 4.34 (dd, J = 10.2, 4.2 Hz, 1H), 3.34 – 3.25 (m, 1H), 2.67 – 2.59 (m, 1H), 2.45 (s, 3H).

^{13}C NMR (151 MHz, CDCl$_3$) δ (ppm): 177.1, 162.5, 154.0, 140.9, 140.2, 137.4, 135.2, 134.9, 132.3, 130.4, 128.8, 128.5, 128.3, 127.9, 127.7, 127.5, 127.0, 125.8 (C-F, $^{1}J_{C\text{-}F}$ = 279.0 Hz), 125.7, 124.0, 123.2, 117.3, 41.2, 37.0 (C-F, $^{2}J_{C\text{-}F}$ = 28.8 Hz), 20.9.

^{19}F NMR (564 MHz, CDCl$_3$) δ (ppm): -65.21 (t, J = 10.7 Hz, 3F).

HRMS (ESI-TOF) *m/z*: [M + H]$^+$ Calcd for $C_{31}H_{24}F_3O_2^+$ 485.1723; Found 485.1726.

2-(1-([1,1'-biphenyl]-4-yl)-3,3,3-trifluoropropyl)-7-methyl-3-phenyl-4H-chromen-4-one 4c

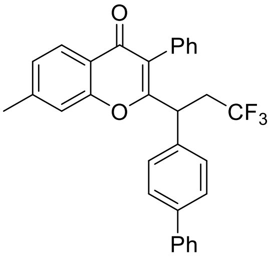

Prepared according to the general procedure to afford 43.1 mg of **4c** in 89% yield as light-yellow solid; m.p. = 79.1 – 83.5 ºC.

NMR and HRMS data for the substrate ***4c***

^{1}H NMR (600 MHz, CDCl$_3$) δ (ppm): 8.09 (d, J = 8.4 Hz, 1H), 7.54 – 7.39 (m, 10H), 7.37 (s, 1H), 7.31 (t, J = 7.8 Hz, 1H), 7.25 (d, J = 8.4 Hz, 2H), 7.19 (d, J = 8.4 Hz, 1H), 7.15 – 7.01 (m, 1H), 4.37 (dd, J = 10.2, 4.8 Hz, 1H), 3.33 – 3.24 (m, 1H), 2.66 – 2.58 (m, 1H), 2.48 (s, 3H).

^{13}C NMR (151 MHz, CDCl$_3$) δ (ppm): 177.0, 162.3, 155.8, 145.0, 140.9, 140.2, 137.5, 132.3, 130.7, 128.8, 128.6, 128.2, 127.9, 127.7, 127.5, 127.0, 126.8, 126.2, 125.8 (C-F, $^1J_{C-F}$ = 277.5 Hz), 124.1, 121.2, 117.4, 41.2, 37.0 (C-F, $^2J_{C-F}$ = 27.5 Hz), 21.8.

^{19}F NMR (564 MHz, CDCl$_3$) δ (ppm): -65.20 (t, J = 9.6 Hz, 3F).

HRMS (ESI-TOF) *m/z*: [M + H]$^+$ Calcd for $C_{31}H_{24}F_3O_2^+$ 485.1723; Found 485.1720.

2-(1-([1,1'-biphenyl]-4-yl)-3,3,3-trifluoropropyl)-7-methoxy-3-phenyl-4H-chromen-4-one 4d

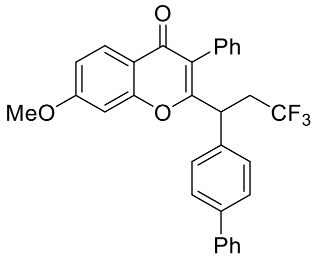

Prepared according to the general procedure to afford 31.0 mg of **4d** in 62% yield as yellow solid; m.p. = 201.3 – 203.5 ºC.

*NMR and HRMS data for the substrate **4d***

^{1}H NMR (600 MHz, CDCl$_3$) δ (ppm): 8.13 (d, J = 9.0 Hz, 1H), 7.55 – 7.41 (m, 9H), 7.34 (t, J = 7.2 Hz, 2H), 7.25 (d, J = 8.4 Hz, 2H), 7.19 – 7.03 (m, 1H), 6.97 – 6.95 (m, 2H), 4.35 (dd, J = 10.2, 4.2 Hz, 1H), 3.94 (s, 3H), 3.33 – 3.23 (m, 1H), 2.68 – 2.60 (m, 1H).

^{13}C NMR (151 MHz, CDCl$_3$) δ (ppm): 176.5, 164.2, 162.1, 157.4, 140.9, 140.2, 137.5, 132.2, 130.3, 128.8, 128.7, 128.2, 127.9, 127.7, 127.5, 127.0, 125.8 (C-F, $^1J_{C-F}$ = 277.5 Hz), 124.0, 117.3, 114.5, 99.9, 55.9, 41.1, 37.0 (C-F, $^2J_{C-F}$ = 27.5 Hz).

^{19}F NMR (564 MHz, CDCl$_3$) δ (ppm): -65.15 (t, J = 11.3 Hz, 3F).

HRMS (ESI-TOF) *m/z*: [M + H]$^+$ Calcd for $C_{31}H_{24}F_3O_3^+$ 501.1672; Found 501.1665.

2-(1-([1,1'-biphenyl]-4-yl)-3,3,3-trifluoropropyl)-7-bromo-3-phenyl-4H-chromen-4-one 4e

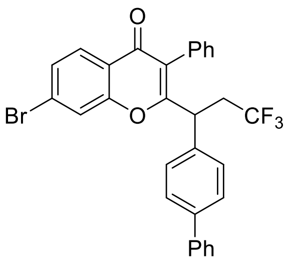

Prepared according to the general procedure to afford 40.1 mg of **4e** in 73% yield as light-yellow solid; m.p. = 101.1 – 104.3 °C.

*NMR and HRMS data for the substrate **4e***

^{1}H NMR (600 MHz, CDCl$_3$) δ (ppm): 8.07 (d, *J* = 9.0 Hz, 1H), 7.79 (s, 1H), 7.55 – 7.45 (m, 8H), 7.43 (t, *J* = 7.8 Hz, 2H), 7.34 (t, *J* = 7.2 Hz, 2H), 7.23 (d, *J* = 9.0 Hz, 2H), 7.17 – 7.07 (m, 1H), 4.36 (dd, *J* = 10.2, 4.2 Hz, 1H), 3.31 – 3.22 (m, 1H), 2.67 – 2.58 (m, 1H).

^{13}C NMR (151 MHz, CDCl$_3$) δ (ppm): 176.4, 162.8, 155.7, 141.1, 140.1, 137.0, 131.7, 130.3, 128.9, 128.8, 128.6, 128.5, 128.0, 127.9, 127.8, 127.6, 127.0, 125.7 (C-F, $^1J_{C-F}$ = 277.7 Hz), 124.5, 122.4, 120.8, 41.2, 36.9 (C-F, $^2J_{C-F}$ = 29.0 Hz).

^{19}F NMR (564 MHz, CDCl$_3$) δ (ppm): -65.20 (t, *J* = 10.7 Hz, 3F).

HRMS (ESI-TOF) *m/z*: [M + H]$^+$ Calcd for $C_{30}H_{21}{}^{79}BrF_3O_2^+$ 549.0672, $C_{30}H_{21}{}^{81}BrF_3O_2^+$ 551.0652; Found 549.0673, 551.0657.

2-(1-([1,1'-biphenyl]-4-yl)-3,3,3-trifluoropropyl)-7-chloro-3-phenyl-4H-chromen-4-one 4f

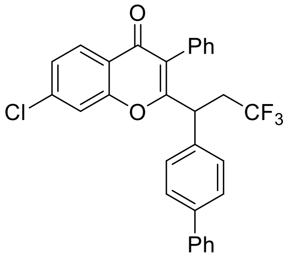

Prepared according to the general procedure to afford 37.4 mg of **4f** in 74% yield as light-yellow solid; m.p. = 87.7 – 89.9 °C.

*NMR and HRMS data for the substrate **4f***

^{1}H NMR (600 MHz, CDCl$_3$) δ (ppm): 8.14 (d, *J* = 7.8 Hz, 1H), 7.61 (s, 1H), 7.55 –7.45 (m, 7H), 7.42 (t, *J* = 7.8 Hz, 2H), 7.37 – 7.32 (m, 3H), 7.23 (d, *J* = 8.4 Hz, 2H), 7.17 – 7.02 (m, 1H), 4.36 (dd, *J* = 10.2, 4.2 Hz, 1H), 3.32 – 3.23 (m, 1H), 2.67 – 2.58 (m, 1H).

^{13}C NMR (151 MHz, CDCl$_3$) δ (ppm): 176.3, 162.9, 155.8, 141.1, 140.1, 139.8, 137.0, 131.7, 130.4, 128.8, 128.6, 128.5, 127.8, 127.8, 127.6, 127.0, 126.1, 125.7 (C-F, $^1J_{C-F}$ = 277.7 Hz), 124.5, 122.0, 117.7, 41.2, 36.9 (C-F, $^2J_{C-F}$ = 29.0 Hz).

^{19}F NMR (564 MHz, CDCl$_3$) δ (ppm): -65.21 (t, *J* = 10.7 Hz, 3F).

HRMS (ESI-TOF) *m/z*: [M + H]$^+$ Calcd for $C_{30}H_{21}{}^{35}ClF_3O_2^+$ 505.1177, $C_{30}H_{21}{}^{37}ClF_3O_2^+$ 507.1147; Found 505.1173, 507.1152.

2-(1-([1,1'-biphenyl]-4-yl)-3,3,3-trifluoropropyl)-7-fluoro-3-phenyl-4H-chromen-4-one 4g

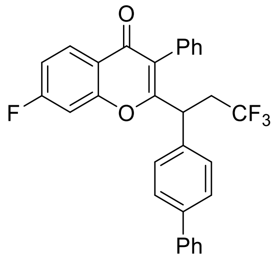

Prepared according to the general procedure to afford 32.2 mg of **4g** in 66% yield as light-yellow solid; m.p. = 75.1 – 78.2 ºC.

*NMR and HRMS data for the substrate **4g***

^{1}H NMR (600 MHz, $CDCl_3$) δ (ppm): 8.22 (t, *J* = 7.8 Hz, 1H), 7.54 – 7.45 (m, 7H), 7.42 (t, *J* = 7.2 Hz, 2H), 7.33 (t, *J* = 7.2 Hz, 2H), 7.28 – 7.23 (m, 3H), 7.17 – 7.10 (m, 2H), 4.36 (dd, *J* = 10.2, 4.2 Hz, 1H), 3.32 – 3.23 (m, 1H), 2.67 – 2.57 (m, 1H).

^{13}C NMR (151 MHz, $CDCl_3$) δ (ppm): 176.2, 165.7 (C-F, $^1J_{C-F}$ = 257.4 Hz), 162.9, 156.6 (C-F, $^3J_{C-F}$ = 13.0 Hz), 141.1, 140.1, 137.1, 131.7, 130.4, 129.0 (C-F, $^3J_{C-F}$ = 10.1 Hz), 128.8, 128.7, 128.5, 127.8, 127.8, 127.6, 127.0, 125.8 (C-F, $^1J_{C-F}$ = 277.5 Hz), 124.3, 120.3, 114.1 (C-F, $^2J_{C-F}$ = 23.1 Hz), 104.4 (C-F, $^2J_{C-F}$ = 24.5 Hz), 41.1, 36.9 ($C-F_3$, $^2J_{C-F}$ = 29.0 Hz).

^{19}F NMR (564 MHz, $CDCl_3$) δ (ppm): -65.21 (t, *J* = 9.9 Hz, 3F), -103.20 – -103.24 (m, 1F).

HRMS (ESI-TOF) *m/z*: $[M + Na]^+$ Calcd for $C_{30}H_{20}F_4O_2Na^+$ 511.1292; Found 511.1295.

2-(1-([1,1'-biphenyl]-4-yl)-3,3,3-trifluoropropyl)-8-methoxy-3-phenyl-4H-chromen-4-one 4h

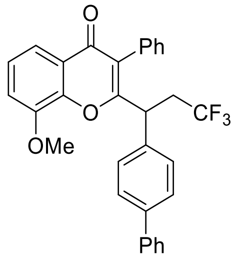

Prepared according to the general procedure to afford 39.0 mg of **4h** in 78% yield as light-yellow solid; m.p. = 170.7 – 171.8 ºC.

*NMR and HRMS data for the substrate **4h***

^{1}H NMR (600 MHz, $CDCl_3$) δ (ppm): 7.75 (d, *J* = 8.4 Hz, 1H), 7.56 – 7.41 (m, 10H), 7.35 – 7.29 (m, 4H), 7.19 (d, *J* = 7.8 Hz, 1H), 7.15 – 7.04 (m, 1H), 4.34 (dd, *J* = 10.2, 4.8 Hz, 1H), 4.08 (s, 3H), 3.42 – 3.32 (m, 1H), 2.68 – 2.60 (m, 1H).

^{13}C NMR (151 MHz, CDCl$_3$) δ (ppm): 177.1, 162.4, 149.0, 146.2, 140.8, 140.3, 137.4, 132.2, 130.4, 128.8, 128.6, 128.3, 128.2, 127.7, 127.5, 127.0, 125.9 (C-F, $^{1}J_{C-F}$ = 277.5 Hz), 124.7, 124.5, 123.9, 117.0, 114.2, 56.5, 41.5, 37.3 (C-F, $^{2}J_{C-F}$ = 28.8 Hz).

^{19}F NMR (564 MHz, CDCl$_3$) δ (ppm): -65.22 (t, J = 11.0 Hz, 3F).

HRMS (ESI-TOF) *m/z*: [M + H]$^+$ Calcd for $C_{31}H_{24}F_3O_3^+$ 501.1672; Found 501.1668.

2-(1-([1,1'-biphenyl]-4-yl)-3,3,3-trifluoropropyl)-8-fluoro-3-phenyl-4H-chromen-4-one 4i

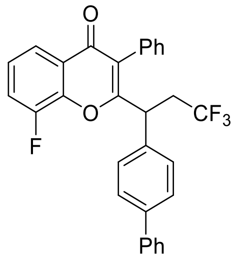

Prepared according to the general procedure afford 30.3 mg of **4i** in 62% yield as light-yellow solid; m.p. = 69.8 – 72.3 ºC.

*NMR and HRMS data for the substrate **4i***

^{1}H NMR (600 MHz, CDCl$_3$) δ (ppm): 7.96 (d, J = 8.4 Hz, 1H), 7.55 – 7.45 (m, 8H), 7.42 (t, J = 7.8 Hz, 2H), 7.37 – 7.31 (m, 3H), 7.29 (d, J = 8.4 Hz, 2H), 7.20 – 7.05 (m, 1H), 4.37 (dd, J = 10.2, 4.2 Hz, 1H), 3.40 – 3.31 (m, 1H), 2.68 – 2.60 (m, 1H).

^{13}C NMR (151 MHz, CDCl$_3$) δ (ppm): 176.2, 162.5, 151.1 (C-F, $^{1}J_{C-F}$ = 253.1 Hz), 144.5 (C-F, $^{2}J_{C-F}$ = 11.6 Hz), 141.1, 140.2, 136.9, 131.6, 130.4, 128.8, 128.7, 128.6, 128.0, 127.8, 127.6, 127.0, 125.8 (C-F, $^{1}J_{C-F}$ = 277.7 Hz), 125.3, 124.7 (C-F, $^{3}J_{C-F}$ = 5.7 Hz), 124.3, 121.4 (C-F, $^{3}J_{C-F}$ = 2.9 Hz), 119.3 (C-F, $^{2}J_{C-F}$ = 15.9 Hz), 41.4, 37.0 (C-F, $^{2}J_{C-F}$ = 29.0 Hz).

^{19}F NMR (564 MHz, CDCl$_3$) δ (ppm): -65.23 (t, J = 11.3 Hz, 3F), -135.07 – -135.10 (m, 1F).

HRMS (ESI-TOF) *m/z*: [M + Na]$^+$ Calcd for $C_{30}H_{20}F_4O_2Na^+$ 511.1292; Found 511.1297.

2-(1-([1,1'-biphenyl]-4-yl)-3,3,3-trifluoropropyl)-3-phenyl-4H-benzo[h]chromen-4-one 4j

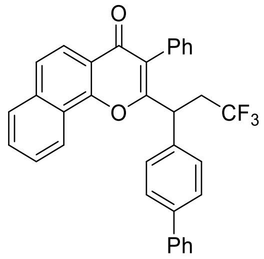

Prepared according to the general procedure to afford 21.3 mg of **4j** in 41% yield as white solid; m.p. = 203.8 – 205.0 ºC.

NMR and HRMS data for the substrate ***4j***

^{1}H NMR (600 MHz, $CDCl_3$) δ (ppm): 8.59 (d, J = 7.8 Hz, 1H), 8.17 (d, J = 9.0 Hz, 1H), 7.98 (d, J = 7.8 Hz, 1H), 7.79 – 7.75 (m, 3H), 7.59 – 7.47 (m, 7H), 7.41 (t, J = 7.8 Hz, 3H), 7.37 – 7.32 (m, 3H), 7.29 – 7.07 (m, 1H), 4.50 (dd, J = 10.2, 4.2 Hz, 1H), 3.49 – 3.39 (m, 1H), 2.85 – 2.77 (m, 1H).

^{13}C NMR (151 MHz, $CDCl_3$) δ (ppm): 176.9, 161.9, 153.0, 141.0, 140.1, 137.3, 136.0, 132.0, 130.2, 129.3, 128.8, 128.7, 128.5, 128.4, 127.9, 127.9, 127.6, 127.4, 127.0, 125.8 (C-F, $^1J_{C\text{-}F}$ = 279.0 Hz), 125.4, 125.4, 124.0, 121.7, 121.4, 119.9, 41.4, 37.5 (C-F, $^2J_{C\text{-}F}$ = 28.8 Hz).

^{19}F NMR (564 MHz, $CDCl_3$) δ (ppm): -65.02 (t, J = 10.7 Hz, 3F).

HRMS (ESI-TOF) *m/z*: $[M + H]^+$ Calcd for $C_{34}H_{24}F_3O_2^+$ 521.1723; Found 521.1729.

<u>3-phenyl-2-(3,3,3-trifluoro-1-phenylpropyl)-4H-chromen-4-one 4k</u>

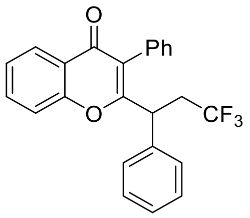

Prepared according to the general procedure to afford 31.6 mg of **4k** in 80% yield as light-yellow solid; m.p. = 140.3 – 143.1 °C.

NMR and HRMS data for the substrate ***4k***

^{1}H NMR (600 MHz, $CDCl_3$) δ (ppm): 8.21 (d, J = 7.2 Hz, 1H), 7.70 (t, J = 7.8 Hz, 1H), 7.56 (d, J = 8.4 Hz, 1H), 7.48 – 7.25 (m, 8H), 7.18 (d, J = 6.6 Hz, 2H), 7.14 – 6.95 (m, 1H), 4.32 (d, J = 10.2 Hz, 1H), 3.32 – 3.23 (m, 1H), 2.64 – 2.57 (m, 1H).

^{13}C NMR (151 MHz, $CDCl_3$) δ (ppm): 177.1, 162.7, 155.7, 138.4, 133.7, 132.1, 130.4, 129.1, 128.8, 128.3, 128.0, 127.4, 126.4, 125.8 (C-F, $^1J_{C\text{-}F}$ = 277.5 Hz), 125.2, 124.2, 123.0, 117.6, 41.5, 37.0 (C-F, $^2J_{C\text{-}F}$ = 27.5 Hz).

^{19}F NMR (564 MHz, $CDCl_3$) δ (ppm): -65.24 (t, J = 10.7 Hz, 3F).

HRMS (ESI-TOF) *m/z*: $[M + Na]^+$ Calcd for $C_{24}H_{17}F_3O_2Na^+$ 417.1073; Found 417.1080.

3-phenyl-2-(3,3,3-trifluoro-1-(4-methoxyphenyl)propyl)-4H-chromen-4-one 4l

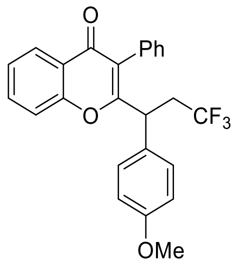

Prepared according to the general procedure to afford 31.4 mg of **4l** in 74% yield as light-yellow solid; m.p. = 99.8 – 103.3 °C.

NMR and HRMS data for the substrate ***4l***

^{1}H NMR (600 MHz, CDCl$_3$) δ (ppm): 8.21 (d, J = 8.4 Hz, 1H), 7.69 (t, J = 7.2 Hz, 1H), 7.55 (d, J = 7.8 Hz, 1H), 7.48 – 7.42 (m, 3H), 7.40 (t, J = 8.4 Hz, 1H), 7.35 – 7.28 (m, 1H), 7.11 – 7.04 (m, 3H), 6.83 (d, J = 9.0 Hz, 2H), 4.28 (dd, J = 10.2, 4.8 Hz, 1H), 3.77 (s, 3H), 3.28 – 3.18 (m, 1H), 2.62 – 2.53 (m, 1H).

^{13}C NMR (151 MHz, CDCl$_3$) δ (ppm): 177.1, 163.1, 159.2, 155.7, 133.6, 132.2, 130.5, 130.4, 128.5, 128.4, 128.3, 126.4, 125.8 (C-F, $^1J_{C\text{-}F}$ = 277.5 Hz), 125.2, 123.8, 123.5, 117.6, 114.4, 55.2, 40.7, 37.1 (C-F, $^2J_{C\text{-}F}$ = 27.5 Hz).

^{19}F NMR (564 MHz, CDCl$_3$) δ (ppm): -65.21 (t, J = 10.7 Hz, 3F).

HRMS (ESI-TOF) *m/z*: [M + Na]$^+$ Calcd for $C_{25}H_{19}F_3O_3Na^+$ 447.1179; Found 447.1185.

3-phenyl-2-(3,3,3-trifluoro-1-(4-fluorophenyl)propyl)-4H-chromen-4-one 4m

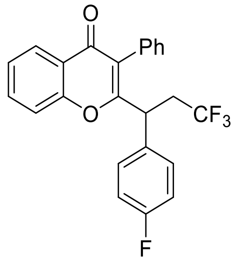

Prepared according to the general procedure to afford 23.9 mg of **4m** in 58% yield as light-yellow solid; m.p. = 113.4 – 117.1 °C.

NMR and HRMS data for the substrate ***4m***

^{1}H NMR (600 MHz, CDCl$_3$) δ (ppm): 8.22 (d, J = 8.4 Hz, 1H), 7.70 (t, J = 7.2 Hz, 1H), 7.55 (d, J = 7.8 Hz, 1H), 7.50 – 7.43 (m, 3H), 7.41 (t, J = 7.2 Hz, 1H), 7.34 – 7.28 (m, 1H), 7.16 – 7.12 (m, 2H), 6.99 (t, J = 8.4 Hz, 3H), 4.32 (dd, J = 9.6, 4.8 Hz, 1H), 3.27 – 3.18 (m, 1H), 2.64 – 2.55 (m, 1H).

^{13}C NMR (151 MHz, $CDCl_3$) δ (ppm): 177.0, 162.4, 162.3 (C-F, $^{1}J_{C\text{-}F}$ = 247.3 Hz), 155.6, 134.1 (C-F, $^{4}J_{C\text{-}F}$ = 2.9 Hz), 133.7, 132.0, 130.3, 129.1 (C-F, $^{3}J_{C\text{-}F}$ = 8.8 Hz), 128.9, 128.4, 126.4, 125.7 (C-F, $^{1}J_{C\text{-}F}$ = 277.5 Hz), 125.3, 124.1, 123.4, 117.5, 116.0 (C-F, $^{2}J_{C\text{-}F}$ = 21.7 Hz), 40.8, 37.0 (C-F, $^{2}J_{C\text{-}F}$ = 29.0 Hz).

^{19}F NMR (564 MHz, $CDCl_3$) δ (ppm): -64.53 (t, J = 10.7 Hz, 3F), -112.81 – -112.85 (m, 1F).

HRMS (ESI-TOF) *m/z*: $[M + Na]^+$ Calcd for $C_{24}H_{16}F_4O_2Na^+$ 435.0979; Found 435.0988.

4-(3,3,3-trifluoro-1-(4-oxo-3-phenyl-4H-chromen-2-yl)propyl)benzonitrile 4n

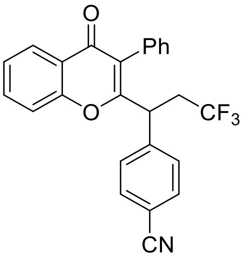

Prepared according to the general procedure to afford 28.9 mg of **4n** in 69% yield as light-yellow solid; m.p. = 193.1 – 194.2 ºC.

*NMR and HRMS data for the substrate **4n***

^{1}H NMR (600 MHz, $CDCl_3$) δ (ppm): 8.22 (d, J = 7.8 Hz, 1H), 7.73 (t, J = 7.2 Hz, 1H), 7.61 (d, J = 9.0 Hz, 2H), 7.55 (d, J = 8.4 Hz, 1H), 7.53 – 7.42 (m, 4H), 7.39 – 7.28 (m, 3H), 7.08 – 6.93 (m, 1H), 4.40 (dd, J = 9.6, 6.0 Hz, 1H), 3.28 – 3.19 (m, 1H), 2.70 – 2.61 (m, 1H).

^{13}C NMR (151 MHz, $CDCl_3$) δ (ppm): 176.9, 161.2, 155.7, 143.5, 134.1, 133.0, 131.8, 130.3, 129.2, 128.7, 128.5, 126.6, 125.7, 125.6 (C-F, $^{1}J_{C\text{-}F}$ = 287.7 Hz), 1124.8, 123.5, 118.2, 117.6, 112.3, 41.7, 36.7 (C-F, $^{2}J_{C\text{-}F}$ = 28.8 Hz).

^{19}F NMR (564 MHz, $CDCl_3$) δ (ppm): -65.07 (t, J = 10.7 Hz, 3F).

HRMS (ESI-TOF) *m/z*: $[M + Na]^+$ Calcd for $C_{25}H_{16}F_3NO_2Na^+$ 442.1025; Found 442.1028.

3-phenyl-2-(3,3,3-trifluoro-1-(3-methoxyphenyl)propyl)-4H-chromen-4-one 4o

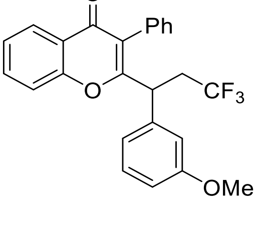

Prepared according to the general procedure to afford 29.7 mg of **4o** in 70% yield as light-yellow solid; m.p. = 124.4 – 125.7 °C.

*NMR and HRMS data for the substrate **4o***

^{1}H NMR (600 MHz, CDCl$_3$) δ (ppm): 8.22 (d, *J* = 7.8 Hz, 1H), 7.70 (t, *J* = 7.8 Hz, 1H), 7.56 (d, *J* = 9.0 Hz, 1H), 7.48 – 7.44 (m, 3H), 7.41 (t, *J* = 7.8 Hz, 1H), 7.35 – 7.26 (m, 1H), 7.22 (t, *J* = 7.8 Hz, 1H), 7.18 – 7.08 (m, 1H), 6.81 (d, *J* = 8.4 Hz, 1H), 6.77 (d, *J* = 7.2 Hz, 1H), 6.69 (s, 1H), 4.29 (dd, *J* = 10.2, 2.4 Hz, 1H), 3.75 (s, 3H), 3.31 – 3.22 (m, 1H), 2.64 – 2.56 (m, 1H).

^{13}C NMR (151 MHz, CDCl$_3$) δ (ppm): 177.1, 162.6, 159.9, 155.6, 139.9, 133.7, 132.1, 130.6, 130.1, 128.7, 128.3, 126.4, 125.8 (C-F, $^1J_{C\text{-}F}$ = 277.5 Hz), 125.2, 124.2, 123.5, 119.6, 117.6, 113.6, 113.1, 55.2, 41.5, 36.9 (C-F, $^2J_{C\text{-}F}$ = 28.8 Hz).

^{19}F NMR (564 MHz, CDCl$_3$) δ (ppm): -65.26 (t, *J* = 10.7 Hz, 3F).

HRMS (ESI-TOF) *m/z*: $[M + Na]^+$ Calcd for $C_{25}H_{19}F_3O_3Na^+$ 447.1179; Found 447.1182.

3-phenyl-2-(3,3,3-trifluoro-1-(3-(trifluoromethyl)phenyl)propyl)-4H-chromen-4-one 4p

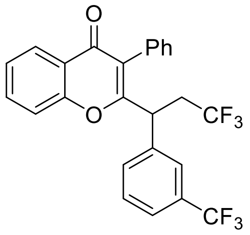

Prepared according to the general procedure to afford 29.1 mg of **4p** in 63% yield as light-yellow solid; m.p. = 114.6 – 118.8 °C.

*NMR and HRMS data for the substrate **4p***

^{1}H NMR (600 MHz, CDCl$_3$) δ (ppm): 8.22 (d, *J* = 7.8 Hz, 1H), 7.72 (t, *J* = 8.4 Hz, 1H), 7.57 – 7.51 (m, 3H), 7.48 – 7.41 (m, 5H), 7.37 (d, *J* = 8.4 Hz, 2H), 7.16 – 7.00 (m, 1H), 4.40 (dd, *J* = 9.6, 4.8 Hz, 1H), 3.33 – 3.24 (m, 1H), 2.69 – 2.61 (m, 1H).

^{13}C NMR (151 MHz, CDCl$_3$) δ (ppm): 176.9, 161.7, 155.6, 139.2, 133.9, 131.8, 131.4 (C-F, $^2J_{C\text{-}F}$ = 33.2 Hz), 130.8, 130.3, 129.6, 128.8, 128.6, 126.5, 125.6 (C-F, $^1J_{C\text{-}F}$ = 277.5 Hz), 125.4, 125.0 (C-F, $^3J_{C\text{-}F}$ = 4.4 Hz), 124.5, 124.5, 123.7 (C-F, $^1J_{C\text{-}F}$ = 273.3 Hz), 123.4, 117.5, 41.4, 36.7 (C-F, $^2J_{C\text{-}F}$ = 29.0 Hz).

^{19}F NMR (564 MHz, CDCl$_3$) δ (ppm): -63.23 (s, 3F), -65.15 (t, *J* = 9.9 Hz, 3F).

HRMS (ESI-TOF) *m/z*: $[M + Na]^+$ Calcd for $C_{25}H_{16}F_6O_2Na^+$ 485.0947; Found 485.0947.

2-(1-(3-chlorophenyl)-3,3,3-trifluoropropyl)-3-phenyl-4H-chromen-4-one 4q

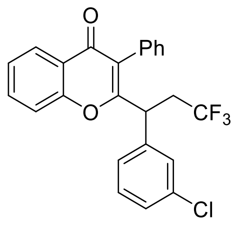

Prepared according to the general procedure to afford 32.2 mg of **4q** in 75% yield as light-yellow solid; m.p. = 125.9 – 129.1 ºC.

*NMR and HRMS data for the substrate **4q***

^{1}H NMR (600 MHz, CDCl$_3$) δ (ppm): 8.22 (d, J = 7.8 Hz, 1H), 7.72 (t, J = 8.4 Hz, 1H), 7.57 (d, J = 7.8 Hz, 1H), 7.50 – 7.44 (m, 3H), 7.42 (t, J = 7.8 Hz, 1H), 7.39 – 7.29 (m, 1H), 7.27 – 7.22 (m, 2H), 7.18 (s, 1H), 7.02 (d, J = 7.2 Hz, 2H), 4.30 (dd, J = 10.2, 4.8 Hz, 1H), 3.28 – 3.19 (m, 1H), 2.65 – 2.56 (m, 1H).

^{13}C NMR (151 MHz, CDCl$_3$) δ (ppm): 177.0, 161.9, 155.6, 140.2, 134.8, 133.8, 131.8, 130.4, 130.3, 128.7, 128.5, 128.3, 127.7, 126.4, 125.7, 125.6 (C-F, $^1J_{C\text{-}F}$ = 277.7 Hz), 125.4, 124.4, 123.4, 117.6, 41.2, 36.8 (C-F, $^2J_{C\text{-}F}$ = 29.0 Hz).

^{19}F NMR (564 MHz, CDCl$_3$) δ (ppm): -65.21 (t, J = 9.6 Hz, 3F).

HRMS (ESI-TOF) *m/z*: [M + Na]$^+$ Calcd for $C_{24}H_{16}{}^{35}ClF_3O_2Na^+$ 451.0684, $C_{24}H_{16}{}^{37}ClF_3O_2Na^+$ 453.0654; Found 451.0681, 453.0662.

3-phenyl-2-(3,3,3-trifluoro-1-(o-tolyl)propyl)-4H-chromen-4-one 4r

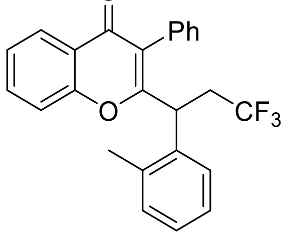

Prepared according to the general procedure to afford 14.7 mg of **4r** in 36% yield as light-yellow solid; m.p. = 127.6 – 131.9 ºC.

*NMR and HRMS data for the substrate **4r***

^{1}H NMR (600 MHz, CDCl$_3$) δ (ppm): 8.25 (d, *J* = 7.2 Hz, 1H), 7.74 (t, *J* = 8.4 Hz, 1H), 7.64 (d, *J* = 8.4 Hz, 1H), 7.44 (t, *J* = 7.2 Hz, 3H), 7.40 – 7.27 (m, 3H), 7.16 – 7.12 (m, 2H), 7.07 – 7.05 (m, 1H), 6.94 – 6.69 (m, 1H), 4.60 (dd, *J* = 10.8, 4.2 Hz, 1H), 3.29 – 3.19 (m, 1H), 2.46 – 2.38 (m, 1H), 1.64 (s, 3H).

^{13}C NMR (151 MHz, CDCl$_3$) δ (ppm): 177.1, 163.3, 155.6, 137.8, 135.2, 133.7, 132.2, 131.1, 130.1, 128.8, 128.2, 127.7, 126.8, 126.5, 126.4, 125.8 (C-F, $^{1}J_{C\text{-}F}$ = 277.7 Hz), 125.4, 124.4, 123.6, 117.6, 37.3, 37.0 (C-F, $^{2}J_{C\text{-}F}$ = 27.7 Hz), 18.3.

^{19}F NMR (564 MHz, CDCl$_3$) δ (ppm): -65.91 (t, *J* = 10.7 Hz, 3F).

HRMS (ESI-TOF) *m/z*: [M + Na]$^+$ Calcd for $C_{25}H_{19}F_3O_2Na^+$ 431.1230; Found 431.1234.

2-(1-(benzo[d][1,3]dioxol-5-yl)-3,3,3-trifluoropropyl)-3-phenyl-4H-chromen-4-one 4s

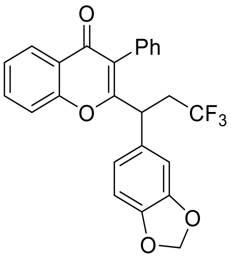

Prepared according to the general procedure to afford 18.4 mg of **4s** in 42% yield as light-yellow solid; m.p. = 166.6 – 169.1 °C.

NMR and HRMS data for the substrate ***4s***

^{1}H NMR (600 MHz, CDCl$_3$) δ (ppm): 8.21 (d, *J* = 7.8 Hz, 1H), 7.70 (t, *J* = 7.8, 1H), 7.55 (d, *J* = 8.4 Hz, 1H), 7.47 – 7.39 (m, 4H), 7.37 – 7.23 (m, 1H), 7.20 – 6.97 (m, 1H), 6.72 – 6.70 (m, 2H), 6.55 (d, *J* = 8.4 Hz, 1H), 5.93 (d, *J* = 10.8 Hz, 2H), 4.24 (dd, *J* = 10.2, 4.8 Hz, 1H), 3.24 – 3.15 (m, 1H), 2.61 – 2.52 (m, 1H).

^{13}C NMR (151 MHz, CDCl$_3$) δ (ppm): 177.1, 162.8, 155.6, 148.1, 147.3, 133.7, 132.0, 132.0, 130.3, 128.8, 128.3, 126.4, 125.7 (C-F, $^{1}J_{C\text{-}F}$ = 277.7 Hz), 125.3, 123.9, 123.4, 121.0, 117.6, 108.6, 107.6, 101.2, 41.1, 37.1 (C-F, $^{2}J_{C\text{-}F}$ = 29.0 Hz).

^{19}F NMR (564 MHz, CDCl$_3$) δ (ppm): -65.22 (t, *J* = 9.9 Hz, 3F).

HRMS (ESI-TOF) *m/z*: [M + Na]$^+$ Calcd for $C_{25}H_{17}F_3O_4Na^+$ 461.0971; Found 461.0977.

3-phenyl-2-(4,4,4-trifluorobutan-2-yl)-4H-chromen-4-one 4t

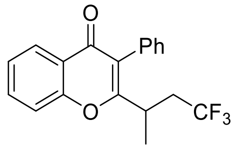

Prepared according to the general procedureto afford 15.3 mg of **4t** in 46% yield as light-yellow solid; m.p. = 104.9 – 107.6 °C.

*NMR and HRMS data for the substrate **4t***

^{1}H NMR (600 MHz, CDCl$_3$) δ (ppm): 8.24 (d, J = 7.8 Hz, 1H), 7.69 (t, J = 7.2 Hz, 1H), 7.49 – 7.44 (m, 3H), 7.43 – 7.39 (m, 2H), 7.25 (d, J = 7.2 Hz, 2H), 3.25 – 3.19 (m, 1H), 2.80 – 2.71 (m, 1H), 2.31 – 2.22 (m, 1H), 1.35 (d, J = 7.2 Hz, 3H).

^{13}C NMR (151 MHz, CDCl$_3$) δ (ppm): 177.1, 165.6, 155.8, 133.6, 132.3, 130.1, 128.6, 128.1, 126.4, 126.0 (C-F, $^{1}J_{C\text{-}F}$ = 276.2 Hz), 125.1, 123.4, 123.3, 117.5, 37.3 (C-F, $^{2}J_{C\text{-}F}$ = 28.8 Hz), 30.9, 19.4.

^{19}F NMR (564 MHz, CDCl$_3$) δ (ppm): -65.17 (t, J = 10.7 Hz, 3F).

HRMS (ESI-TOF) *m/z*: $[M + Na]^+$ Calcd for $C_{19}H_{15}F_3O_2Na^+$ 355.0917; Found 355.0925.

4-(3,3,3-trifluoro-1-(6-methoxy-4-oxo-3-phenyl-4H-chromen-2-yl)propyl)benzonitrile 4u

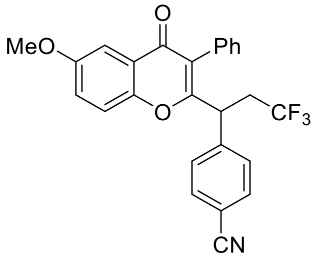

Prepared according to the general procedure to afford 21.6 mg of **4u** in 48% yield as light-yellow solid; m.p. = 184.7 – 187.2 °C.

*NMR and HRMS data for the substrate **4u***

^{1}H NMR (600 MHz, CDCl$_3$) δ (ppm): 7.60 (d, J = 8.4 Hz, 2H), 7.57 (d, J = 3.0 Hz, 1H), 7.52 – 7.39 (m, 4H), 7.36 – 7.30 (m, 2H), 7.26 (d, J = 9.0 Hz, 2H), 7.12 – 6.91 (m, 1H), 4.39 (dd, J = 9.6, 5.4 Hz, 1H), 3.88 (s, 3H), 3.26 – 3.17 (m, 1H), 2.68 – 2.60 (m, 1H).

^{13}C NMR (151 MHz, CDCl$_3$) δ (ppm): 176.6, 160.9, 157.2, 150.4, 143.5, 132.8, 131.9, 130.3, 129.0, 128.6, 128.3, 125.5 (C-F, $^{1}J_{C\text{-}F}$ = 277.7 Hz), 124.0, 123.9, 123.9, 118.9, 118.1, 112.2, 105.6, 56.0, 41.6, 36.7 (C-F, $^{2}J_{C\text{-}F}$ = 28.8 Hz).

^{19}F NMR (564 MHz, CDCl$_3$) δ (ppm): -65.10 (t, J = 11.0 Hz, 3F).

HRMS (ESI-TOF) *m/z*: $[M + Na]^+$ Calcd for $C_{26}H_{18}F_3NO_3Na^+$ 472.1131; Found 472.1141.

6-methoxy-3-phenyl-2-(3,3,3-trifluoro-1-(3-(trifluoromethyl)phenyl)propyl)-4H-chromen-4-one 4v

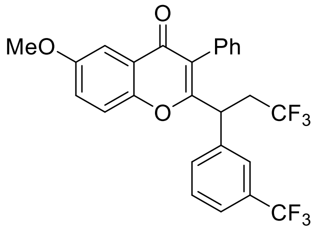

Prepared according to the general procedure to afford 32.0 mg of **4v** in 65% yield as light-yellow solid; m.p. = 117.2 – 120.9 ºC.

NMR and HRMS data for the substrate ***4v***

^{1}H NMR (600 MHz, CDCl$_3$) δ (ppm): 7.57 (d, J = 3.6 Hz, 1H), 7.55 – 7.42 (m, 6H), 7.39 (s, 1H), 7.36 – 7.30 (m, 3H), 7.16 – 6.89 (m, 1H), 4.38 (dd, J = 9.6, 4.8 Hz, 1H), 3.88 (s, 3H), 3.31 – 3.22 (m, 1H), 2.68 – 2.59 (m, 1H).

^{13}C NMR (151 MHz, CDCl$_3$) δ (ppm): 176.8, 161.5, 157.2, 150.5, 139.4, 132.0, 131.4 (C-F, $^2J_{C\text{-}F}$ = 33.4 Hz), 130.8, 130.4, 129.6, 129.0, 128.5, 125.6 (C-F, $^1J_{C\text{-}F}$ = 277.7 Hz), 124.9 (C-F, $^3J_{C\text{-}F}$ = 2.9 Hz), 124.5 (C-F, $^3J_{C\text{-}F}$ = 2.9 Hz), 124.1, 123.9, 123.7, 123.7 (C-F, $^1J_{C\text{-}F}$ = 271.8 Hz), 118.9, 105.6, 56.0, 41.4, 36.7 (C-F, $^2J_{C\text{-}F}$ = 29.0 Hz).

^{19}F NMR (564 MHz, CDCl$_3$) δ (ppm): -63.23 (s, 3F), -65.17 (t, J = 11.0 Hz, 3F).

HRMS (ESI-TOF) *m/z*: $[M + Na]^+$ Calcd for $C_{26}H_{18}F_6O_3Na^+$ 515.1053; Found 515.1062.

6-methoxy-3-phenyl-2-(3,3,3-trifluoro-1-(3-methoxyphenyl)propyl)-4H-chromen-4-one 4w

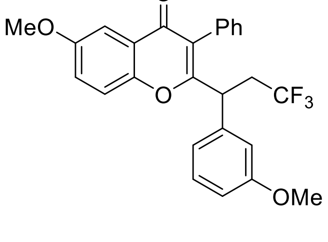

Prepared according to the general procedure to afford 29.5 mg of **4w** in 65% yield as light-yellow solid; m.p. = 61.5 – 65.4 ºC.

*NMR and HRMS data for the substrate **4w***

^{1}H NMR (600 MHz, CDCl$_3$) δ (ppm): 7.57 (s, 1H), 7.49 – 7.27 (m, 6H), 7.21 (t, J = 7.8 Hz, 1H), 7.18 – 7.00 (m, 1H), 6.80 (d, J = 8.4 Hz, 1H), 6.76 (d, J = 7.8 Hz, 1H), 6.67 (s, 1H), 4.28 (dd, J = 10.2, 3.6 Hz, 1H), 3.86 (s, 3H), 3.74 (s, 3H), 3.29 – 3.20 (m, 1H), 2.63 – 2.54 (m, 1H).

^{13}C NMR (151 MHz, CDCl$_3$) δ (ppm): 176.9, 162.4, 159.8, 157.0, 150.5, 140.0, 132.3, 130.8, 130.0, 128.8, 128.2, 125.8 (C-F, $^1J_{C\text{-}F}$ = 277.7 Hz), 124.0, 123.7, 123.4, 119.6, 119.0, 113.6, 113.0, 105.5, 55.9, 55.2, 41.4, 36.9 (C-F, $^2J_{C\text{-}F}$ = 28.4 Hz).

^{19}F NMR (564 MHz, CDCl$_3$) δ (ppm): -65.28 (t, J = 9.9 Hz, 3F).

HRMS (ESI-TOF) *m/z*: [M + Na]$^+$ Calcd for $C_{26}H_{21}F_3O_4Na^+$ 477.1284; Found 477.1279.

2-(1-(3,4-dimethoxyphenyl)-3,3,3-trifluoropropyl)-6-methoxy-3-phenyl-4H-chromen-4-one 4x

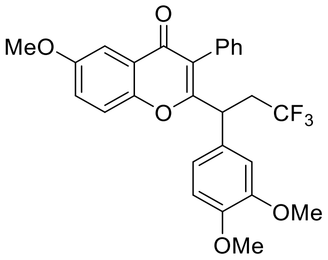

Prepared according to the general procedure to afford 26.6 mg of **4x** in 55% yield as light-yellow solid; m.p. = 135.1 – 137.6 ºC.

*NMR and HRMS data for the substrate **4x***

^{1}H NMR (600 MHz, CDCl$_3$) δ (ppm): 7.57 (d, J = 2.4 Hz, 1H), 7.52 – 7.28 (m, 6H), 7.21 – 7.03 (m, 1H), 6.80 – 6.76 (m, 2H), 6.54 (s, 1H), 4.26 (dd, J = 10.8, 4.8 Hz, 1H), 3.88 (s, 3H), 3.84 (s, 3H), 3.79 (s, 3H), 3.30 – 3.20 (m, 1H), 2.62 – 2.54 (m, 1H).

^{13}C NMR (151 MHz, CDCl$_3$) δ (ppm): 176.9, 162.8, 157.0, 150.5, 149.0, 148.7, 132.5, 130.9, 130.2, 128.9, 128.3, 125.8 (C-F, $^1J_{C\text{-}F}$ = 277.5 Hz), 124.1, 123.7, 123.0, 119.3, 118.9, 111.4, 110.9, 105.5, 55.9, 55.8, 55.8, 41.0, 36.9 (C-F, $^2J_{C\text{-}F}$ = 28.8 Hz).

^{19}F NMR (564 MHz, CDCl$_3$) δ (ppm): -65.22 (t, J = 9.9 Hz, 3F).

HRMS (ESI-TOF) *m/z*: [M + Na]$^+$ Calcd for $C_{27}H_{23}F_3O_5Na^+$ 507.1390; Found 507.1400.

4-(3,3,3-trifluoro-1-(7-methoxy-4-oxo-3-phenyl-4H-chromen-2-yl)propyl)benzonitrile 4y

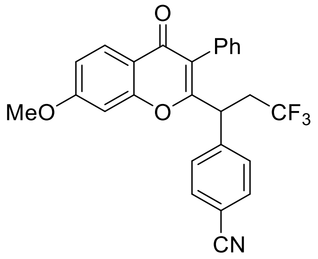

Prepared according to the general procedure to afford 20.2 mg of **4y** in 45% yield as light-yellow solid; m.p. = 175.7 – 178.9 °C.

NMR and HRMS data for the substrate ***4y***

^{1}H NMR (600 MHz, CDCl$_3$) δ (ppm): 8.11 (d, J = 9.0 Hz, 1H), 7.61 (d, J = 8.4 Hz, 2H), 7.54 – 7.24 (m, 6H), 7.04 – 6.98 (m, 2H), 6.93 (s, 1H), 4.37 (dd, J = 9.6, 4.8 Hz, 1H), 3.96 (s, 3H), 3.26 – 3.17 (m, 1H), 2.68 – 2.60 (m, 1H).

^{13}C NMR (151 MHz, CDCl$_3$) δ (ppm): 176.2, 164.4, 160.6, 157.3, 143.5, 132.9, 131.8, 130.2, 129.0, 128.5, 128.3, 127.9, 125.5 (C-F, $^1J_{C\text{-}F}$ = 277.5 Hz), 124.6, 118.2, 117.2, 114.7, 112.2, 99.9, 55.9, 41.5, 36.6 (C-F, $^2J_{C\text{-}F}$ = 30.4 Hz).

^{19}F NMR (564 MHz, CDCl$_3$) δ (ppm): -65.05 (t, J = 11.0 Hz, 3F).

HRMS (ESI-TOF) *m/z*: $[M + Na]^+$ Calcd for $C_{26}H_{18}F_3NO_3Na^+$ 472.1131; Found 472.1129.

7-methoxy-3-phenyl-2-(3,3,3-trifluoro-1-(3-methoxyphenyl)propyl)-4H-chromen-4-one 4z

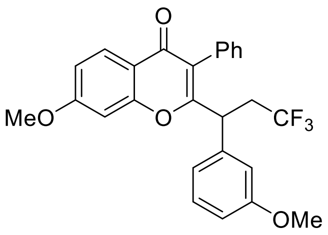

Prepared according to the general procedure to afford 24.5 mg of **4z** in 54% yield as light-yellow solid; m.p. = 117.9 – 120.3 °C.

NMR and HRMS data for the substrate ***4z***

^{1}H NMR (600 MHz, CDCl$_3$) δ (ppm): 8.11 (d, J = 9.0 Hz, 1H), 7.54 – 7.33 (m, 4H), 7.23 (t, J = 7.8 Hz, 1H), 7.14 – 7.02 (m, 1H), 6.97 (d, J = 9.0 Hz, 1H), 6.93 (s, 1H), 6.81 (d, J = 8.4, 3.0 Hz, 1H), 6.77 (d, J = 7.8 Hz, 1H), 6.68 (s, 1H), 4.27 (dd, J = 10.2, 4.2 Hz, 1H), 3.95 (s, 3H), 3.76 (s, 3H), 3.29 – 3.19 (m, 1H), 2.63 – 2.54 (m, 1H).

^{13}C NMR (151 MHz, CDCl$_3$) δ (ppm): 176.5, 164.2, 162.1, 159.8, 157.3, 140.0, 132.2, 130.7, 130.0, 128.7, 128.2, 127.8, 125.8 (C-F, $^1J_{C\text{-}F}$ = 277.7 Hz), 124.0, 119.6, 117.3, 114.5, 113.7, 112.9, 99.9, 55.8, 55.2, 41.4, 36.8 (C-F, $^2J_{C\text{-}F}$ = 28.4 Hz).

^{19}F NMR (564 MHz, CDCl$_3$) δ (ppm): -65.22 (t, J = 9.9 Hz, 3F).

HRMS (ESI-TOF) *m/z*: [M + Na]$^+$ Calcd for $C_{26}H_{21}F_3O_4Na^+$ 477.1284; Found 477.1289.

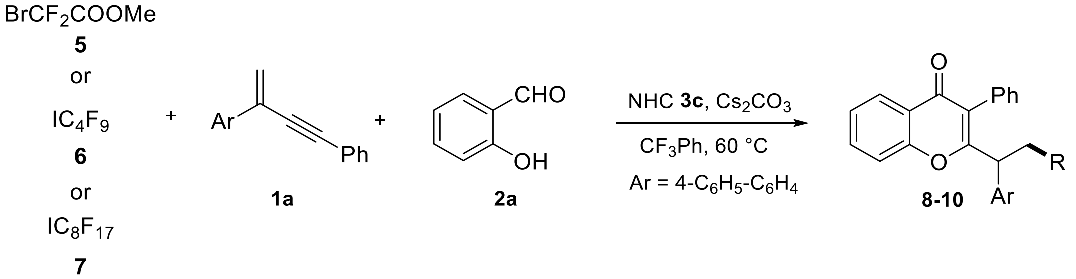

To a flame-dried Schlenk tube were added NHC **3c** (0.02 mmol), 1,3-enynes **1a** (0.15 mmol) and Cs_2CO_3 (0.15 mmol), after which the tube was evacuated and back-filled with argon three times. Subsequently, under the protection of Ar, a solution of the corresponding aldehydes **2a** (0.10 mmol) and radical precursor (0.15 mmol) in anhydrous CF_3Ph (1.0 mL) were added via syringe. The resulting suspension was heated to 60 °C for 12 h, after which the reaction mixture was concentrated under reduced pressure and the resulting crude material was purified by column chromatography on silica gel to afford the corresponding products, which were dried under vacuum and further analyzed by ^{1}H NMR, ^{13}C NMR, HRMS.

methyl 4-([1,1'-biphenyl]-4-yl)-2,2-difluoro-4-(4-oxo-3-phenyl-4H-chromen-2-yl)butanoate 8

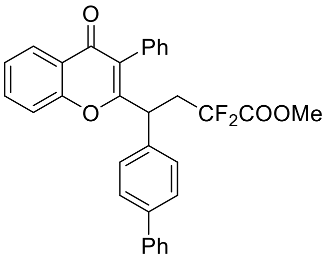

Prepared according to the general procedure to afford 29.1 mg of **8** in 57% yield as light-yellow solid; m.p. = 71.8 – 74.6 ºC.

*NMR and HRMS data for the substrate **8***

^{1}H NMR (600 MHz, CDCl$_3$) δ (ppm): 8.21 (d, *J* = 8.4 Hz, 1H), 7.68 (t, *J* = 7.2 Hz, 1H), 7.57 (d, *J* = 8.4 Hz, 1H), 7.54 – 7.37 (m, 11H), 7.32 (t, *J* = 7.2 Hz, 1H), 7.26 (d, *J* = 8.4 Hz, 2H), 7.18 – 7.02 (m, 1H), 4.38 (dd, *J* = 8.4, 6.0 Hz, 1H), 3.60 (s, 3H), 3.28 – 3.19 (m, 1H), 2.79 – 2.70 (m, 1H).

^{13}C NMR (151 MHz, CDCl$_3$) δ (ppm): 177.1, 163.9 (C-F, $^2J_{C-F}$ = 33.2 Hz), 163.3, 155.7, 140.8, 140.3, 137.6, 133.6, 132.2, 130.5, 128.8, 128.6, 128.3, 128.1, 127.6, 127.5, 127.0, 126.4, 125.2, 123.9, 123.5, 117.6, 115.0 (C-F, $^1J_{C-F}$ = 252.9 Hz), 53.3, 41.0, 37.7 (C-F, $^2J_{C-F}$ = 23.1 Hz).

^{19}F NMR (564 MHz, CDCl$_3$) δ (ppm): -104.06 (dq, *J* = 264.5, 10.2 Hz, 1F), -105.67 (dt, *J* = 264.0, 16.1 Hz, 1F).

HRMS (ESI-TOF) *m/z*: [M + H]$^+$ Calcd for $C_{32}H_{25}F_2O_4^+$ 511.1715; Found 511.1721.

2-(1-([1,1'-biphenyl]-4-yl)-3,3,4,4,5,5,6,6,6-nonafluorohexyl)-3-phenyl-4H-chromen-4-one 9

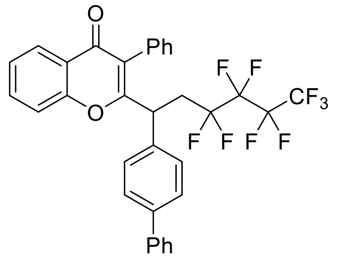

Prepared according to the general procedure to afford 45.3 mg of **9** in 73% yield as light-yellow solid; m.p. = 66.9 – 70.6 °C.

*NMR and HRMS data for the substrate **9***

^{1}H NMR (600 MHz, CDCl$_3$) δ (ppm): 8.23 (d, *J* = 8.4 Hz, 1H), 7.72 (t, *J* = 7.8 Hz, 1H), 7.59 (d, *J* = 8.4 Hz, 1H), 7.55 – 7.33 (m, 12H), 7.26 (d, *J* = 8.4 Hz, 2H), 7.18 – 7.00 (m, 1H), 4.49 (dd, *J* = 10.2, 4.2 Hz, 1H), 3.37 – 3.27 (m, 1H), 2.64 – 2.55 (m, 1H).

^{13}C NMR (151 MHz, CDCl$_3$) δ (ppm): 177.1, 162.8, 155.7, 141.0, 140.2, 137.7, 133.7, 132.1, 130.4, 128.8, 128.6, 128.3, 127.9, 127.8, 127.6, 127.0, 126.5, 125.3, 123.9, 123.6, 117.5, 121.8 – 106.9 (m, 4C), 40.0, 33.8 (C-F, $^2J_{C-F}$ = 21.0 Hz).

^{19}F NMR (564 MHz, CDCl$_3$) δ (ppm): -81.43 (t, *J* = 9.9 Hz, 3F), -113.86 – -114.90 (m, 2F), -124.77 – -125.25 (m, 2F), -126.12 – -126.78 (m, 2F).

HRMS (ESI-TOF) *m/z*: [M + H]$^+$ Calcd for $C_{33}H_{22}F_9O_2^+$ 621.1471; Found 621.1478.

2-(1-([1,1'-biphenyl]-4-yl)-3,3,4,4,5,5,6,6,7,7,8,8,9,9,10,10,10-heptadecafluorodecyl)-3-phenyl-4H-chromen-4-one 10

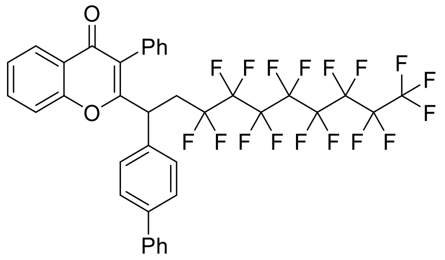

Prepared according to the general procedure to afford 62.4 mg of **10** in 76% yield as light-yellow solid; m.p. = 74.5 – 76.4 °C.

*NMR and HRMS data for the substrate **10***

^{1}H NMR (600 MHz, CDCl$_3$) δ (ppm): 8.24 (d, J = 8.4 Hz, 1H), 7.72 (t, J = 9.0 Hz, 1H), 7.59 (d, J = 8.4 Hz, 1H), 7.55 – 7.53 (m, 4H), 7.50 – 7.40 (m, 6H), 7.34 (t, J = 7.2 Hz, 2H), 7.26 (d, J = 8.4 Hz, 2H), 7.13 – 7.07 (m, 1H), 4.49 (dd, J = 10.2, 4.2 Hz, 1H), 3.38 – 3.27 (m, 1H), 2.65 – 2.55 (m, 1H).

^{13}C NMR (151 MHz, CDCl$_3$) δ (ppm): 177.1, 162.9, 155.7, 141.0, 140.2, 137.7, 133.7, 132.1, 130.4, 128.8, 128.5, 128.4, 127.9, 127.8, 127.6, 127.0, 126.5, 125.3, 123.9, 123.5, 118.0 – 108.4 (m, 8C), 117.5, 40.0, 33.6 (C-F, $^2J_{C\text{-}F}$ = 21.6 Hz).

^{19}F NMR (564 MHz, CDCl$_3$) δ (ppm): -81.18 (t, J = 10.7 Hz, 3F), -114.08 (s, 2F), -121.90 (s, 2F), -122.32 (s, 4F), -123.12 (s, 2F), -123.89 (s, 2F), -126.53 (s, 2F).

HRMS (ESI-TOF) *m/z*: [M + H]$^+$ Calcd for $C_{37}H_{22}F_{17}O_2^+$ 821.1343; Found 821.1345.

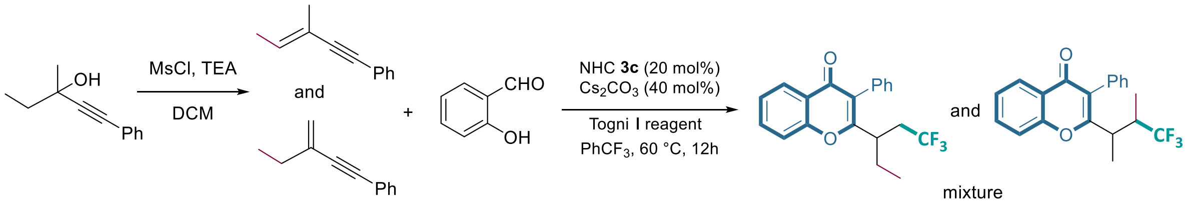

To a flame-dried Schlenk tube were added NHC **3c** (0.02 mmol), a mixture 1,3-enynes **1** (0.15 mmol) (difficult to purify the substrate) and Cs_2CO_3 (0.15 mmol), after which the tube was evacuated and back-filled with argon three times. Subsequently, under the protection of Ar, a solution of the corresponding aldehydes **2a** (0.10 mmol) and radical precursor (0.15 mmol) in anhydrous CF_3Ph (1.0 mL) were added via syringe. The resulting suspension was heated to

60 °C for 12 h, after which the reaction mixture was concentrated under reduced pressure and the resulting crude material was purified by column chromatography on silica gel to afford the corresponding mixture products, which were was detected by HRMS.

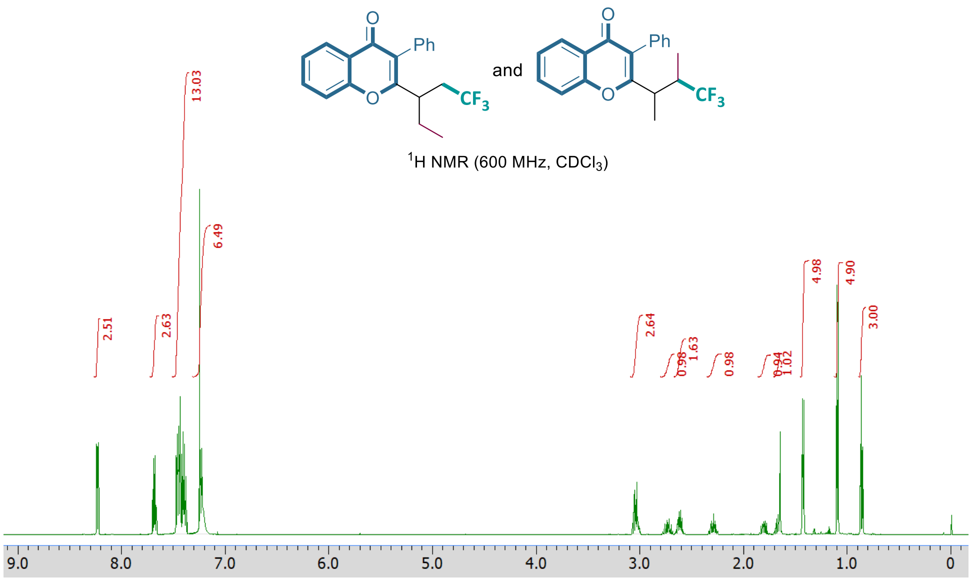

^{1}H NMR spectrum of the mixture with the R1 group of 1,3-enyne as ethyl

4. Crystal Data and Structure Refinement for 4n

To a tube containing **4n** (18 mg) was added a 20:1 mixture of petroleum ether and ethyl acetate (about 3 mL). The tube was kept aside for 5 days at room temperature to obtain crystals. The crystals were subjected for single crystal XRD to determine the structure of **4n**. The data were collected by a Bruker APEX-II CCD equipped with a Mo radiation source (Kα = 0.71073 Å) at 302.0 K. CCDC 2108112 (**4n**) contains the supplementary crystallographic data for this paper.

Crystal Data (at 50% probability level)

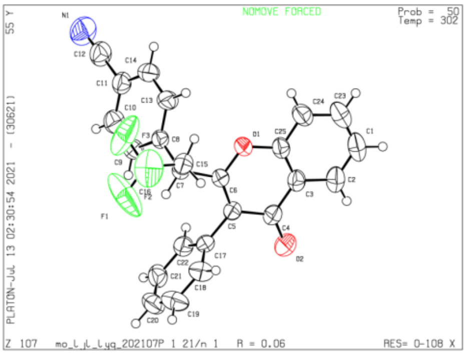

≡

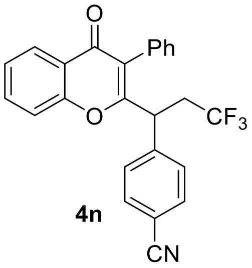

Identification code	**4n**
Empirical formula	$C_{25}H_{16}F_3NO_2$
Formula weight	419.39
Temperature/K	302.0
Crystal system	monoclinic
Space group	$P2_1/n$
a/Å	9.3387(6)
b/Å	17.5279(11)
c/Å	12.6351(8)
α /°	90
β /°	98.041(2)
γ /°	90
Volume/Å^3	2047.9(2)
Z	4
ρ_{calc}g/cm^3	1.360
μ /mm^{-1}	0.105
F(000)	864.0
Crystal size/mm^3	0.36 × 0.35 × 0.19
Radiation	MoKα (λ = 0.71073)
2Θ range for data collection/°	4 to 55.068

Index ranges	$-12 \leqslant h \leqslant 12$, $-22 \leqslant k \leqslant 22$, $-16 \leqslant l \leqslant 16$
Reflections collected	65268
Independent reflections	4711 [R_{int} = 0.0773, R_{sigma} = 0.0285]
Data/restraints/parameters	4711/0/280
Goodness-of-fit on F^2	1.027
Final R indexes [I>=2σ (I)]	R_1 = 0.0557, wR_2 = 0.1386
Final R indexes [all data]	R_1 = 0.0821, wR_2 = 0.1572
Largest diff. peak/hole / e $Å^{-3}$	0.44/-0.49

5. Biological Procedures

5.1 Experimental procedures

Cell culture and reagents. The MDA-MB-231 cells were purchased from American Type Culture Collection (ATCC, Manassas, VA, USA) and were maintained in DMEM containing 10% fetal bovine serum (FBS) at 37 °C. MTT (M2128), 3-MA (M9281), CQ (C6628) and DAPI (D9542) were purchased from Sigma-Aldrich (St. Louis, MO, USA). Bafilomycin A1 (ab120497) was purchased from Abcam (Cambridge, UK). Antibodies used in this study were as follow: Beclin1 (3495, CST), MMP-2 (87809, CST), E-cadherin (14472, CST), β-actin (66009-1-Ig, Proteintech, IL, USA), LAMP1(25630, Abcam).

Cell viability assay. The MDA-MB-231 cells (6×10^3 cells/well) were plated in 96-well and incubated at 37 °C for 24 h. Then, cells were treated with indicated experimental conditions for 24 h. Cell viability was measured by MTT assay.

Edu cell-proliferation assay. The MDA-MB-231 cells (3×10^4 cells/well) were seeded in 24-well plates and maintained for 24 h. Then, cells were further incubated with 10 μM Edu reagent for 4 h and performed according to the manufacture's instruction (Beyotime; Cat: C0071S, China).

Colony formation assay. The MDA-MB-231 cells (1×10^2 cells/well) were cultured in 12-well plates and allowed to adhere overnight. And cells were treated with or without product **8** for 7 days. Then, cells were fixed with 4% paraformaldehyde and stained with crystal violet (Beyotime; Cat: C0121, China). The number of colonies of MDA-MB-231 cells was counted.

3D cell culture. The MDA-MB-231 cells (1×10^3 cells/well) were cultured in 96-well plates of 3D cell culture (Primesurface; Cat: MS9096UZ, Japan) and allowed to adhere overnight. And cells were treated with or without product **8** for 7 days. Then, cells were stained with Hoechst 33342 (Beyotime; Cat: C1022, China) and captured using a fluorescence microscope.

Calcein/PI staining. According to the instructions provided by the supplier (Beyotime; Cat: C2015M, China), the Calcein and PI staining solution were added into the 3D cell balls, incubated at 37 degrees for 30 minutes, and tested under an inverted fluorescence microscope.

Immunoblotting analysis. The MDA-MB-231 cells and animal tumors as well as lung tissues were collected and lysed by lysis buffer at 4 °C for 30 min. And sequentially centrifugation (12000 rpm for 10 min) and quantified (Bio-Rad Laboratories, Hercules, CA, USA). Equal amounts of the total protein were separated by 10-15% SDS-PAGE and transferred to PVDF membranes. After blocking with 5% nonfat dried milk, the proteins of membranes were detected using primary antibodies, followed by HRP-conjugated secondary antibodies, and visualized by employing ECL as the HRP substrate.

Wound heading. The MDA-MB-231 cells (20×10^4 cells/well) were seeded in 6-well plates and cultured in a serum-free medium for 24 h. Then, the cells were scratch-wounded by a plastic pipette tip and cultured with or without normal medium or product **8**. The wound healing rate of cells was observed by a phase-contrast microscope at the indicated time.

Invasion assay. The MDA-MB-231 cells (2×10^4 cells/well) were seeded on transwell filters (8 μm pore, Corning). Inoculate serum-free DMEM medium in the top chamber, and add DMEM supplemented with 5% FBS in the bottom chamber. Cells were allowed to migrate/invade for 12 h at 37°C. Cells on the bottom of filter were fixed in 4% paraformaldehyde and stained with 0.1% crystal violet. Images were taken under an inverted microscope and quantified by Image J.

5.2 Supplementary figures

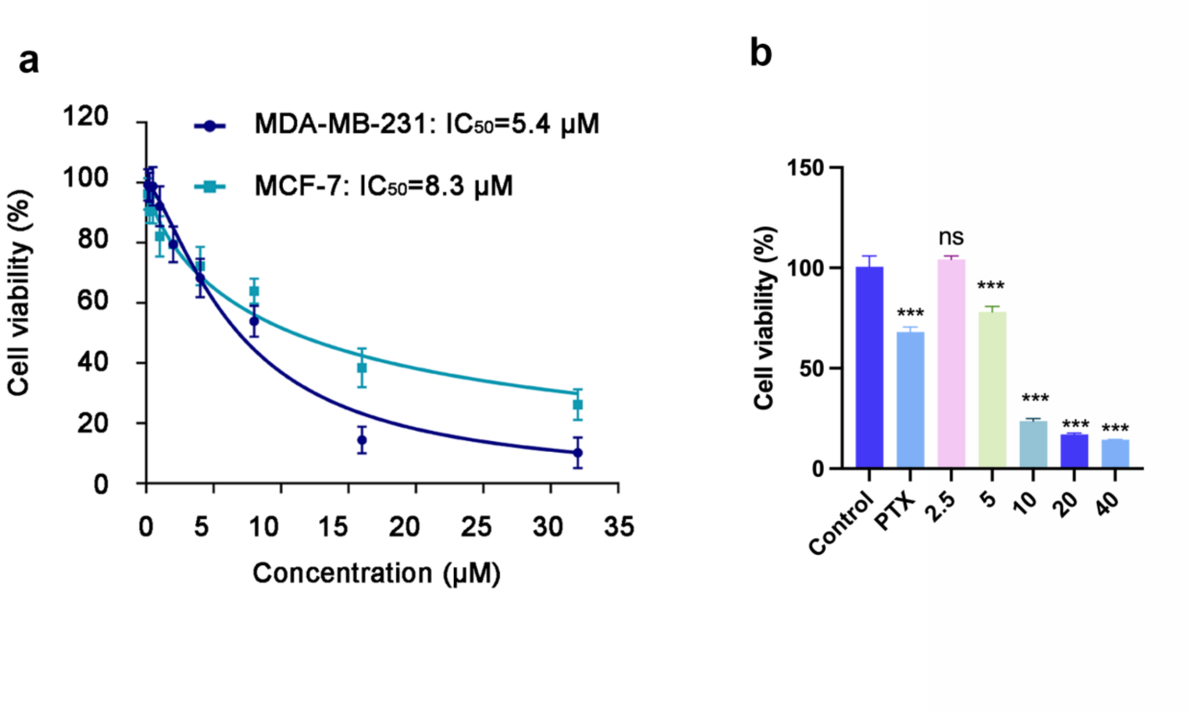

Figure S1. Antiproliferative activity of product **8. (a, b)** Cell viabilities were measured by MTT assay, and the IC_{50} values were calculated by Prism 8.0. Data represent mean ± SD.PTX: Paclitaxel (10 µM).

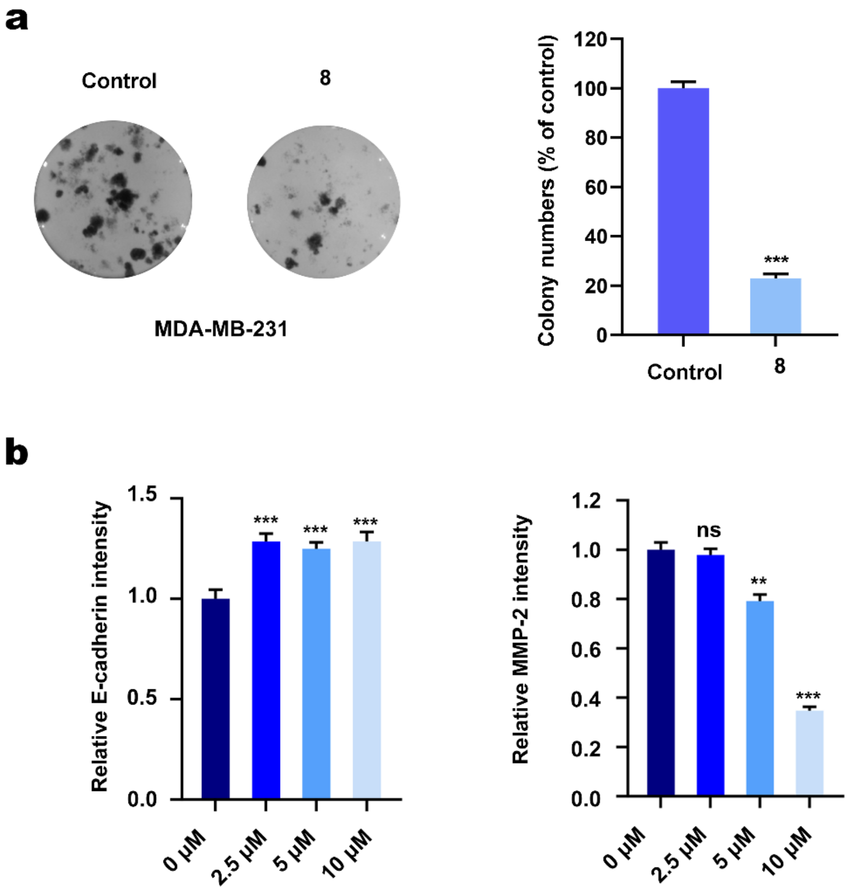

Figure S2. Product **8** inhibits cell proliferation and migration in MDA-MB-231 cells. **(a)** Colony formation assay of MDA-MB-231 cells treated with or without product **8** (5 μM). Representative images and quantification of colonies were shown. (**b)** Quantification of immunoblotting analysis of E-cadherin and MMP-2 was shown. Data represent mean ± SD. *p< 0.05, **p< 0.01, ***p< 0.001 compared with the control groups.

5.3 Raw images of Western blot assays

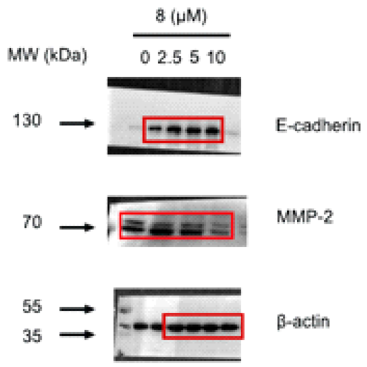

6. References and Notes

1. E. Krell, Handbook of Laboratory Distillation, Elseriver Publishing Company, Amsterdam-London-New York 1963.

2. M. J. Rosengart, The Technique of Distillation and Rectification in the Laboratory, VEB Verlag Technik, Berlin 1954.

3. F. Stage, Die Kolonnen zur Laboratoriumsdestillation. Eine Übersicht über den Entwicklungsstand der Kolonnen zur Destillation im Laboratorium. *Angew. Chem.* **1947**, *19*, 175–183.

4. X. Zhu, W. Deng, M. F. Chiou, C. Ye, W. Jian, Y. Zeng, Y. Jiao, L. Ge, Y. Li, X. Zhang, H. Bao, Copper-Catalyzed Radical 1,4-Difunctionalization of 1,3-Enynes with Alkyl Diacyl Peroxides and *N*-Fluorobenzenesulfonimide. *J. Am. Chem. Soc.* **2019**, *141*, 548−559.

5. K. F. Zhang, K. J. Bian, C. Li, J. Sheng, Y. Li, X. S. Wang, Nickel-Catalyzed Carbofluoroalkylation of 1,3-Enynes to Access Structurally Diverse Fluoroalkylated Allenes. *Angew. Chem. Int. Ed.* **2019**, *58*, 5069–5074.

7. Copies of ^{1}H, ^{13}C and ^{19}F NMR Spectra

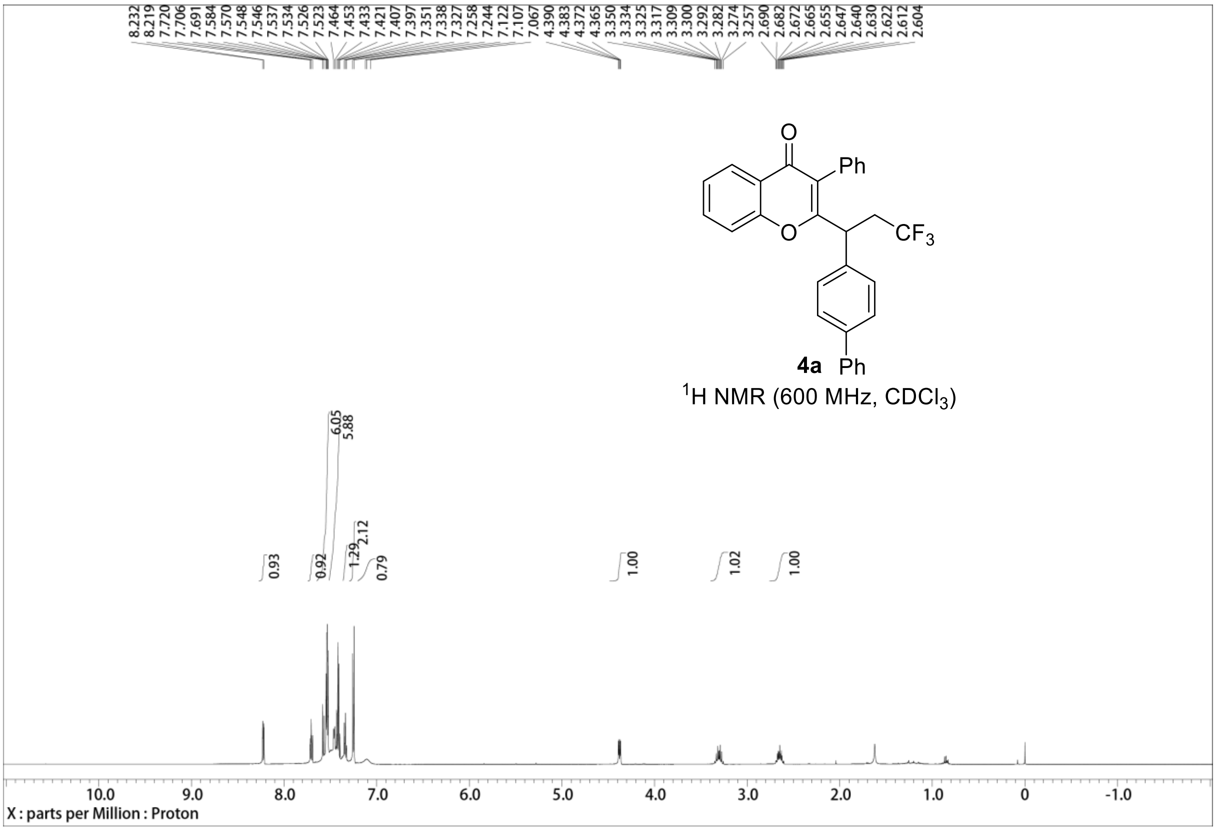

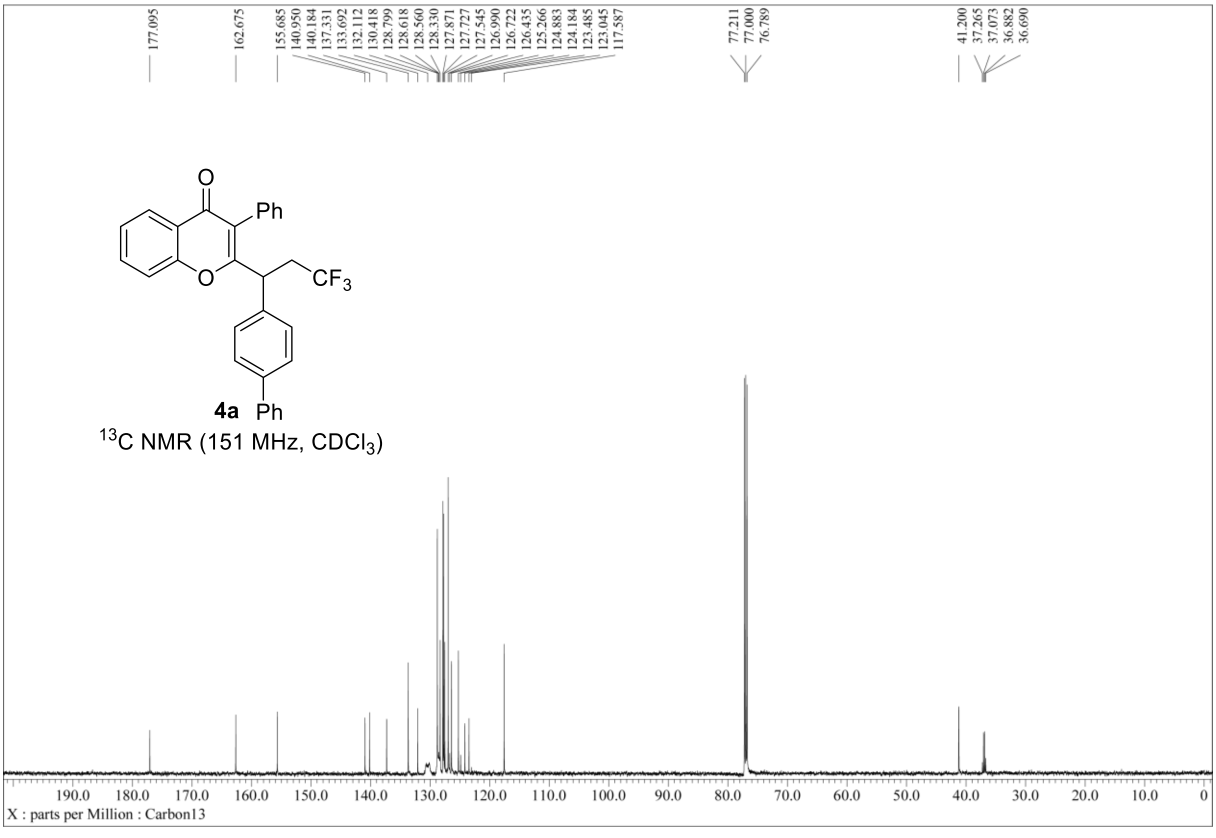

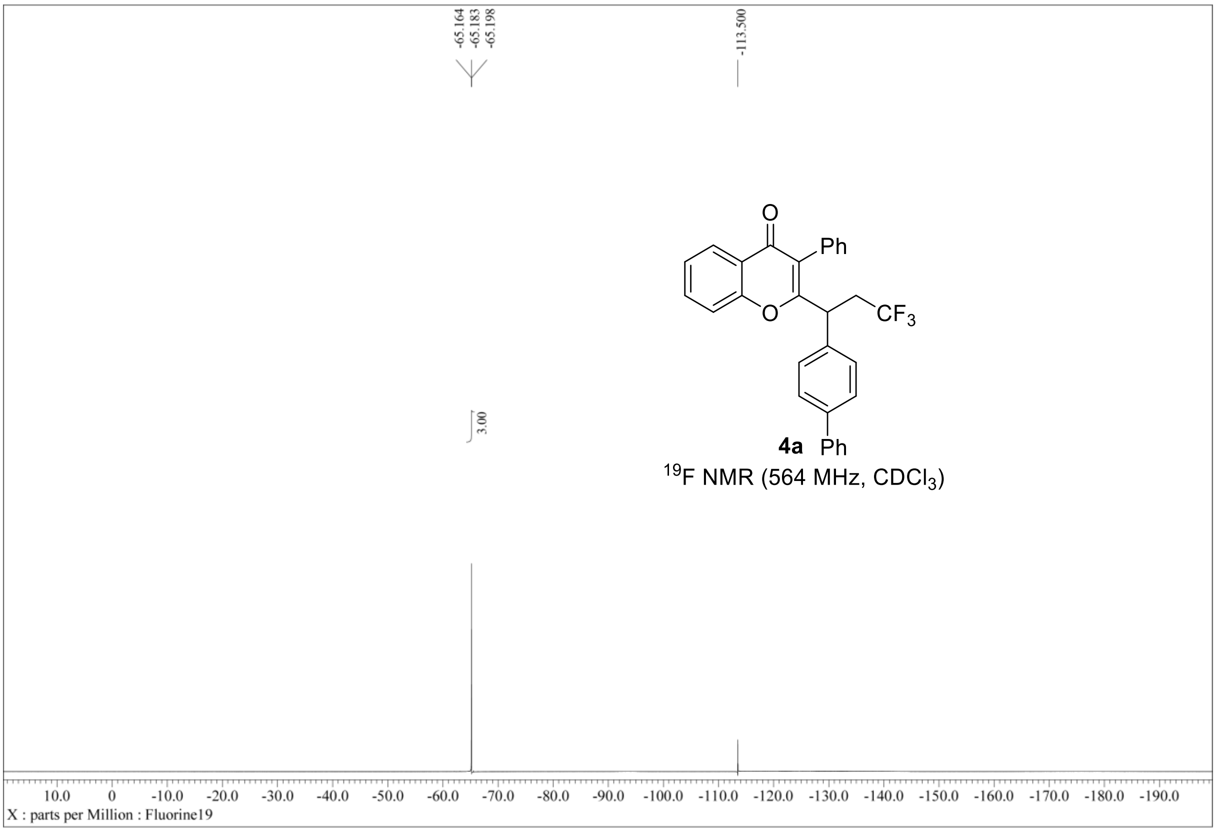

-65.164
-65.183
-65.198
-113.500
3.00
O
Ph
O
CF3
4a Ph
19F NMR (564 MHz, CDCl3)
10.0 0 -10.0 -20.0 -30.0 -40.0 -50.0 -60.0 -70.0 -80.0 -90.0 -100.0 -110.0 -120.0 -130.0 -140.0 -150.0 -160.0 -170.0 -180.0 -190.0
X : parts per Million : Fluorine19

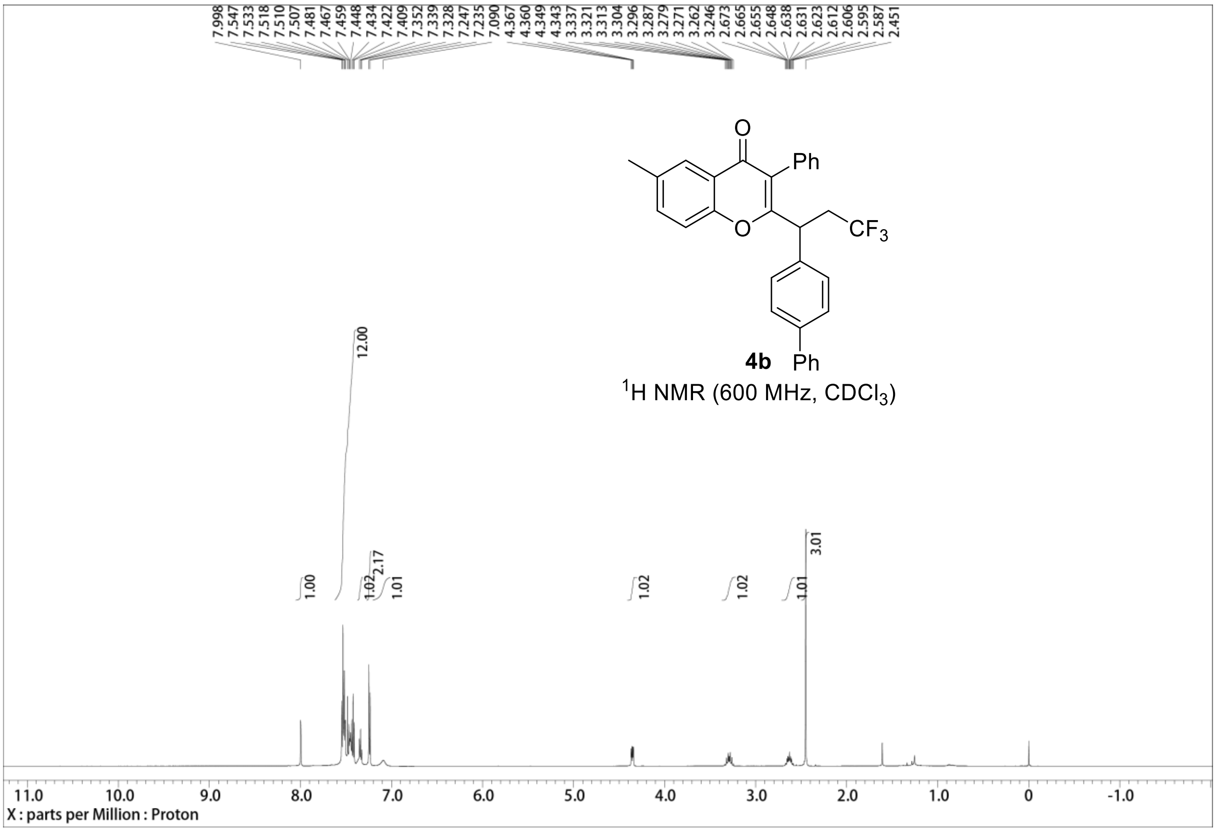

7.998 7.547 7.533 7.518 7.510 7.507 7.481 7.467 7.459 7.448 7.434 7.422 7.409 7.352 7.339 7.328 7.247 7.235 7.090 4.367 4.360 4.349 4.343 3.337 3.321 3.313 3.304 3.296 3.287 3.279 3.271 3.262 3.246 2.673 2.665 2.655 2.648 2.638 2.631 2.623 2.612 2.606 2.595 2.587 2.451
O
Ph
O
CF3
4b Ph
1H NMR (600 MHz, CDCl3)
12.00
1.00
1.02
2.17
1.01
1.02
1.02
1.01
3.01
11.0 10.0 9.0 8.0 7.0 6.0 5.0 4.0 3.0 2.0 1.0 0 -1.0
X : parts per Million : Proton

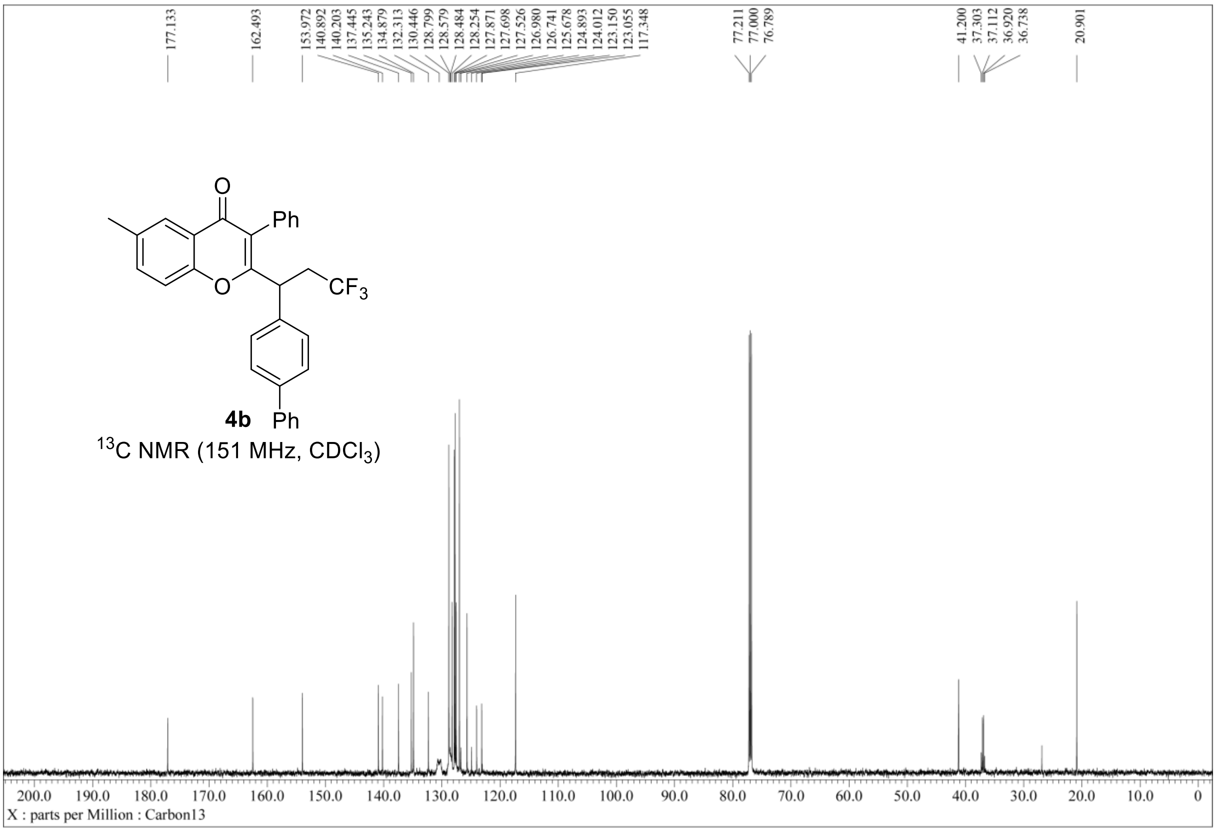
177.133
162.493
153.972
140.892
140.203
137.445
135.243
134.879
132.313
130.446
128.799
128.579
128.484
128.254
127.871
127.698
127.526
126.980
126.741
125.678
124.893
124.012
123.150
123.055
117.348
77.211
77.000
76.789
41.200
37.303
37.112
36.920
36.738
20.901
O
Ph
O
CF3
4b
Ph
13C NMR (151 MHz, CDCl3)
X : parts per Million : Carbon13

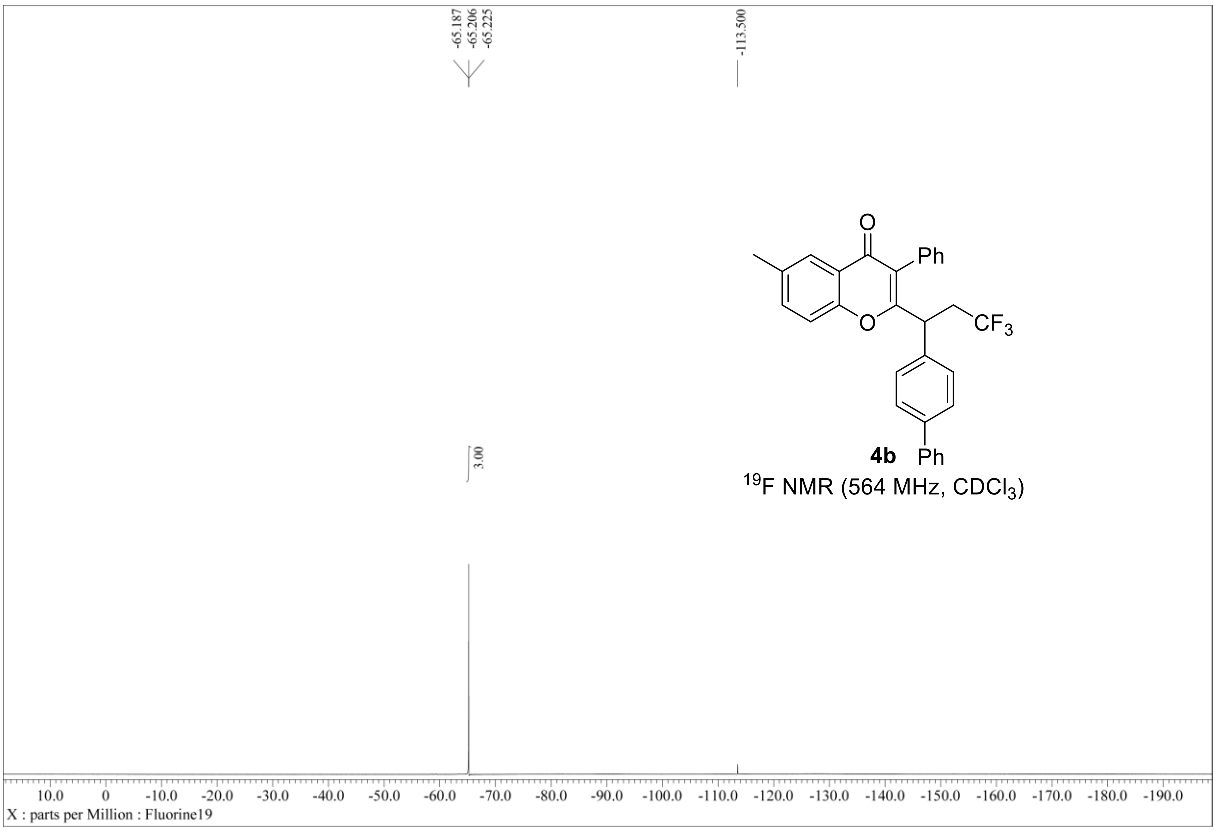
-65.187
-65.206
-65.225
-113.500
3.00
O
Ph
O
CF3
4b
Ph
19F NMR (564 MHz, CDCl3)
X : parts per Million : Fluorine19

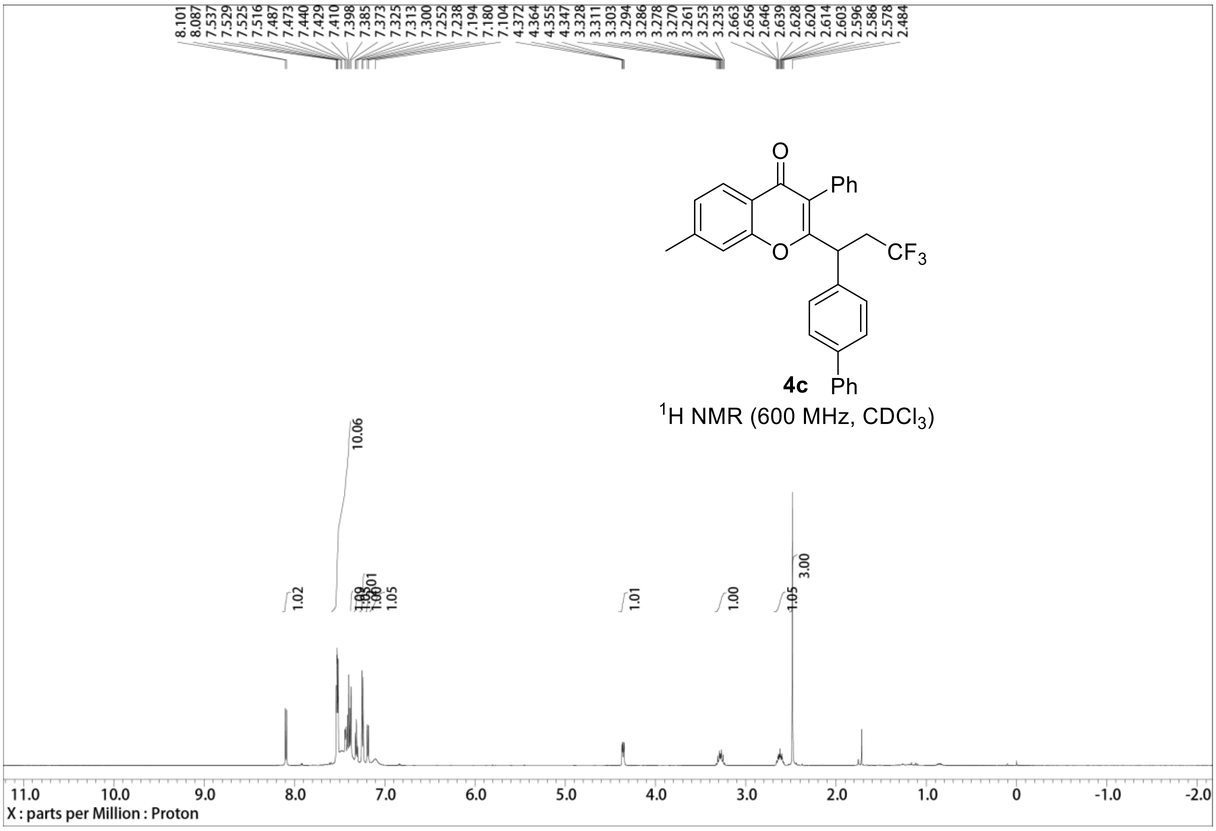

8.101 8.087 7.537 7.529 7.525 7.516 7.487 7.473 7.440 7.429 7.410 7.398 7.385 7.373 7.325 7.313 7.300 7.252 7.238 7.194 7.180 7.104 4.372 4.364 4.355 4.347 3.328 3.311 3.303 3.294 3.286 3.278 3.270 3.261 3.253 3.235 2.663 2.656 2.646 2.639 2.628 2.620 2.614 2.603 2.596 2.586 2.578 2.484
O
Ph
O
CF3
4c
Ph
1H NMR (600 MHz, CDCl3)
1.02
10.06
1.05
1.01
1.00
1.05
3.00
11.0 10.0 9.0 8.0 7.0 6.0 5.0 4.0 3.0 2.0 1.0 0 -1.0 -2.0
X : parts per Million : Proton

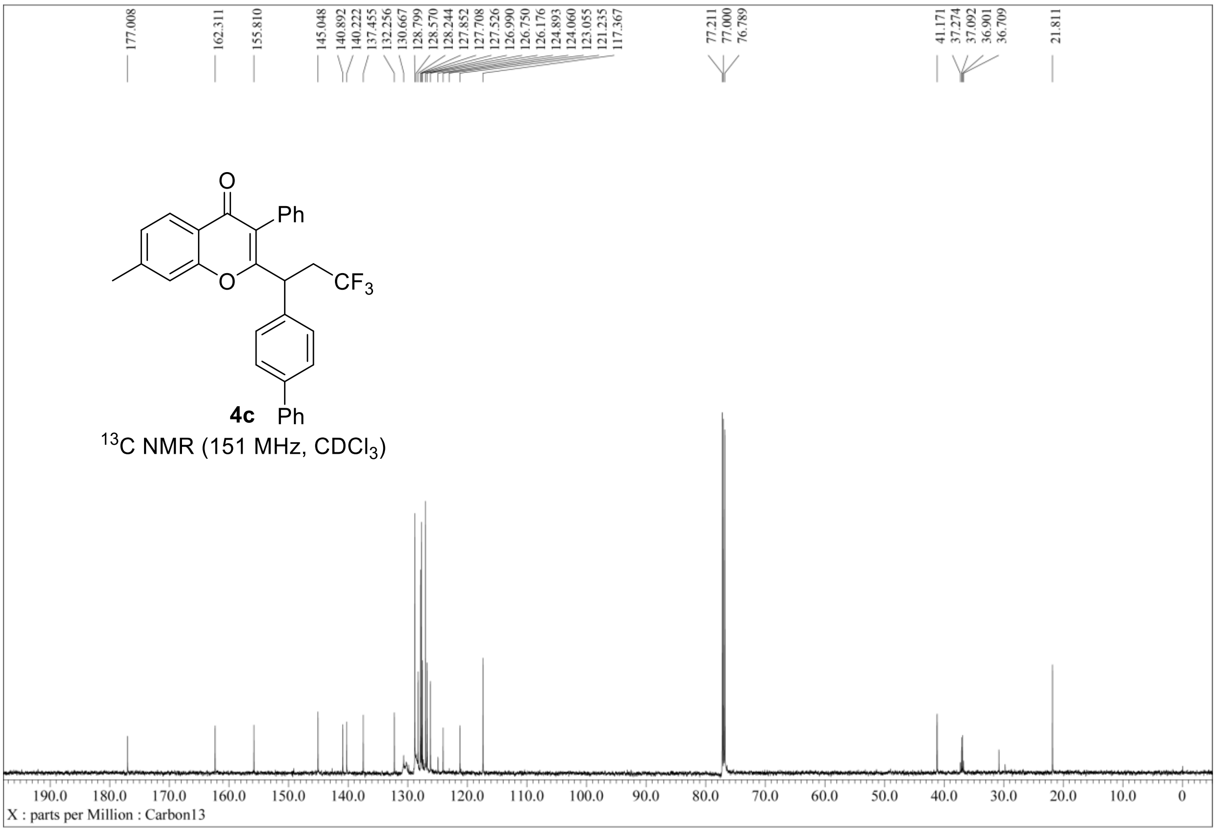

177.008 162.311 155.810 145.048 140.892 140.222 137.455 132.256 130.667 128.799 128.570 128.244 127.852 127.708 127.526 126.990 126.750 126.176 124.893 124.060 123.055 121.235 117.367 77.211 77.000 76.789 41.171 37.274 37.092 36.901 36.709 21.811
O
Ph
O
CF3
4c
Ph
13C NMR (151 MHz, CDCl3)
190.0 180.0 170.0 160.0 150.0 140.0 130.0 120.0 110.0 100.0 90.0 80.0 70.0 60.0 50.0 40.0 30.0 20.0 10.0 0
X : parts per Million : Carbon13

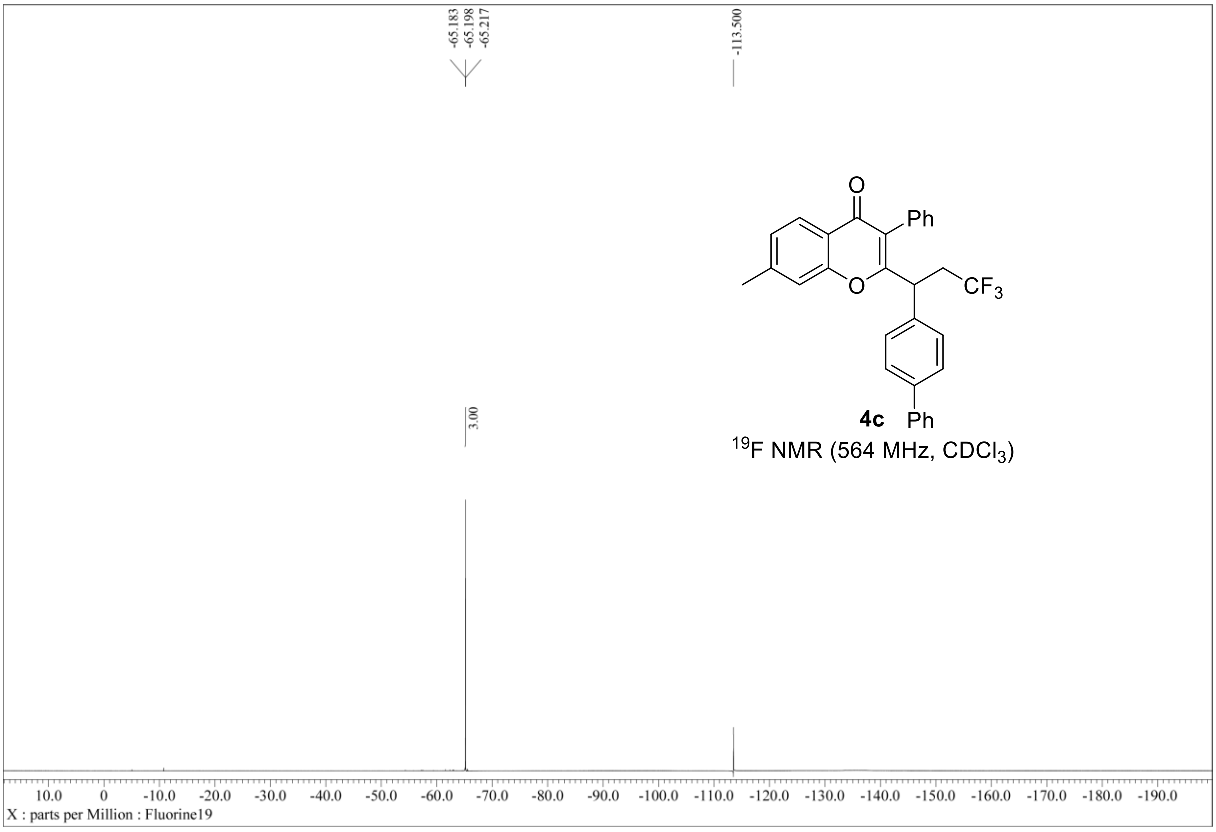
-65.183
-65.198
-65.217
-113.500
3.00
O
Ph
O
CF3
Ph
4c
19F NMR (564 MHz, CDCl3)
X : parts per Million : Fluorine19

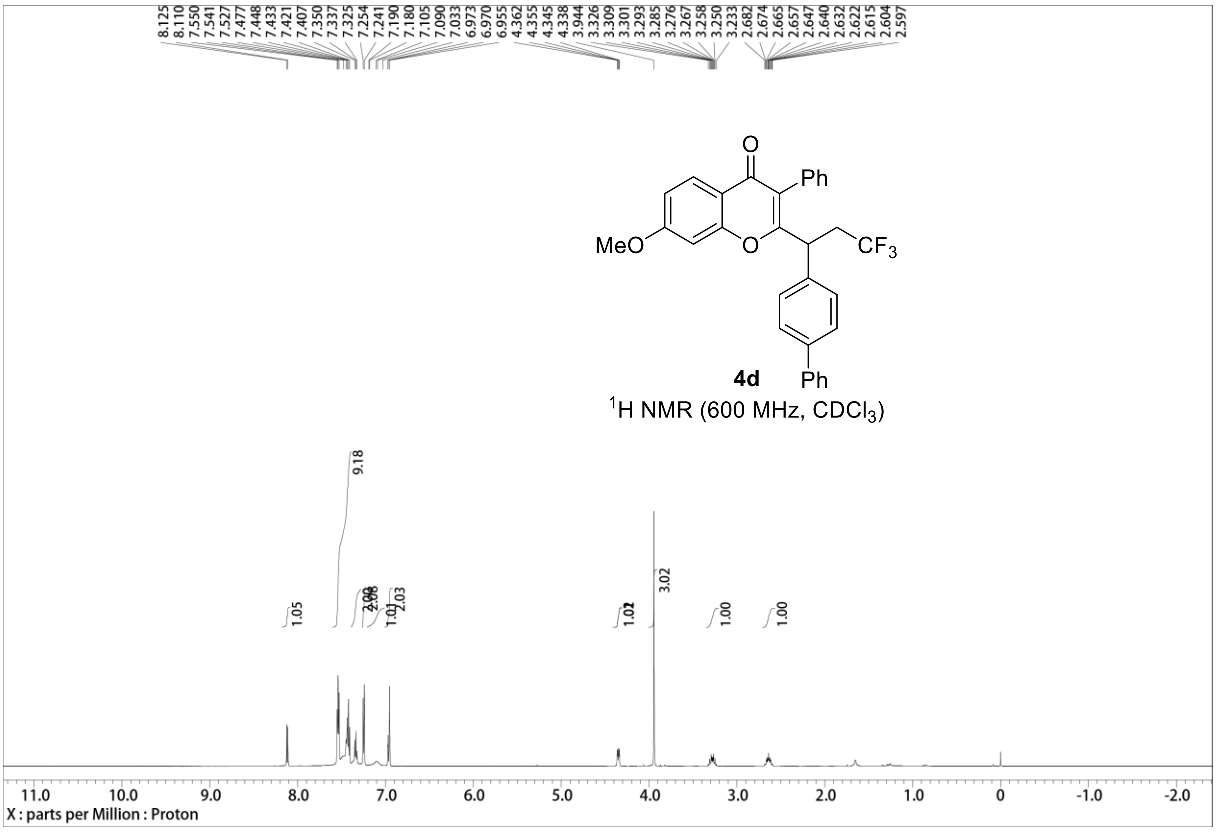
O
Ph
MeO
O
CF3
Ph
4d
1H NMR (600 MHz, CDCl3)
9.18
3.02
X : parts per Million : Proton

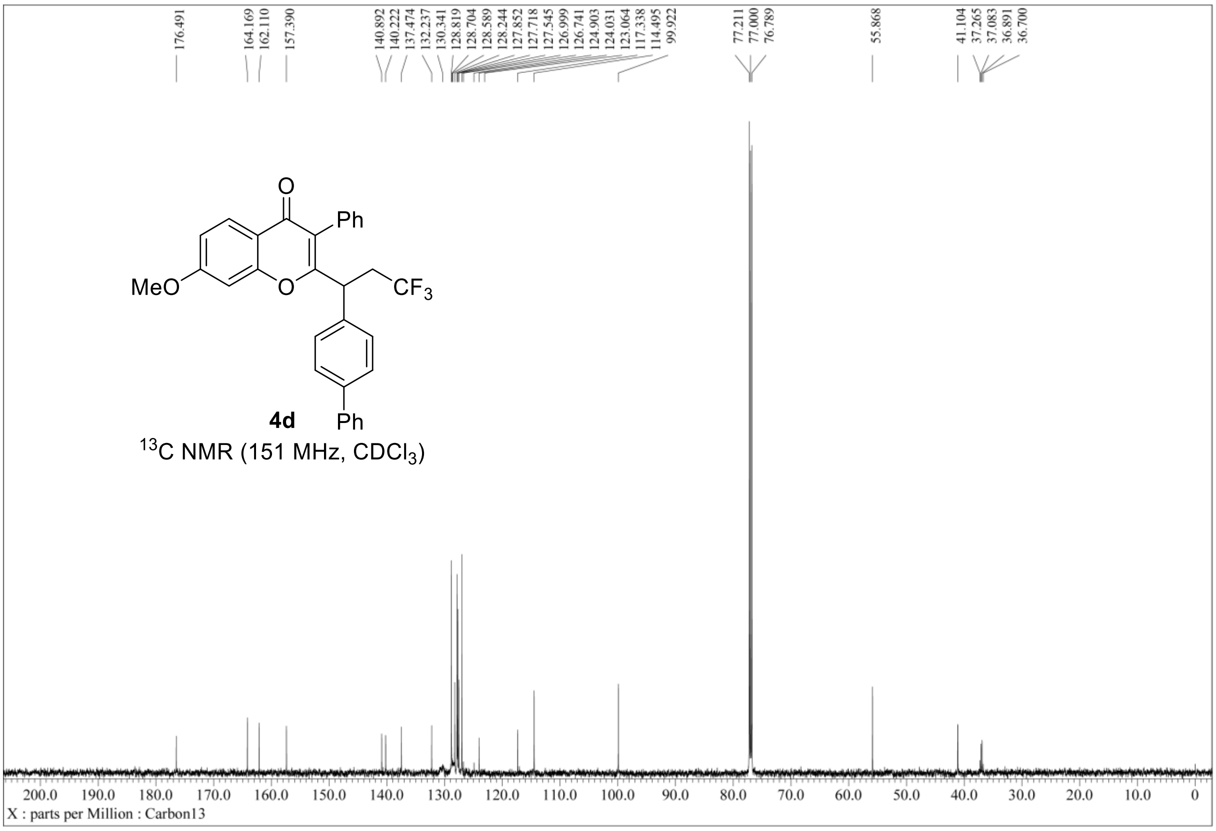

176.491
164.169
162.110
157.390
140.892
140.222
137.474
132.237
130.341
128.819
128.704
128.589
128.244
127.852
127.718
127.545
126.999
126.741
124.903
124.031
123.064
117.338
114.495
99.922
77.211
77.000
76.789
55.868
41.104
37.265
37.083
36.891
36.700
O
Ph
MeO
O
CF3
Ph
4d
13C NMR (151 MHz, CDCl3)
X : parts per Million : Carbon13

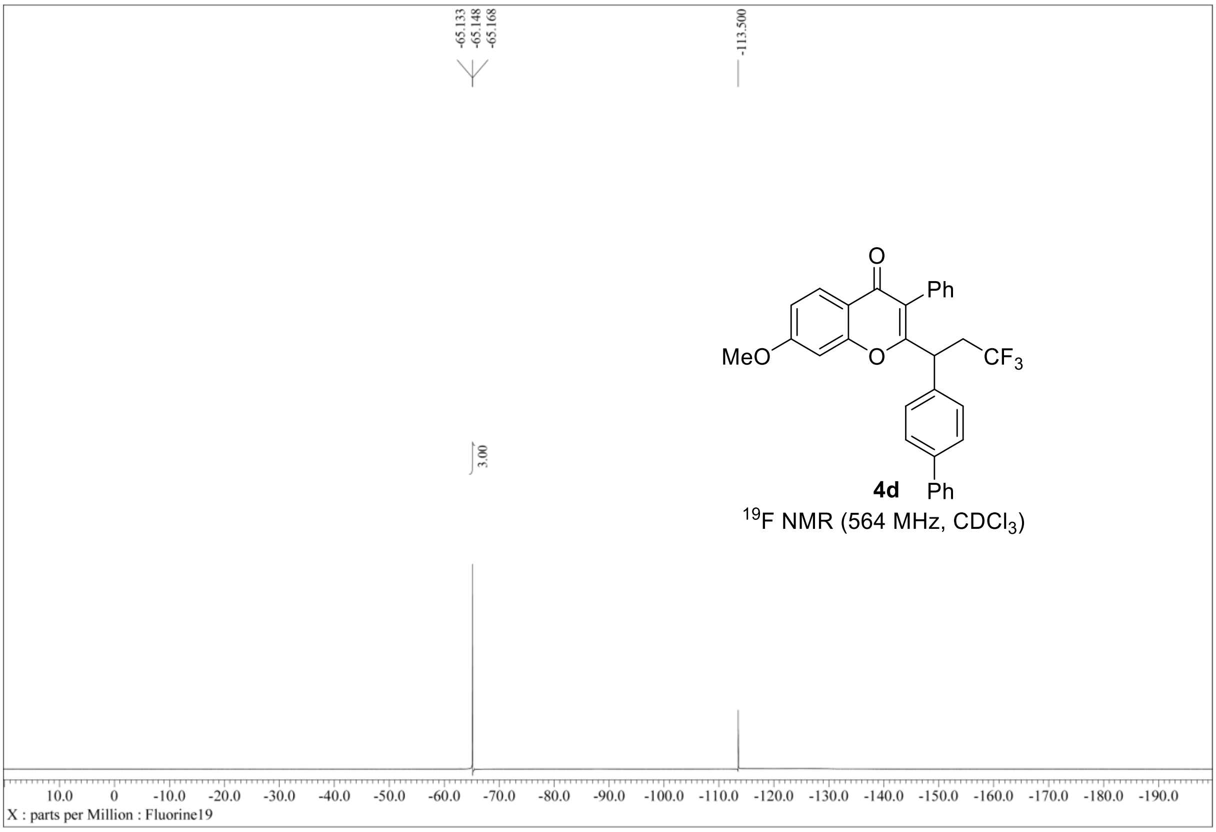

-65.133
-65.148
-65.168
-113.500
3.00
O
Ph
MeO
O
CF3
Ph
4d
19F NMR (564 MHz, CDCl3)
X : parts per Million : Fluorine19

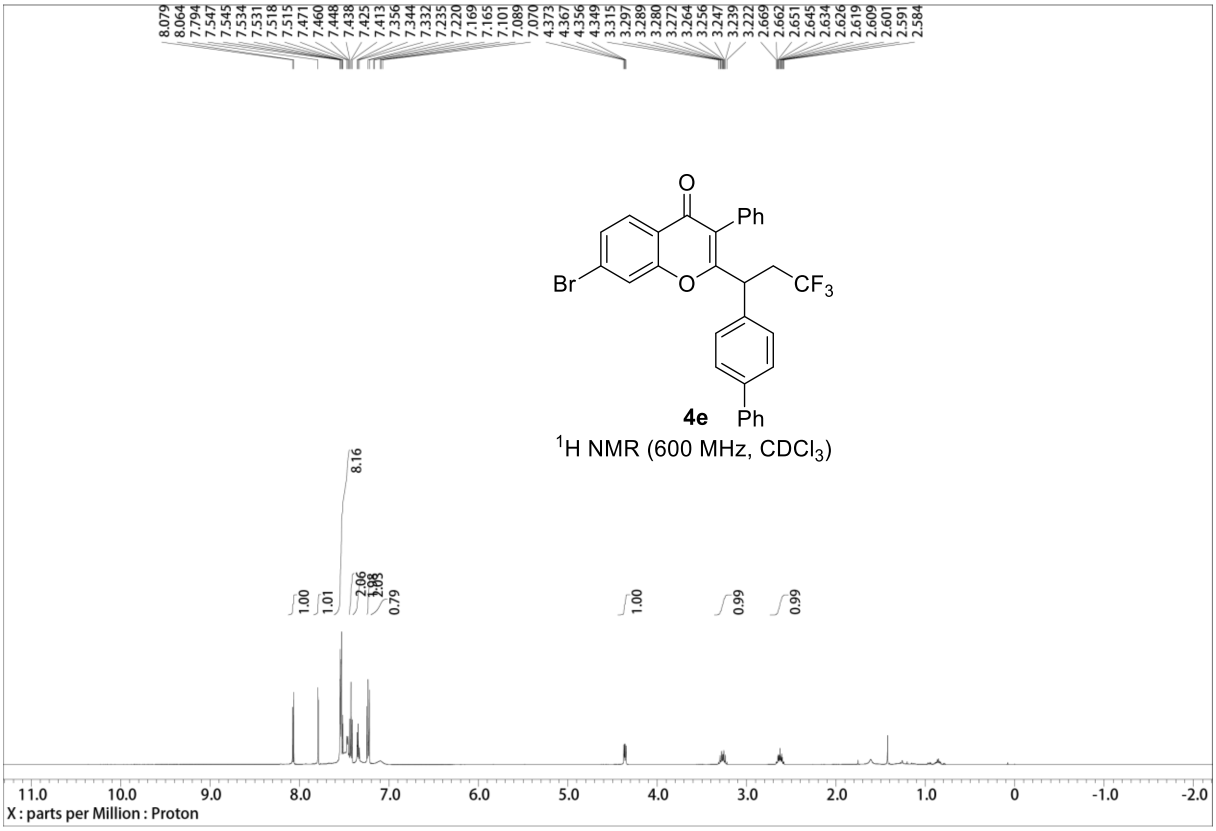
8.079 8.064 7.794 7.547 7.545 7.534 7.531 7.518 7.515 7.471 7.460 7.448 7.438 7.425 7.413 7.356 7.344 7.332 7.235 7.220 7.169 7.165 7.101 7.089 7.070 4.373 4.367 4.356 4.349 3.315 3.297 3.289 3.280 3.272 3.264 3.256 3.247 3.239 3.222 2.669 2.662 2.651 2.645 2.634 2.626 2.619 2.609 2.601 2.591 2.584
O
Ph
Br
O
CF3
4e
Ph
1H NMR (600 MHz, CDCl3)
8.16
1.00 1.01 2.06 1.98 2.03 0.79
1.00
0.99
0.99
11.0 10.0 9.0 8.0 7.0 6.0 5.0 4.0 3.0 2.0 1.0 0 -1.0 -2.0
X : parts per Million : Proton

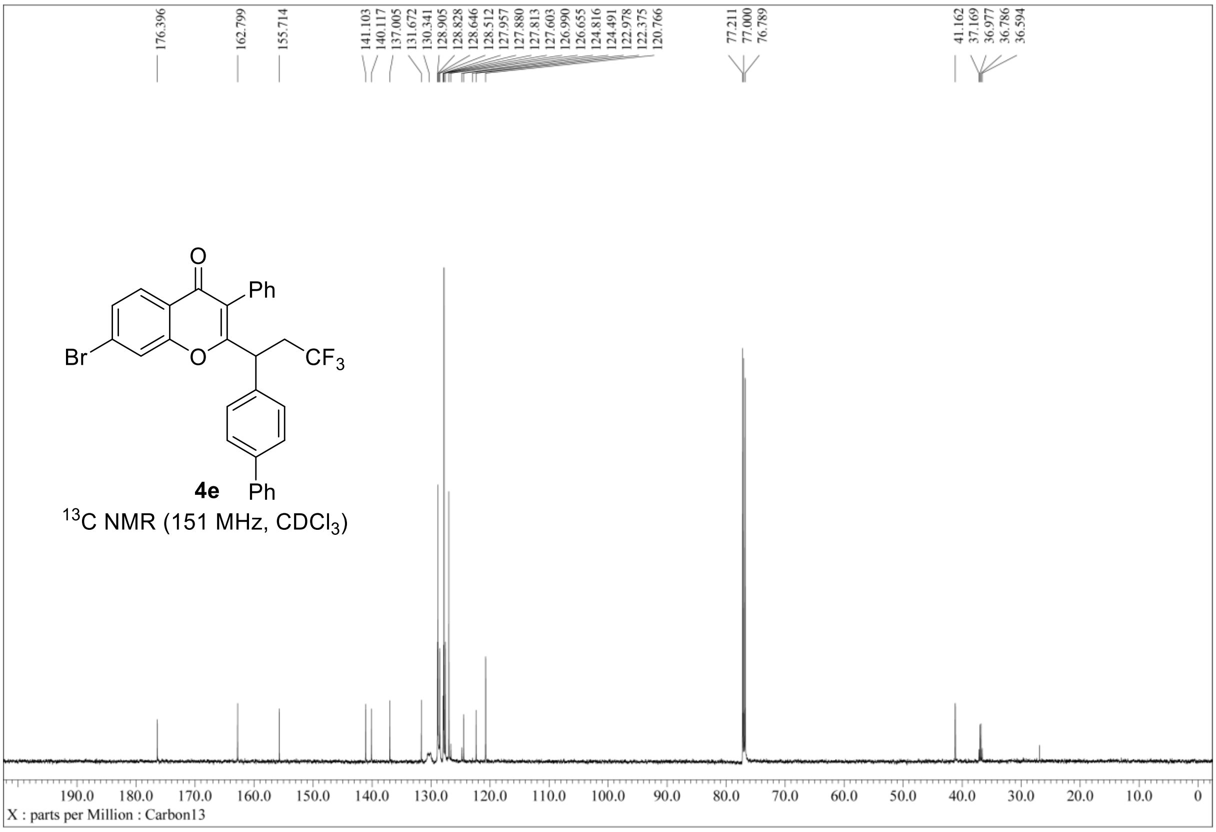
176.396
162.799
155.714
141.103 140.117 137.005 131.672 130.341 128.905 128.828 128.646 128.512 127.957 127.880 127.813 127.603 126.990 126.655 124.816 124.491 122.978 122.375 120.766
77.211 77.000 76.789
41.162 37.169 36.977 36.786 36.594
O
Ph
Br
O
CF3
4e
Ph
13C NMR (151 MHz, CDCl3)
190.0 180.0 170.0 160.0 150.0 140.0 130.0 120.0 110.0 100.0 90.0 80.0 70.0 60.0 50.0 40.0 30.0 20.0 10.0 0
X : parts per Million : Carbon13

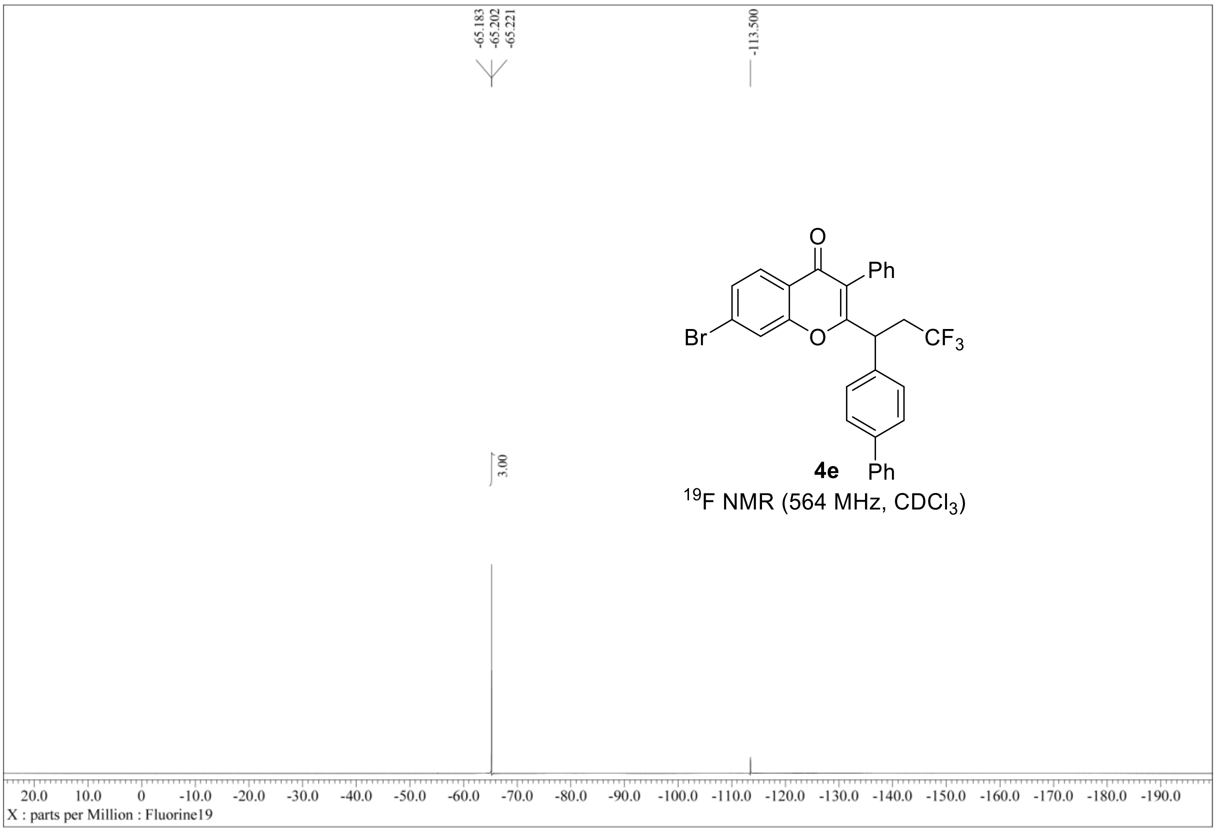
-65.183
-65.202
-65.221
-113.500
3.00
O
Ph
Br
O
CF3
Ph
4e
19F NMR (564 MHz, CDCl3)
20.0 10.0 0 -10.0 -20.0 -30.0 -40.0 -50.0 -60.0 -70.0 -80.0 -90.0 -100.0 -110.0 -120.0 -130.0 -140.0 -150.0 -160.0 -170.0 -180.0 -190.0
X : parts per Million : Fluorine19

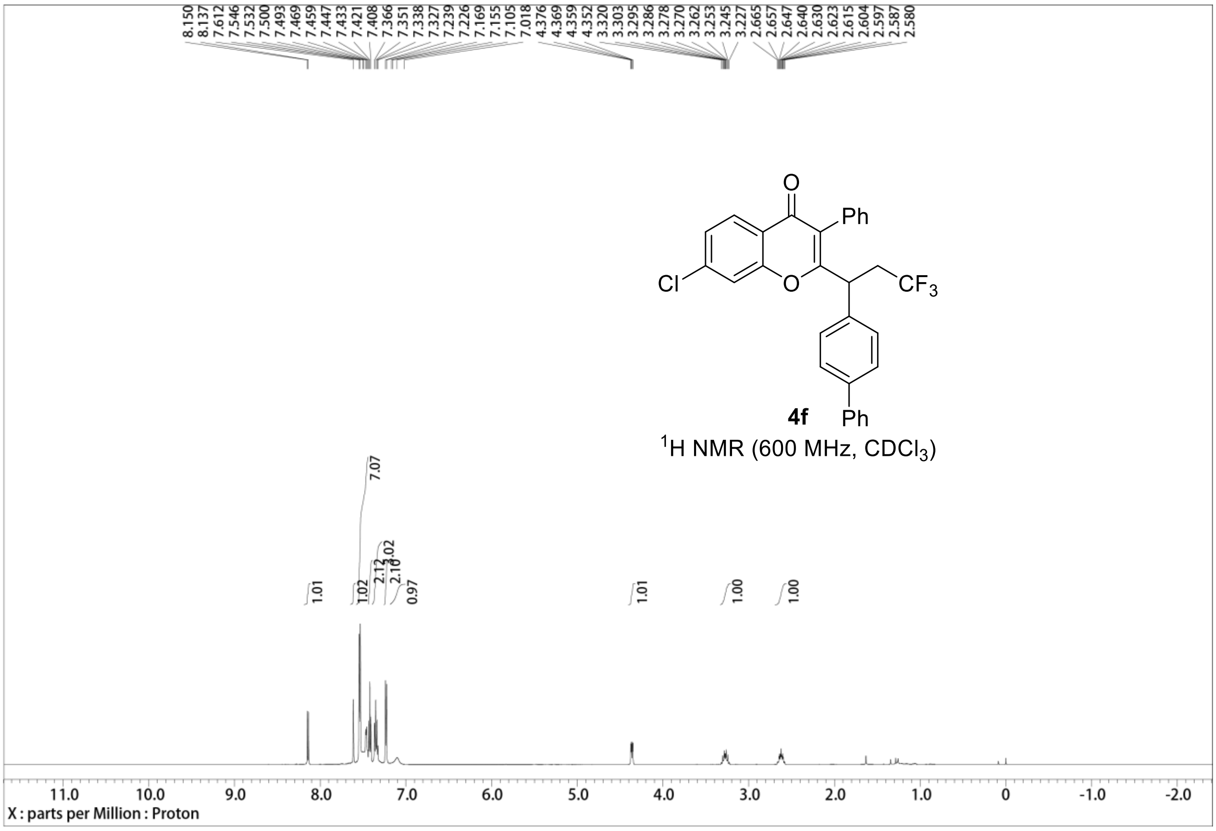
8.150 8.137 7.612 7.546 7.532 7.500 7.493 7.469 7.459 7.447 7.433 7.421 7.408 7.366 7.351 7.338 7.327 7.239 7.226 7.169 7.155 7.105 7.018 4.376 4.369 4.359 4.352 3.320 3.303 3.295 3.286 3.278 3.270 3.262 3.253 3.245 3.227 2.665 2.657 2.647 2.640 2.630 2.623 2.615 2.604 2.597 2.587 2.580
O
Ph
Cl
O
CF3
Ph
4f
1H NMR (600 MHz, CDCl3)
1.01
7.07
1.02
2.12
2.02
2.10
0.97
1.01
1.00
1.00
11.0 10.0 9.0 8.0 7.0 6.0 5.0 4.0 3.0 2.0 1.0 0 -1.0 -2.0
X : parts per Million : Proton

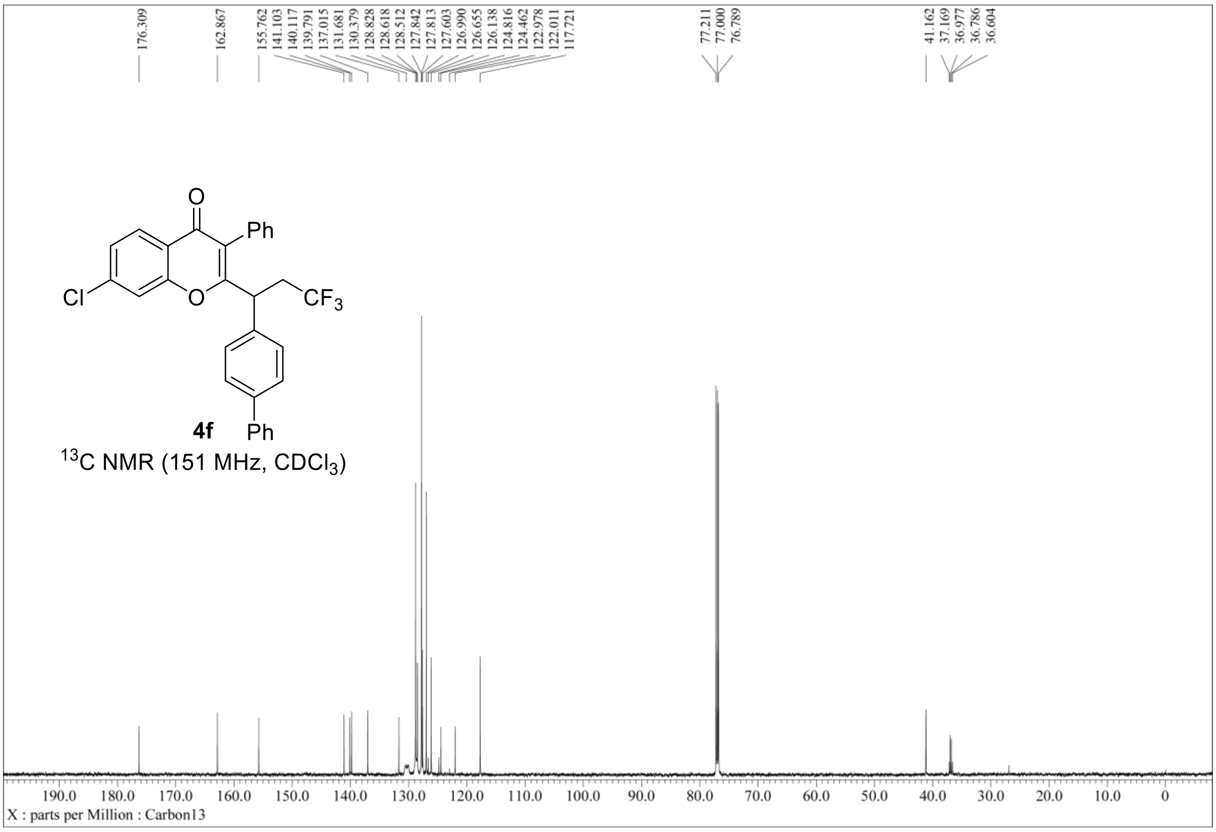

176.309
162.867
155.762
141.103
140.117
139.791
137.015
131.681
130.379
128.828
128.618
128.512
127.842
127.813
127.603
126.990
126.655
126.138
124.816
124.462
122.978
122.011
117.721
77.211
77.000
76.789
41.162
37.169
36.977
36.786
36.604
O
Ph
Cl
O
CF3
4f
Ph
13C NMR (151 MHz, CDCl3)
190.0 180.0 170.0 160.0 150.0 140.0 130.0 120.0 110.0 100.0 90.0 80.0 70.0 60.0 50.0 40.0 30.0 20.0 10.0 0
X : parts per Million : Carbon13

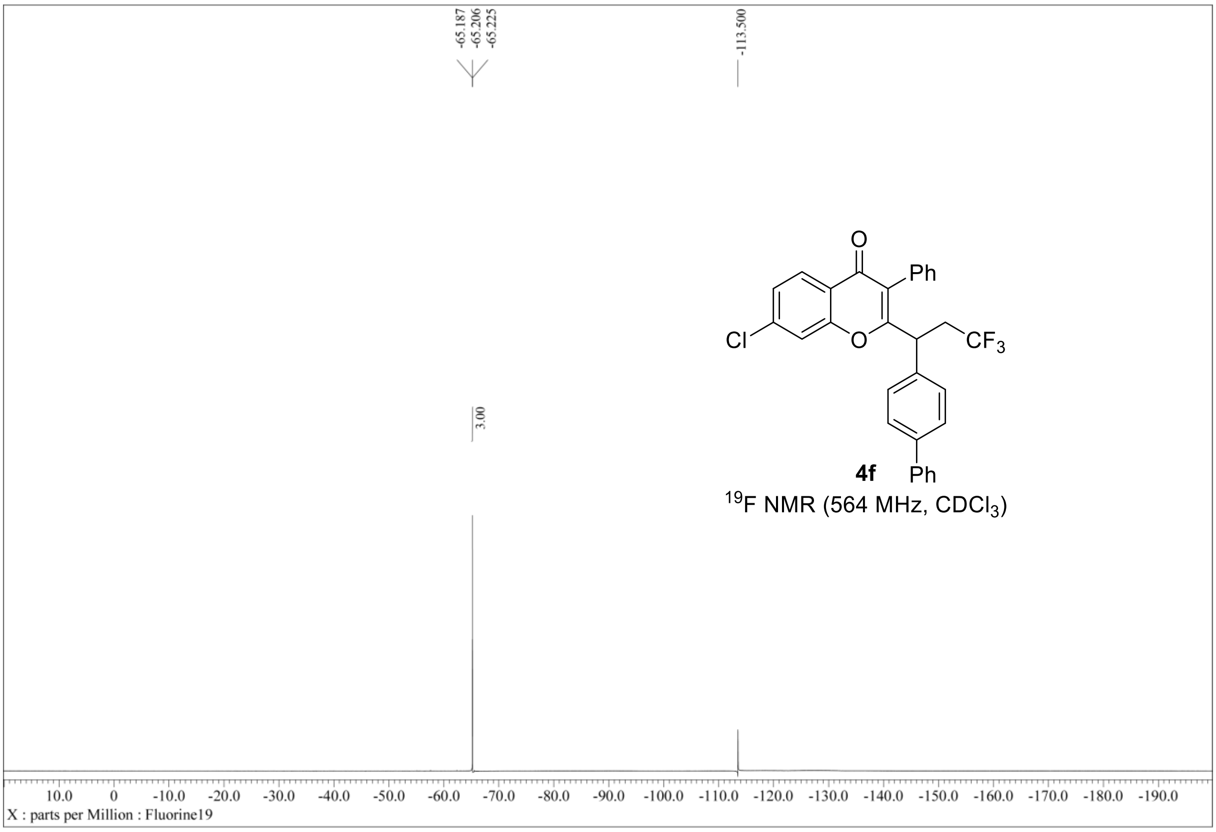

-65.187
-65.206
-65.225
-113.500
3.00
O
Ph
Cl
O
CF3
4f
Ph
19F NMR (564 MHz, CDCl3)
10.0 0 -10.0 -20.0 -30.0 -40.0 -50.0 -60.0 -70.0 -80.0 -90.0 -100.0 -110.0 -120.0 -130.0 -140.0 -150.0 -160.0 -170.0 -180.0 -190.0
X : parts per Million : Fluorine19

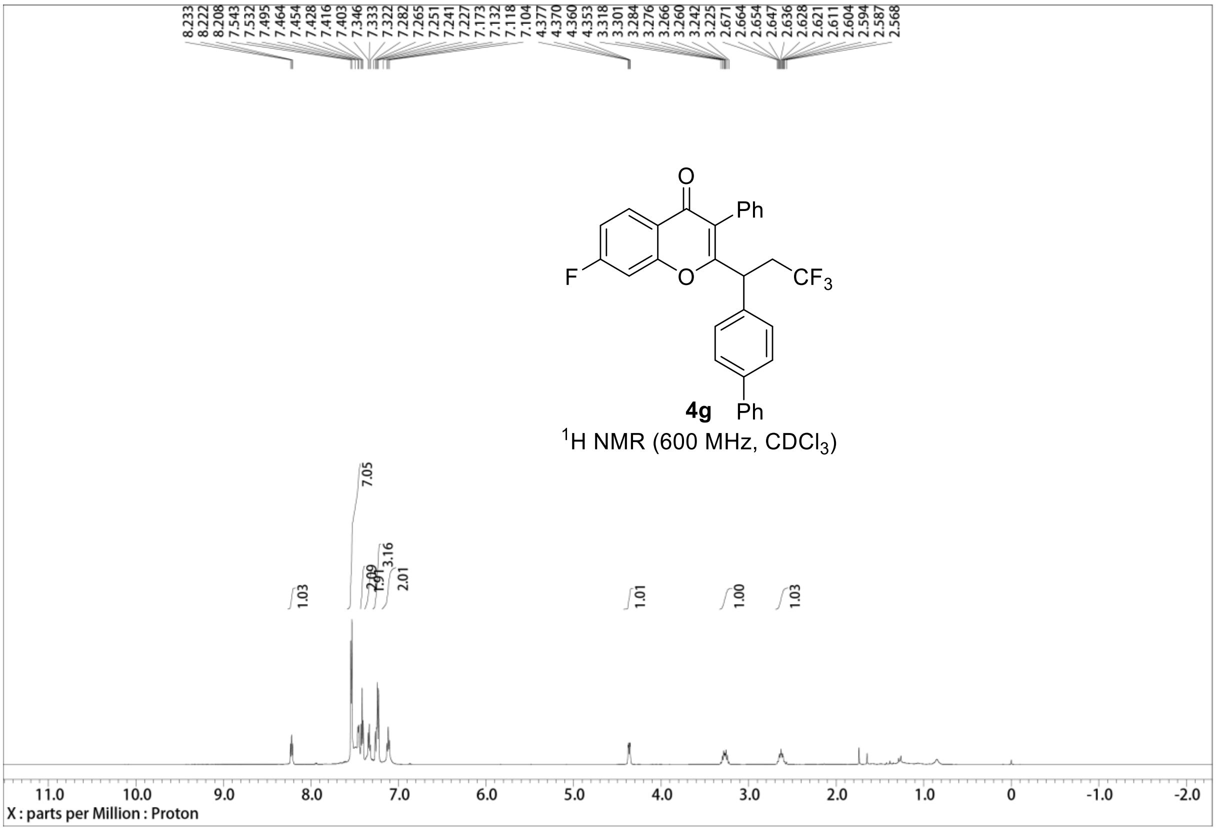
8.233 8.222 8.208 7.543 7.532 7.495 7.464 7.454 7.428 7.416 7.403 7.346 7.333 7.322 7.282 7.265 7.251 7.241 7.227 7.173 7.132 7.118 7.104 4.377 4.370 4.360 4.353 3.318 3.301 3.284 3.276 3.266 3.260 3.242 3.225 2.671 2.664 2.654 2.647 2.636 2.628 2.621 2.611 2.604 2.594 2.587 2.568
O
Ph
F
O
CF3
4g
Ph
1H NMR (600 MHz, CDCl3)
1.03
7.05
2.09
1.91
3.16
2.01
1.01
1.00
1.03
X : parts per Million : Proton

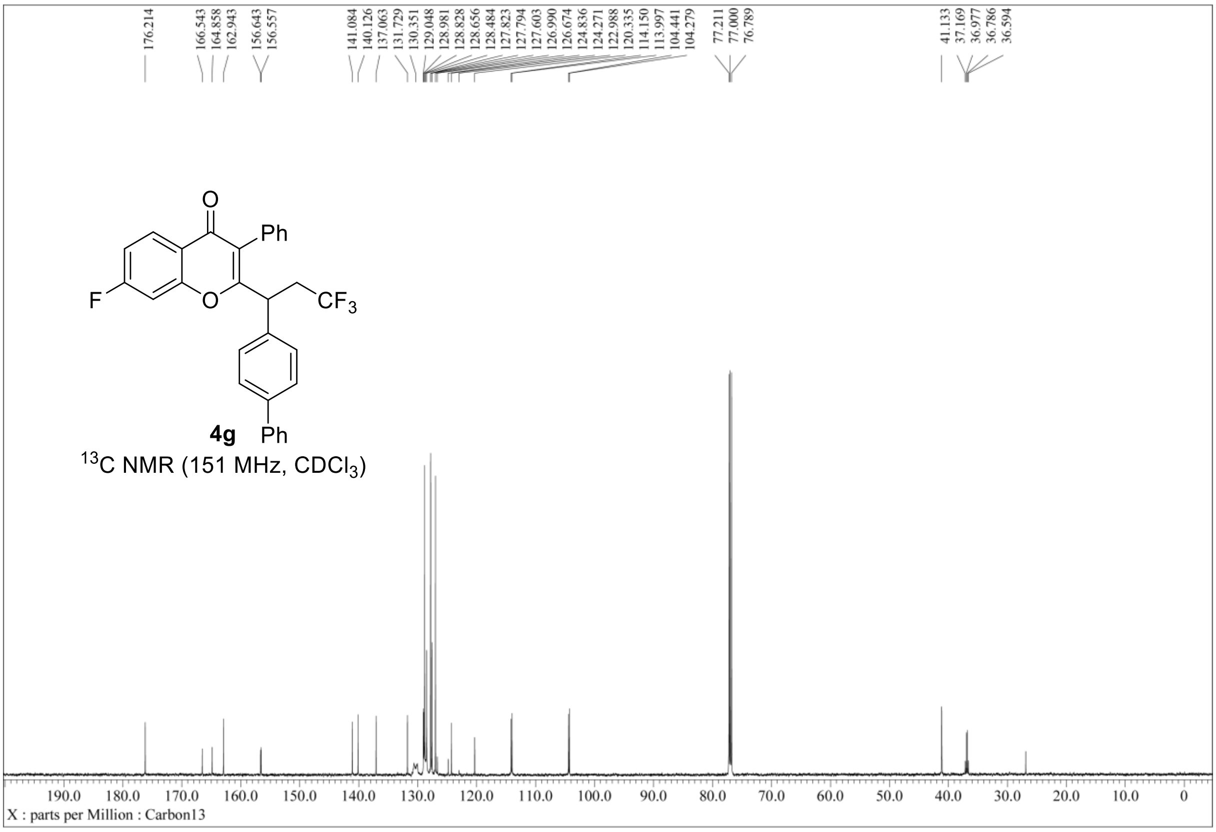
176.214 166.543 164.858 162.943 156.643 156.557 141.084 140.126 137.063 131.729 130.351 129.048 128.981 128.828 128.656 128.484 127.823 127.794 127.603 126.990 126.674 124.836 124.271 122.988 120.335 114.150 113.997 104.441 104.279 77.211 77.000 76.789 41.133 37.169 36.977 36.786 36.594
O
Ph
F
O
CF3
4g
Ph
13C NMR (151 MHz, CDCl3)
X : parts per Million : Carbon13

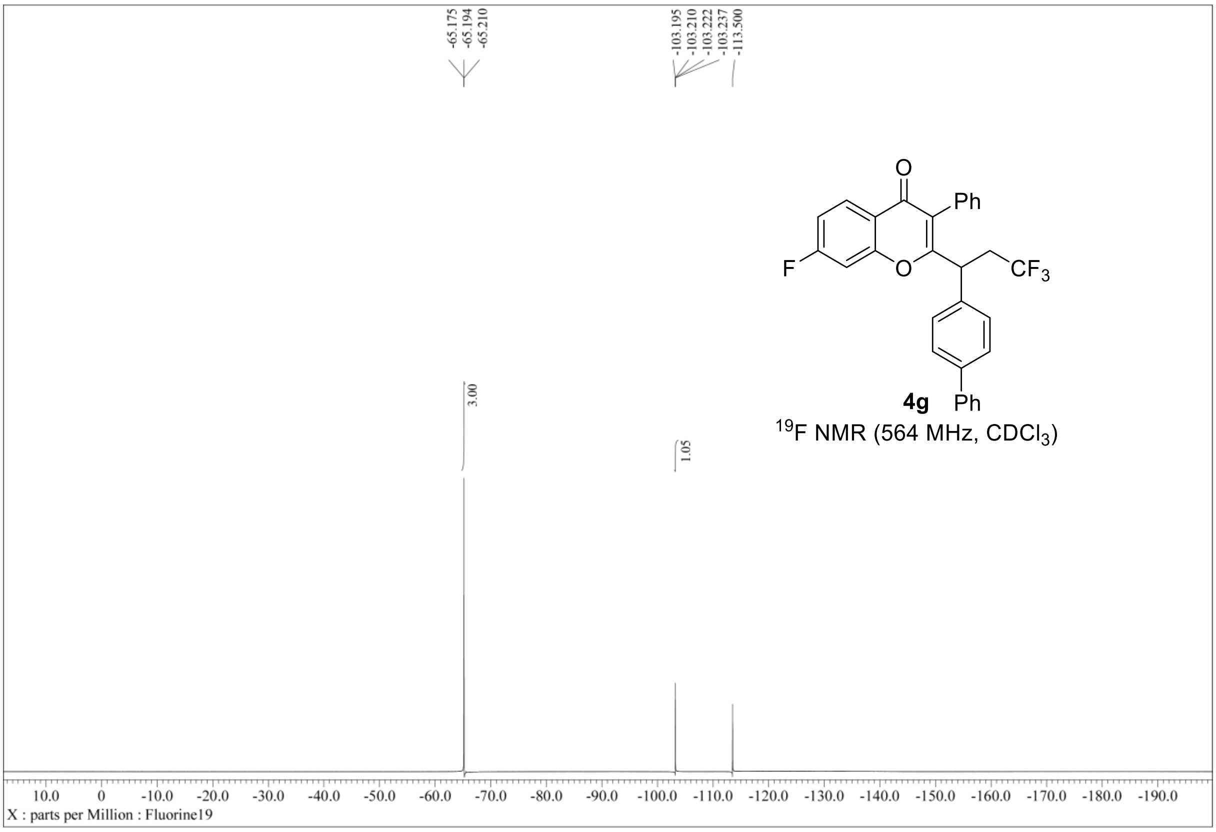
-65.175
-65.194
-65.210
-103.195
-103.210
-103.222
-103.237
-113.500
3.00
1.05
O
Ph
F
O
CF3
4g Ph
19F NMR (564 MHz, CDCl3)
10.0 0 -10.0 -20.0 -30.0 -40.0 -50.0 -60.0 -70.0 -80.0 -90.0 -100.0 -110.0 -120.0 -130.0 -140.0 -150.0 -160.0 -170.0 -180.0 -190.0
X : parts per Million : Fluorine19

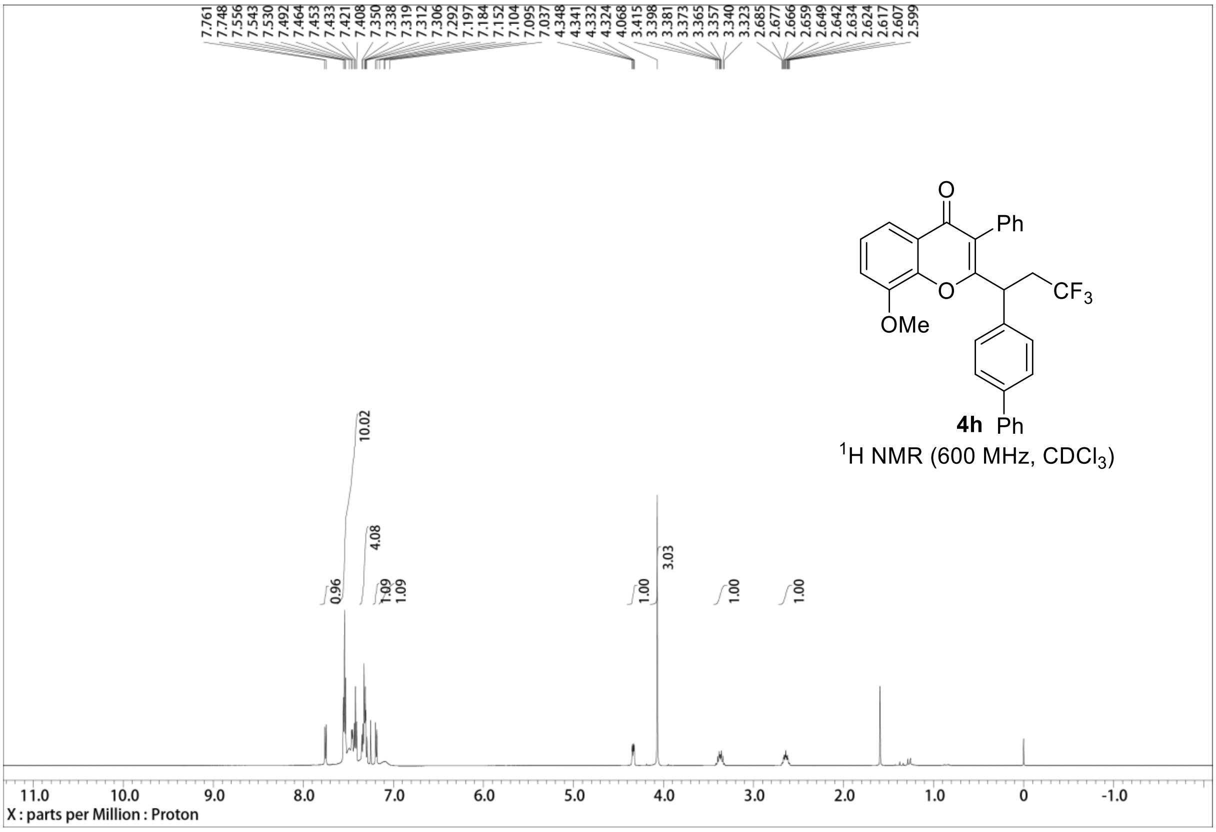
7.761
7.748
7.556
7.543
7.530
7.492
7.464
7.453
7.433
7.421
7.408
7.350
7.338
7.319
7.312
7.306
7.292
7.197
7.184
7.152
7.104
7.095
7.037
4.348
4.341
4.332
4.324
4.068
3.415
3.398
3.381
3.373
3.365
3.357
3.340
3.323
2.685
2.677
2.666
2.659
2.649
2.642
2.634
2.624
2.617
2.607
2.599
O
Ph
O
CF3
OMe
4h Ph
1H NMR (600 MHz, CDCl3)
10.02
4.08
0.96
1.09
1.09
1.00
3.03
1.00
1.00
11.0 10.0 9.0 8.0 7.0 6.0 5.0 4.0 3.0 2.0 1.0 0 -1.0
X : parts per Million : Proton

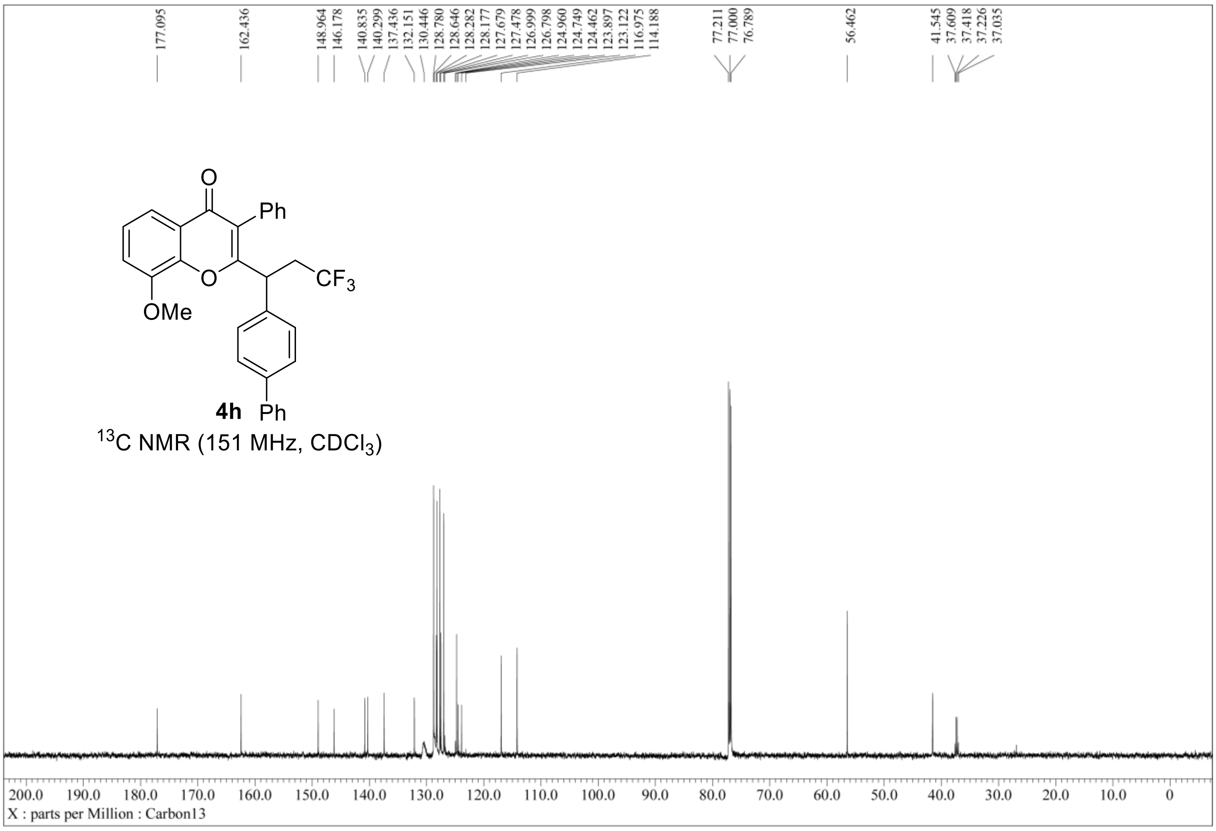
177.095
162.436
148.964
146.178
140.835
140.299
137.436
132.151
130.446
128.780
128.646
128.282
128.177
127.679
127.478
126.999
126.798
124.960
124.749
124.462
123.897
123.122
116.975
114.188
77.211
77.000
76.789
56.462
41.545
37.609
37.418
37.226
37.035
O
Ph
O
CF3
OMe
4h Ph
13C NMR (151 MHz, CDCl3)
X : parts per Million : Carbon13

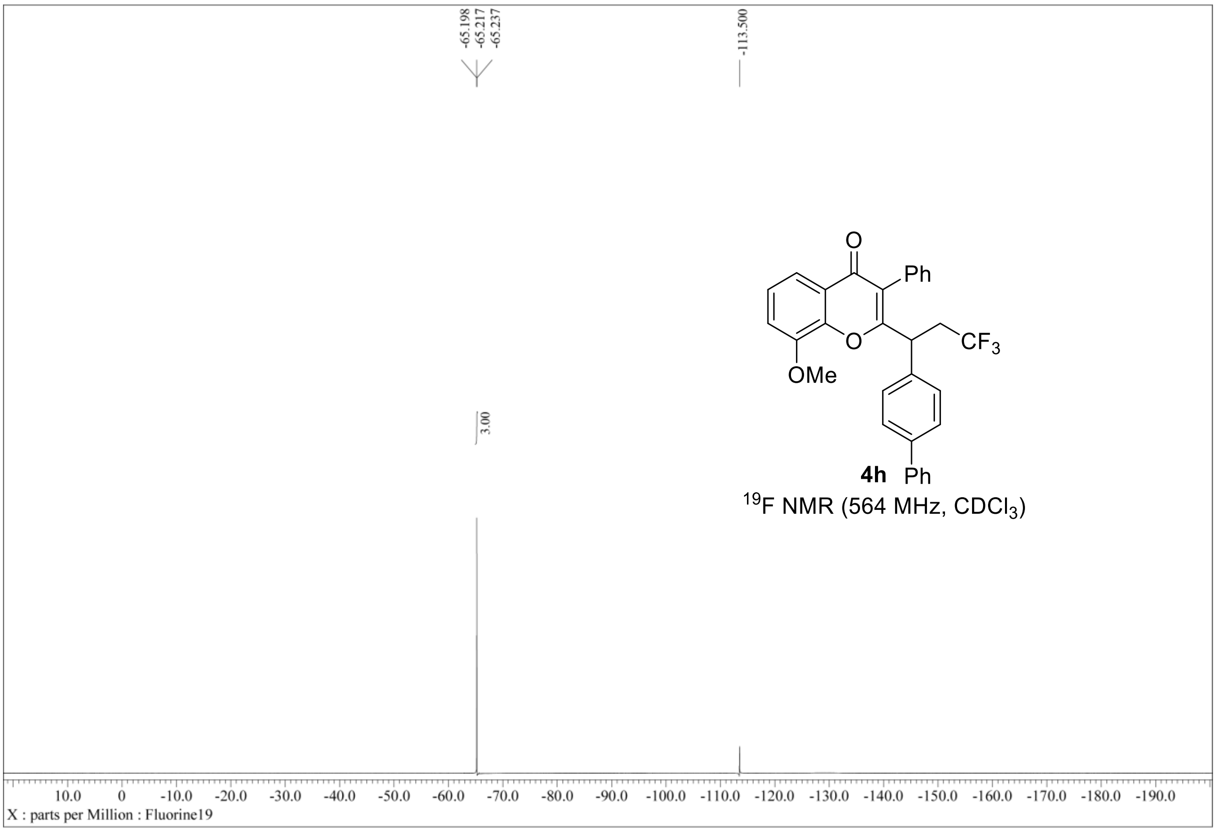
-65.198
-65.217
-65.237
-113.500
3.00
O
Ph
O
CF3
OMe
4h Ph
19F NMR (564 MHz, CDCl3)
X : parts per Million : Fluorine19

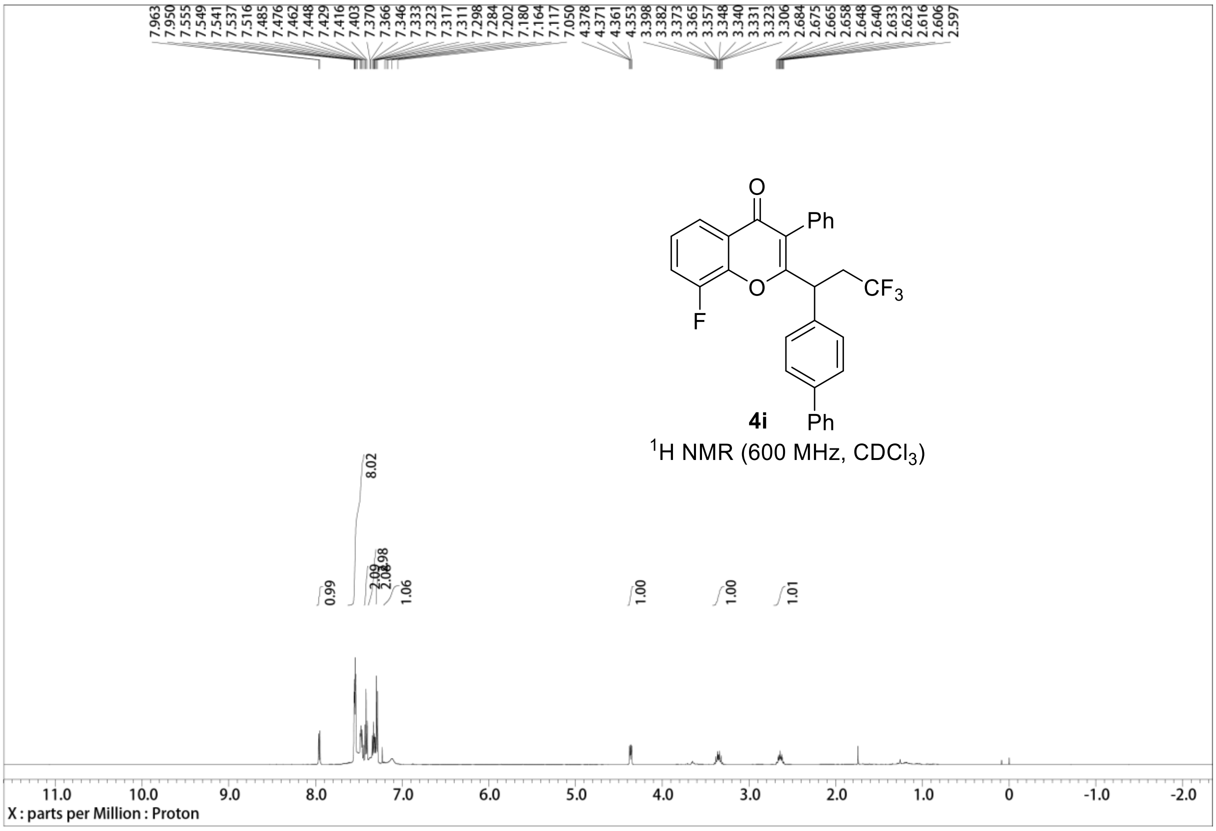

4i
^{1}H NMR (600 MHz, CDCl$_3$)
X : parts per Million : Proton

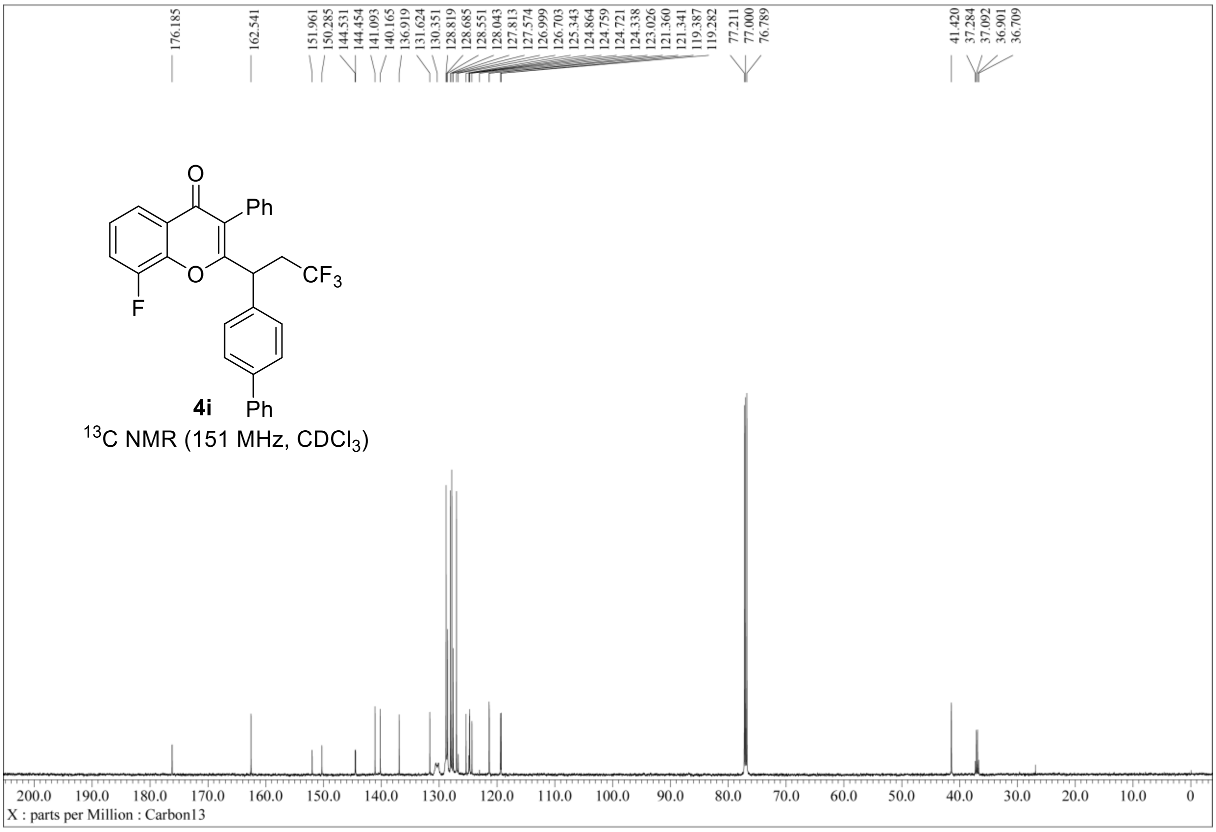

4i
^{13}C NMR (151 MHz, CDCl$_3$)
X : parts per Million : Carbon13

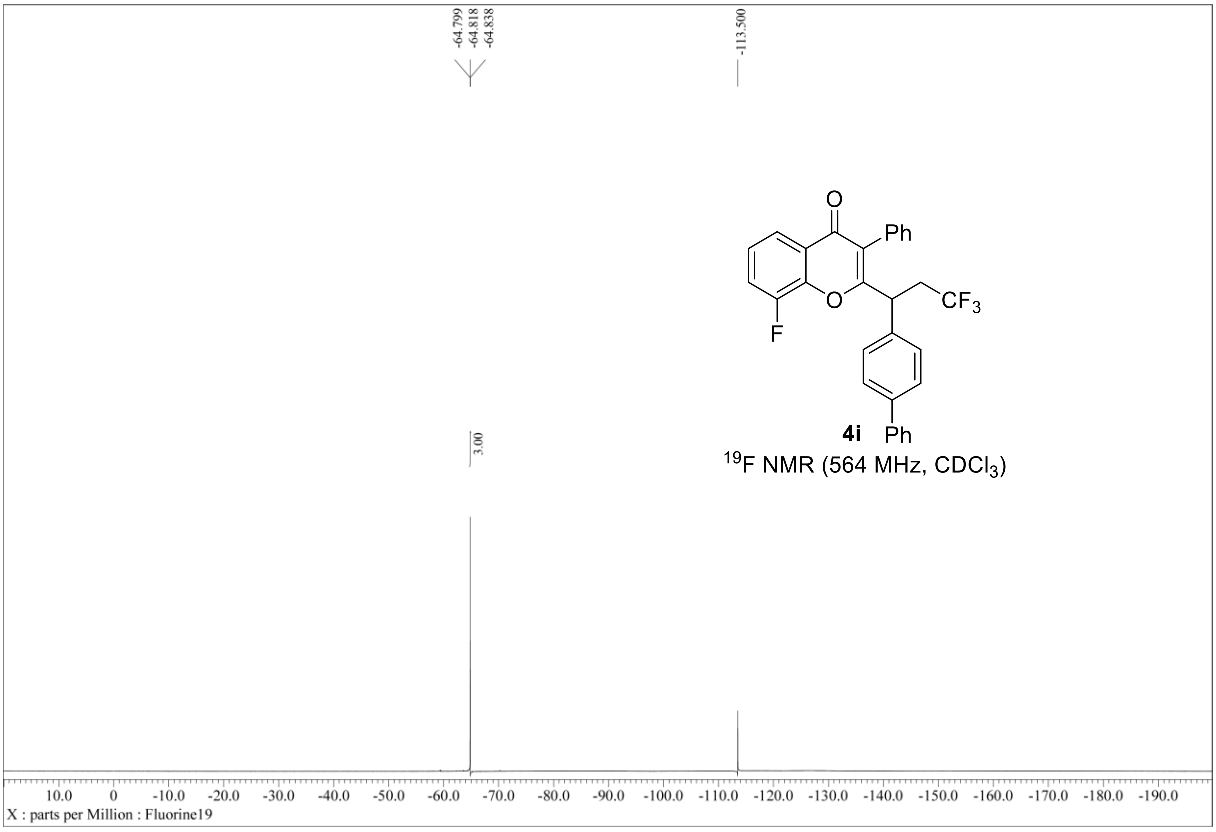
-64.799
-64.818
-64.838
-113.500
Ph
CF3
F
4i
Ph
19F NMR (564 MHz, CDCl3)
3.00
10.0 0 -10.0 -20.0 -30.0 -40.0 -50.0 -60.0 -70.0 -80.0 -90.0 -100.0 -110.0 -120.0 -130.0 -140.0 -150.0 -160.0 -170.0 -180.0 -190.0
X : parts per Million : Fluorine19

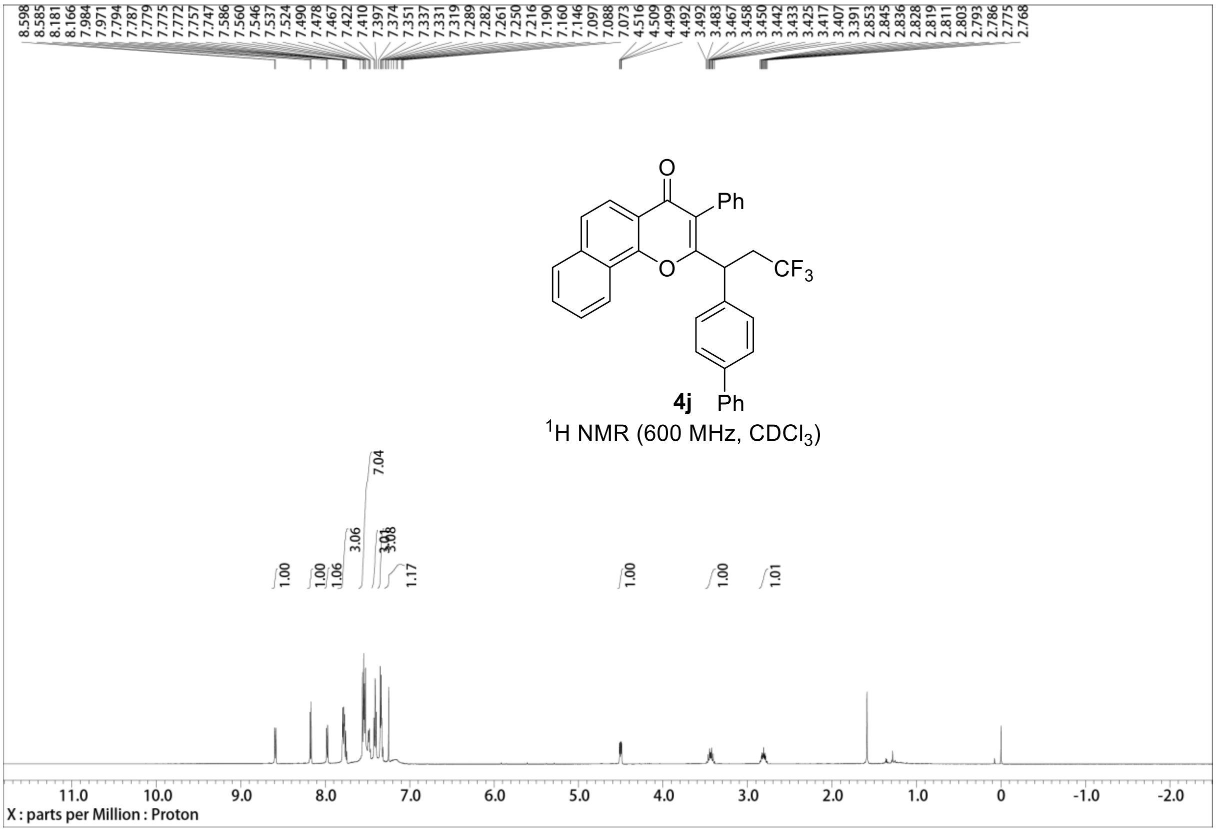
8.598 8.585 8.181 8.166 7.984 7.971 7.794 7.787 7.779 7.775 7.772 7.757 7.747 7.586 7.560 7.546 7.537 7.524 7.490 7.478 7.467 7.422 7.410 7.397 7.374 7.351 7.337 7.331 7.319 7.289 7.282 7.261 7.250 7.216 7.190 7.160 7.146 7.097 7.088 7.073 4.516 4.509 4.499 4.492 3.492 3.483 3.467 3.458 3.450 3.442 3.433 3.425 3.417 3.407 3.391 2.853 2.845 2.836 2.828 2.819 2.811 2.803 2.793 2.786 2.775 2.768
Ph
CF3
4j
Ph
1H NMR (600 MHz, CDCl3)
1.00
1.00
1.06
3.06
7.04
3.01
3.08
1.17
1.00
1.00
1.01
11.0 10.0 9.0 8.0 7.0 6.0 5.0 4.0 3.0 2.0 1.0 0 -1.0 -2.0
X : parts per Million : Proton

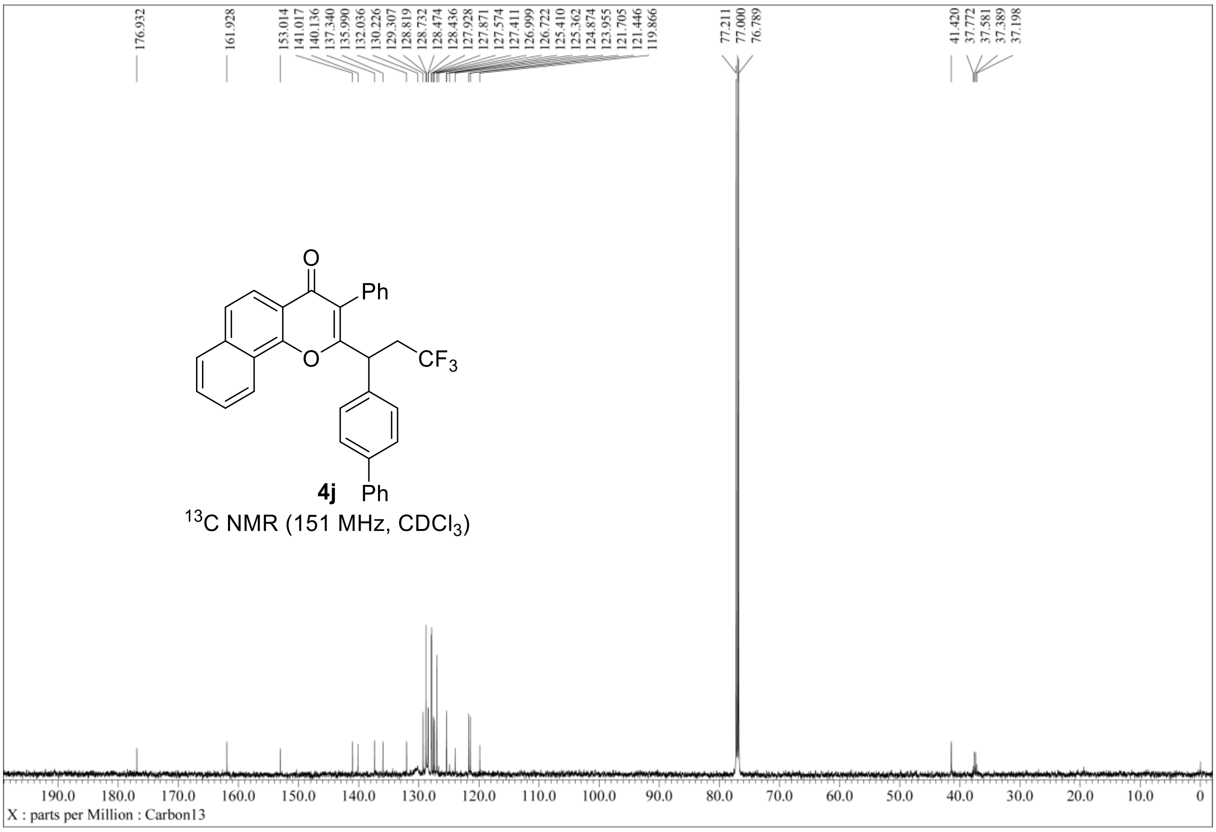
176.932
161.928
153.014 141.017 140.136 137.340 135.990 132.036 130.226 129.307 128.819 128.732 128.474 128.436 127.928 127.871 127.574 127.411 126.999 126.722 125.410 125.362 124.874 123.955 121.705 121.446 119.866
77.211 77.000 76.789
41.420 37.772 37.581 37.389 37.198
O
Ph
O
CF3
4j
Ph
13C NMR (151 MHz, CDCl3)
190.0 180.0 170.0 160.0 150.0 140.0 130.0 120.0 110.0 100.0 90.0 80.0 70.0 60.0 50.0 40.0 30.0 20.0 10.0 0
X : parts per Million : Carbon13

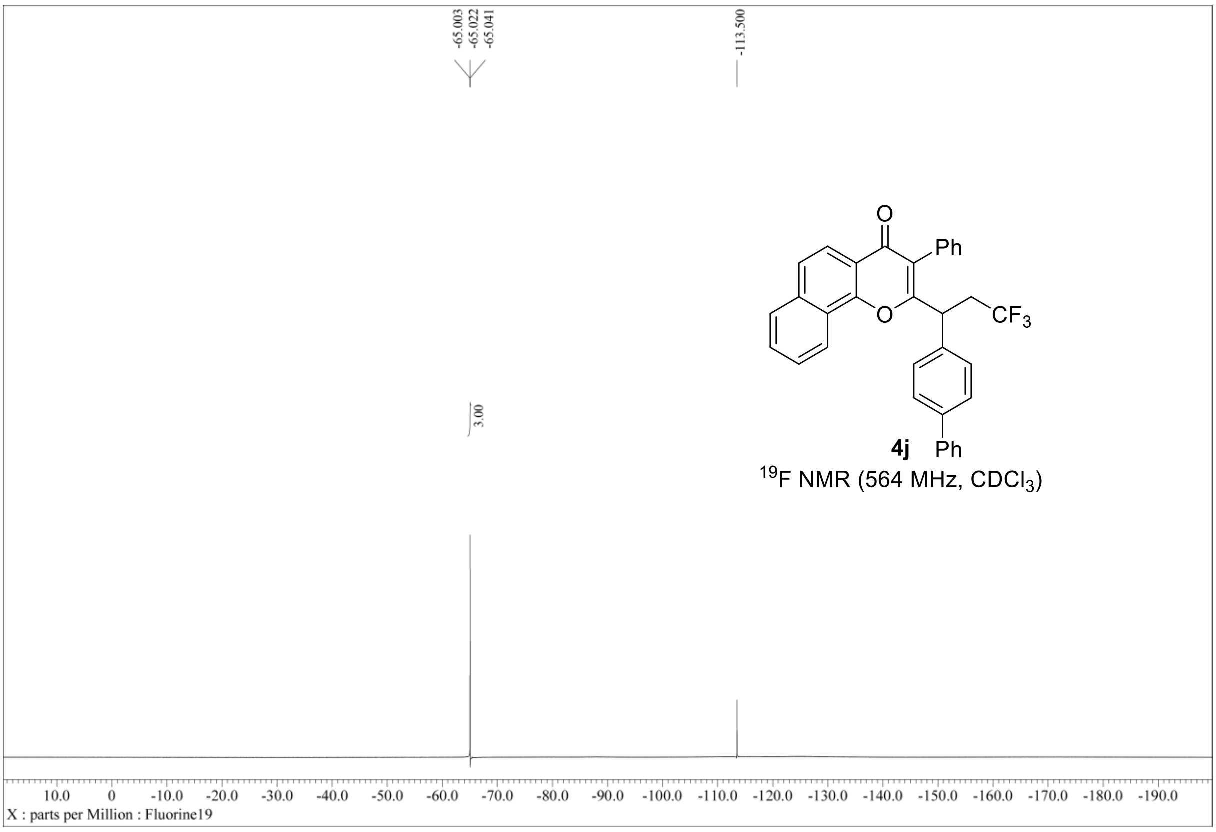
-65.003 -65.022 -65.041
-113.500
3.00
O
Ph
O
CF3
4j
Ph
19F NMR (564 MHz, CDCl3)
10.0 0 -10.0 -20.0 -30.0 -40.0 -50.0 -60.0 -70.0 -80.0 -90.0 -100.0 -110.0 -120.0 -130.0 -140.0 -150.0 -160.0 -170.0 -180.0 -190.0
X : parts per Million : Fluorine19

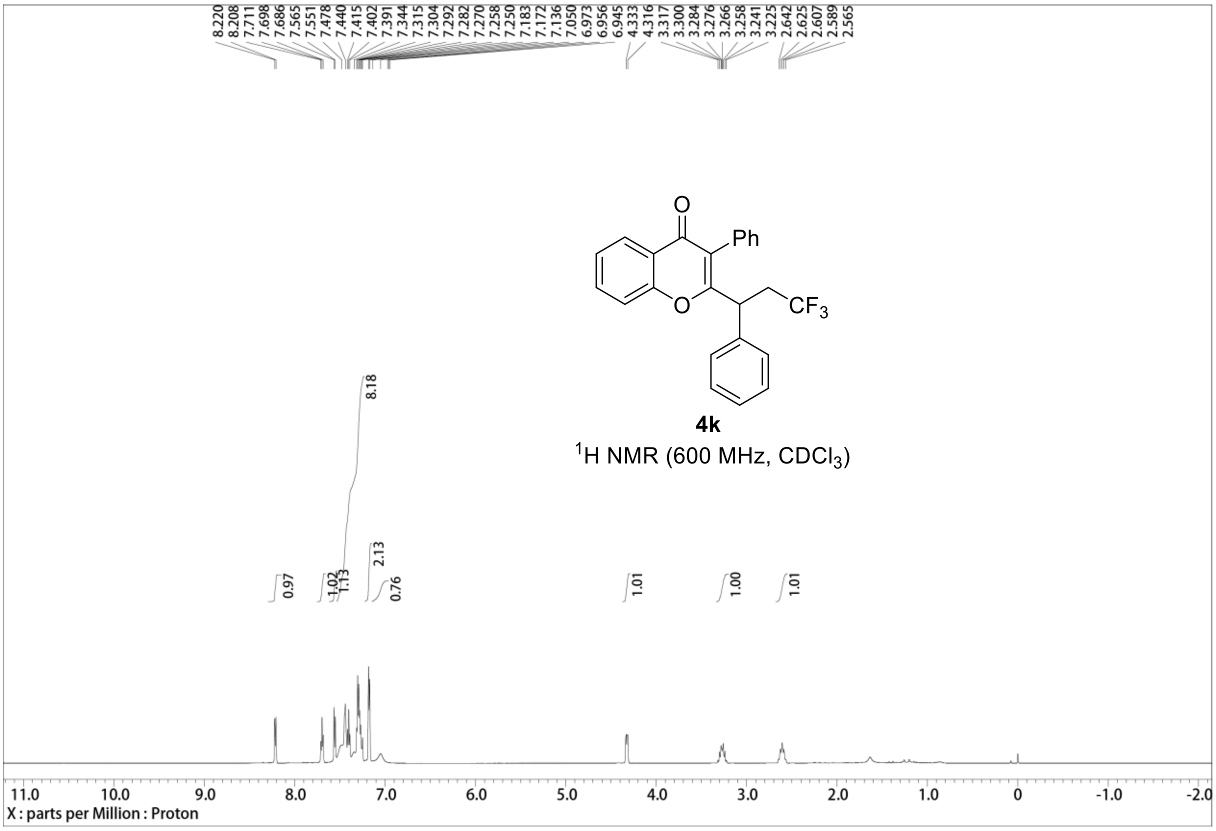
Ph
O
CF3
4k
1H NMR (600 MHz, CDCl3)
X : parts per Million : Proton

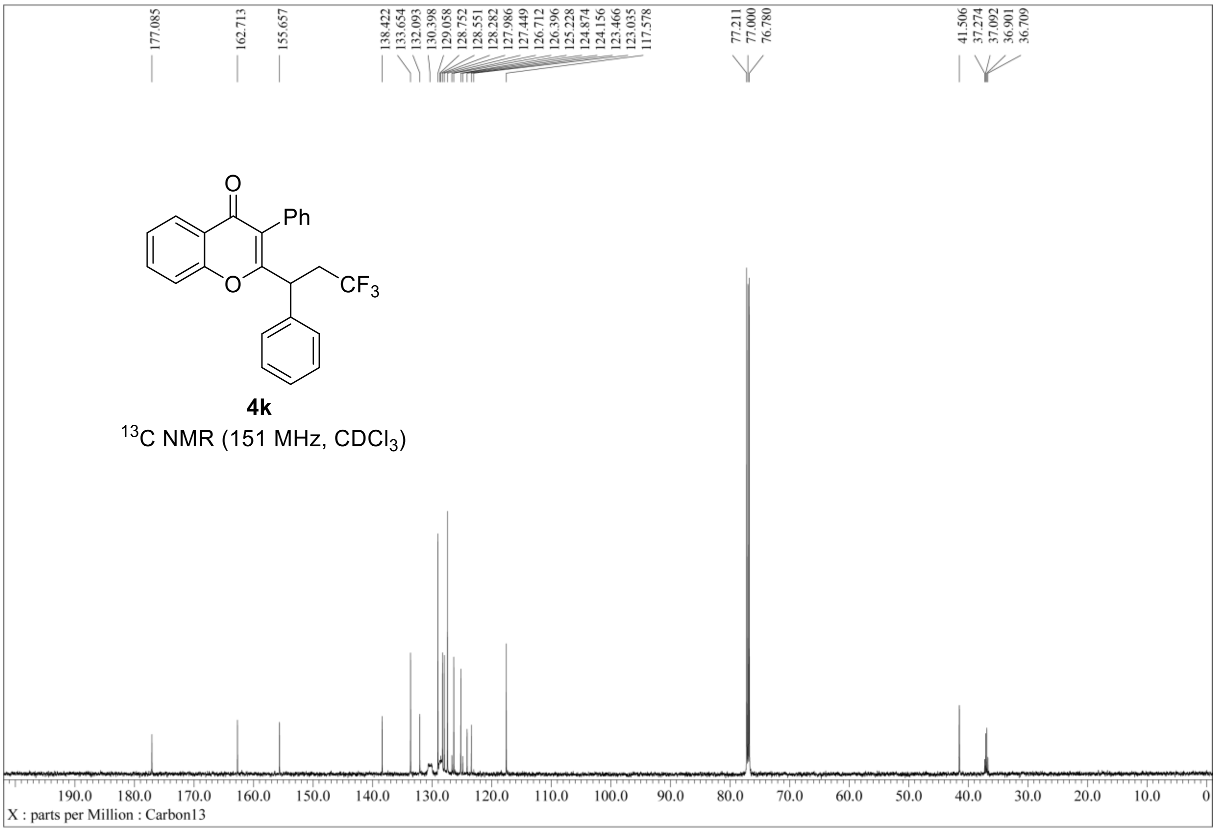
Ph
O
CF3
4k
13C NMR (151 MHz, CDCl3)
X : parts per Million : Carbon13

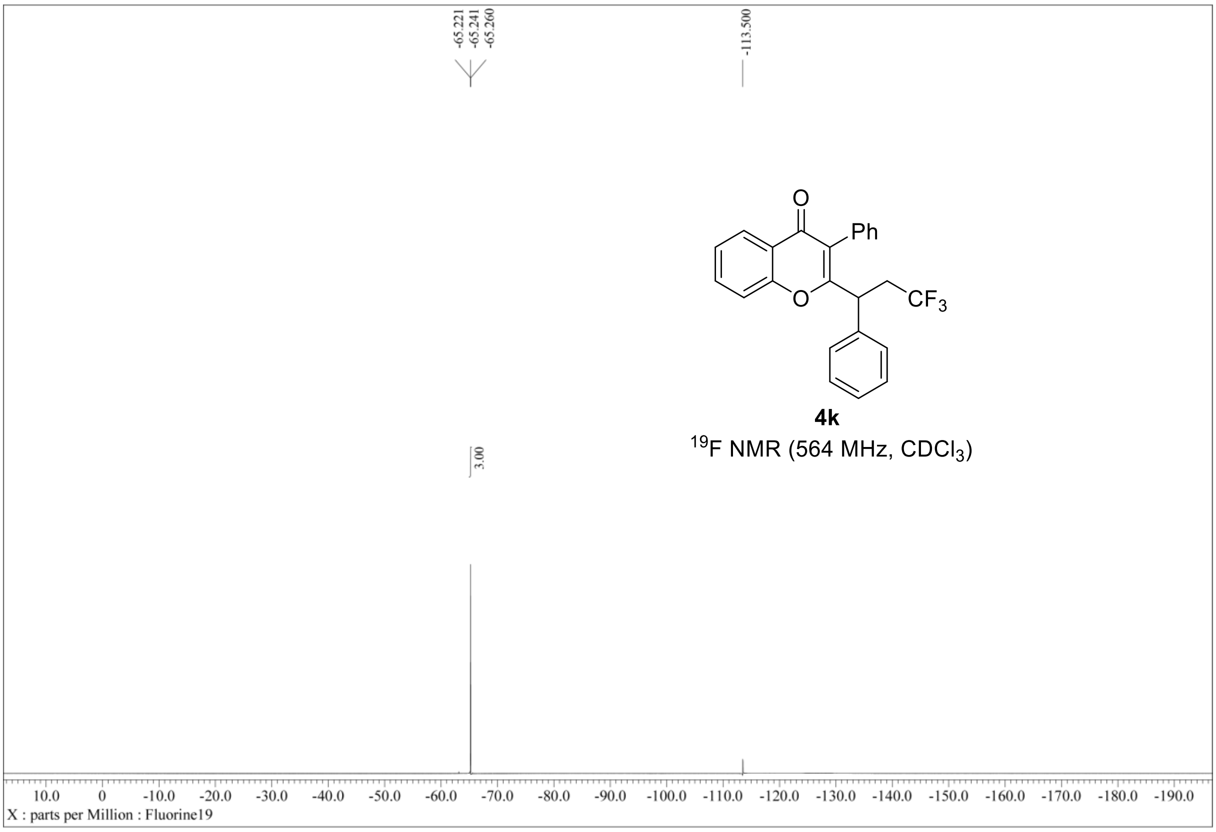
-65.221
-65.241
-65.260
-113.500
O
Ph
O
CF3
4k
19F NMR (564 MHz, CDCl3)
3.00
10.0 0 -10.0 -20.0 -30.0 -40.0 -50.0 -60.0 -70.0 -80.0 -90.0 -100.0 -110.0 -120.0 -130.0 -140.0 -150.0 -160.0 -170.0 -180.0 -190.0
X : parts per Million : Fluorine19

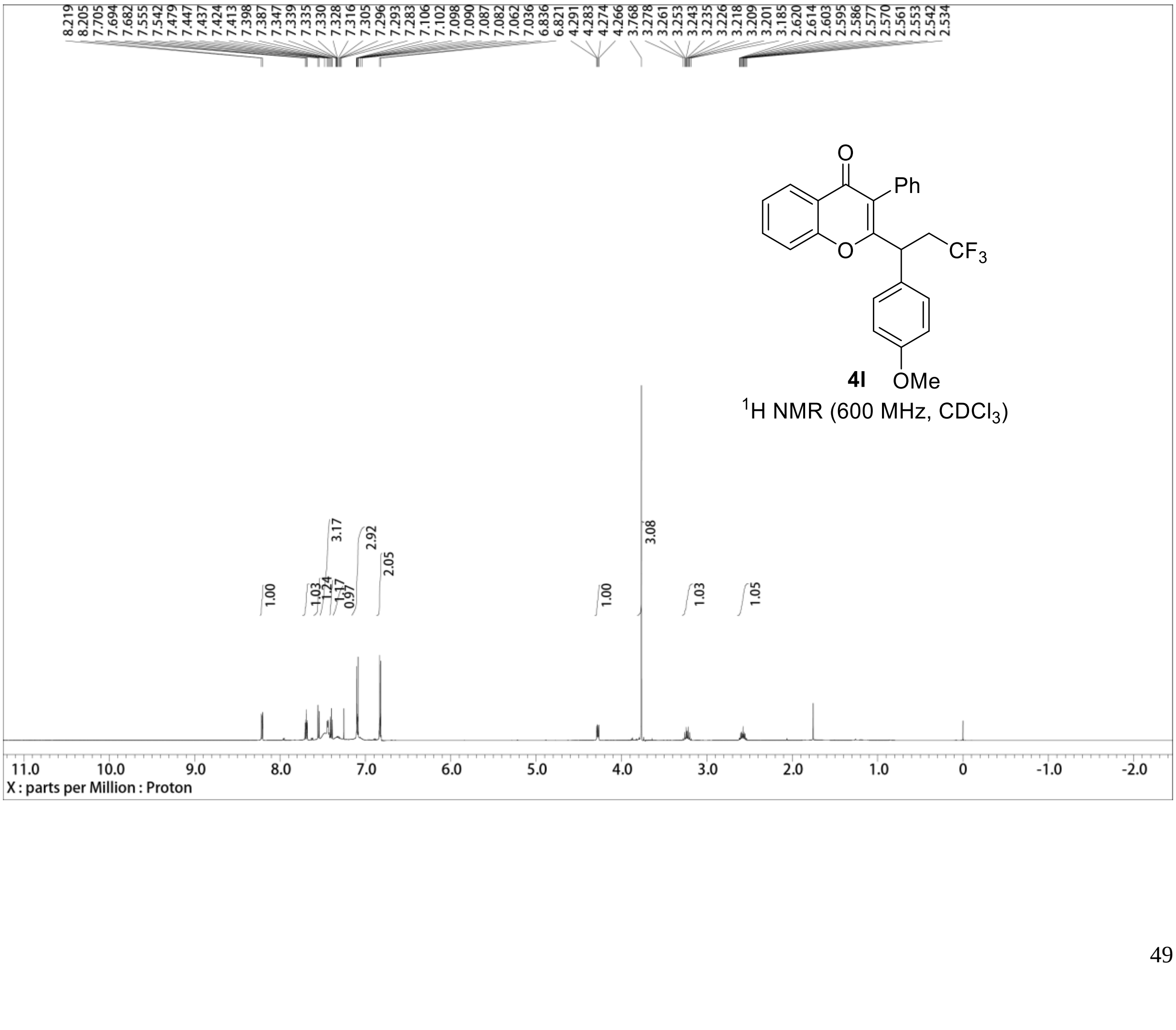
8.219
8.205
7.705
7.694
7.682
7.555
7.542
7.479
7.447
7.437
7.424
7.413
7.398
7.387
7.347
7.339
7.335
7.330
7.328
7.316
7.305
7.296
7.293
7.283
7.106
7.102
7.098
7.090
7.087
7.082
7.062
7.036
6.836
6.821
4.291
4.283
4.274
4.266
3.768
3.278
3.261
3.253
3.243
3.235
3.226
3.218
3.209
3.201
3.185
2.620
2.614
2.603
2.595
2.586
2.577
2.570
2.561
2.553
2.542
2.534
O
Ph
O
CF3
4l
OMe
1H NMR (600 MHz, CDCl3)
1.00
1.03
1.24
1.17
0.97
3.17
2.92
2.05
1.00
3.08
1.03
1.05
11.0 10.0 9.0 8.0 7.0 6.0 5.0 4.0 3.0 2.0 1.0 0 -1.0 -2.0
X : parts per Million : Proton

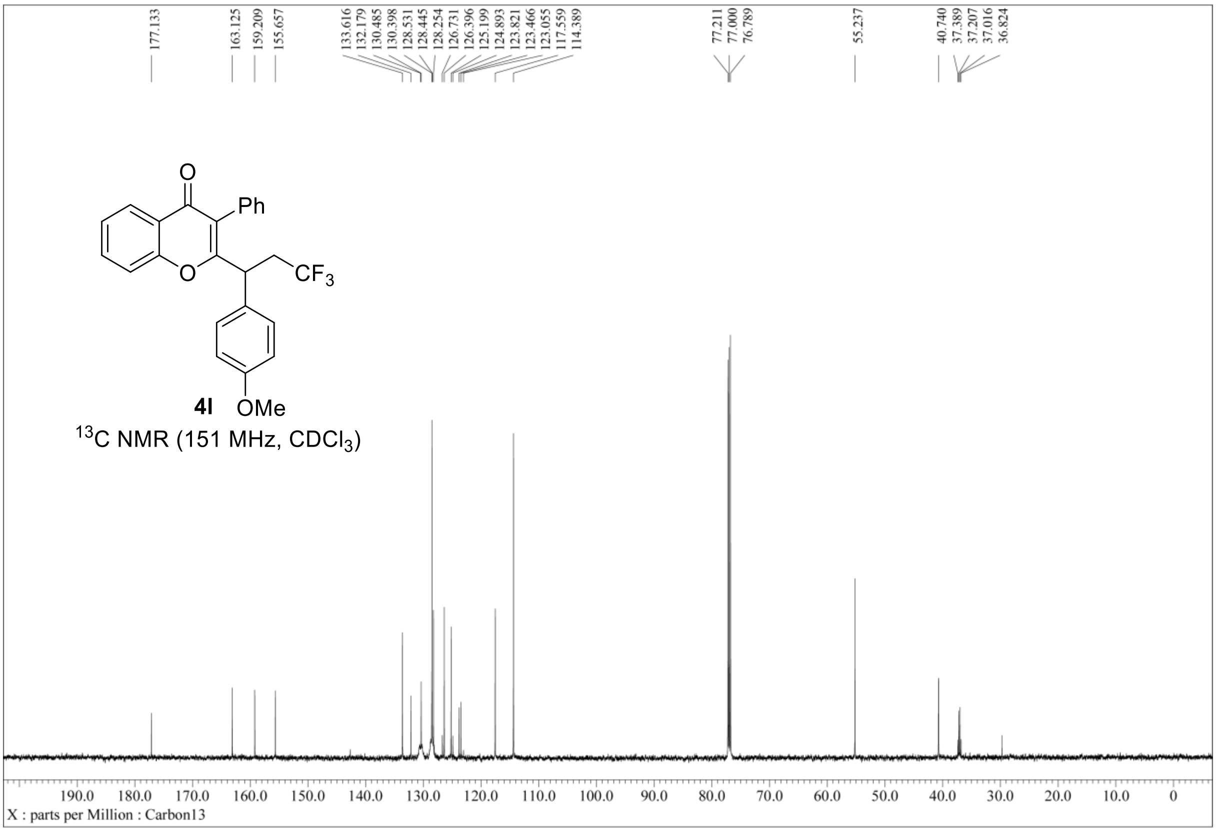
177.133
163.125
159.209
155.657
133.616
132.179
130.485
130.398
128.531
128.445
128.254
126.731
126.396
125.199
124.893
123.821
123.466
123.055
117.559
114.389
77.211
77.000
76.789
55.237
40.740
37.389
37.207
37.016
36.824
Ph
CF3
4l
OMe
13C NMR (151 MHz, CDCl3)
190.0 180.0 170.0 160.0 150.0 140.0 130.0 120.0 110.0 100.0 90.0 80.0 70.0 60.0 50.0 40.0 30.0 20.0 10.0 0
X : parts per Million : Carbon13

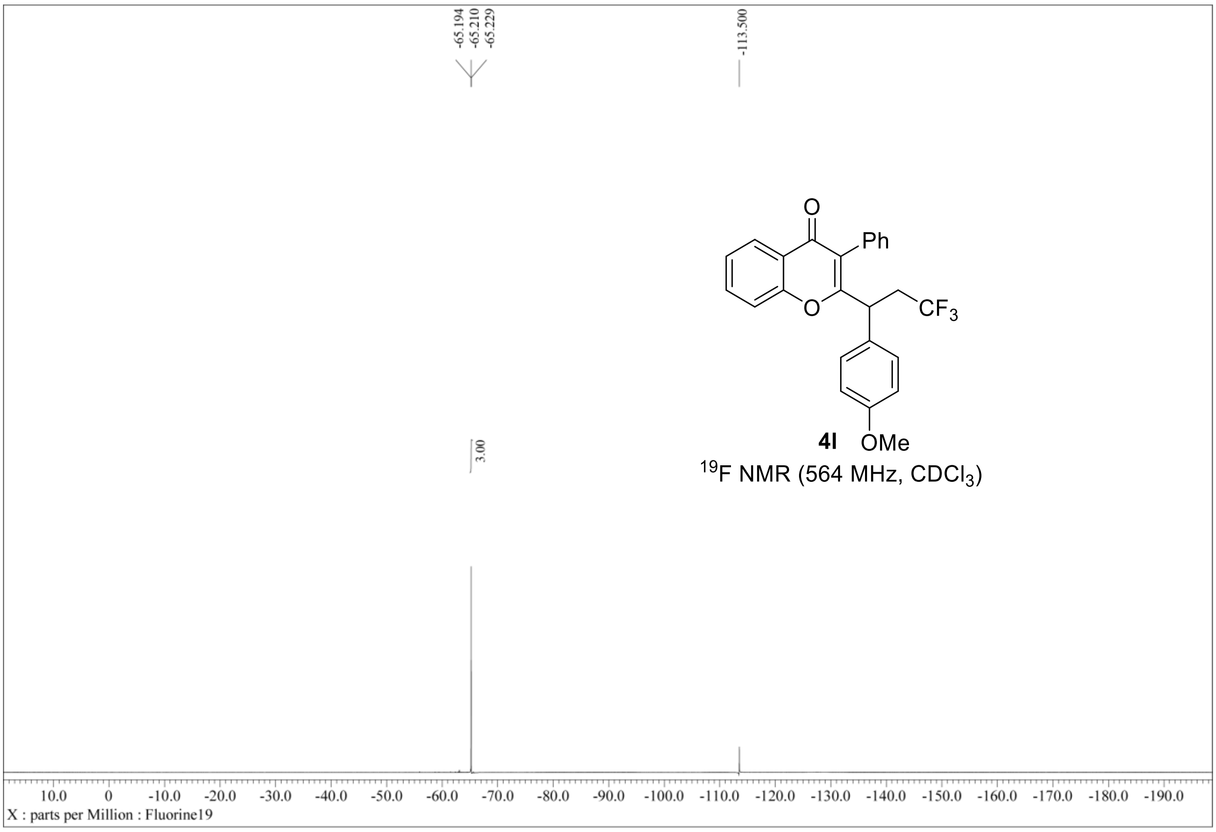
-65.194
-65.210
-65.229
-113.500
Ph
CF3
4l
OMe
19F NMR (564 MHz, CDCl3)
3.00
10.0 0 -10.0 -20.0 -30.0 -40.0 -50.0 -60.0 -70.0 -80.0 -90.0 -100.0 -110.0 -120.0 -130.0 -140.0 -150.0 -160.0 -170.0 -180.0 -190.0
X : parts per Million : Fluorine19

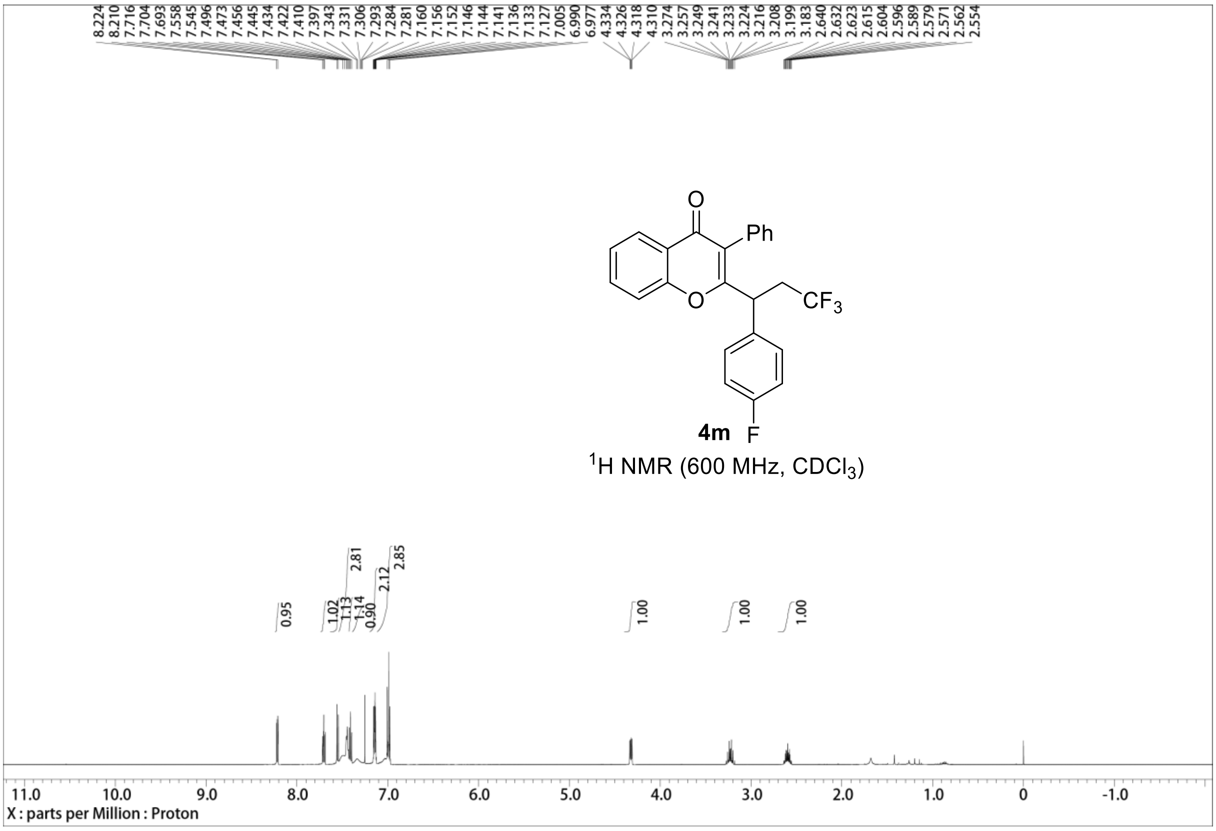

Ph
CF3
4m
F
1H NMR (600 MHz, CDCl3)
X : parts per Million : Proton

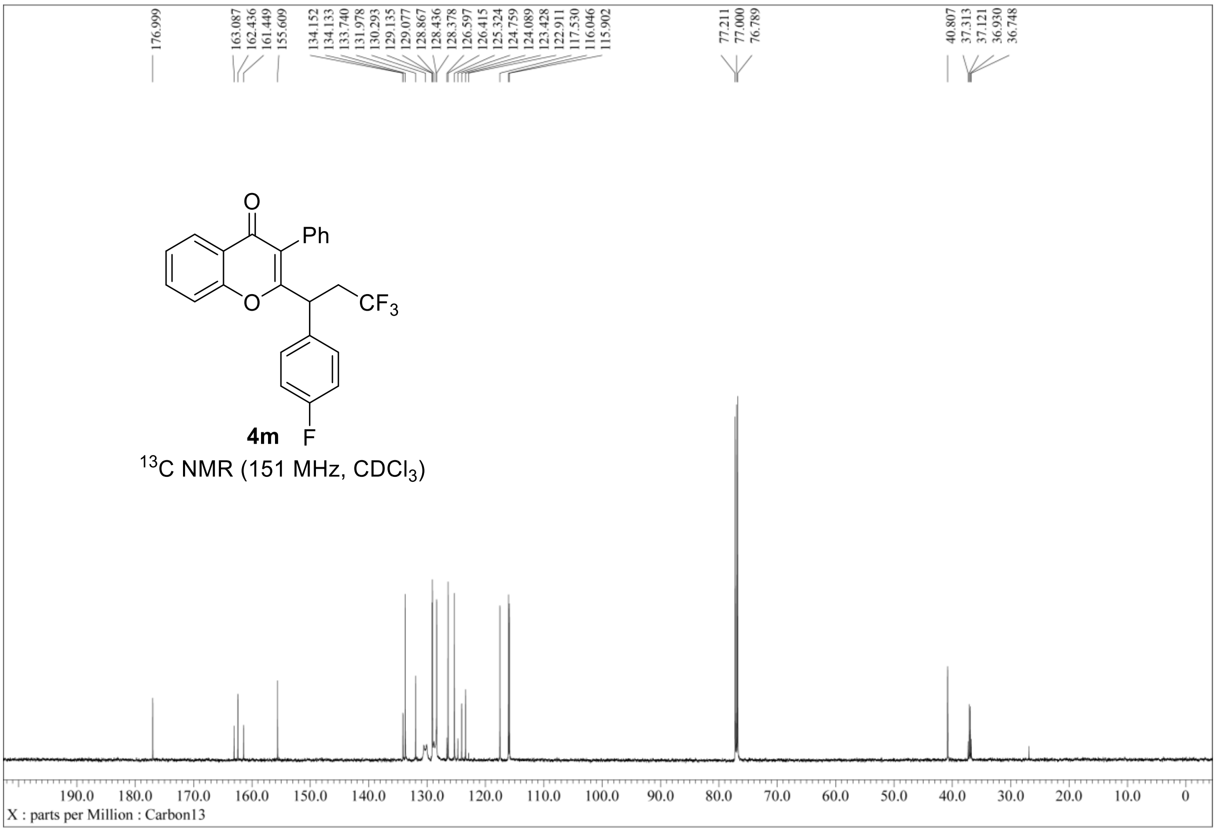

Ph
CF3
4m
F
13C NMR (151 MHz, CDCl3)
X : parts per Million : Carbon13

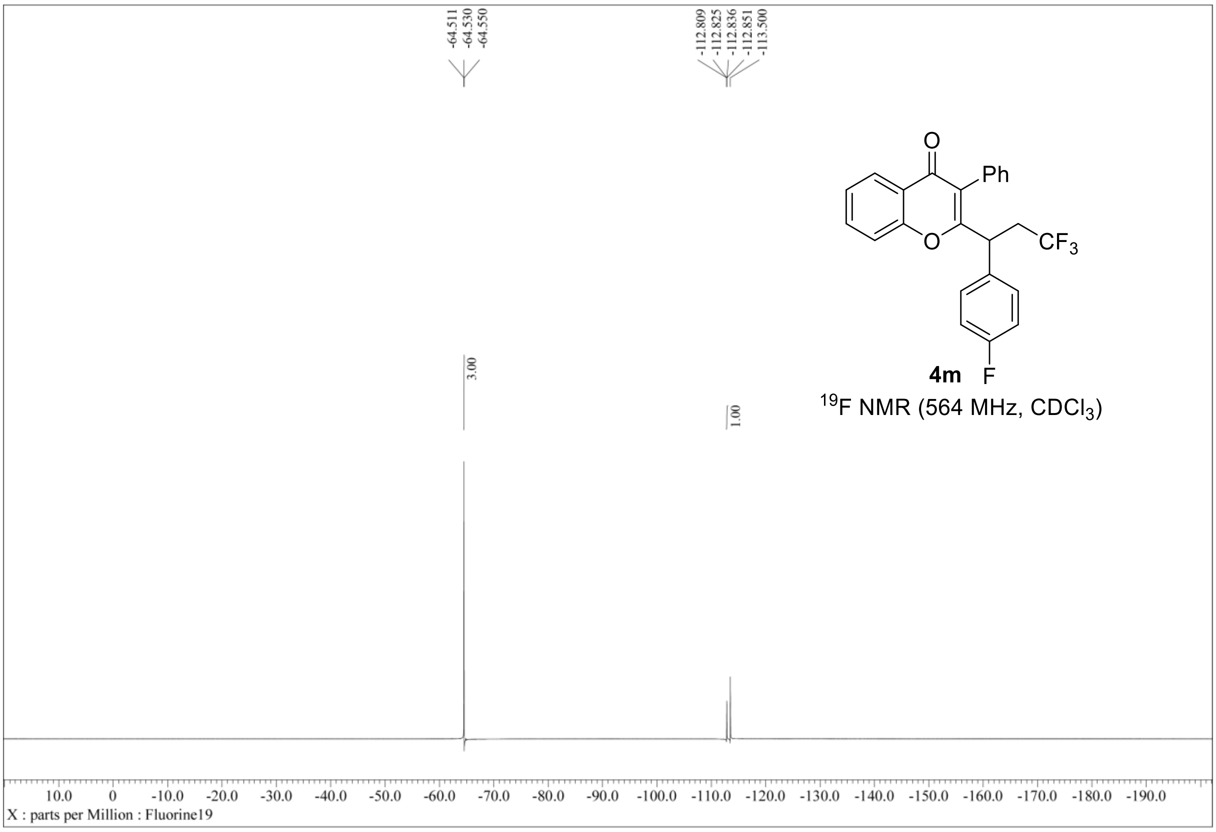
-64.511
-64.530
-64.550
-112.809
-112.825
-112.836
-112.851
-113.500
3.00
1.00
O
Ph
O
CF3
F
4m
19F NMR (564 MHz, CDCl3)
10.0 0 -10.0 -20.0 -30.0 -40.0 -50.0 -60.0 -70.0 -80.0 -90.0 -100.0 -110.0 -120.0 -130.0 -140.0 -150.0 -160.0 -170.0 -180.0 -190.0
X : parts per Million : Fluorine19

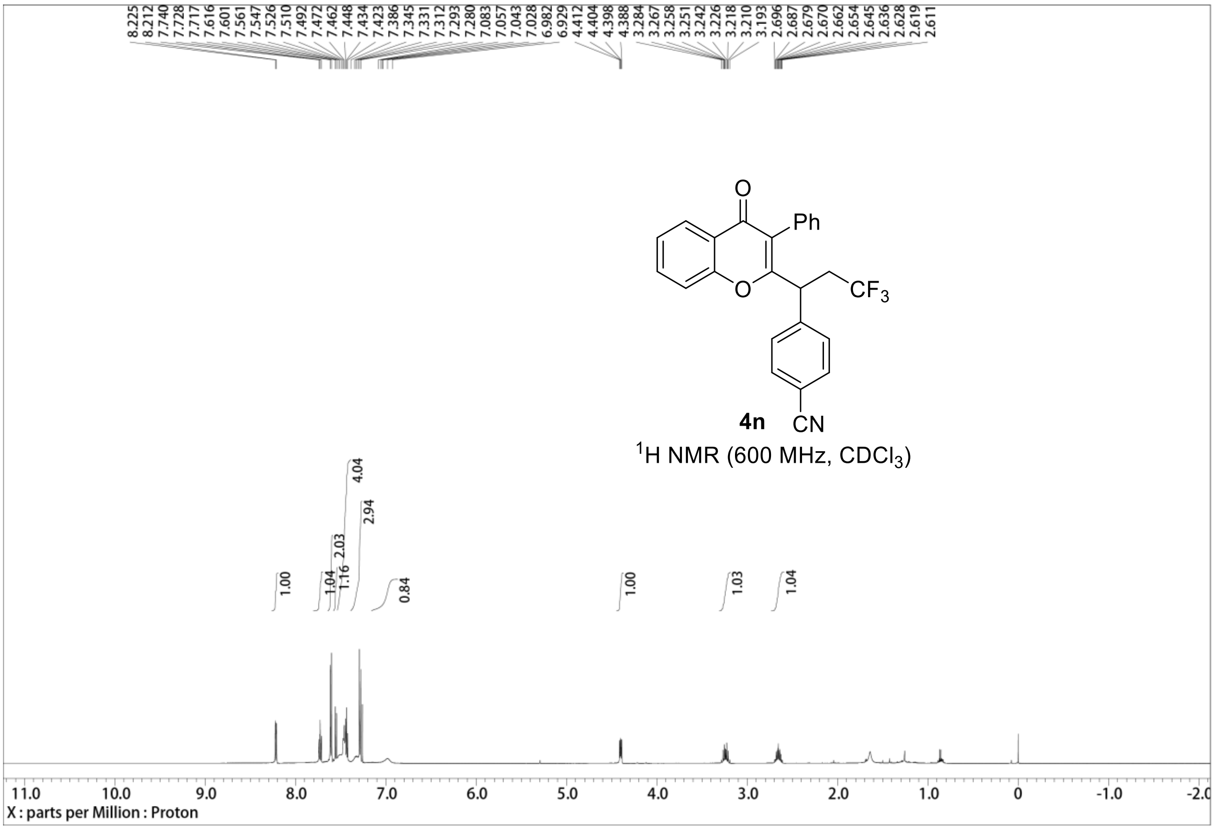
8.225 8.212 7.740 7.728 7.717 7.616 7.601 7.561 7.547 7.526 7.510 7.492 7.472 7.462 7.448 7.434 7.423 7.386 7.345 7.331 7.312 7.293 7.280 7.083 7.057 7.043 7.028 6.982 6.929 4.412 4.404 4.398 4.388 3.284 3.267 3.258 3.251 3.242 3.226 3.218 3.210 3.193 2.696 2.687 2.679 2.670 2.662 2.654 2.645 2.636 2.628 2.619 2.611
O
Ph
O
CF3
CN
4n
1H NMR (600 MHz, CDCl3)
1.00
1.04
1.16
2.03
4.04
2.94
0.84
1.00
1.03
1.04
11.0 10.0 9.0 8.0 7.0 6.0 5.0 4.0 3.0 2.0 1.0 0 -1.0 -2.0
X : parts per Million : Proton

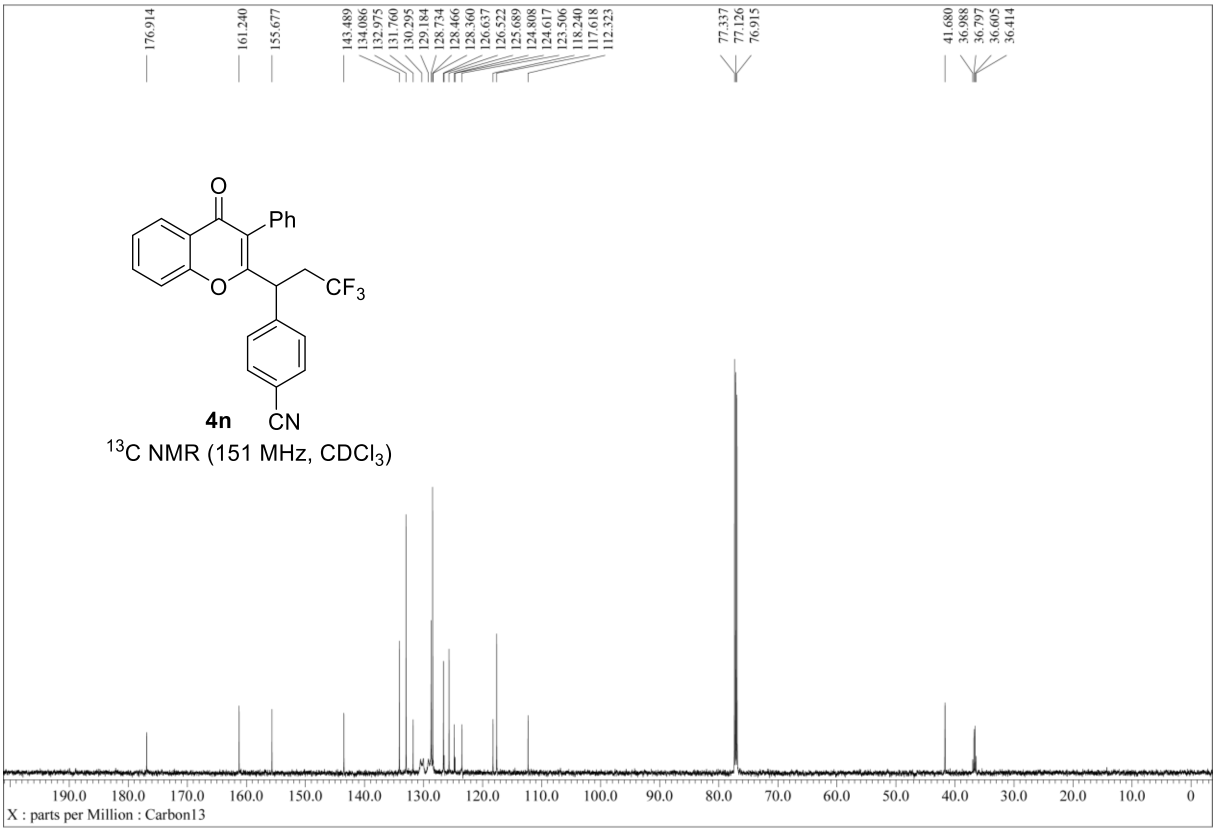
176.914
161.240
155.677
143.489
134.086
132.975
131.760
130.295
129.184
128.734
128.466
128.360
126.637
126.522
125.689
124.808
124.617
123.506
118.240
117.618
112.323
77.337
77.126
76.915
41.680
36.988
36.797
36.605
36.414
O
Ph
O
CF3
4n
CN
13C NMR (151 MHz, CDCl3)
190.0 180.0 170.0 160.0 150.0 140.0 130.0 120.0 110.0 100.0 90.0 80.0 70.0 60.0 50.0 40.0 30.0 20.0 10.0 0
X : parts per Million : Carbon13

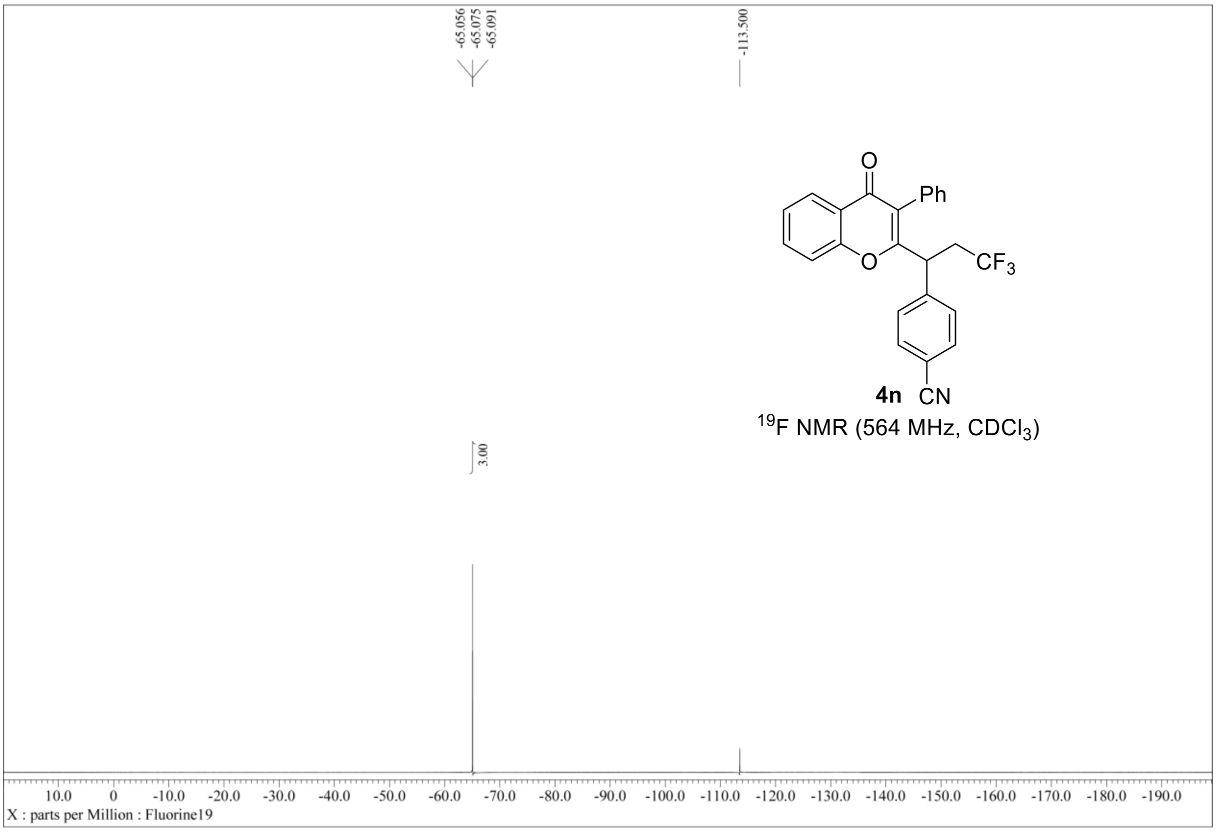
-65.056
-65.075
-65.091
-113.500
O
Ph
O
CF3
4n
CN
19F NMR (564 MHz, CDCl3)
3.00
10.0 0 -10.0 -20.0 -30.0 -40.0 -50.0 -60.0 -70.0 -80.0 -90.0 -100.0 -110.0 -120.0 -130.0 -140.0 -150.0 -160.0 -170.0 -180.0 -190.0
X : parts per Million : Fluorine19

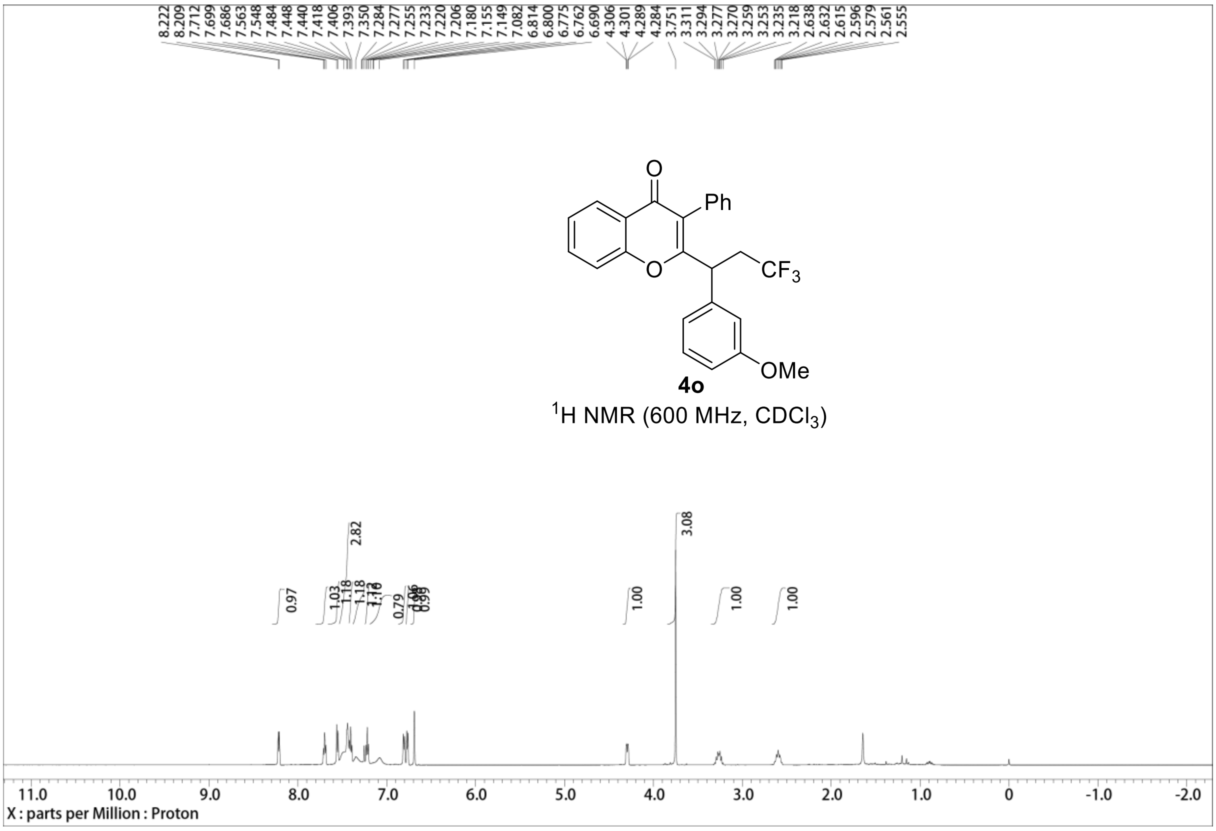

4o
^{1}H NMR (600 MHz, CDCl$_3$)
X : parts per Million : Proton

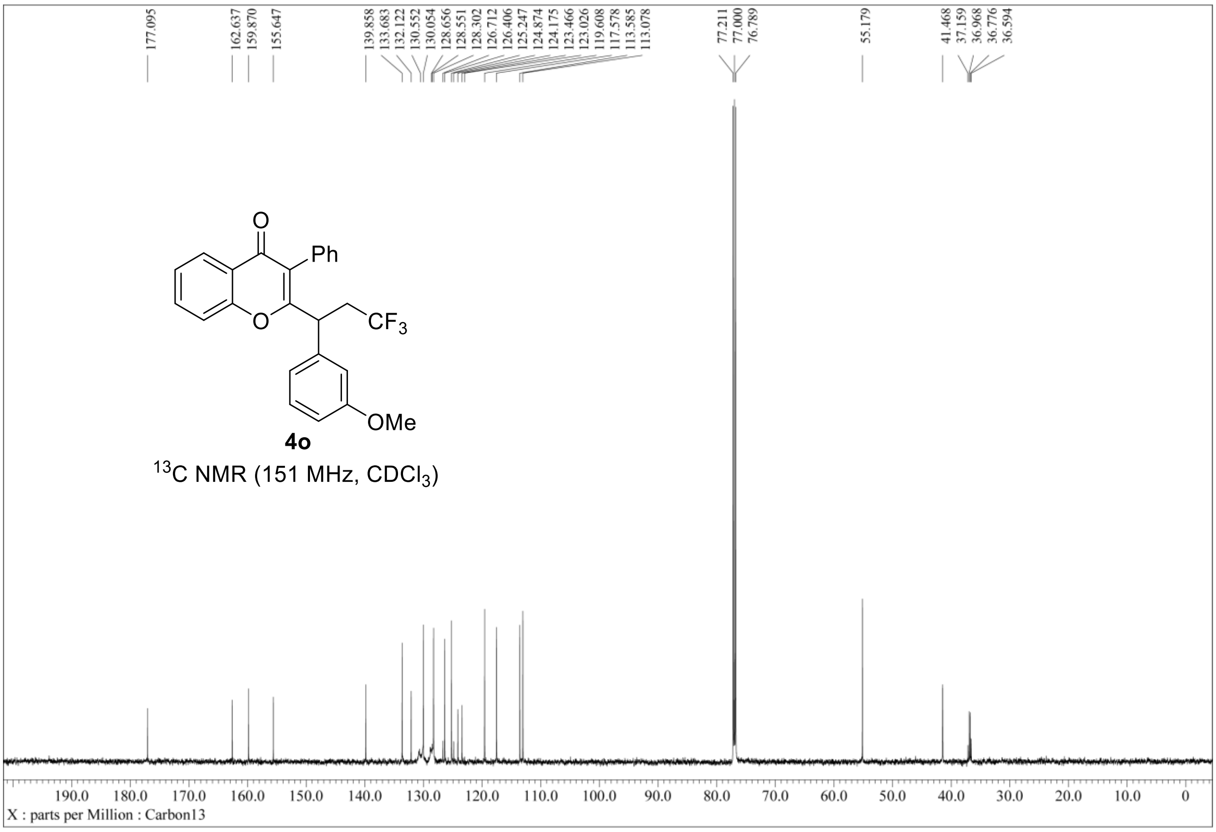

4o
^{13}C NMR (151 MHz, CDCl$_3$)
X : parts per Million : Carbon13

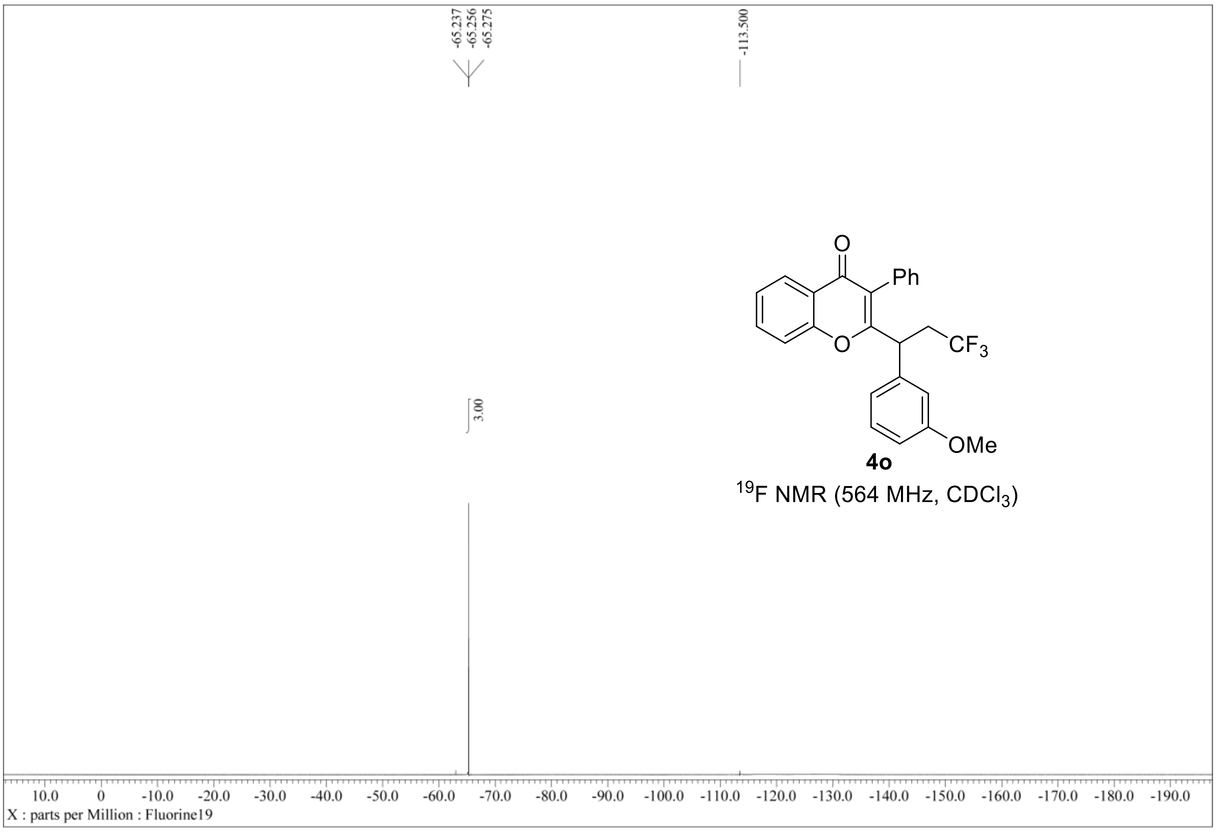
O
Ph
O
CF3
OMe
4o
19F NMR (564 MHz, CDCl3)
-65.237
-65.256
-65.275
-113.500
3.00
X : parts per Million : Fluorine19

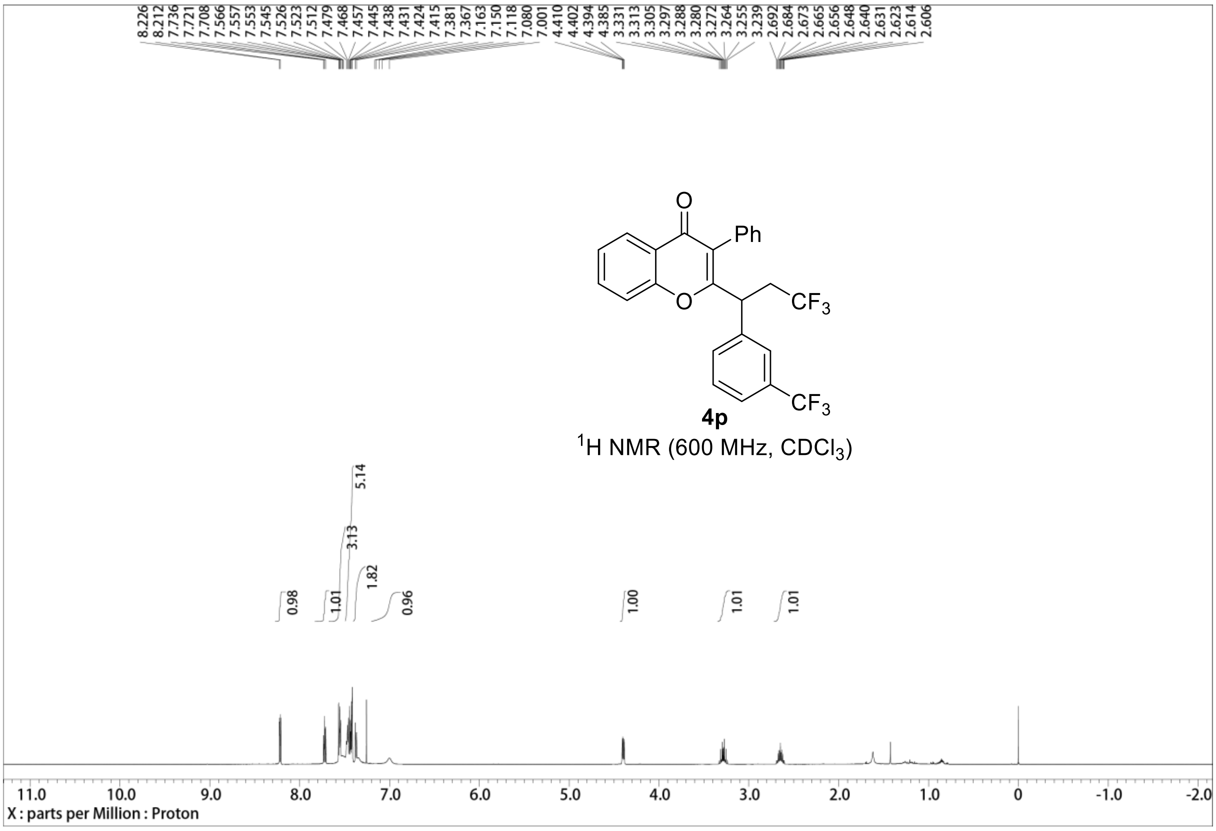
O
Ph
O
CF3
CF3
4p
1H NMR (600 MHz, CDCl3)
0.98
1.01
3.13
5.14
1.82
0.96
1.00
1.01
1.01
X : parts per Million : Proton

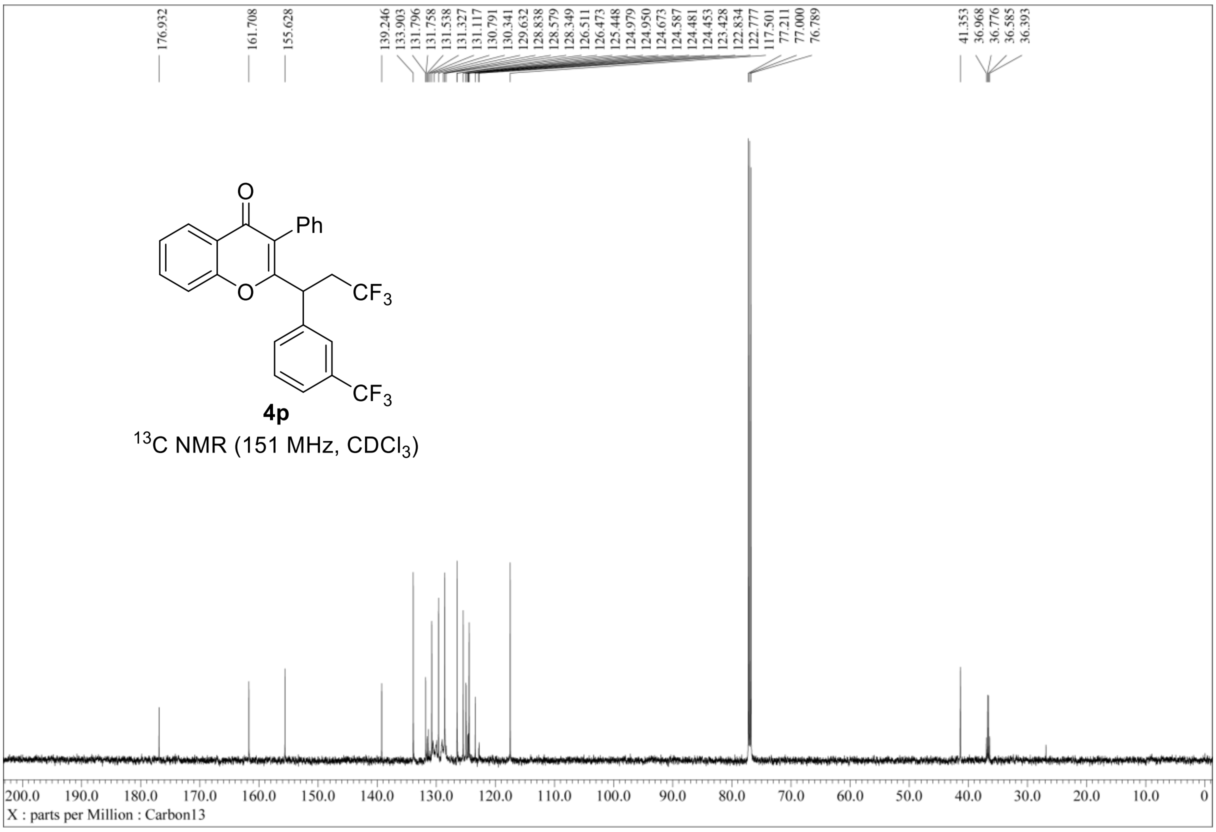
176.932
161.708
155.628
139.246
133.903
131.796
131.758
131.538
131.327
131.117
130.791
130.341
129.632
128.838
128.579
128.349
126.511
126.473
125.448
124.979
124.950
124.673
124.587
124.481
124.453
123.428
122.834
122.777
117.501
77.211
77.000
76.789
41.353
36.968
36.776
36.585
36.393
O
Ph
O
CF3
CF3
4p
13C NMR (151 MHz, CDCl3)
200.0 190.0 180.0 170.0 160.0 150.0 140.0 130.0 120.0 110.0 100.0 90.0 80.0 70.0 60.0 50.0 40.0 30.0 20.0 10.0 0
X : parts per Million : Carbon13

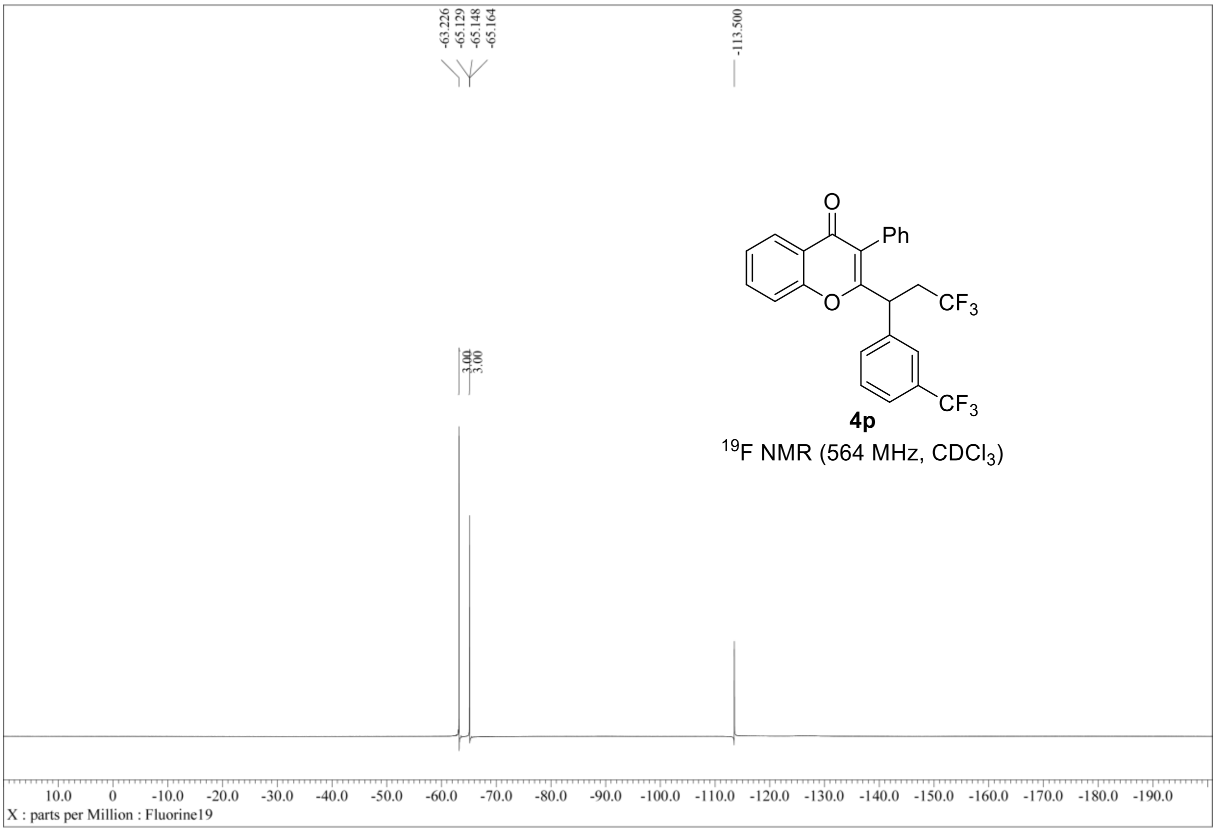
-63.226
-65.129
-65.148
-65.164
-113.500
O
Ph
O
CF3
CF3
4p
19F NMR (564 MHz, CDCl3)
3.00
3.00
10.0 0 -10.0 -20.0 -30.0 -40.0 -50.0 -60.0 -70.0 -80.0 -90.0 -100.0 -110.0 -120.0 -130.0 -140.0 -150.0 -160.0 -170.0 -180.0 -190.0
X : parts per Million : Fluorine19

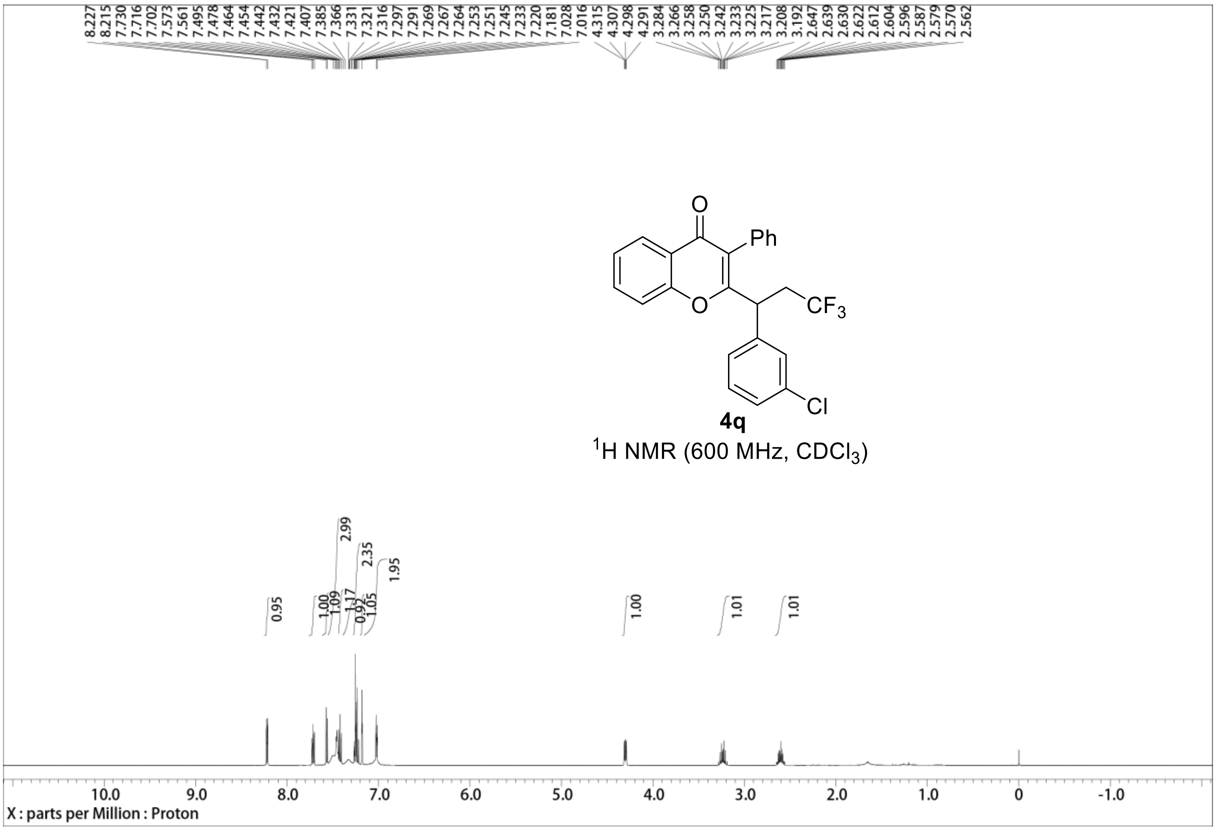
8.227 8.215 7.730 7.716 7.702 7.573 7.561 7.495 7.478 7.464 7.454 7.442 7.432 7.421 7.407 7.385 7.366 7.331 7.321 7.316 7.297 7.291 7.269 7.267 7.264 7.253 7.251 7.245 7.233 7.220 7.181 7.028 7.016 4.315 4.307 4.298 4.291 3.284 3.266 3.258 3.250 3.242 3.233 3.225 3.217 3.208 3.192 2.647 2.639 2.630 2.622 2.612 2.604 2.596 2.587 2.579 2.570 2.562
O
Ph
O
CF3
Cl
4q
1H NMR (600 MHz, CDCl3)
0.95 1.00 1.09 1.17 0.92 1.05 2.99 2.35 1.95 1.00 1.01 1.01
10.0 9.0 8.0 7.0 6.0 5.0 4.0 3.0 2.0 1.0 0 -1.0
X : parts per Million : Proton

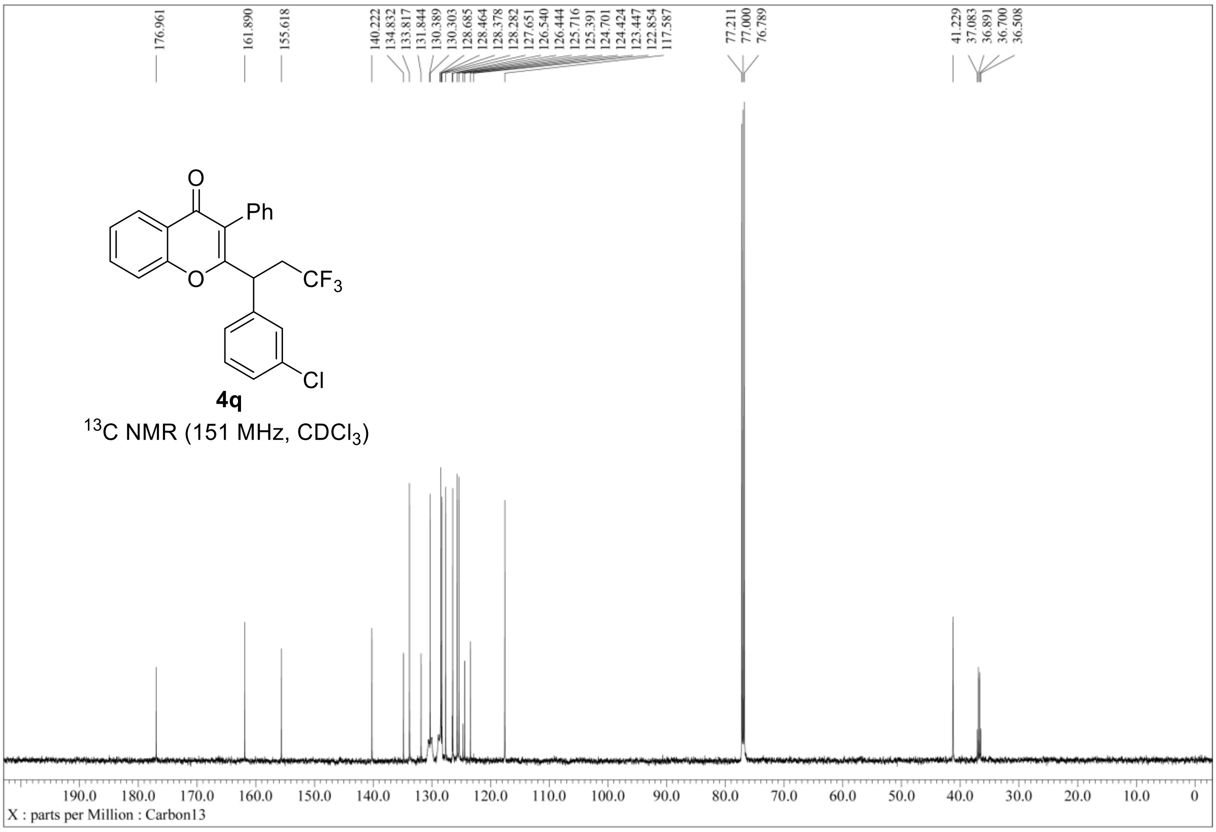
176.961 161.890 155.618 140.222 134.832 133.817 131.844 130.389 130.303 128.685 128.464 128.378 128.282 127.651 126.540 126.444 125.716 125.391 124.701 124.424 123.447 122.854 117.587 77.211 77.000 76.789 41.229 37.083 36.891 36.700 36.508
O
Ph
O
CF3
Cl
4q
13C NMR (151 MHz, CDCl3)
190.0 180.0 170.0 160.0 150.0 140.0 130.0 120.0 110.0 100.0 90.0 80.0 70.0 60.0 50.0 40.0 30.0 20.0 10.0 0
X : parts per Million : Carbon13

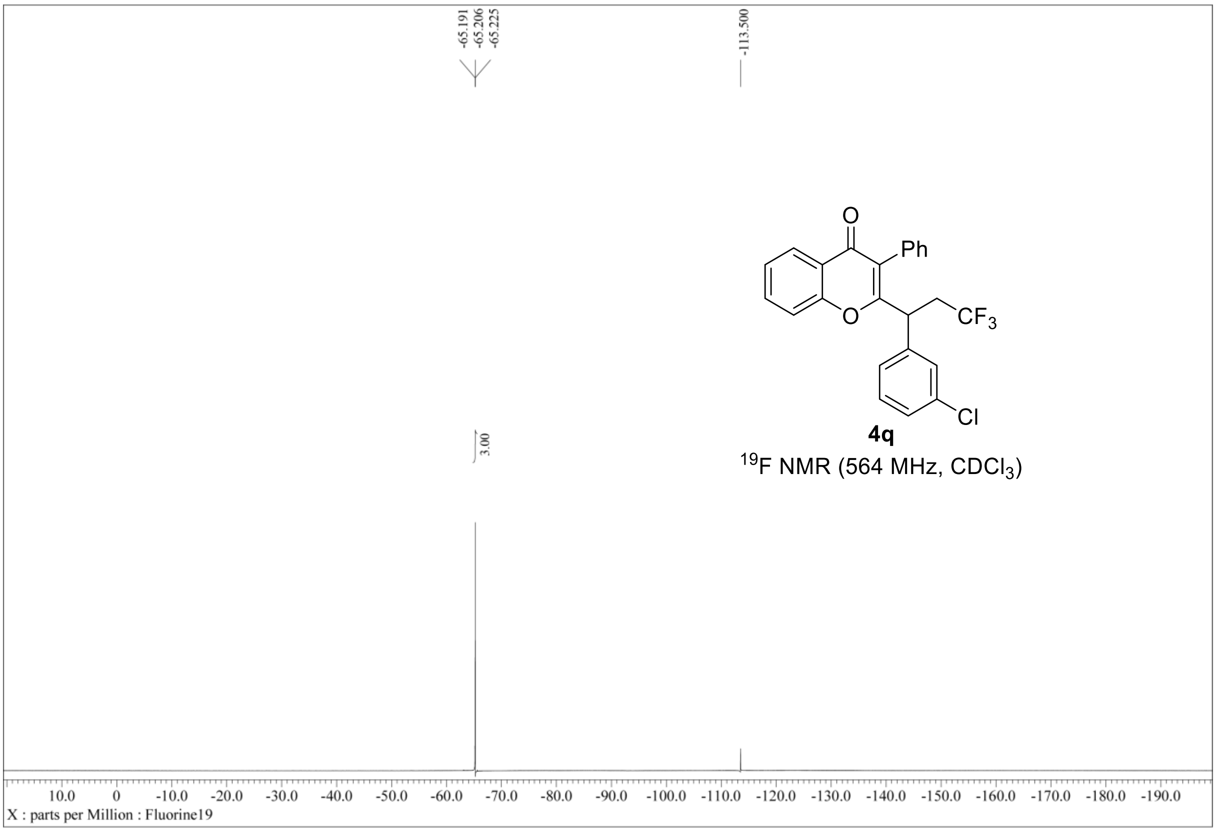

-65.191
-65.206
-65.225
-113.500
O
Ph
O
CF3
Cl
4q
19F NMR (564 MHz, CDCl3)
3.00
X : parts per Million : Fluorine19

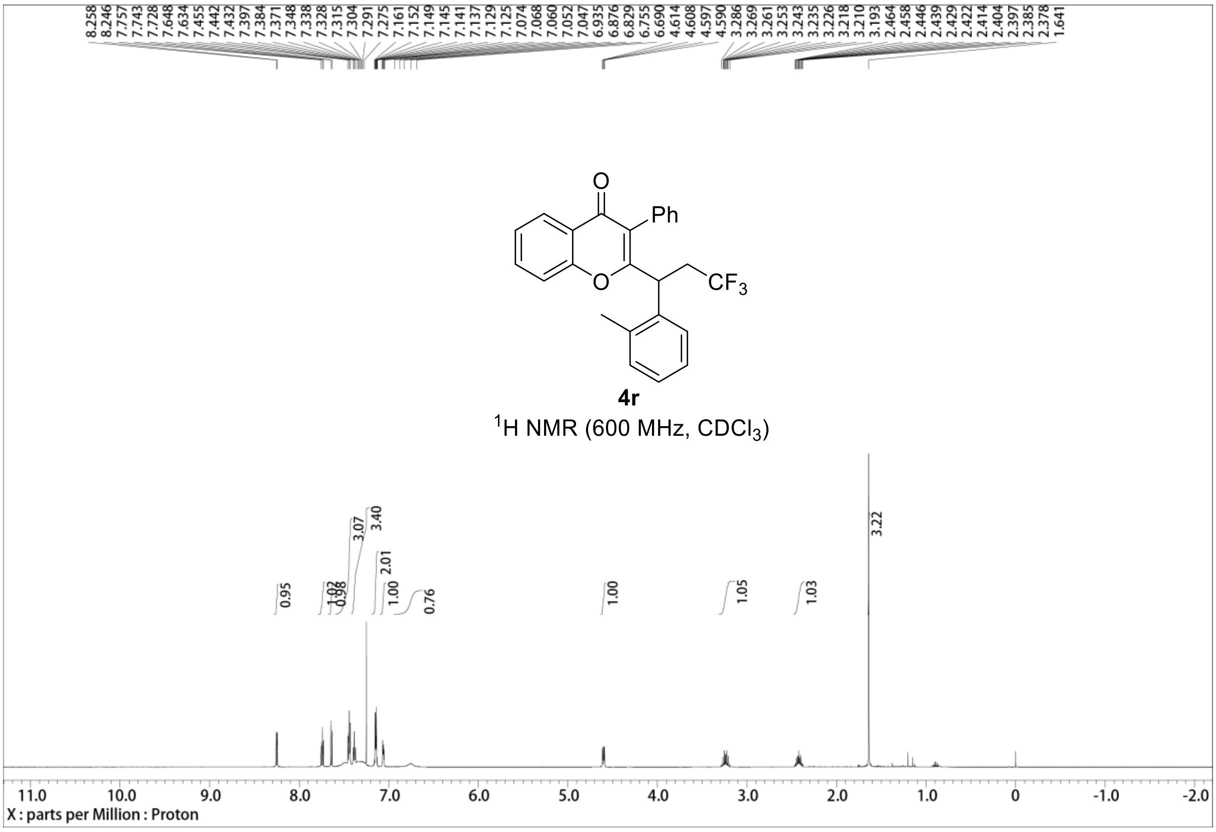

8.258
8.246
7.757
7.743
7.728
7.648
7.634
7.455
7.442
7.432
7.397
7.384
7.371
7.348
7.338
7.328
7.315
7.304
7.291
7.275
7.161
7.152
7.149
7.145
7.141
7.137
7.129
7.125
7.074
7.068
7.060
7.052
7.047
6.935
6.876
6.829
6.755
6.690
4.614
4.608
4.597
4.590
3.286
3.269
3.261
3.253
3.243
3.235
3.226
3.218
3.210
3.193
2.464
2.458
2.446
2.439
2.429
2.422
2.414
2.404
2.397
2.385
2.378
1.641
O
Ph
O
CF3
4r
1H NMR (600 MHz, CDCl3)
X : parts per Million : Proton

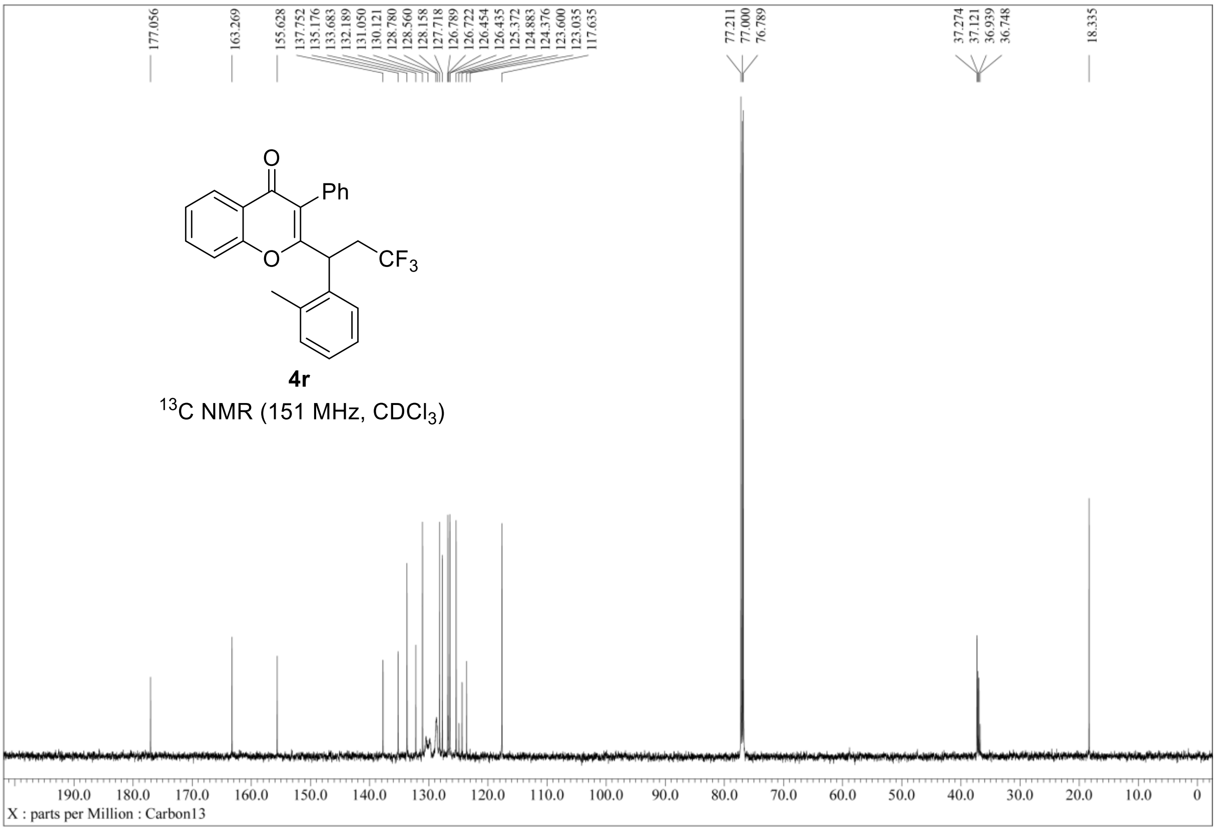
177.056
163.269
155.628
137.752
135.176
133.683
132.189
131.050
130.121
128.780
128.560
128.158
127.718
126.789
126.722
126.454
126.435
125.372
124.883
124.376
123.600
123.035
117.635
77.211
77.000
76.789
37.274
37.121
36.939
36.748
18.335
Ph
CF3
4r
13C NMR (151 MHz, CDCl3)
190.0 180.0 170.0 160.0 150.0 140.0 130.0 120.0 110.0 100.0 90.0 80.0 70.0 60.0 50.0 40.0 30.0 20.0 10.0 0
X : parts per Million : Carbon13

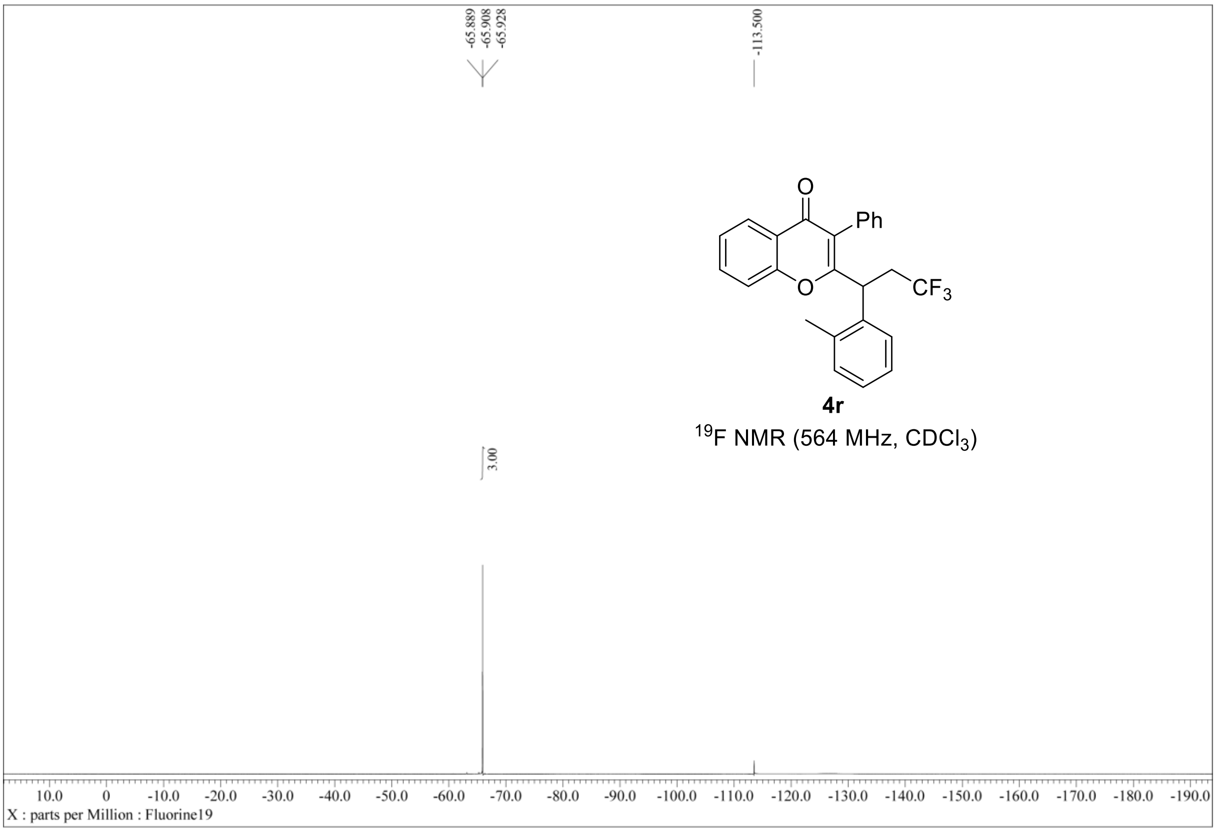
-65.889
-65.908
-65.928
-113.500
Ph
CF3
4r
19F NMR (564 MHz, CDCl3)
3.00
10.0 0 -10.0 -20.0 -30.0 -40.0 -50.0 -60.0 -70.0 -80.0 -90.0 -100.0 -110.0 -120.0 -130.0 -140.0 -150.0 -160.0 -170.0 -180.0 -190.0
X : parts per Million : Fluorine19

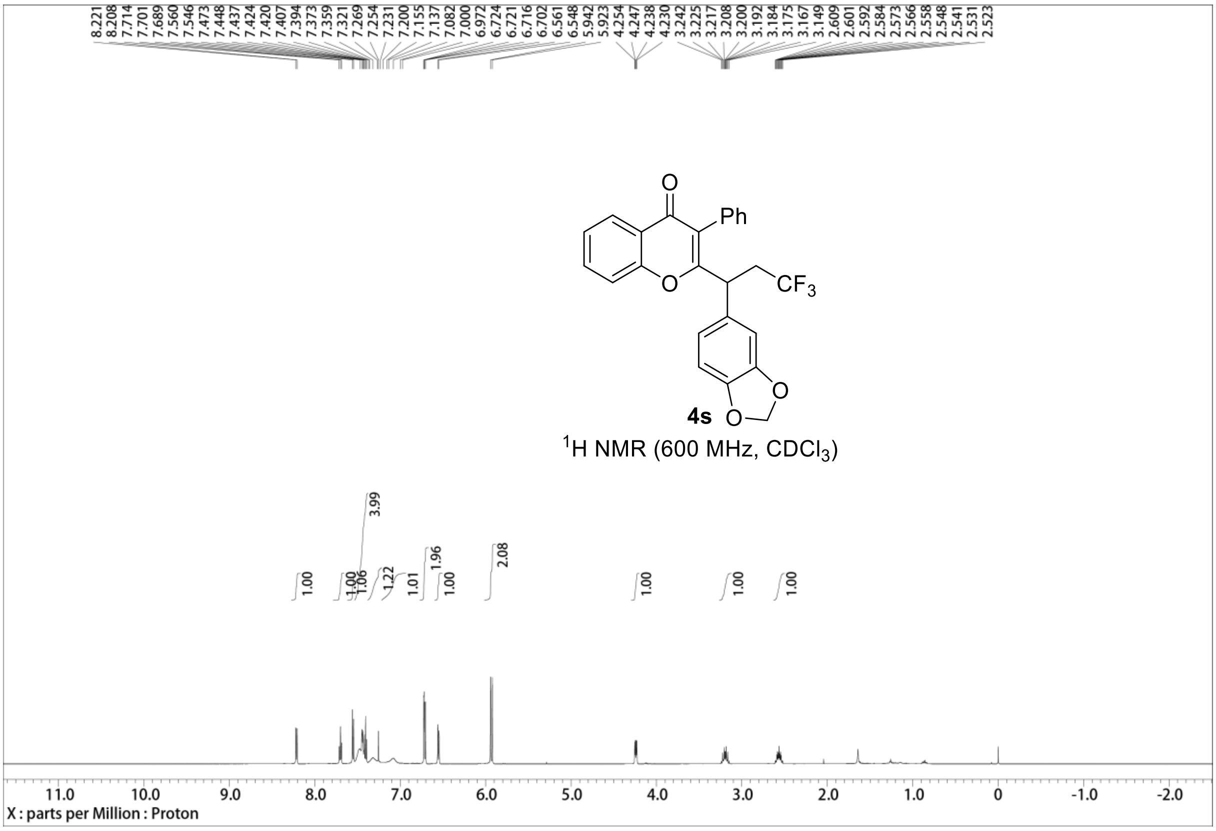
8.221 8.208 7.714 7.701 7.689 7.560 7.546 7.473 7.448 7.437 7.424 7.420 7.407 7.394 7.373 7.359 7.321 7.269 7.254 7.231 7.200 7.155 7.137 7.082 7.000 6.972 6.724 6.721 6.716 6.702 6.561 6.548 5.942 5.923 4.254 4.247 4.238 4.230 3.242 3.225 3.217 3.208 3.200 3.192 3.184 3.175 3.167 3.149 2.609 2.601 2.592 2.584 2.573 2.566 2.558 2.548 2.541 2.531 2.523
O
Ph
O
CF3
O
O
4s
1H NMR (600 MHz, CDCl3)
1.00 1.00 1.06 3.99 1.22 1.01 1.96 1.00 2.08 1.00 1.00 1.00
11.0 10.0 9.0 8.0 7.0 6.0 5.0 4.0 3.0 2.0 1.0 0 -1.0 -2.0
X : parts per Million : Proton

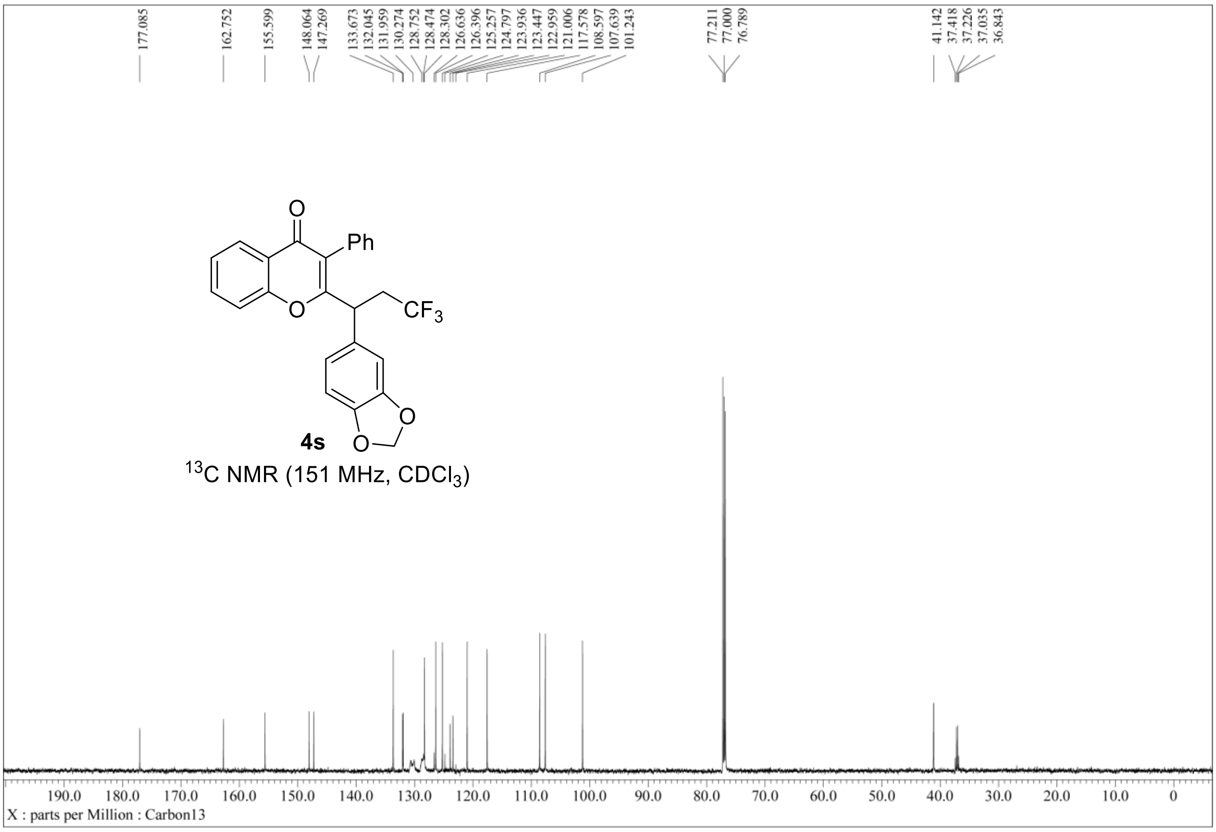
177.085 162.752 155.599 148.064 147.269 133.673 132.045 131.959 130.274 128.752 128.474 128.302 126.636 126.396 125.257 124.797 123.936 123.447 122.959 121.006 117.578 108.597 107.639 101.243 77.211 77.000 76.789 41.142 37.418 37.226 37.035 36.843
O
Ph
O
CF3
O
O
4s
13C NMR (151 MHz, CDCl3)
190.0 180.0 170.0 160.0 150.0 140.0 130.0 120.0 110.0 100.0 90.0 80.0 70.0 60.0 50.0 40.0 30.0 20.0 10.0 0
X : parts per Million : Carbon13

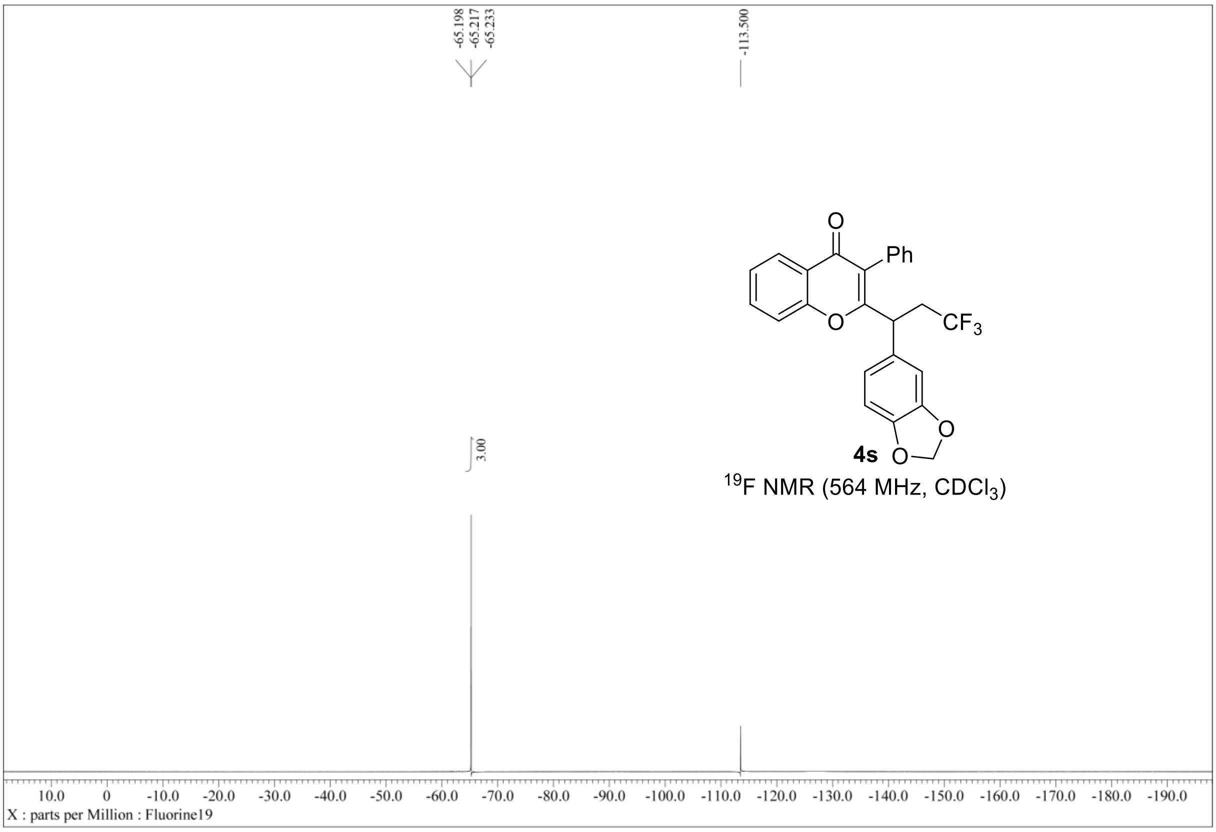
-65.198
-65.217
-65.233
-113.500
3.00
O
Ph
O
CF3
O
O
4s
19F NMR (564 MHz, CDCl3)
10.0 0 -10.0 -20.0 -30.0 -40.0 -50.0 -60.0 -70.0 -80.0 -90.0 -100.0 -110.0 -120.0 -130.0 -140.0 -150.0 -160.0 -170.0 -180.0 -190.0
X : parts per Million : Fluorine19

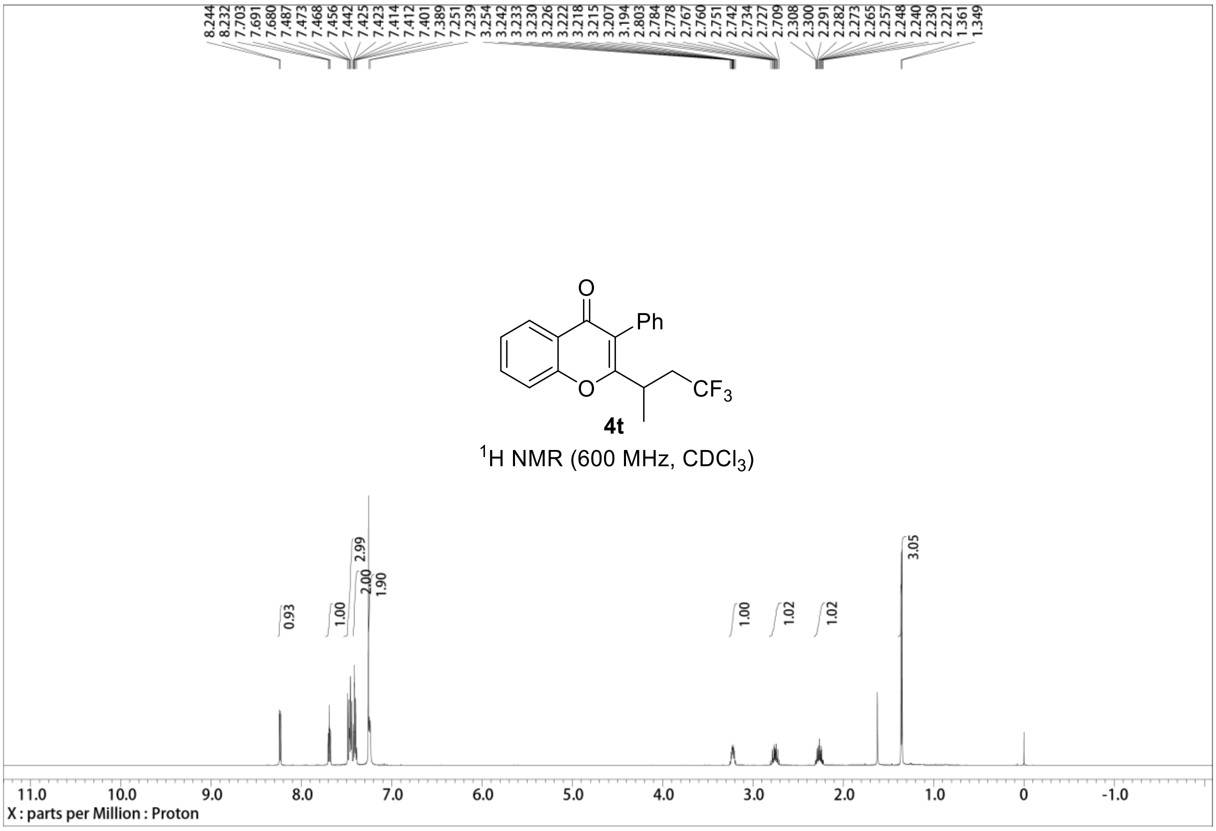
8.244 8.232 7.703 7.691 7.680 7.487 7.473 7.468 7.456 7.442 7.425 7.423 7.414 7.412 7.401 7.389 7.251 7.239 3.254 3.242 3.233 3.230 3.226 3.222 3.218 3.215 3.207 3.194 2.803 2.784 2.778 2.767 2.760 2.751 2.742 2.734 2.727 2.709 2.308 2.300 2.291 2.282 2.273 2.265 2.257 2.248 2.240 2.230 2.221 1.361 1.349
O
Ph
O
CF3
4t
1H NMR (600 MHz, CDCl3)
0.93
1.00
2.00
2.99
1.90
1.00
1.02
1.02
3.05
11.0 10.0 9.0 8.0 7.0 6.0 5.0 4.0 3.0 2.0 1.0 0 -1.0
X : parts per Million : Proton

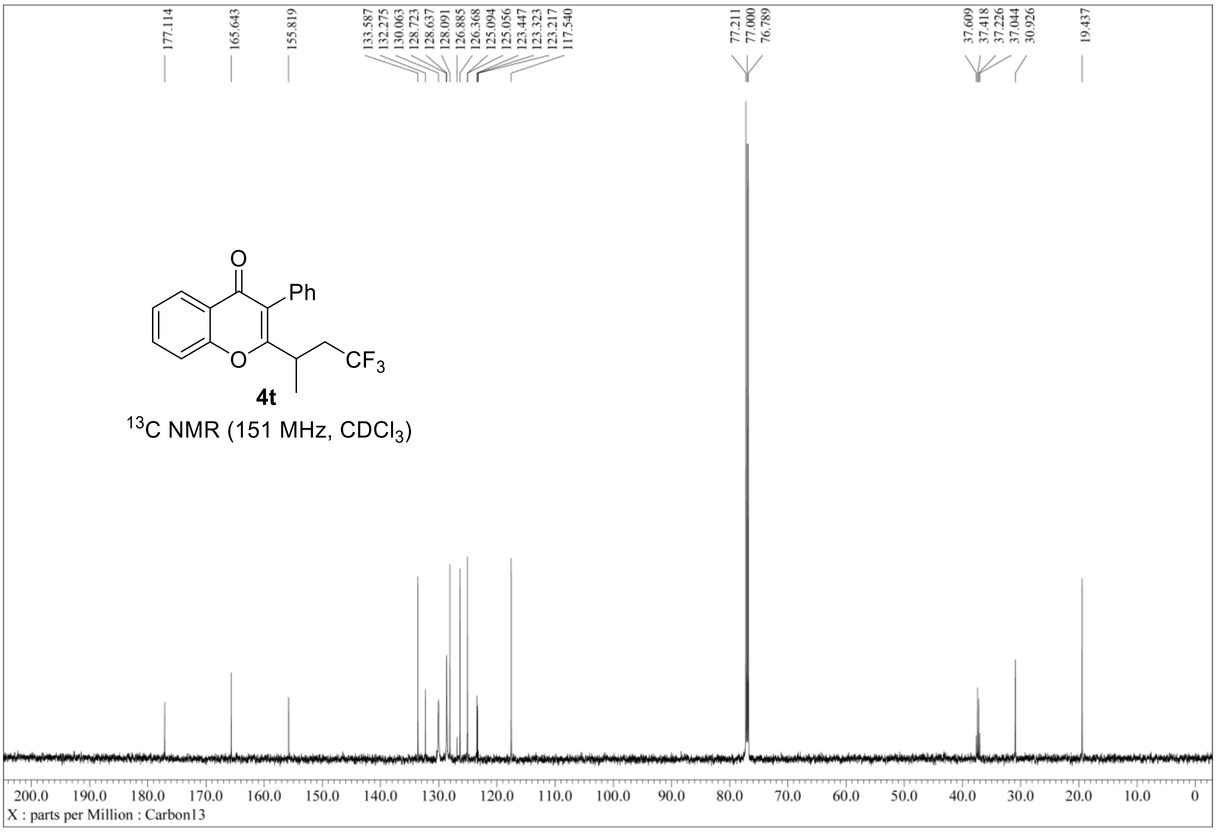
177.114
165.643
155.819
133.587
132.275
130.063
128.723
128.637
128.091
126.885
126.368
125.094
125.056
123.447
123.323
123.217
117.540
77.211
77.000
76.789
37.609
37.418
37.226
37.044
30.926
19.437
O
Ph
O
CF3
4t
13C NMR (151 MHz, CDCl3)
200.0 190.0 180.0 170.0 160.0 150.0 140.0 130.0 120.0 110.0 100.0 90.0 80.0 70.0 60.0 50.0 40.0 30.0 20.0 10.0 0
X : parts per Million : Carbon13

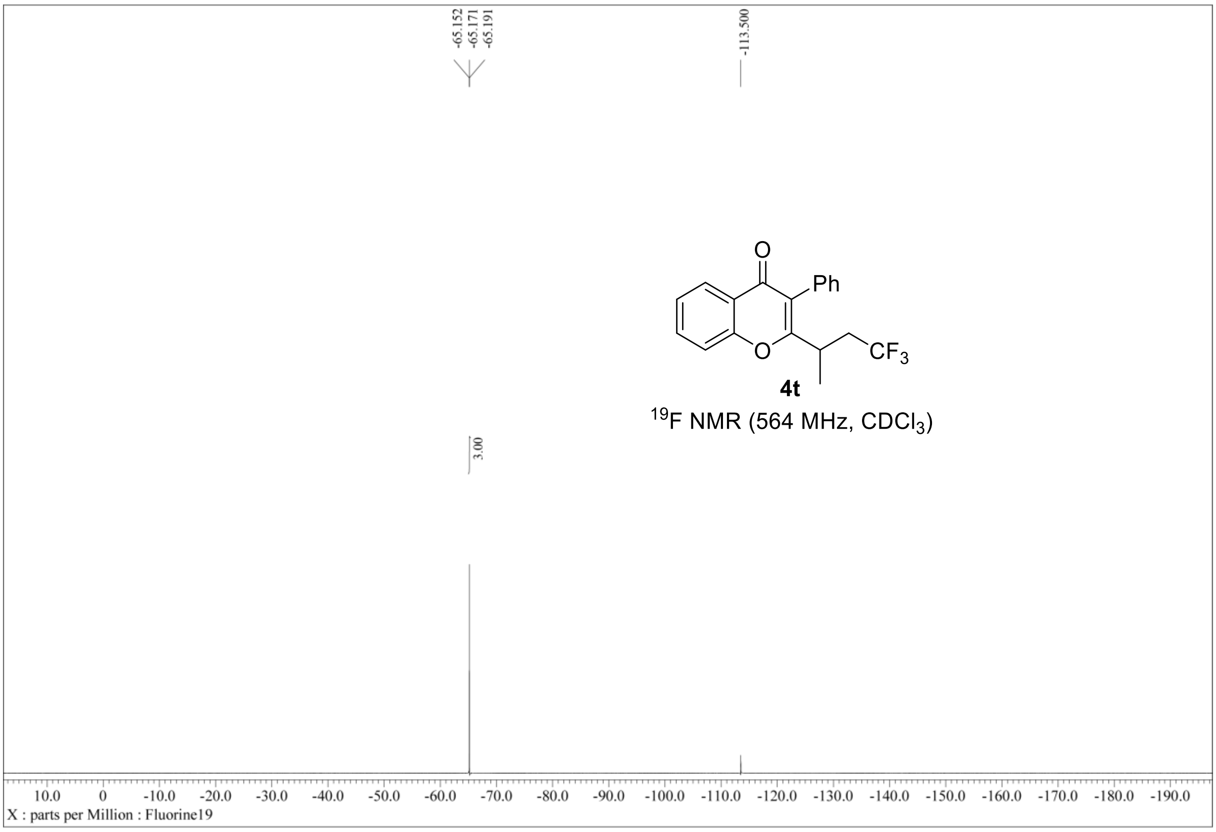
-65.152
-65.171
-65.191
-113.500
O
Ph
O
CF3
4t
19F NMR (564 MHz, CDCl3)
3.00
10.0 0 -10.0 -20.0 -30.0 -40.0 -50.0 -60.0 -70.0 -80.0 -90.0 -100.0 -110.0 -120.0 -130.0 -140.0 -150.0 -160.0 -170.0 -180.0 -190.0
X : parts per Million : Fluorine19

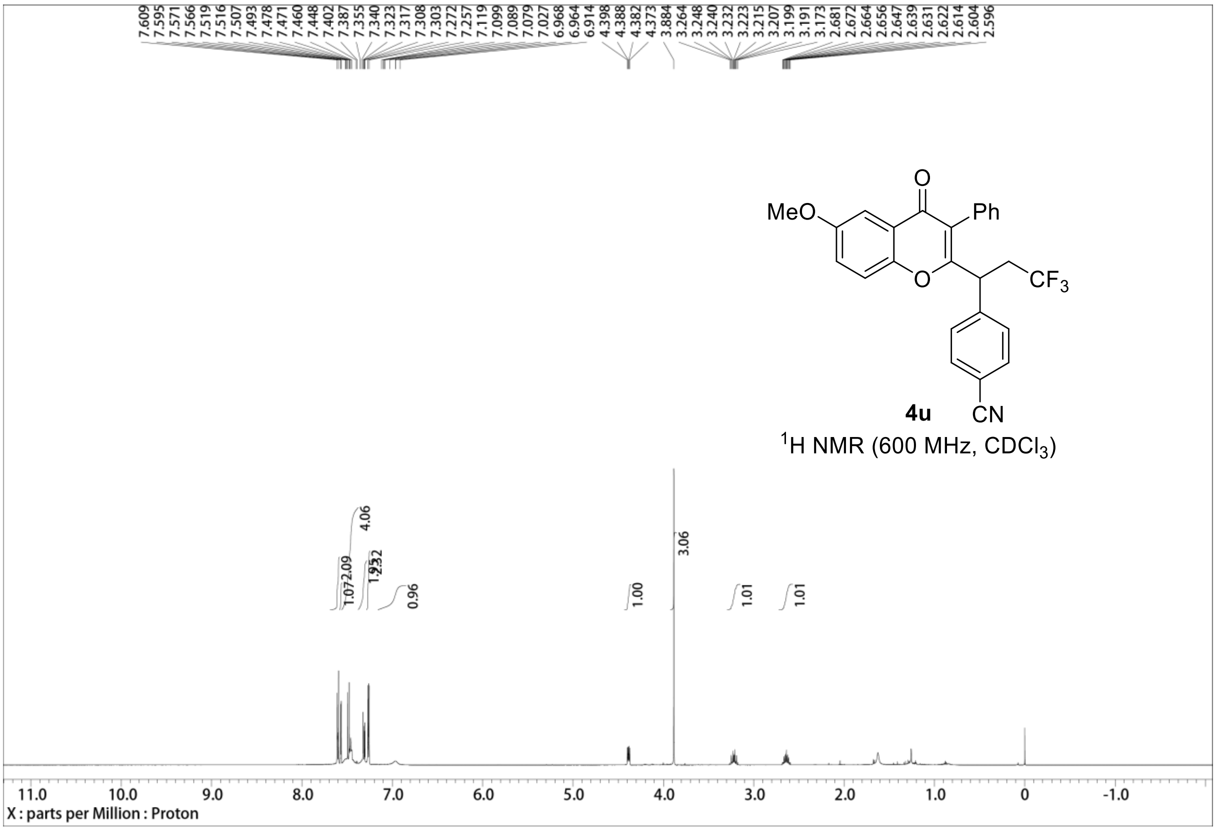
7.609 7.595 7.571 7.566 7.519 7.516 7.507 7.493 7.478 7.471 7.460 7.448 7.402 7.387 7.355 7.340 7.323 7.317 7.308 7.303 7.272 7.257 7.119 7.099 7.089 7.079 7.027 6.968 6.964 6.914 4.398 4.388 4.382 4.373 3.884 3.264 3.248 3.240 3.232 3.223 3.215 3.207 3.199 3.191 3.173 2.681 2.672 2.664 2.656 2.647 2.639 2.631 2.622 2.614 2.604 2.596
MeO
O
Ph
O
CF3
CN
4u
1H NMR (600 MHz, CDCl3)
1.07 2.09 4.06 1.95 2.32 0.96 1.00 3.06 1.01 1.01
11.0 10.0 9.0 8.0 7.0 6.0 5.0 4.0 3.0 2.0 1.0 0 -1.0
X : parts per Million : Proton

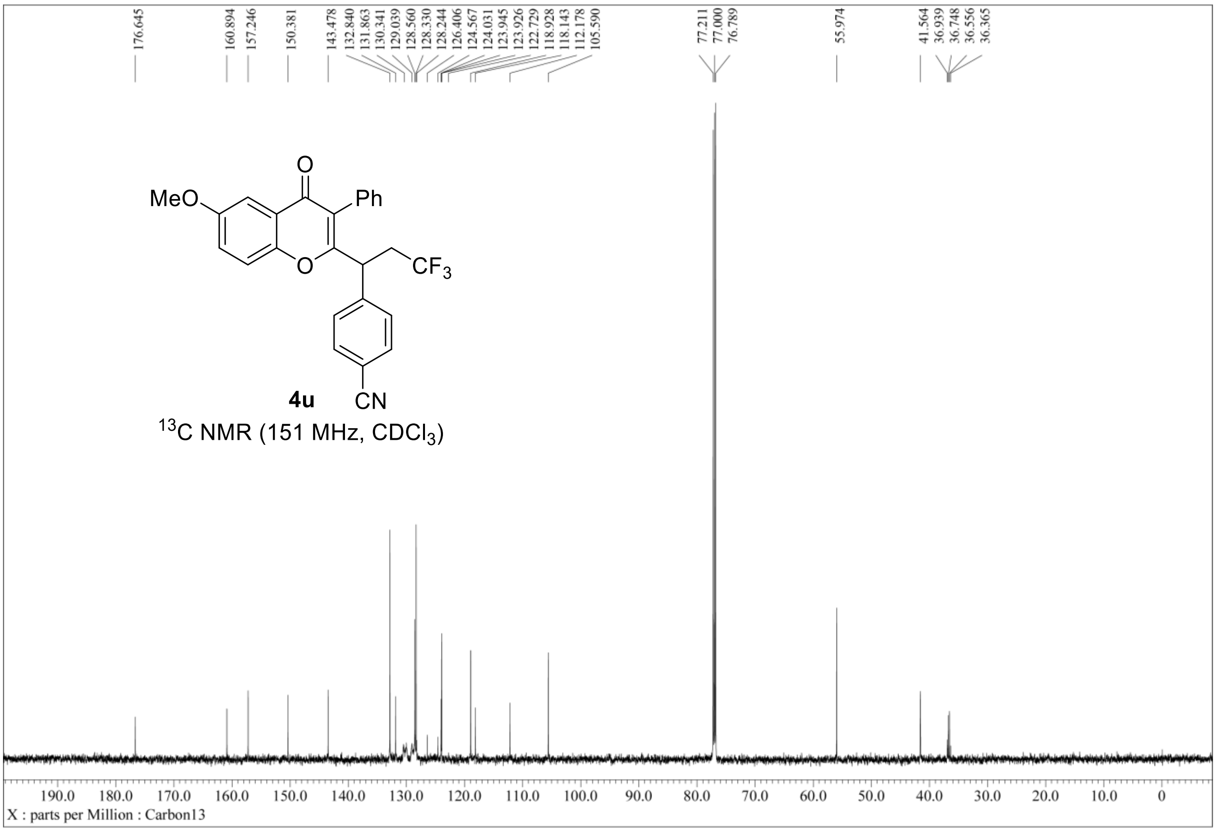
176.645 160.894 157.246 150.381 143.478 132.840 131.863 130.341 129.039 128.560 128.330 128.244 126.406 124.567 124.031 123.945 123.926 122.729 118.928 118.143 112.178 105.590 77.211 77.000 76.789 55.974 41.564 36.939 36.748 36.556 36.365
MeO
O
Ph
O
CF3
CN
4u
13C NMR (151 MHz, CDCl3)
190.0 180.0 170.0 160.0 150.0 140.0 130.0 120.0 110.0 100.0 90.0 80.0 70.0 60.0 50.0 40.0 30.0 20.0 10.0 0
X : parts per Million : Carbon13

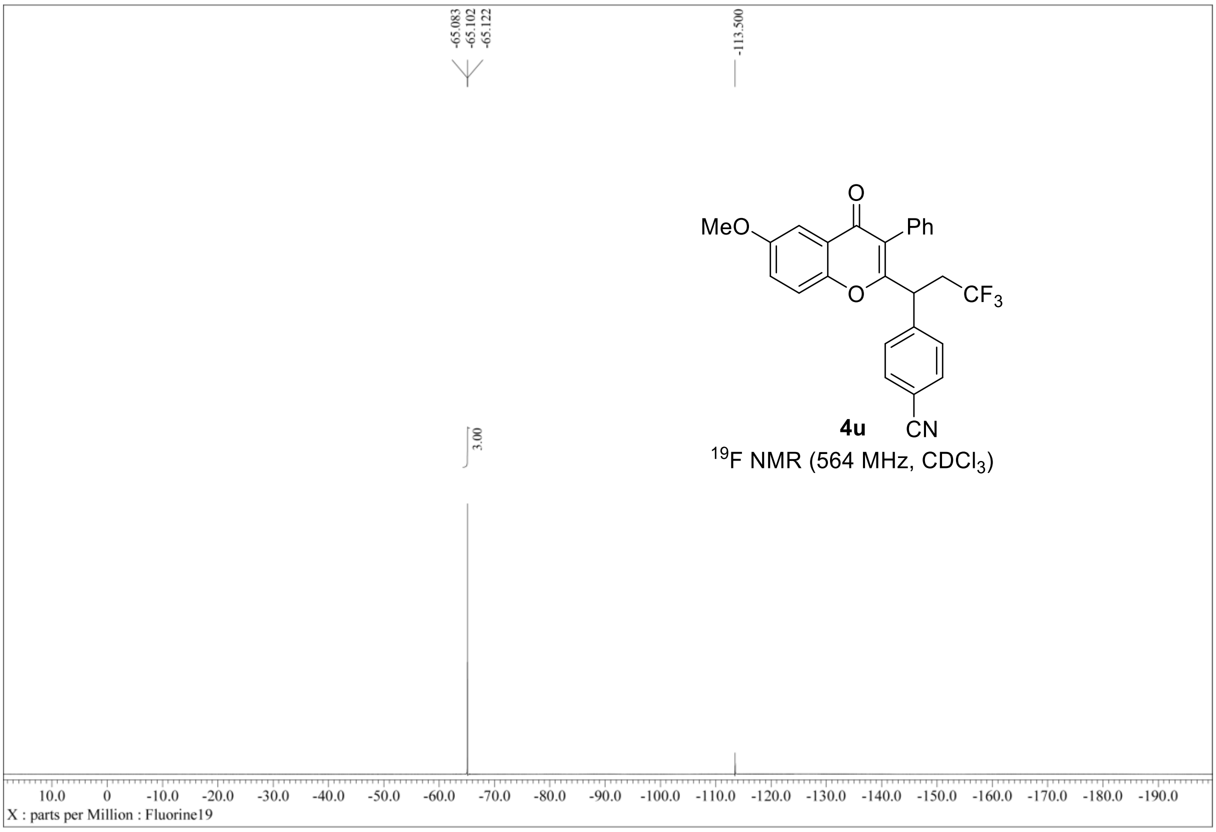
-65.083
-65.102
-65.122
-113.500
3.00
MeO
O
Ph
CF3
4u
CN
19F NMR (564 MHz, CDCl3)
10.0 0 -10.0 -20.0 -30.0 -40.0 -50.0 -60.0 -70.0 -80.0 -90.0 -100.0 -110.0 -120.0 -130.0 -140.0 -150.0 -160.0 -170.0 -180.0 -190.0
X : parts per Million : Fluorine19

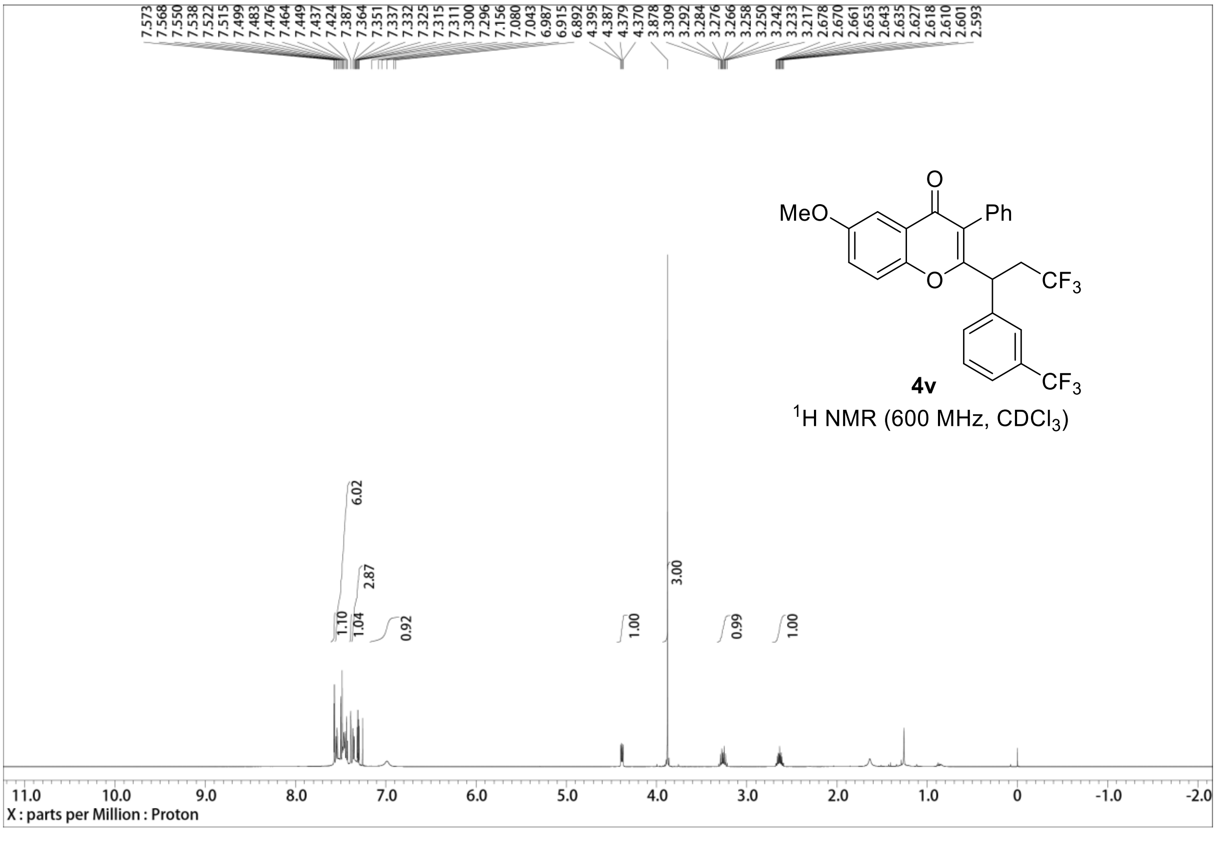
7.573 7.568 7.550 7.538 7.522 7.515 7.499 7.483 7.476 7.464 7.449 7.437 7.424 7.387 7.364 7.351 7.337 7.332 7.325 7.315 7.311 7.300 7.296 7.156 7.080 7.043 6.987 6.915 6.892 4.395 4.387 4.379 4.370 3.878 3.309 3.292 3.284 3.276 3.266 3.258 3.250 3.242 3.233 3.217 2.678 2.670 2.661 2.653 2.643 2.635 2.627 2.618 2.610 2.601 2.593
6.02
2.87
1.10
1.04
0.92
1.00
3.00
0.99
1.00
MeO
O
Ph
CF3
4v
CF3
1H NMR (600 MHz, CDCl3)
11.0 10.0 9.0 8.0 7.0 6.0 5.0 4.0 3.0 2.0 1.0 0 -1.0 -2.0
X : parts per Million : Proton

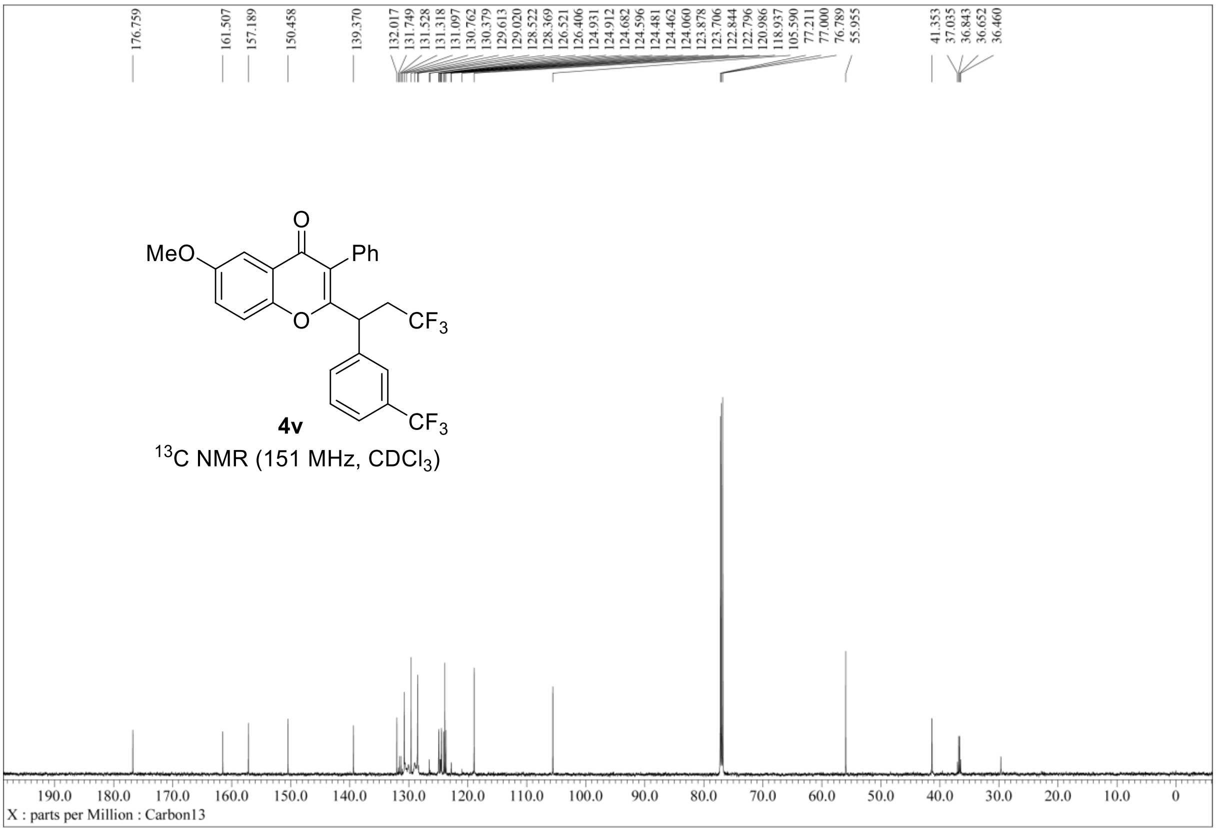
176.759
161.507
157.189
150.458
139.370
132.017
131.749
131.528
131.318
131.097
130.762
130.379
129.613
129.020
128.522
128.369
126.521
126.406
124.931
124.912
124.682
124.596
124.481
124.462
124.060
123.878
123.706
122.844
122.796
120.986
118.937
105.590
77.211
77.000
76.789
55.955
41.353
37.035
36.843
36.652
36.460
MeO
O
Ph
O
CF3
CF3
4v
13C NMR (151 MHz, CDCl3)
190.0 180.0 170.0 160.0 150.0 140.0 130.0 120.0 110.0 100.0 90.0 80.0 70.0 60.0 50.0 40.0 30.0 20.0 10.0 0
X : parts per Million : Carbon13

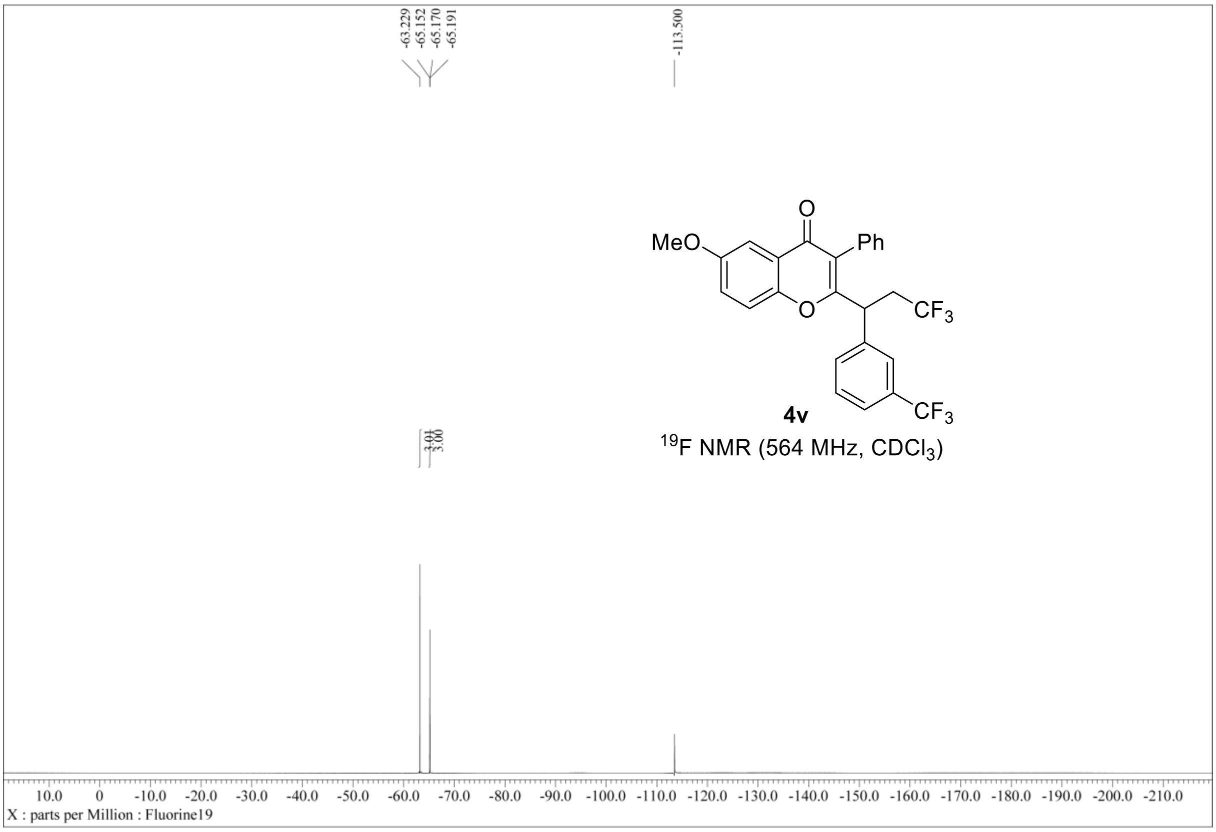
-63.229
-65.152
-65.170
-65.191
-113.500
MeO
O
Ph
O
CF3
CF3
4v
19F NMR (564 MHz, CDCl3)
3.01
3.00
10.0 0 -10.0 -20.0 -30.0 -40.0 -50.0 -60.0 -70.0 -80.0 -90.0 -100.0 -110.0 -120.0 -130.0 -140.0 -150.0 -160.0 -170.0 -180.0 -190.0 -200.0 -210.0
X : parts per Million : Fluorine19

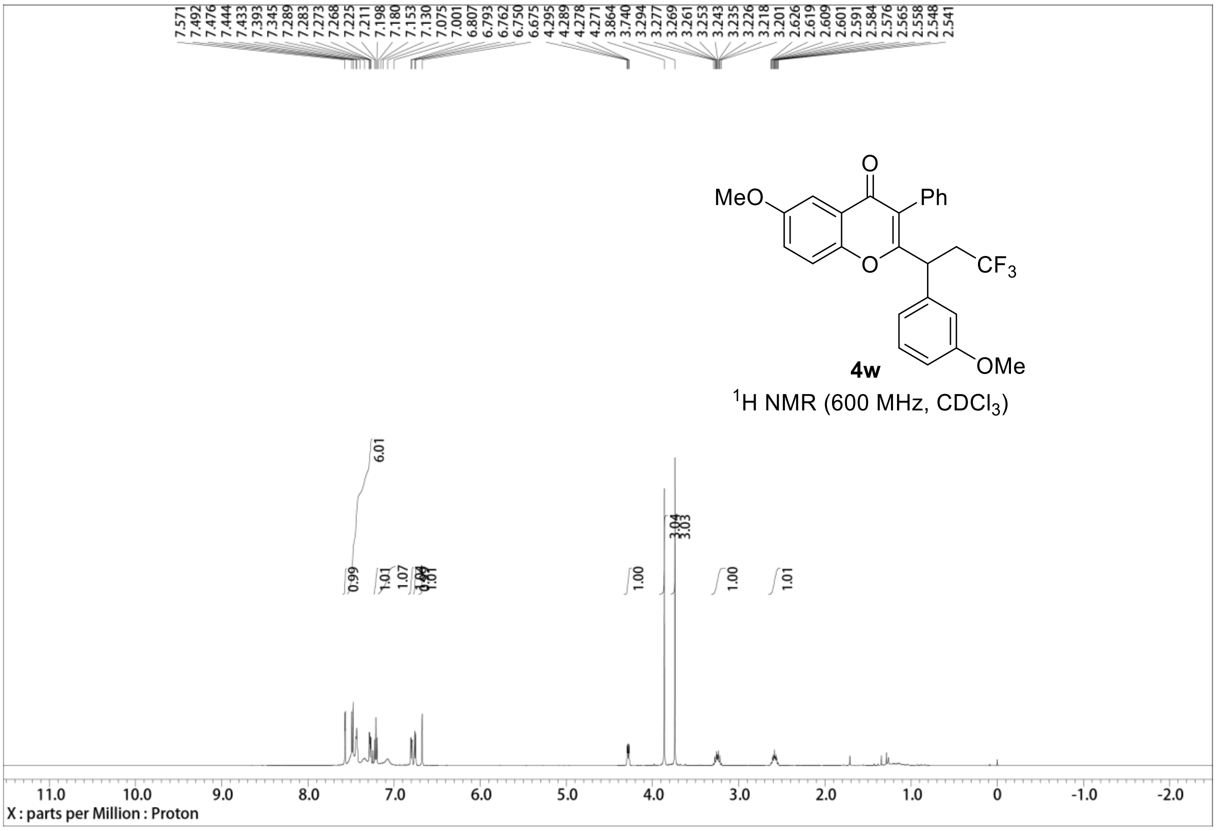
MeO
O
Ph
CF3
OMe
4w
1H NMR (600 MHz, CDCl3)
X : parts per Million : Proton

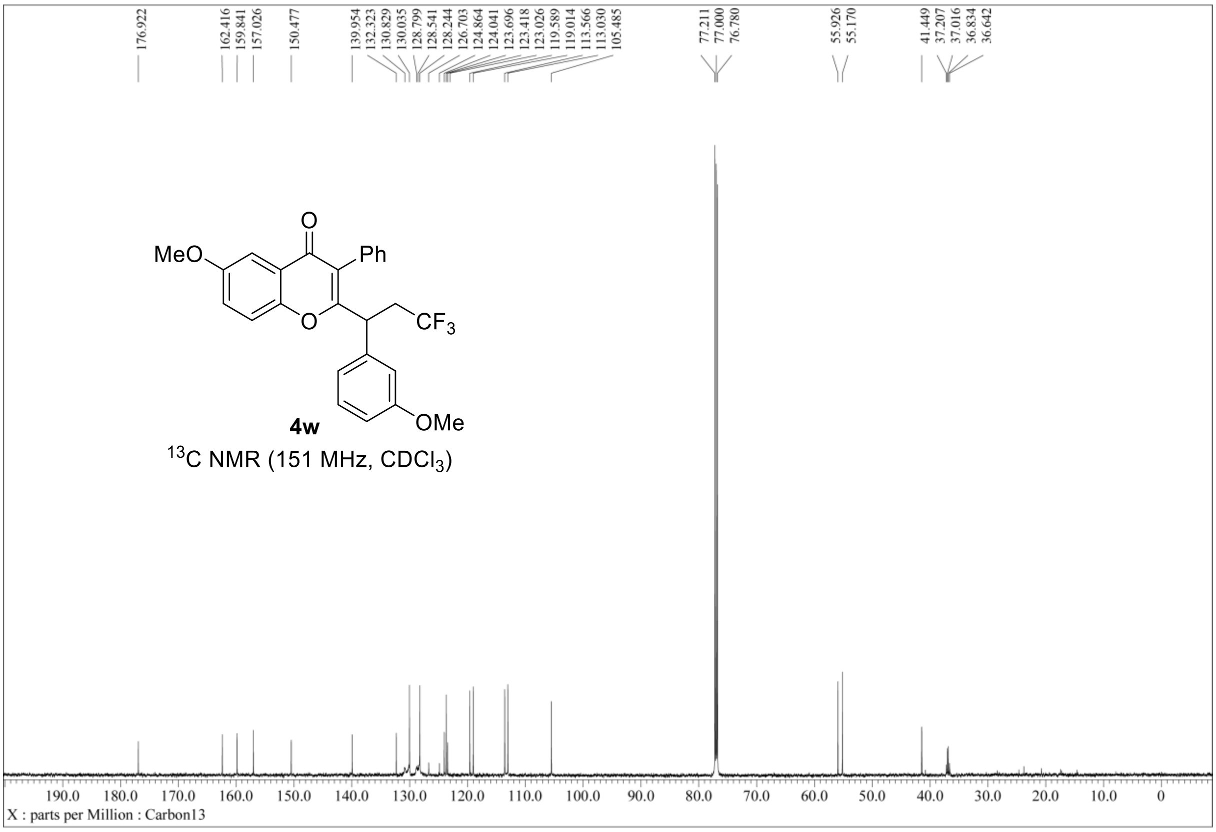
MeO
O
Ph
CF3
OMe
4w
13C NMR (151 MHz, CDCl3)
X : parts per Million : Carbon13

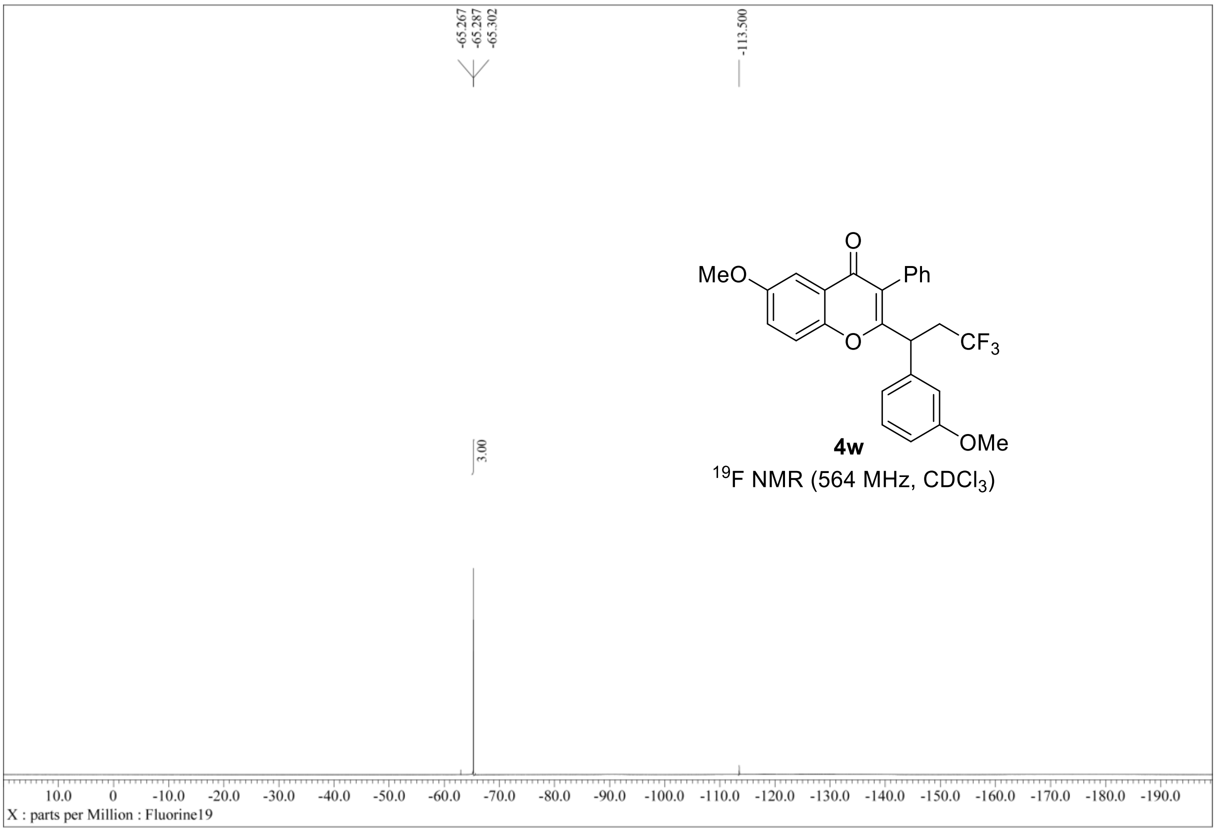
-65.267
-65.287
-65.302
-113.500
3.00
MeO
O
Ph
O
CF3
OMe
4w
19F NMR (564 MHz, CDCl3)
10.0 0 -10.0 -20.0 -30.0 -40.0 -50.0 -60.0 -70.0 -80.0 -90.0 -100.0 -110.0 -120.0 -130.0 -140.0 -150.0 -160.0 -170.0 -180.0 -190.0
X : parts per Million : Fluorine19

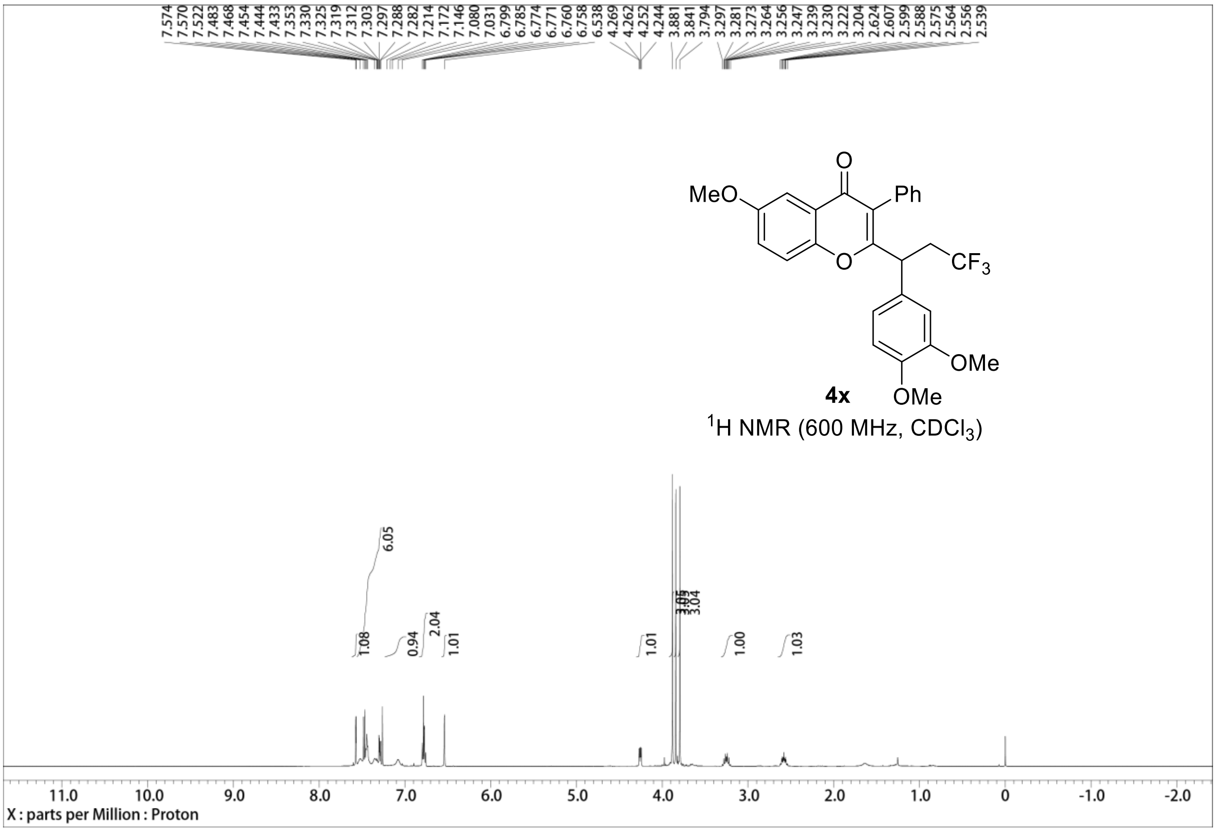
MeO
O
Ph
O
CF3
OMe
OMe
4x
1H NMR (600 MHz, CDCl3)
6.05
1.08
0.94
2.04
1.01
1.01
3.04
1.00
1.03
11.0 10.0 9.0 8.0 7.0 6.0 5.0 4.0 3.0 2.0 1.0 0 -1.0 -2.0
X : parts per Million : Proton

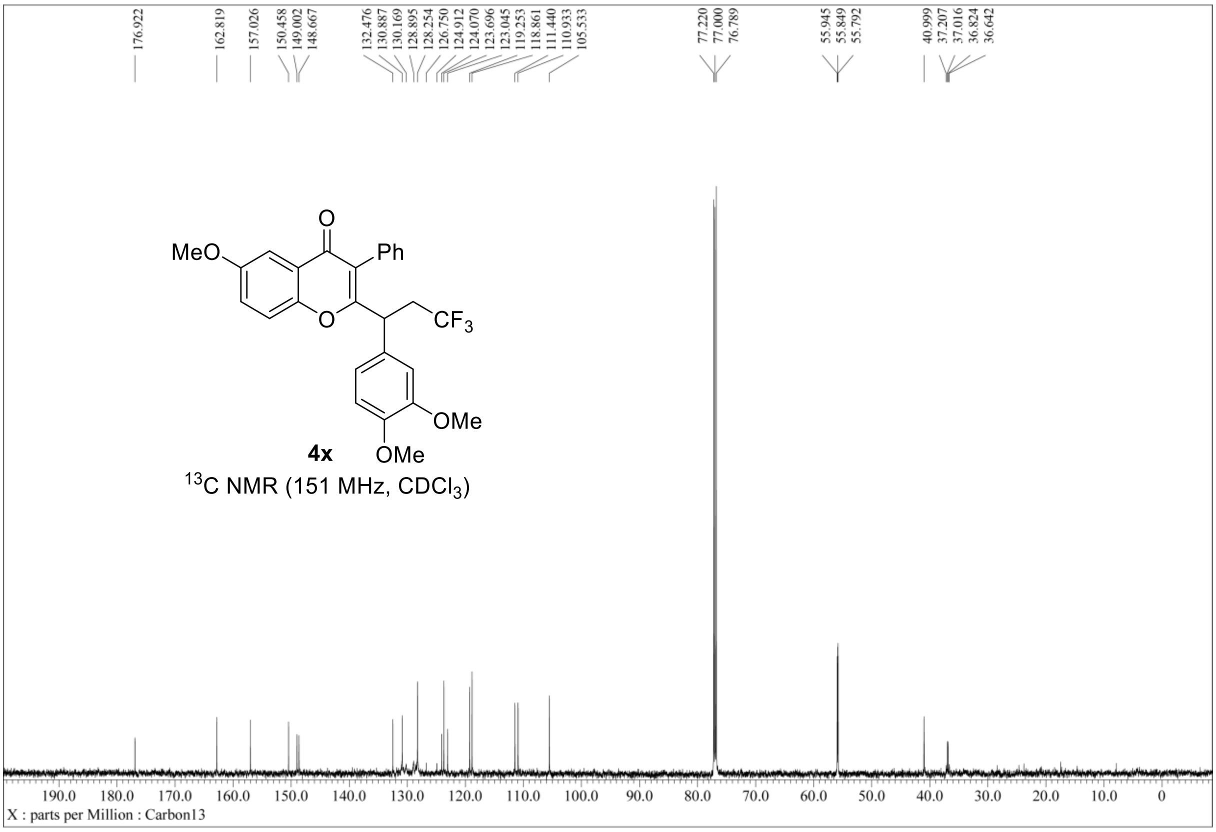
176.922
162.819
157.026
150.458
149.002
148.667
132.476
130.887
130.169
128.895
128.254
126.750
124.912
124.070
123.696
123.045
119.253
118.861
111.440
110.933
105.533
77.220
77.000
76.789
55.945
55.849
55.792
40.999
37.207
37.016
36.824
36.642
MeO
O
Ph
CF3
OMe
4x
OMe
13C NMR (151 MHz, CDCl3)
X : parts per Million : Carbon13

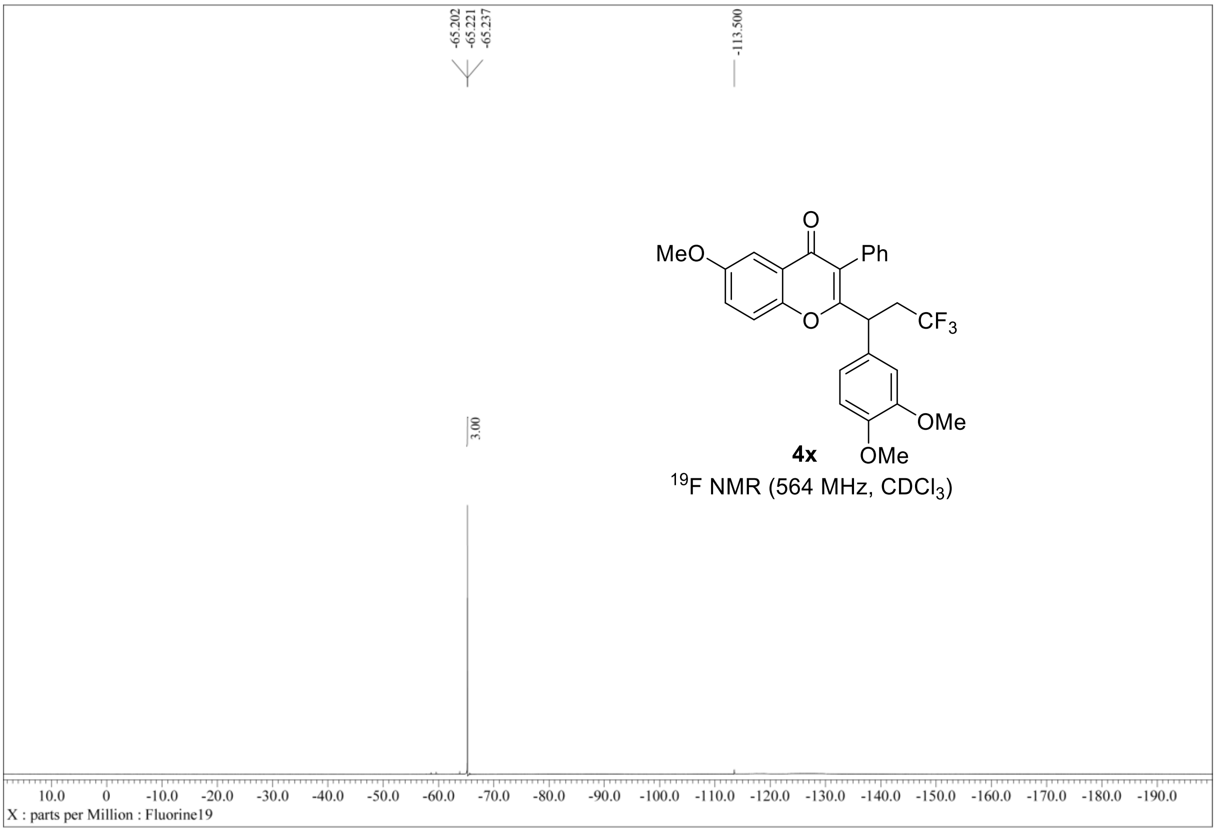
-65.202
-65.221
-65.237
-113.500
3.00
MeO
O
Ph
CF3
OMe
4x
OMe
19F NMR (564 MHz, CDCl3)
X : parts per Million : Fluorine19

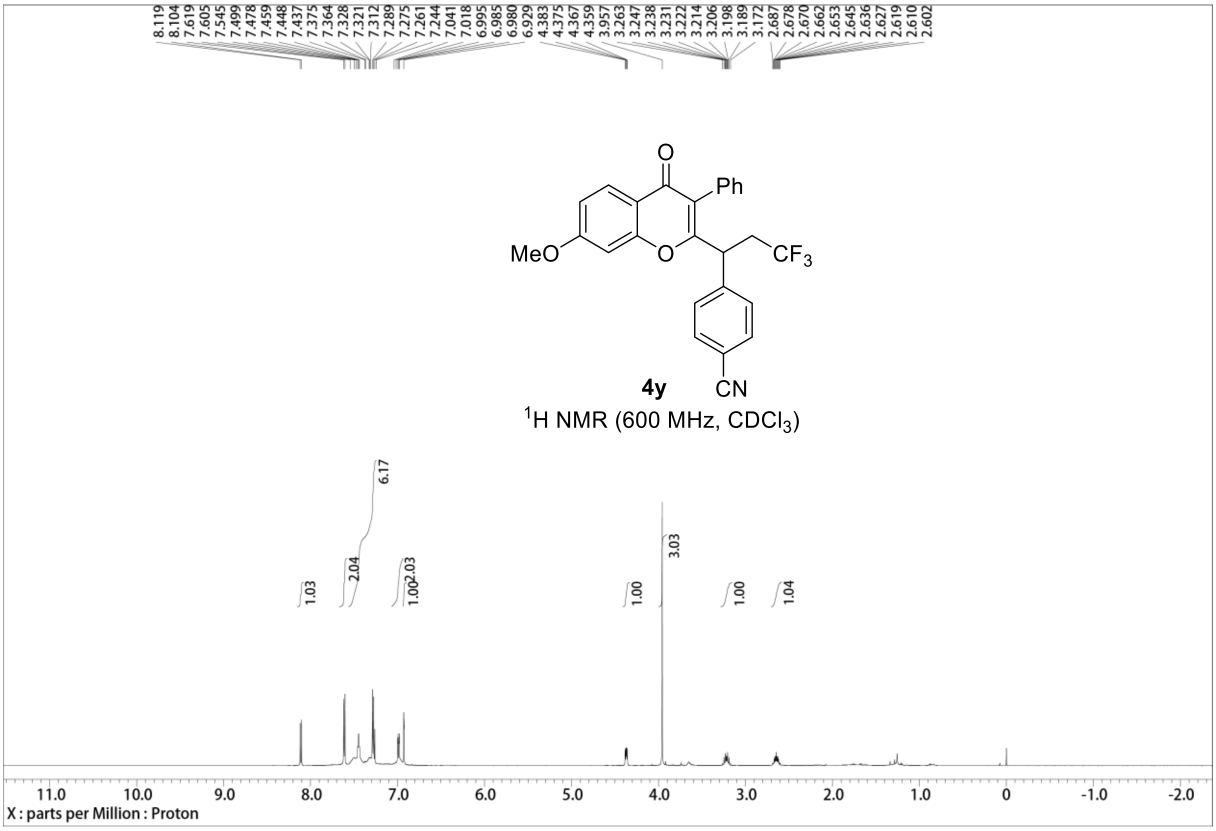
8.119 8.104 7.619 7.605 7.545 7.499 7.478 7.459 7.448 7.437 7.375 7.364 7.328 7.321 7.312 7.289 7.275 7.261 7.244 7.041 7.018 6.995 6.985 6.980 6.929 4.383 4.375 4.367 4.359 3.957 3.263 3.247 3.238 3.231 3.222 3.214 3.206 3.198 3.189 3.172 2.687 2.678 2.670 2.662 2.653 2.645 2.636 2.627 2.619 2.610 2.602
O
Ph
MeO
O
CF3
4y
CN
1H NMR (600 MHz, CDCl3)
1.03
2.04
6.17
1.00
2.03
1.00
3.03
1.00
1.04
11.0 10.0 9.0 8.0 7.0 6.0 5.0 4.0 3.0 2.0 1.0 0 -1.0 -2.0
X : parts per Million : Proton

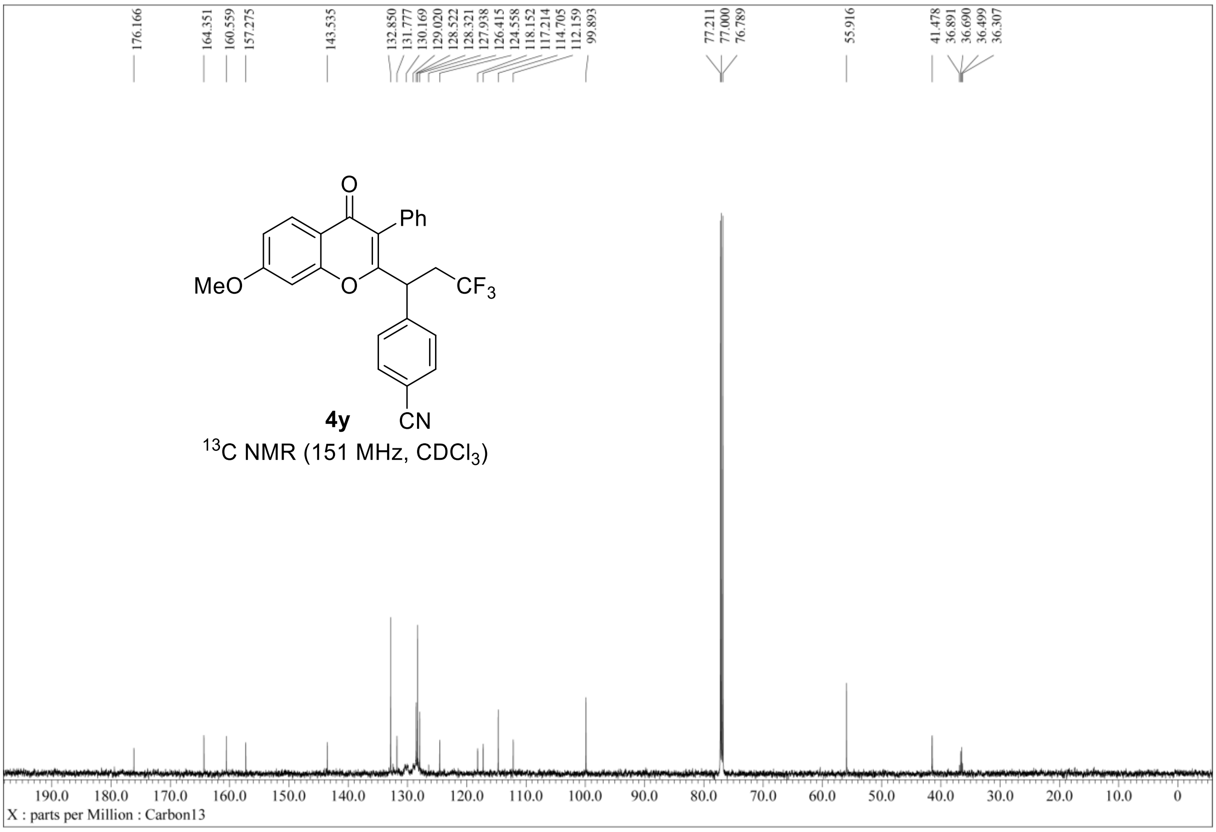
176.166 164.351 160.559 157.275 143.535 132.850 131.777 130.169 129.020 128.522 128.321 127.938 126.415 124.558 118.152 117.214 114.705 112.159 99.893 77.211 77.000 76.789 55.916 41.478 36.891 36.690 36.499 36.307
O
Ph
MeO
O
CF3
4y
CN
13C NMR (151 MHz, CDCl3)
190.0 180.0 170.0 160.0 150.0 140.0 130.0 120.0 110.0 100.0 90.0 80.0 70.0 60.0 50.0 40.0 30.0 20.0 10.0 0
X : parts per Million : Carbon13

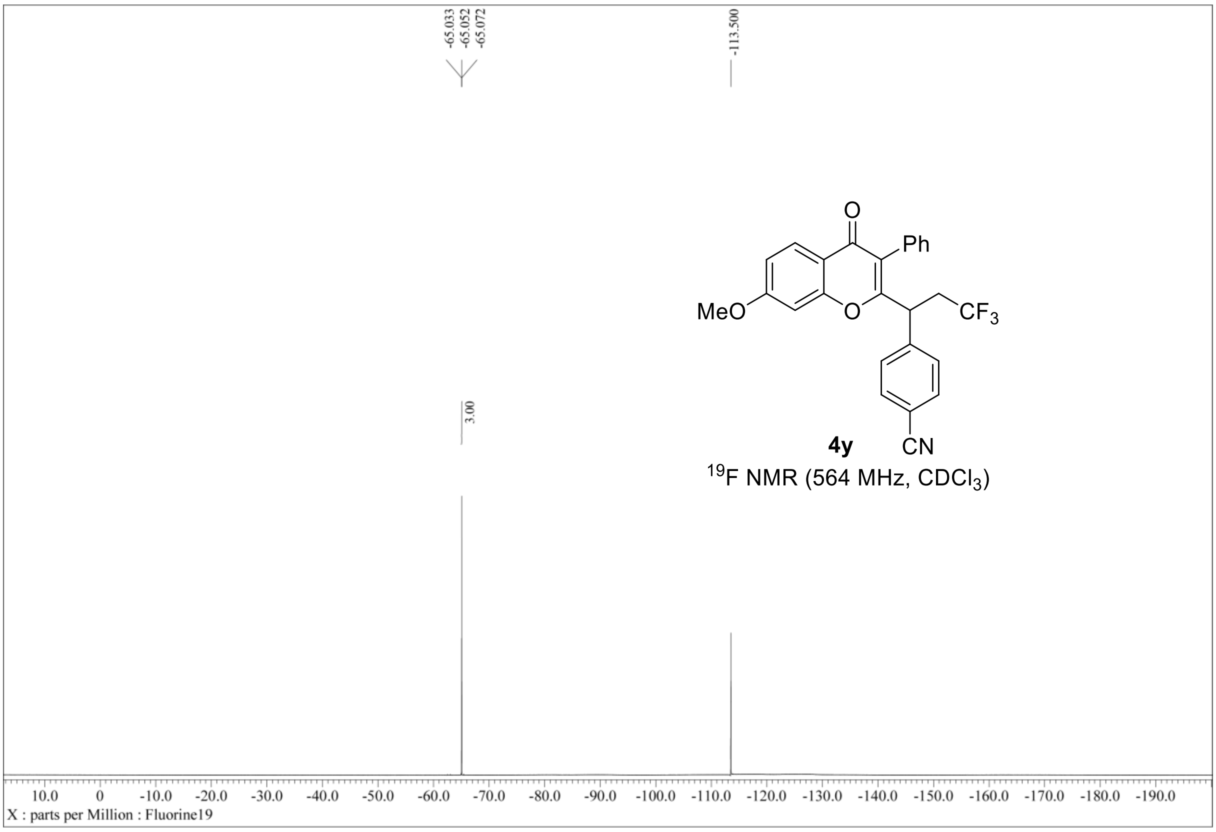
-65.033
-65.052
-65.072
-113.500
3.00
4y
19F NMR (564 MHz, CDCl3)
X : parts per Million : Fluorine19

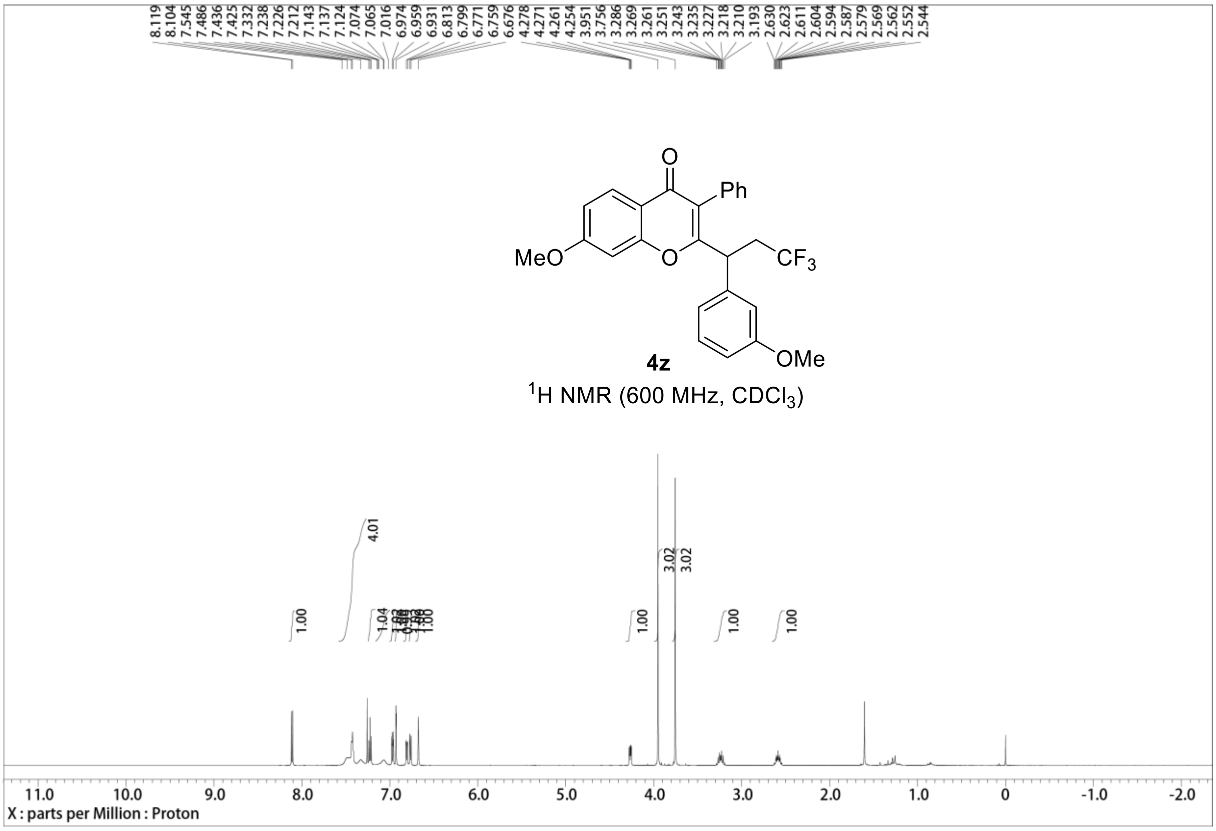
4z
1H NMR (600 MHz, CDCl3)
X : parts per Million : Proton

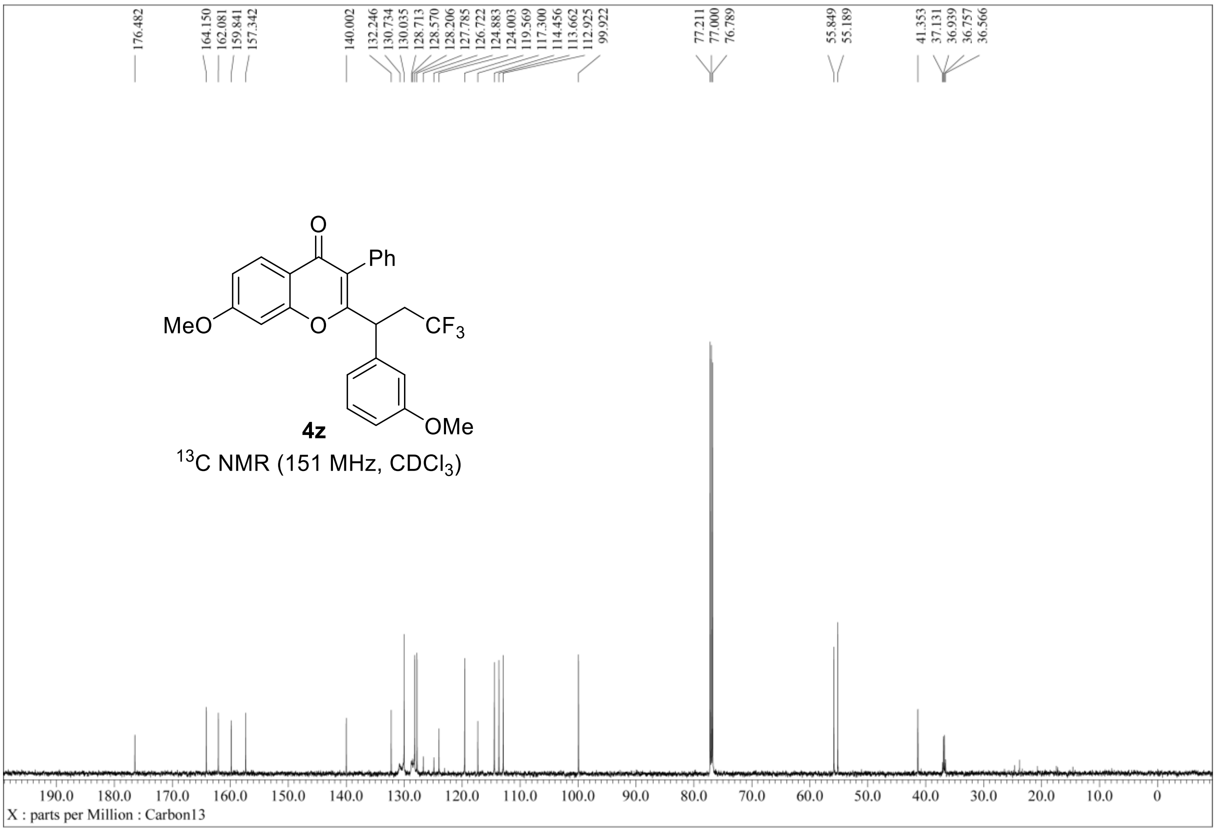
176.482
164.150
162.081
159.841
157.342
140.002
132.246
130.734
130.035
128.713
128.570
128.206
127.785
126.722
124.883
124.003
119.569
117.300
114.456
113.662
112.925
99.922
77.211
77.000
76.789
55.849
55.189
41.353
37.131
36.939
36.757
36.566
O
Ph
MeO
O
CF3
OMe
4z
13C NMR (151 MHz, CDCl3)
190.0 180.0 170.0 160.0 150.0 140.0 130.0 120.0 110.0 100.0 90.0 80.0 70.0 60.0 50.0 40.0 30.0 20.0 10.0 0
X : parts per Million : Carbon13

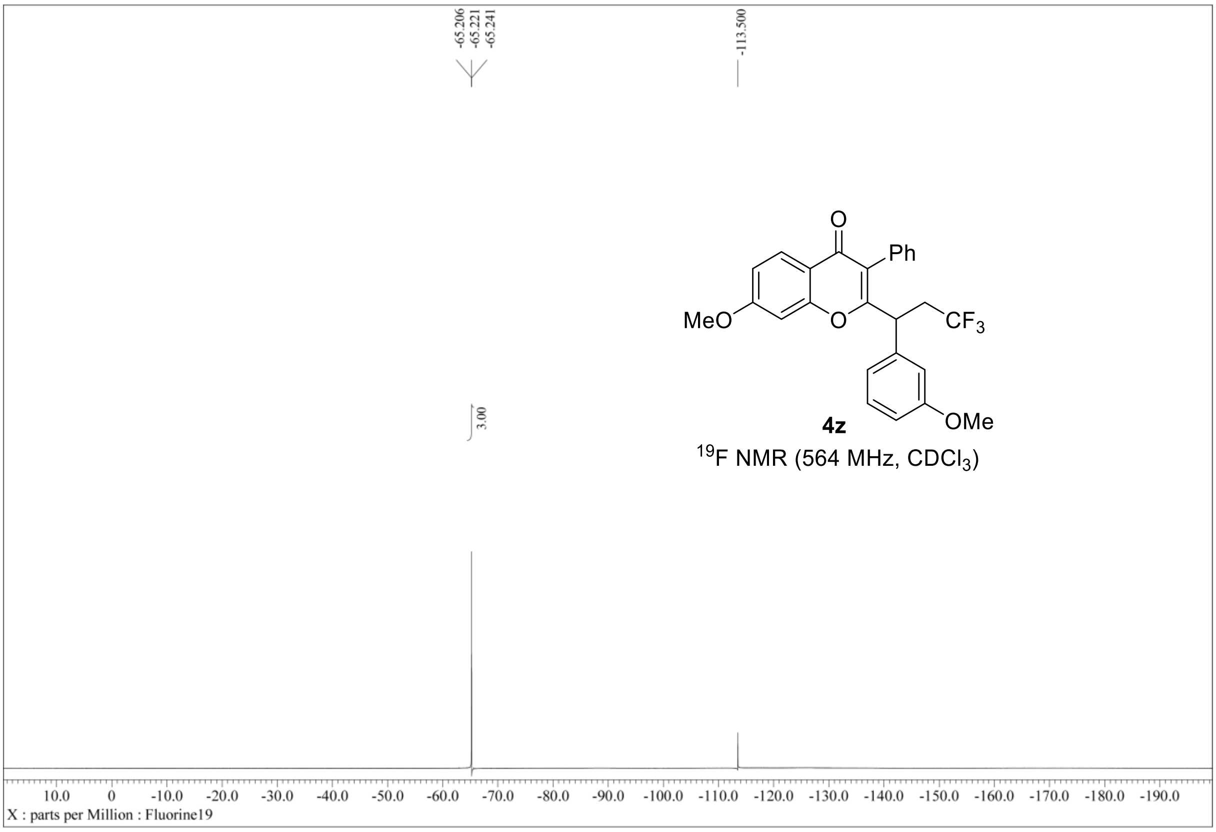
-65.206
-65.221
-65.241
-113.500
O
Ph
MeO
O
CF3
OMe
4z
19F NMR (564 MHz, CDCl3)
3.00
10.0 0 -10.0 -20.0 -30.0 -40.0 -50.0 -60.0 -70.0 -80.0 -90.0 -100.0 -110.0 -120.0 -130.0 -140.0 -150.0 -160.0 -170.0 -180.0 -190.0
X : parts per Million : Fluorine19

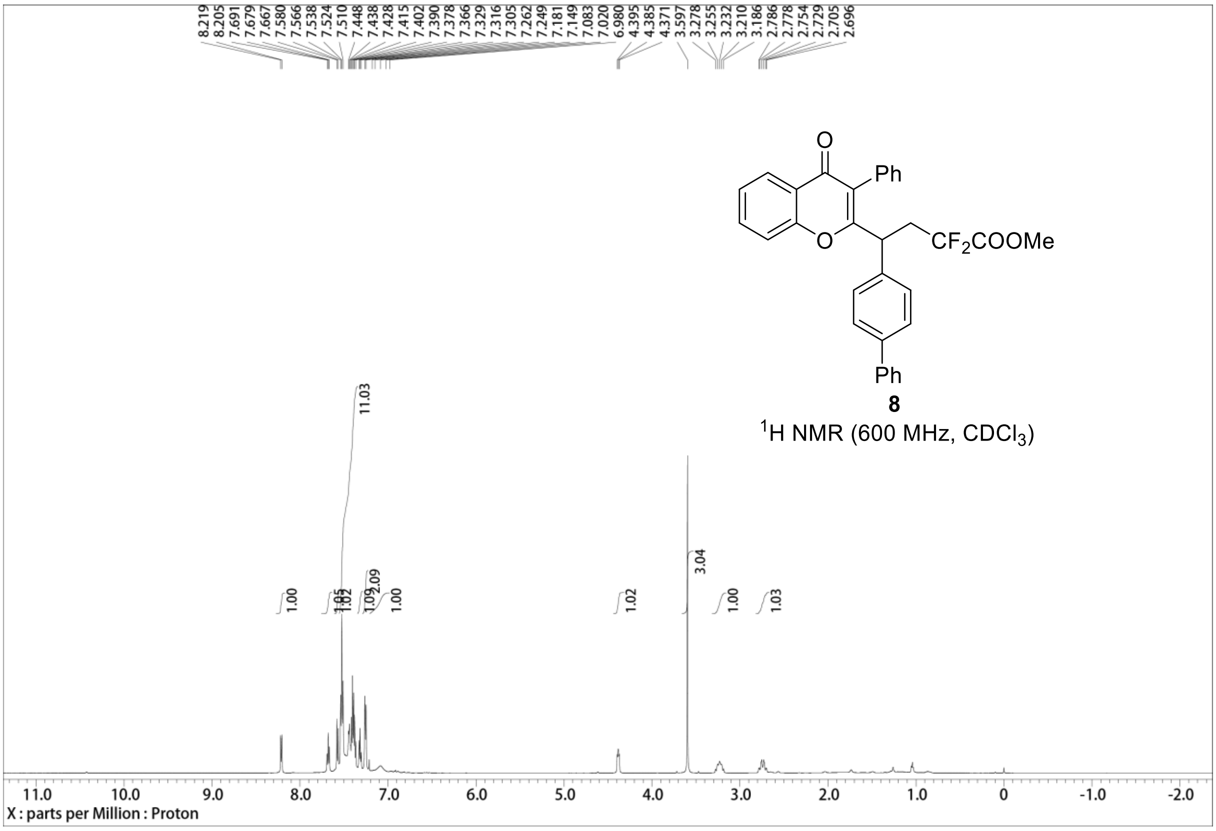
8.219
8.205
7.691
7.679
7.667
7.580
7.566
7.538
7.524
7.510
7.448
7.438
7.428
7.415
7.402
7.390
7.378
7.366
7.329
7.316
7.305
7.262
7.249
7.181
7.149
7.083
7.020
6.980
4.395
4.385
4.371
3.597
3.278
3.255
3.232
3.210
3.186
2.786
2.778
2.754
2.729
2.705
2.696
O
Ph
O
CF2COOMe
Ph
8
1H NMR (600 MHz, CDCl3)
11.03
3.04
1.00
1.05
1.02
1.09
2.09
1.00
1.02
1.00
1.03
11.0
10.0
9.0
8.0
7.0
6.0
5.0
4.0
3.0
2.0
1.0
0
-1.0
-2.0
X : parts per Million : Proton

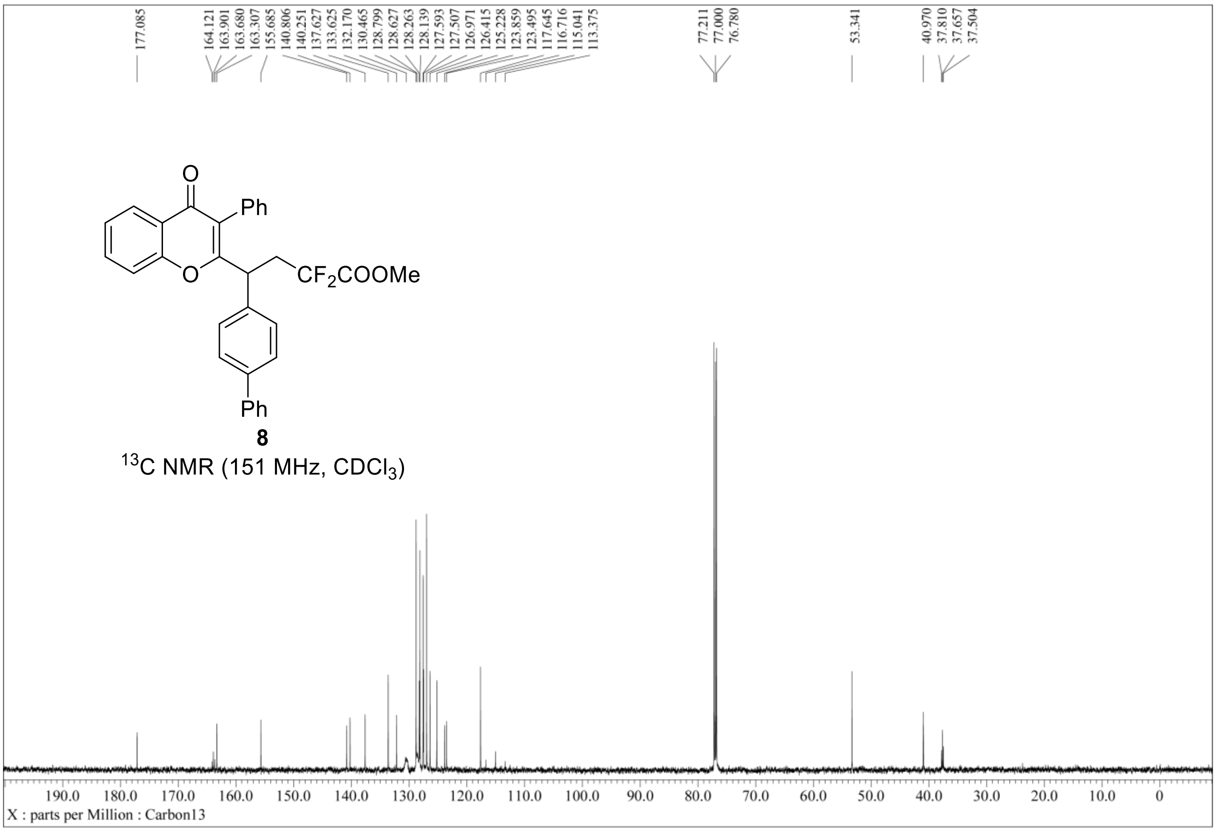
177.085
164.121
163.901
163.680
163.307
155.685
140.806
140.251
137.627
133.625
132.170
130.465
128.799
128.627
128.263
128.139
127.593
127.507
126.971
126.415
125.228
123.859
123.495
117.645
116.716
115.041
113.375
77.211
77.000
76.780
53.341
40.970
37.810
37.657
37.504
O
Ph
O
CF2COOMe
Ph
8
13C NMR (151 MHz, CDCl3)
190.0
180.0
170.0
160.0
150.0
140.0
130.0
120.0
110.0
100.0
90.0
80.0
70.0
60.0
50.0
40.0
30.0
20.0
10.0
0
X : parts per Million : Carbon13

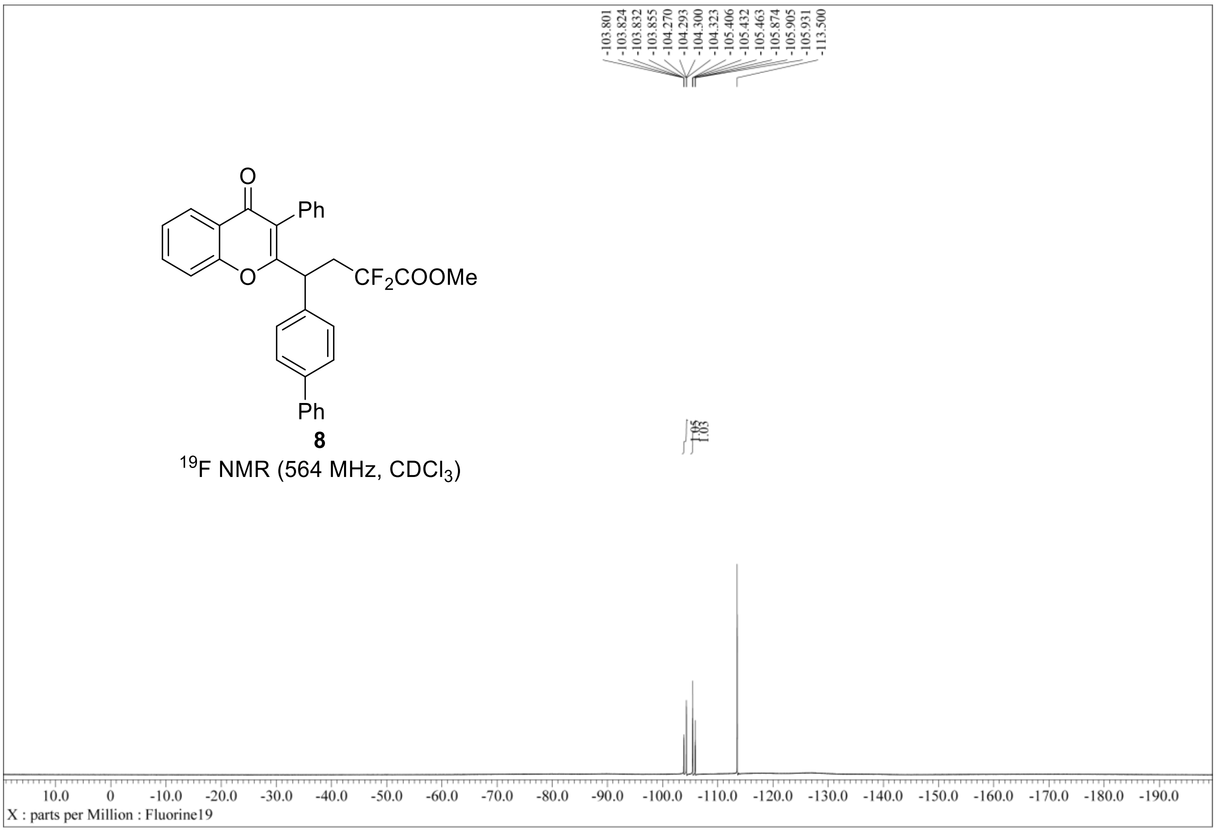
-103.801
-103.824
-103.832
-103.855
-104.270
-104.293
-104.300
-104.323
-105.406
-105.432
-105.463
-105.874
-105.905
-105.931
-113.500
Ph
CF2COOMe
Ph
8
19F NMR (564 MHz, CDCl3)
1.05
1.03
X : parts per Million : Fluorine19

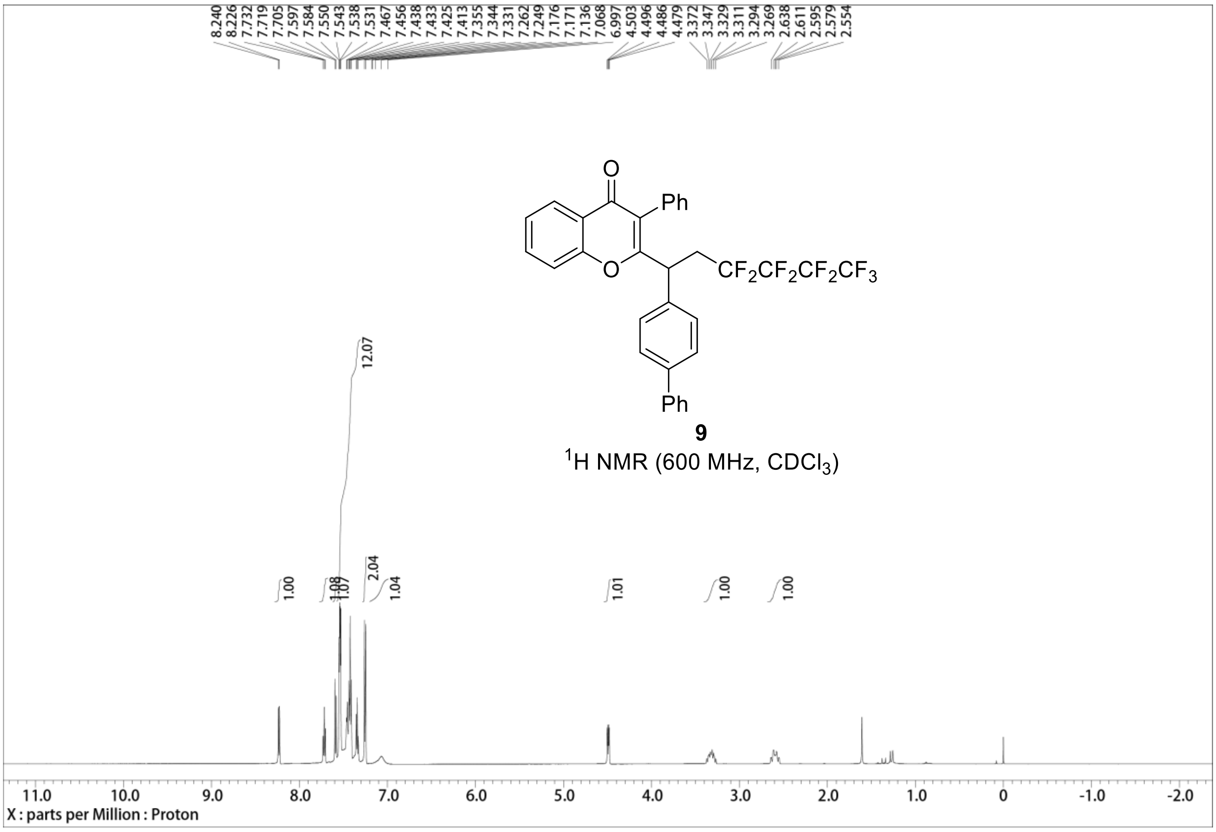
8.240
8.226
7.732
7.719
7.705
7.597
7.584
7.550
7.543
7.538
7.531
7.467
7.456
7.438
7.433
7.425
7.413
7.355
7.344
7.331
7.262
7.249
7.176
7.171
7.136
7.068
6.997
4.503
4.496
4.486
4.479
3.372
3.347
3.329
3.311
3.294
3.269
2.638
2.611
2.595
2.579
2.554
Ph
CF2CF2CF2CF3
Ph
9
1H NMR (600 MHz, CDCl3)
12.07
1.00
1.08
1.07
2.04
1.04
1.01
1.00
1.00
X : parts per Million : Proton

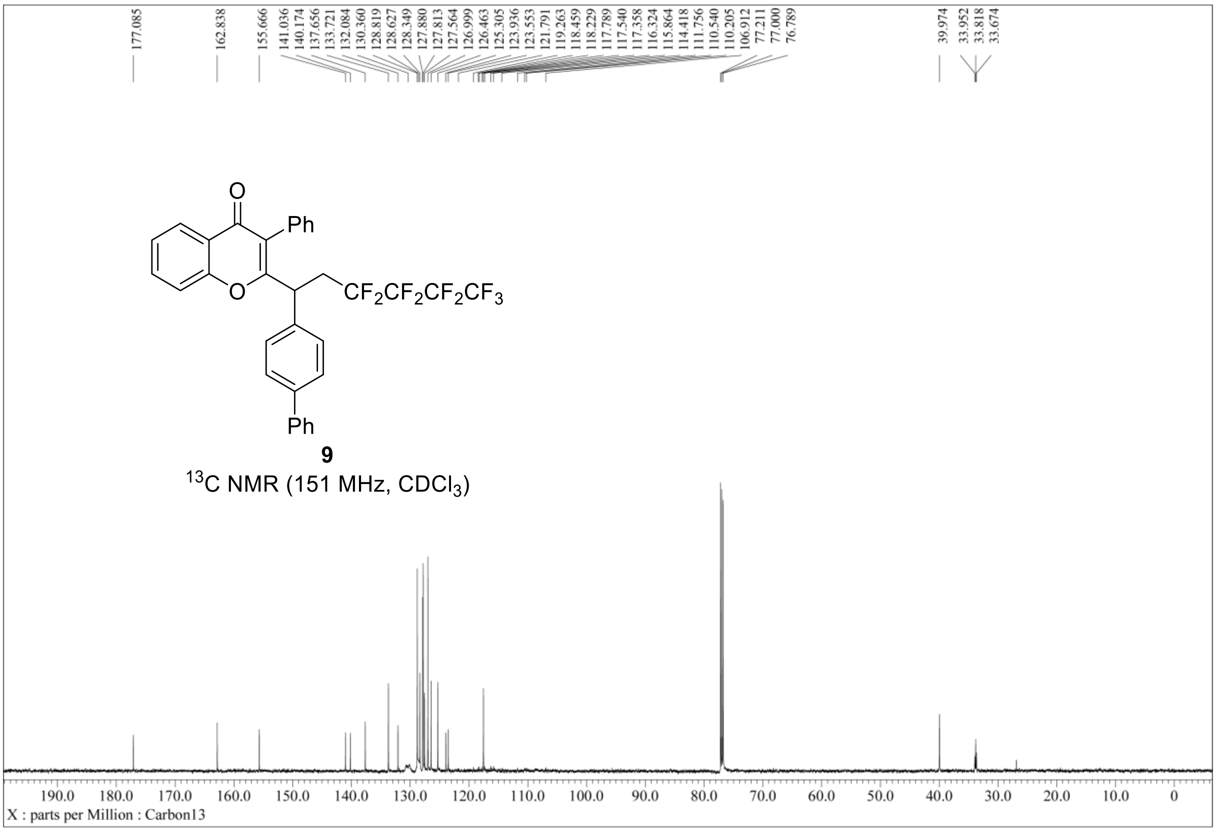
177.085
162.838
155.666
141.036
140.174
137.656
133.721
132.084
130.360
128.819
128.627
128.349
127.880
127.813
127.564
126.999
126.463
125.305
123.936
123.553
121.791
119.263
118.459
118.229
117.789
117.540
117.358
116.324
115.864
114.418
111.756
110.540
110.205
106.912
77.211
77.000
76.789
39.974
33.952
33.818
33.674
O
Ph
O
$CF_2CF_2CF_2CF_3$
Ph
9
^{13}C NMR (151 MHz, $CDCl_3$)
190.0 180.0 170.0 160.0 150.0 140.0 130.0 120.0 110.0 100.0 90.0 80.0 70.0 60.0 50.0 40.0 30.0 20.0 10.0 0
X : parts per Million : Carbon13

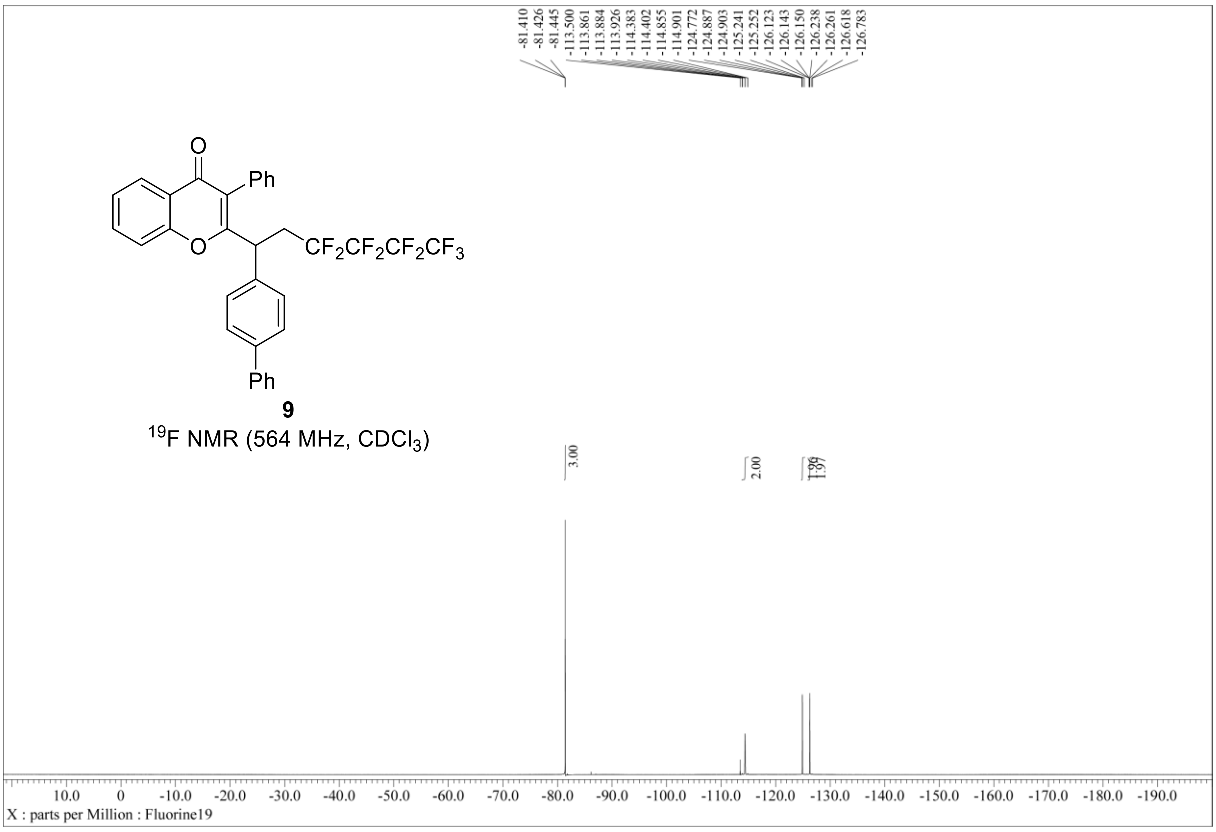
-81.410
-81.426
-81.445
-113.500
-113.861
-113.884
-113.926
-114.383
-114.402
-114.855
-114.901
-124.772
-124.887
-124.903
-125.241
-125.252
-126.123
-126.143
-126.150
-126.238
-126.261
-126.618
-126.783
O
Ph
O
$CF_2CF_2CF_2CF_3$
Ph
9
^{19}F NMR (564 MHz, $CDCl_3$)
3.00
2.00
1.97
10.0 0 -10.0 -20.0 -30.0 -40.0 -50.0 -60.0 -70.0 -80.0 -90.0 -100.0 -110.0 -120.0 -130.0 -140.0 -150.0 -160.0 -170.0 -180.0 -190.0
X : parts per Million : Fluorine19

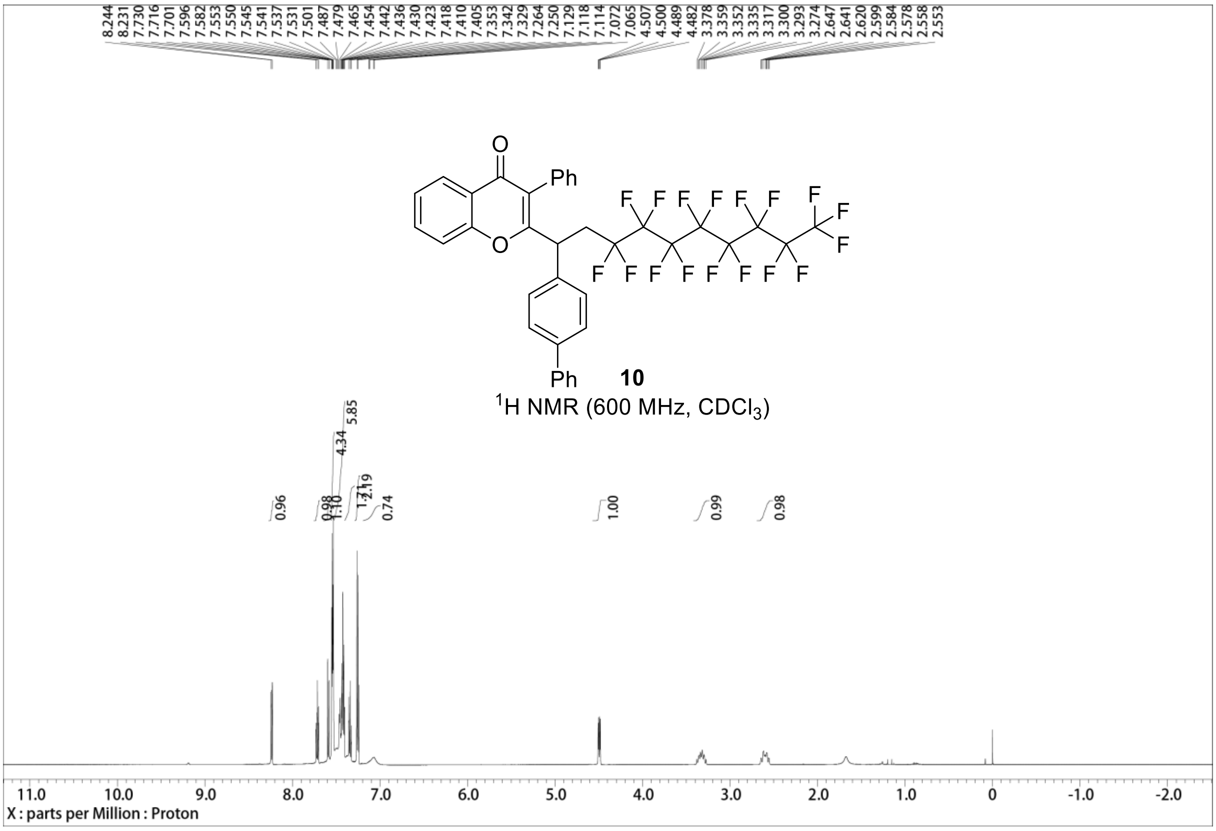
8.244 8.231 7.730 7.716 7.701 7.596 7.582 7.553 7.550 7.545 7.541 7.537 7.531 7.501 7.487 7.479 7.465 7.454 7.442 7.436 7.430 7.423 7.418 7.410 7.405 7.353 7.342 7.329 7.264 7.250 7.129 7.118 7.114 7.072 7.065 4.507 4.500 4.489 4.482 3.378 3.359 3.352 3.335 3.317 3.300 3.293 3.274 2.647 2.641 2.620 2.599 2.584 2.578 2.558 2.553
O
Ph
F F F F F F F F F F F F F F F F F
O
Ph
10
1H NMR (600 MHz, CDCl3)
0.96 0.98 1.10 4.34 5.85 1.19 2.11 0.74 1.00 0.99 0.98
11.0 10.0 9.0 8.0 7.0 6.0 5.0 4.0 3.0 2.0 1.0 0 -1.0 -2.0
X : parts per Million : Proton

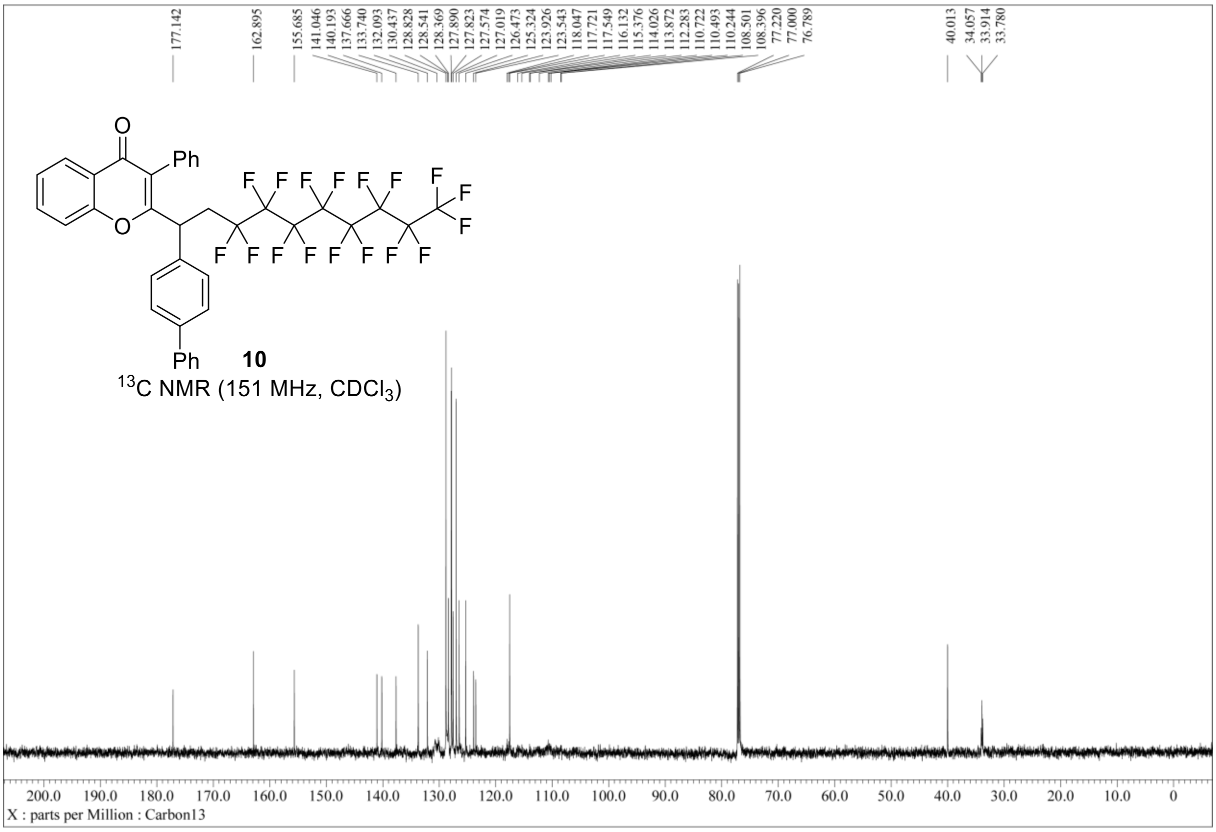
177.142 162.895 155.685 141.046 140.193 137.666 133.740 132.093 130.437 128.828 128.541 128.369 127.890 127.823 127.574 127.019 126.473 125.324 123.926 123.543 118.047 117.721 117.549 116.132 115.376 114.026 113.872 112.283 110.722 110.493 110.244 108.501 108.396 77.220 77.000 76.789 40.013 34.057 33.914 33.780
O
Ph
F F F F F F F F F F F F F F F F F
O
Ph
10
13C NMR (151 MHz, CDCl3)
200.0 190.0 180.0 170.0 160.0 150.0 140.0 130.0 120.0 110.0 100.0 90.0 80.0 70.0 60.0 50.0 40.0 30.0 20.0 10.0 0
X : parts per Million : Carbon13

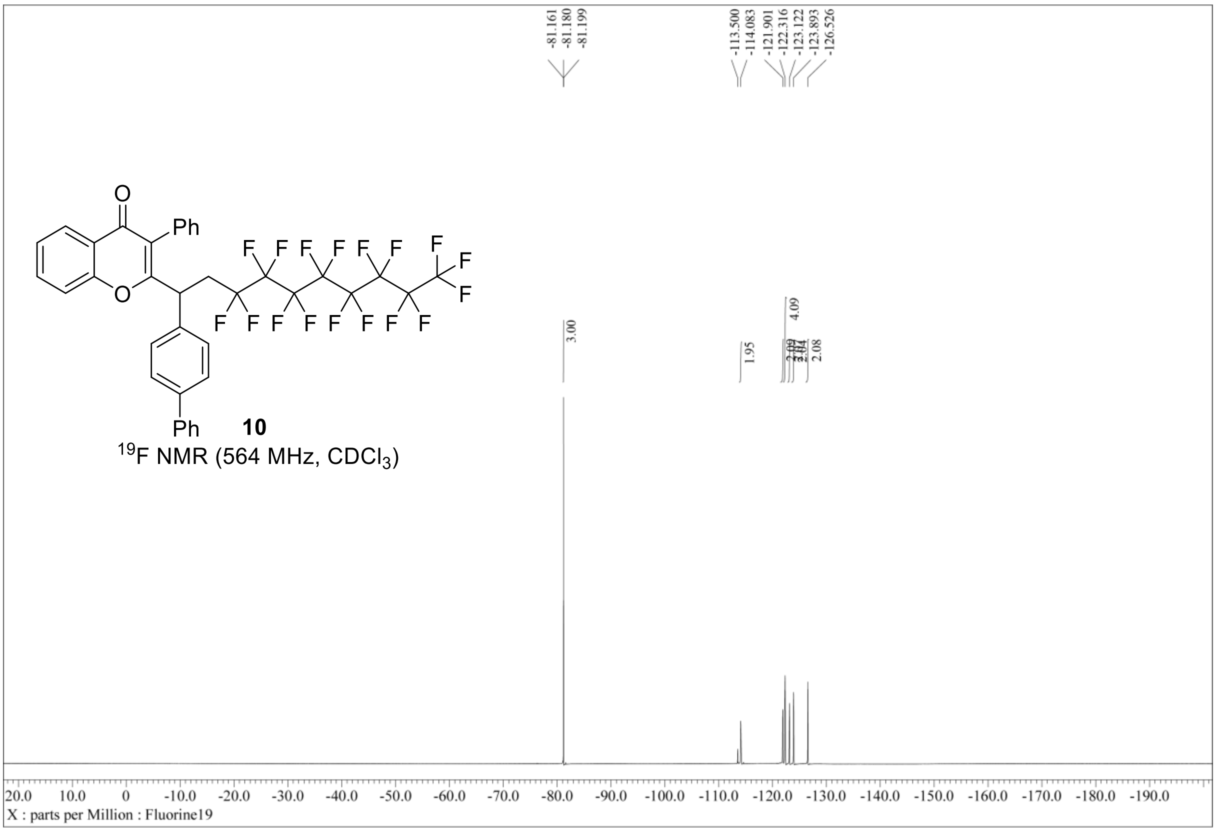

-81.161
-81.180
-81.199
-113.500
-114.083
-121.901
-122.316
-123.122
-123.893
-126.526
3.00
1.95
4.09
2.08
Ph
O
F
10
19F NMR (564 MHz, CDCl3)
20.0 10.0 0 -10.0 -20.0 -30.0 -40.0 -50.0 -60.0 -70.0 -80.0 -90.0 -100.0 -110.0 -120.0 -130.0 -140.0 -150.0 -160.0 -170.0 -180.0 -190.0
X : parts per Million : Fluorine19
